# Global coverage of interventions for reduction of injecting drug use-related harm, HIV, viral hepatitis and tuberculosis in prisons and other carceral settings: A systematic review

**DOI:** 10.1016/j.drugpo.2025.105069

**Published:** 2026-02-05

**Authors:** Thomas Santo, Matthew Hickman, Frederick L. Altice, Jason Grebely, Sophia Taylor, Michelle Lynch, Aleksa Kamenjaš, Jack Marsden, Lucy T. Tran, Paige Webb, Olivia Price, Christel Macdonald, Linda Montanari, Luis Royuela, Anja Busse, Jack Stone, Filipa Alves da Costa, Justin Berk, Colleen Daniels, Evan Cunningham, Peter Vickerman, Behzad Hajarizadeh, Keith Sabin, Annette Verster, Michael Farrell, Louisa Degenhardt

**Affiliations:** a National Drug and Alcohol Research Centre (NDARC), University of New South Wales Sydney (UNSW Sydney), Sydney, Australia; b Population Health Sciences, Bristol Medical School, University of Bristol, Bristol, United Kingdom; c Yale University, New Haven, United States; d Kirby Institute, University of New South Wales Sydney (UNSW Sydney), Sydney, Australia; e European Union Drugs Agency, Lisbon, Portugal; f Department of Mental Health, Brain Health and Substance Use, World Health Organization (WHO), Geneva, Switzerland; g University of Lisbon, Lisbon, Portugal; h Brown University, Providence, United States; i Joint United Nations Programme on HIV/AIDS (UNAIDS), Geneva, Switzerland; j Department of Global HIV, Viral Hepatitis and STI Programmes (HSS), World Health Organization (WHO), Geneva, Switzerland; k Médecins Sans Frontières (MSF) Access Asia Pacific, Kuala Lumpur, Malaysia

**Keywords:** Prisons, Harm reduction, Opioid agonist treatment, Needle and syringe programs, HIV, Hepatitis, Tuberculosis, Health services coverage

## Abstract

**Background:**

People who are incarcerated experience disproportionately high rates of injecting drug use and infectious disease, including HIV, viral hepatitis and tuberculosis. However, comprehensive global data regarding the availability of services that prevent and manage infectious disease, injecting drug use and related harms remain limited and outdated. We provide the first systematic review to comprehensively examine the availability and coverage of infectious disease prevention, treatment, and harm reduction services for incarcerated populations globally.

**Methods:**

We conducted a systematic review of evidence for provision of opioid agonist treatment (OAT), needle syringe programs (NSPs), HIV testing and antiretroviral therapy (ART), hepatitis C virus (HCV) testing and direct-acting antiviral (DAA) treatment, tuberculosis screening and treatment, hepatitis B virus (HBV) testing, treatment, and vaccination in carceral settings. We searched from peer-reviewed and grey literature databases between 2000 and 2025 and used the most recent data available for each indicator.

**Findings:**

OAT was documented in 59/207 countries (29 %), and NSPs in ten (5 %). HIV testing was documented in 86 countries (42 %) and ART in 79 countries (38 %). HCV testing was confirmed in 55 (27 %), with DAA treatment in 47 (23 %). HBV testing was identified in 51 countries (25 %), treatment in 36 (17 %), and vaccination in 41 (20 %). Tuberculosis screening was documented in 96 countries (46 %) and treatment in 81 (39 %). Fewer than 2 % (approximately 172,000) of the 11.3 million people incarcerated worldwide live in countries that offer OAT, NSPs, and treatment for HIV, HCV, HBV, and tuberculosis in at least one carceral facility. There is not a single country where incarcerated people have access to all such services in every facility. Programme level evidence was rarely available.

**Interpretation:**

The global shortage of services that prevent and treat infectious disease and harms related to injecting drug use in carceral settings is a critical public health issue and, compared with community standards, a breach of human rights. This study underscores the urgent need for international collaboration and policy reform to scale up and stabilise services that address the health needs of incarcerated populations, ultimately improving health outcomes for both incarcerated populations and wider community.

**Funding:**

Australian National Health and Medical Research Council.

## Introduction

Globally, an estimated 11.3 million people are detained in carceral settings—a rate of 221 per 100,000 among individuals aged 15–64 years (Degenhardt, 2025). People who are incarcerated bear a disproportionate burden of morbidity (Kinner & Young, 2018; Kronfli et al., 2025) and mortality (Borschmann et al., 2024), particularly from HIV, hepatitis C virus (HCV), hepatitis B virus (HBV), and tuberculosis (Degenhardt, 2025; Dolan et al., 2016). Shared risk factors for incarceration and infectious disease, such as socioeconomic disadvantage, a history of sex work (Aldridge et al., 2018), and injecting drug use, which is 53 times more common among incarcerated people than in the general population (Aldridge et al., 2018; Degenhardt, 2025), as well as structural conditions including overcrowding and inadequate healthcare (Puglisi & Wang, 2021; Walter et al., 2021). Together, these conditions create high-risk environments that drive infectious disease transmission, endangering both incarcerated individuals and the broader community (Altice et al., 2016).

Despite the high burden of HIV, tuberculosis, and viral hepatitis, prevention, testing, and treatment services, data on the availability and coverage of effective services in carceral settings have never been comprehensively collated. For example, existing reports and reviews on the availability and coverage of interventions that prevent and treat HIV, tuberculosis, and viral hepatitis (Emanuele, 2005; Haeusler et al., 2022; Kronfli et al., 2025; Rumble et al., 2015; Smith et al., 2017; Zampino et al., 2015) often exclude key data sources, such as grey literature (Kronfli et al., 2025; Rumble et al., 2015; Smith et al., 2017; Zampino et al., 2015), and rely on outdated evidence (Zampino et al., 2015). National and regional coverage data are required to inform public health strategies and evaluate progress towards multiple international health and human rights commitments (United Nations Office on Drugs & Crime, 2015), including the United Nations Sustainable Development Goals (United Nations, 2025).

We provide the first global comprehensive assessment of the availability and coverage of services that treat opioid dependence and reduce the harms associated with injecting drug use, including opioid agonist therapies (OAT) and needle and syringe programs (NSPs), and preventing and treating infectious diseases (HIV, HBV, HCV, and tuberculosis) in carceral settings. We define carceral settings to include prisons, jails, or other detention facilities, excluding compulsory detention or detoxification sites for people who use drugs, which are inconsistent with WHO and UN guidance on voluntary, evidence-based treatment for drug treatment and lie outside the scope of this review. Specifically, this review aims to: document the availability of each service in carceral settings; collate coverage data for each service; and review availability and coverage at the regional and global levels.

## Methods

The methods used were consistent with previous global reviews (Degenhardt et al., 2017; Mathers et al., 2008; Nelson et al., 2011) and in accordance with PRISMA (Moher et al., 2009) and GATHER (Stevens et al., 2016, in press) guidelines (Appendix 1). The review protocol was registered on PROSPERO (CRD42023425532). There were no limitations on languages.

### Search strategy and selection criteria

Searches of five electronic peer-reviewed literature databases (Medline, EMBASE, PsycINFO, Web of Science and CINAHL) were undertaken, using a comprehensive set of search terms developed in consultation with a specialist drug and alcohol librarian (see Appendix 2). Searches of peer-reviewed literature were conducted on March 7th–10th 2023, and were limited to those published from January 1st, 2000 onwards. An updated search was done on June 2nd 2025, and was limited to reports published between March 10th 2023 and June 2nd, 2025. Relevant systematic reviews were excluded but were hand-searched for relevant original papers/reports within them.

We used built-in search functions and Google Advanced Search to explore grey literature and online databases relevant to incarcerated populations. Our initial searches took place in October 2023, continuing until December 2024. National websites for governmental agencies, global and regional NGOs, harm reduction organisations, and national health departments were systematically reviewed for relevant documents. Health-focused websites (including those of NGOs, harm reduction organisations, national health departments, and blood-borne virus treatment programs) were searched using incarceration-related terms, while criminal legal–focused websites were searched using health-related terms. All searches were conducted in English and other local languages, aided by Google Translate. Refer to Appendix 3 for the list of websites (see also Ottaviano, et al., 2023).

Key documents and data from relevant international agencies were retrieved from the European Union Drug Agency (EUDA), the Global Fund, Harm Reduction International (HRI), UNAIDS, the UN Office on Drugs and Crime (UNODC), and the World Health Organization (WHO). We also reviewed Harm Reduction International’s Global State of Harm Reduction reports (Harm Reduction International, 2021), and UNODC’s World Drug Reports (United Nations Office on Drugs & Crime, 2022), among others. Reports were obtained from EUDA, UNAIDS, UNODC, WHO and WHO/Europe staff, and we liaised with these organisations throughout the review. In parallel, we contacted the Global HCV and HIV Treatment Restrictions Group (166 experts across 160 countries, including ministry representatives) to request country-level documentation and clarifications, and consulted experts involved in prior well-cited global coverage and data-guidance work (e.g., Colledge--Frisby et al., 2023; Marshall et al., 2024) to identify additional sources and verify interpretations.

We relied solely on publicly accessible, verifiable documentation and followed established approaches used in global coverage assessments (Colledge-Frisby et al., 2023; Larney et al., 2017; Marshall et al., 2024). We document three evidence streams: (1) systematic searches of the peer-reviewed literature; (2) structured searches of national government websites across health and justice sectors (grey literature; multilingual where needed); and (3) direct engagement with global and regional agencies (EUDA, UNAIDS, UNODC, WHO and WHO/Europe, HRI) and international experts.

Data were requested via an email distribution process and through social media. We began by sending initial emails to key experts and organisations and posting announcements on X (formerly Twitter) and Facebook (Appendix 4). Searches and contacts with country experts and other key agencies continued until December 2024.

### Screening, data extraction and analysis

Studies were eligible if they reported availability of OAT, NSPs, HIV testing or treatment, HCV testing or treatment, tuberculosis screening, or HBV vaccination, testing, or treatment. We prioritised national over subnational sources. All identified documents were catalogued in an Endnote (version 20) library for initial de-duplication of publications. Records identified through database searches were imported into Covidence (https://www.covidence.org) (Veritas Health Innovation, 2024) for screening.

Two reviewers independently screened titles, abstracts, and full texts, with discrepancies resolved by consensus with a third reviewer. Full details of the screening process and the names of reviewers involved at each stage are provided in Appendix 5.1. We had members proficient in reading English, French, Serbo-Croatian, Portuguese, and Spanish; other languages were read via Google Translate or the Microsoft Word translate function.

Data from eligible studies were extracted into a purpose-built database in REDCap (https://projectredcap.org) (Harris et al., 2019, 2009) at country level and double-checked for accuracy. Countries and regional groupings were based upon those used by UNAIDS, WHO and UNODC, consistent with our previous studies (Colledge-Frisby et al., 2023; Degenhardt et al., 2017, 2023; Larney et al., 2017).

We established data availability for each service: OAT for opioid dependence; NSPs providing sterile injection equipment; HIV testing and antiretroviral therapy; HCV testing and direct acting antiviral treatment; any form of screening for tuberculosis (e.g. symptom screening, chest x ray, sputum analysis) or tuberculosis treatment; and HBV vaccination, testing, and treatment. For HIV, HCV and HBV testing meant diagnostic services delivered to individuals, including provider initiated and client initiated testing and self-testing where applicable, with the purpose of confirming infection and enabling linkage to care (World Health Organization, 2009, 2017). For tuberculosis, we assessed systematic screening of custodial populations, including screening at entry or periodic population based screening that used routine symptom checks, chest X ray, or other clearly specified routine approaches, as recommended by the World Health Organization (Charalambous et al., 2023; Narayan et al., 2023). Sputum/Xpert/culture/IGRA/TST are diagnostic confirmation, not “screening,” and are outside the screening indicator unless the source explicitly states they are used as part of a systematic screening program (e.g., entry screening uses CXR then Xpert for positives). Notification datasets, including from a global surveillance synthesis (Martinez et al., 2023), were not treated as evidence of screening delivery unless the source explicitly described a systematic prison-screening program, for example entry or periodic screening. In the absence of such documentation, notifications and outcomes were treated as surveillance outputs and not as evidence of screening-delivery availability.

We counted availability where there was evidence of routine service availability at the time of the most recent source, provided to people while in carceral settings, whether delivered within facilities (for example, in reach services or telemedicine) or by transfer to a community facility for standard care. Pilot or time-limited demonstration programmes and reports, guidelines or strategies to improve service coverage were not considered to describe service availability.

Service availability was categorised as “All carceral settings” to address the issue of reach where provision was confirmed in every correctional facility nationwide. “One or more carceral setting” was used where availability was confirmed in at least one facility, including jurisdictions where universal provision could not be verified. “Not available” indicated confirmed absence across all facilities, and “Unknown” was applied where data were insufficient to determine availability. If sources offered comprehensive, national-level data on the number of facilities delivering a service, we recorded those facility-level statistics separately. Additional information is in Appendix 5.

We also extracted programmatic coverage data, which included the number of individuals who received a given intervention, the volume of tests or treatments administered (e.g., HCV RNA testing, HBV vaccinations), or the amount of equipment provided (e.g., needles-syringes in an NSP). When studies reported proportions rather than absolute figures (e.g., the percentage of incarcerated individuals tested for HCV), we noted these percentages where we had a denominator available. For each programmatic coverage data point, we recorded the timeframe (snapshot or past year) and the collection year.

For each service, data on availability and programmatic coverage data were obtained from all relevant sources, prioritising national-level data and reporting years over publication dates (only national-level data was used for programmatic coverage data). When necessary, multiple sources were combined to ensure the most current and comprehensive data for each specific outcome, such as integrating client-level data from one source with facility-level data from another for the same service and country. Further details regarding our decision rules and the prioritization of data appear in Appendix 5. All national estimates are based on country-specific data from published or grey literature or verified personal communication. Regional or multi-country sources were not used to impute national availability. Regional estimates were calculated by summing the number of countries with documented availability in that region or by aggregating their incarcerated populations, ensuring that all summaries are grounded in verifiable national data.

### Study quality

Established risk of bias assessment tools were not feasible to conduct for this review, as we report availability of the specified interventions (e.g. OAT, NSP) at a country level, rather than at a study level. Additionally, extracted data were limited to whether specific interventions were reported as available in carceral settings and the year of their reported introduction, as well as facility and client level data for these interventions. Instead, we assess study quality by reporting on availability of the intervention for that country, recency of the source (defined by year of data collection reported by the source), source scope (whether the included source reported availability at a National or Subnational level), whether the source provided any client or facility level information, as well as the year reported for the facility or client numbers if the source reported these. The results of the study quality appraisal for each country by indicator are presented in Appendix 10.

## Data analysis

Data cleaning and analysis were conducted using Stata (version 14) and R (version 4.4.1; R Foundation for Statistical Computing, Vienna, Austria). We reviewed data on the number of countries with documented service availability and coverage data. A total of 207 countries were included in our review.

For each intervention, we estimated the proportion of the global incarcerated population with potential access to each of the key treatment interventions for infectious disease and OAT and NSPs using estimates for people incarcerated aged 15–64 years (Degenhardt, 2025). We attempted to estimate coverage using programmatic data on client numbers or equipment distributed using methodologies established by previous reviews of infectious disease and harm reduction service provision in the community for people who inject drugs (Colledge-Frisby et al., 2023; Larney et al., 2017). Coverage estimates were calculated by dividing the number of people accessing services by the estimated population in need (e.g., people who inject drugs for OAT and NSPs). Due to limited availability of programmatic data, however, coverage estimates could only be generated for OAT in countries with sufficient client-level data. Full methodological details are provided in Appendix 5.2.

We generated composite measures for the key interventions (OAT, NSP, HIV treatment, HCV treatment, HBV treatment, and tuberculosis treatment) in two ways: one composite including NSP and one excluding NSP, as there are limited prison based NSPs (Kronfli et al., 2025). In both cases, if any service within the composite was available in one or more facility (rather than universally), that country’s population was labelled as lacking evidence of comprehensive provision. Intervention availability was visualised using maps generated with the ggplot2 package (Wickham, 2016) in R, while the overlap of service combinations was illustrated with an UpSet plot created using the UpSetR package (Lex et al., 2014) (Fig. 3). Bar charts display these estimates using the global incarcerated population as the denominator (Fig. 4).

### Role of funding source

The funders had no role in the design, conduct, analysis or interpretation of findings.

## Results

**Fig. 1.**
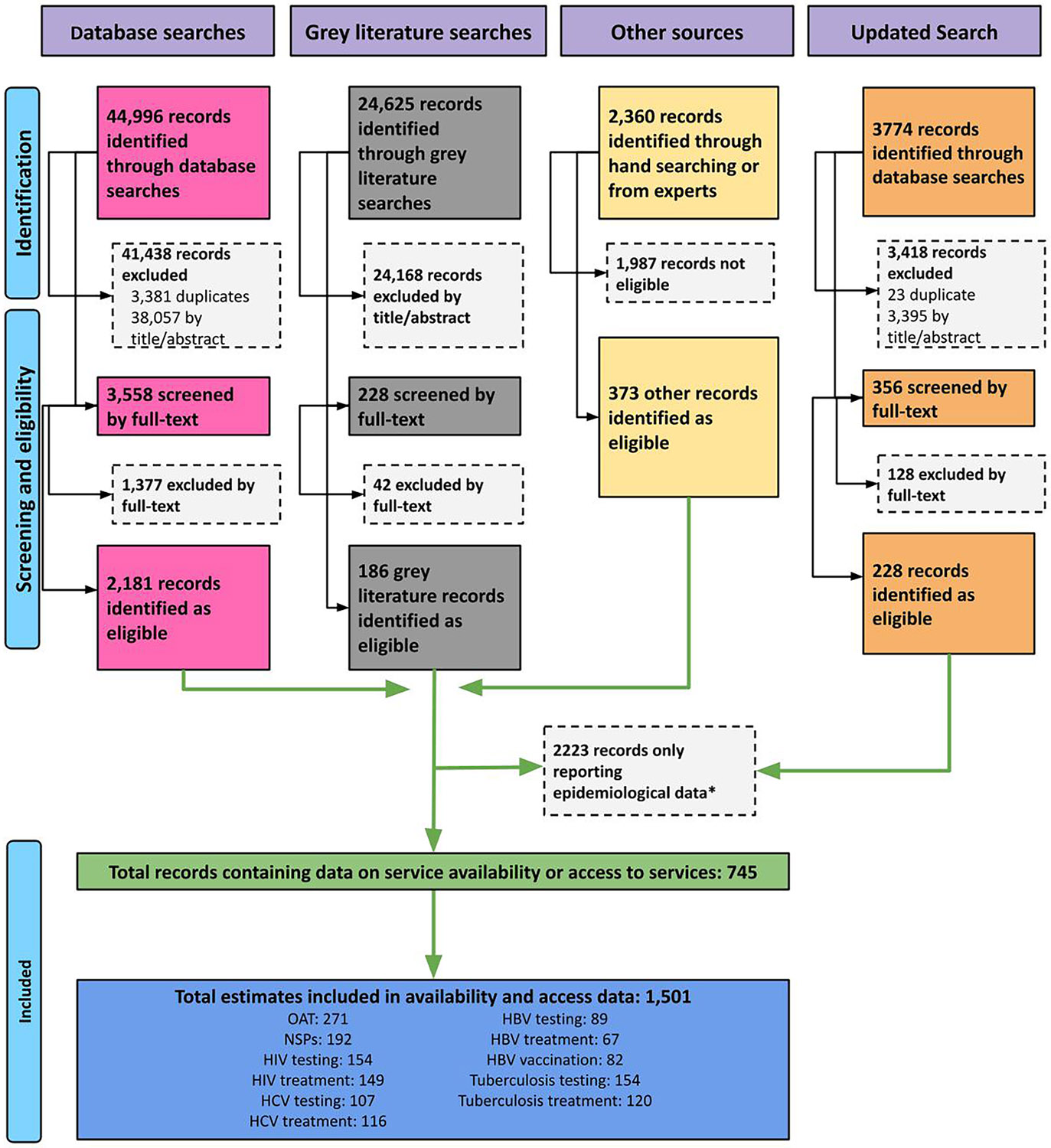
PRISMA Flowchart of studies included in the current review. Note: The search strategy was used to generate estimates for multiple reviews, the number of documents identified as eligible by the search and excluded here includes records used in epidemiological analyses which were not eligible for the current review.

Among the 75,755 records screened (2000–2025) followed by full-text review, 745 records were eligible for data extraction. As displayed in Fig. 1, 1501 estimates were included to provide the most recent and robust data on at least one outcome of interest in carceral settings across 207 countries.

Table 1 presents the number and proportion of countries in each region where interventions are available in one or more carceral facility, in all carceral facilities, in no facilities, or where availability is unknown. In parallel, the table reports the proportion of the regional and global incarcerated population residing in countries within each of these categories.

Programmatic data on the number of clients or equipment distributed were unavailable for most interventions. This prevented global coverage estimation. Opioid agonist treatment (OAT), although the most frequently reported intervention, remained uncommon. Only 33 countries reported the number of clients receiving OAT. Coverage estimates could be calculated for just 19 countries that also had population prevalence estimates of people who inject drugs in carceral settings (Degenhardt, 2025). **See** Appendix 6 for by-country tables and available coverage data for each intervention.

### Opioid agonist treatment (OAT)

Evidence of OAT was documented in one or more carceral setting in 59 (29 %) countries; 20 (10 %) countries had OAT available in all carceral settings (covering 5 % of the global incarcerated population). OAT was unavailable in carceral settings in 124 (60 %) countries and unknown in 24 (12 %) countries (Table 1; Fig. 2a).

Programmatic data on the number of clients receiving OAT were located for 33 countries (16 %). Of the 22 countries that reported programmatic data and had an estimate of the number of people who inject drugs (the denominator), 16 of 21 (76 %) were from Western Europe; 13 of these had coverage fewer than 40 people on OAT per 100 people who inject drugs. Appendix Table 6.1 shows country-level availability of OAT in more detail.

### Needle and syringe programs (NSPs)

Evidence of NSPs were documented in one or more carceral setting in ten (5 %) countries; only three (1 %) countries, Luxembourg, San Marino and Spain, had NSPs in all carceral facilities (covering 1 % of the global incarcerated population) (Table 1; Fig. 2b). NSPs were unavailable in carceral settings in 162 (78 %) countries and availability was unknown in 35 (17 %) countries. Data on the number of needles distributed were reported in five countries (2 %), whereas the number of individuals accessing NSPs was only reported in three countries (1 %). See Appendix Table 6.2 for the country-level availability of NSP in more detail.

### HIV testing and treatment

Evidence of HIV testing was documented in one or more carceral setting in 86 (42 %) countries; in all carceral settings in 38 (18 %) countries (covering 31 % of the global incarcerated population). HIV testing was unavailable in one (<1 %) countries, and unknown in 120 (58 %) countries (Table 1). Data on the number of individuals accessing HIV testing in carceral settings were available in 11 (5 %) countries, with nine (4 %) countries providing data on the number of HIV tests distributed.

Evidence of HIV treatment was documented in one or more carceral setting in 79 (38 %) countries and in all carceral settings in 22 (11 %) countries (covering 31 % of the global incarcerated population). HIV treatment was unavailable in two (1 %) countries and unknown in 126 (61 %) countries (Table 1; Fig. 2c). Data on the number of individuals accessing HIV treatment in carceral settings was available in 36 countries (17 %). See Table 1 for regional variation and Appendix Tables 6.3 and 6.4 for the country-level availability of HIV testing and HIV treatment. Around 3492,063 individuals (30.8 %) are in countries with universal HIV treatment provision, whereas 3881,847 (34.3 %) are in countries where one or more, but not all facilities offer HIV treatment.

### Hepatitis C testing and treatment

Evidence of HCV testing was documented in one or more carceral setting in 55 (27 %) countries and in all carceral settings in 30 (15 %) countries (covering 13 % of the global incarcerated population). HCV testing was unknown in 152 (73 %) countries, whereas no countries reported it as unavailable (Table 1). Unknown evidence of HCV testing was documented in Central Asia, the Caribbean, and Pacific Island States and territories. Data on the number of clients accessing HCV testing were only available for eight (4 %) countries and data on the number of HCV tests conducted in carceral settings were only available in four (2 %).

Evidence of HCV treatment was documented in one or more carceral setting in 47 (23 %) countries and in all carceral settings in 16 (8 %) countries (covering 2 % of the global incarcerated population). HCV treatment was unavailable in 13 (6 %) countries and unknown in 147 (71 %) countries (Table 1; Fig. 2d). In many regions, there was no evidence of HCV treatment being available in carceral settings including in East and South-East Asia, Central Asia, the Caribbean, Pacific Island states and territories, and Sub-Saharan Africa. Data on the number of people receiving HCV treatment in carceral settings were available in 29 (14 %) countries. See Appendix Tables 6.5 and 6.6 for the country-level availability of HCV testing and HCV treatment. Around 215,594 people (2 %) are in countries with HCV treatment availability in every facility, and 3739,344 (33 %) people in countries with partial availability.

### Hepatitis B virus testing, treatment and vaccination

Evidence of HBV testing was documented in one or more carceral setting in 51 (25 %) countries and all carceral settings in 26 (13 %) countries (covering 5 % of the global incarcerated population). HBV testing was unavailable in carceral settings in two (1 %) countries and was unknown in 154 (74 %) countries (Table 1). East and South-East Asia, Central Asia, the Caribbean, and Pacific Island states and territories had no evidence of HBV testing availability. Only three (1 %) countries had data on the number of individuals accessing HBV tests.

Evidence of HBV treatment was documented in one or more carceral setting in 36 (17 %) countries and all carceral settings in 11 (5 %) countries (covering 2 % of the global incarcerated population). HBV treatment was unavailable in carceral settings in two (1 %) countries and was unknown in 169 (82 %) countries (Table 1; Fig. 2e). Treatment availability varied by region, with Eastern Europe (*n* = 12; 71 %) reporting the greatest proportion of countries with availability in one or more carceral setting. Numerous regions reported either no or unknown availability of HBV treatment across East and South-East Asia, Central Asia, South Asia, the Caribbean, North America, the Pacific Island states and territories and Sub-Saharan Africa. Data on the number of individuals who received HBV treatment in carceral settings were available in 15 (7 %) countries. See Appendix Tables 6.7 and 6.8 for the country-level availability of HBV testing and HBV treatment. Hepatitis B treatment was confirmed to be provided in every facility for only 2 % of incarcerated people globally (229,179 people).

Evidence of HBV vaccination was documented in carceral settings in 41 (20 %) countries; 30 (14 %) countries had availability in all carceral settings (covering 5 % of the global incarcerated population). HBV vaccination was unavailable in carceral settings in seven (3 %) countries and was unknown in 159 (77 %) countries. See Appendix Tables 6.9.1 and 6.9.2 for complete details on regional and country-level availability of HBV vaccination.

### Tuberculosis

Evidence of tuberculosis screening and diagnosis was documented in one or more carceral setting in 96 (46 %) countries and all carceral settings in 42 (20 %) countries (covering 9 % of the global incarcerated population); unavailable in one (1 %) country and unknown in 110 (53 %) countries (Table 1). Six countries (3 %) had data on the number of people accessing tuberculosis screening in carceral settings.

Evidence of tuberculosis treatment was documented in one or more carceral setting in 81 (39 %) countries and all carceral settings in seven (3 %) countries (covering 1 % of the global incarcerated population). Tuberculosis treatment was unknown in 126 (61 %) countries, however no countries reported that tuberculosis treatment was unavailable in carceral settings (Table 1; Fig. 2f). Data on the number of people accessing tuberculosis treatment in carceral settings were available in 29 (14 %) countries. See Appendix Tables 6.10 and 6.11 for the country-level availability of tuberculosis screening and tuberculosis treatment. Tuberculosis treatment was confirmed to be provided in every facility for only 1 % of incarcerated people globally (128,355 people).

### Overlap of key treatment and harm reduction interventions in carceral settings

**Fig. 3.**
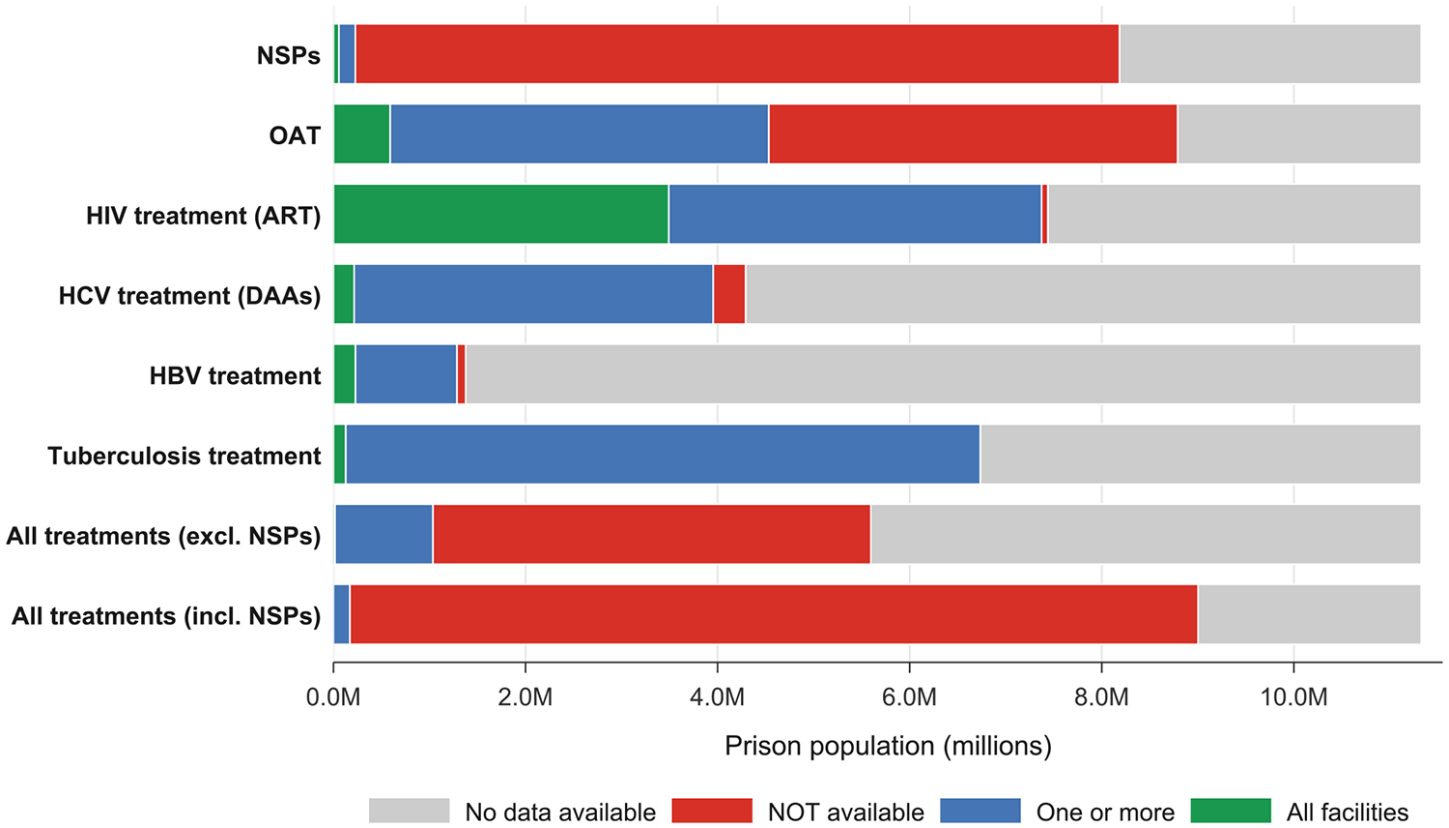
Global harm reduction and treatment service availability in carceral settings by total carceral population per country. Figure Notes: Prison population per country was used to determine the global availability of interventions. For “All Interventions” and “All Interventions but NSP,” orange represents populations in countries where at least one intervention (OAT, HIV/HCV/HBV, or tuberculosis treatment) is unavailable nationally, and light blue where one or more is in some facilities; Carceral Settings: Including prisons, jails or other carceral settings; Acronyms: OAT: Opioid Agonist Treatment; NSP: Needle & Syringe Programme(s); HIV: Human Immunodeficiency Virus; ART: Antiretroviral Therapy;; HCV: Hepatitis C; DAA: Direct-Acting Antiviral; HBV: Hepatitis B.

**Fig. 4.**
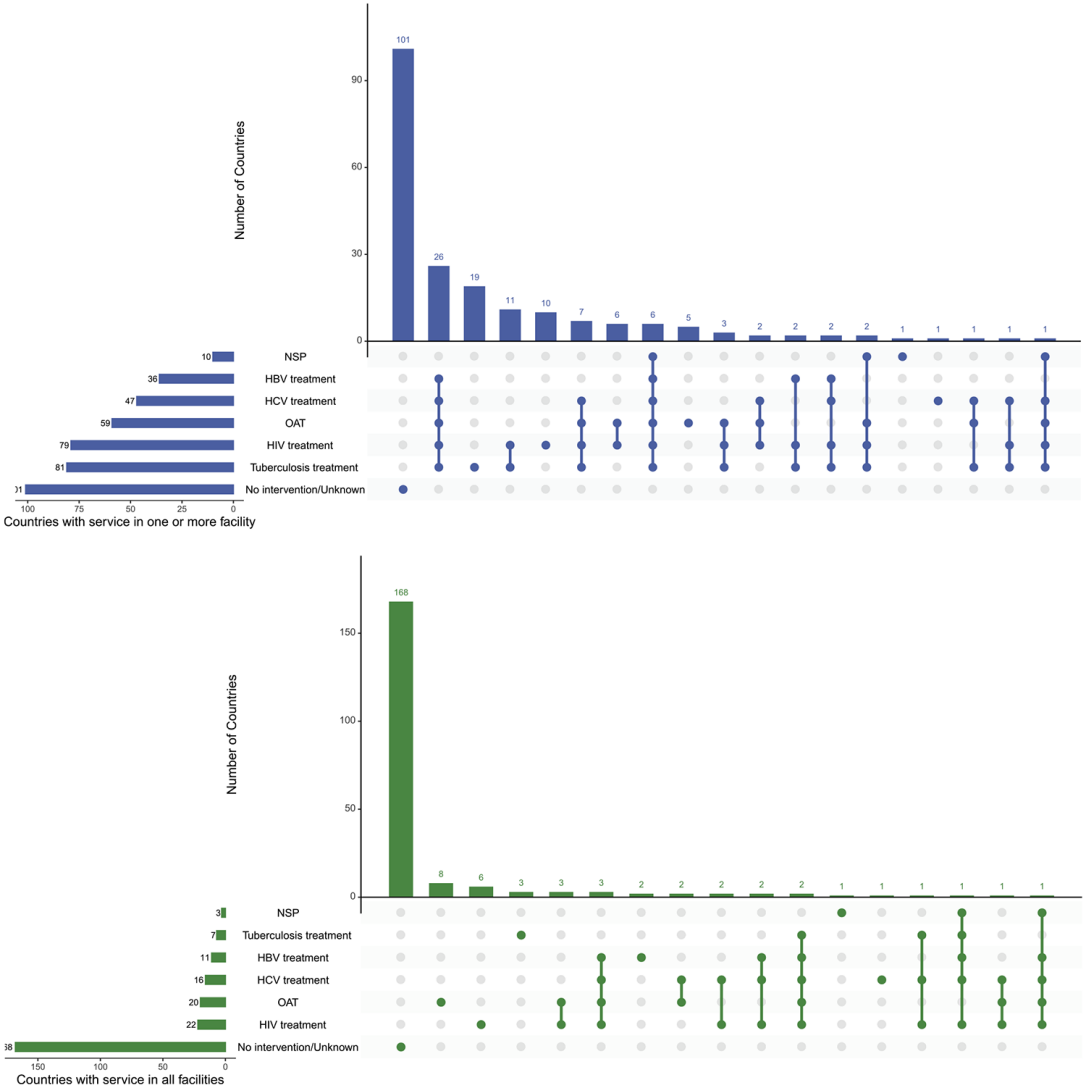
Overlap of key interventions and infectious disease treatment services in carceral settings by country. Figure Notes: Carceral Settings: Including prisons, jails or other carceral settings; Acronyms: OAT: Opioid Agonist Treatment; NSP: Needle & Syringe Programme(s); HIV: Human Immunodeficiency Virus; ART: Antiretroviral Therapy; HCV: Hepatitis C; HBV: Hepatitis B.

Figs. 3 and 4 present the global overlap in the availability of six essential interventions in carceral settings (OAT, NSPs, and treatment for HIV, HCV, HBV, and tuberculosis). As shown in Fig. 3, no incarcerated individuals reside in a country where all six interventions are available in every carceral facility.

Partial availability is also extremely limited. Based on documented sources, only 171,853 people (<2 % of the global incarcerated population) are in countries where all six interventions are available in one or more facility; for many countries, availability of one or more components is unknown, so these values represent minimum documented availability due to non-reporting. Access to OAT and HIV, tuberculosis, and hepatitis interventions is documented as partially available to approximately 1020,067 incarcerated individuals (covering 9.0 % of the global incarcerated population).

### Study quality and recency

Across interventions, most availability estimates were drawn from national-level data, though the proportion varied by service. OAT (86 %), NSPs (100 %), HIV testing (90 %), HCV testing (93 %), HCV treatment with DAAs (97 %), and HIV treatment (90 %) were almost entirely based on national sources. In contrast, HBV treatment (34 % national) and tuberculosis treatment (41 % national) more often relied on subnational or facility-level reporting. For all interventions, the majority of estimates were recent, with over three-quarters of records for HCV testing (89 %), HBV testing (81 %), HBV treatment (92 %), HCV treatment (87 %), OAT (92 %), NSP (97 %) and HBV vaccination (92 %) collected from 2020 onwards. Median data years clustered around 2021–2022 for most interventions, ranging from 2020 for tuberculosis treatment (IQR 2015–2020) to 2023 for NSP (2023–2023).

The availability of programmatic metrics varied considerably. The greatest number of countries reporting at least one measure of client enrolment or facility provision was reported for OAT with 68 %, and for HBV treatment the figure was 61 %. By contrast, HBV vaccination had no client counts and only 59 % of countries reported facility counts; HBV testing had 57 % reporting facility counts but only one country with a client count; tuberculosis screening (24 % any client/facility metric), HIV treatment (39 % any client/facility metric), TB treatment (41 % any client/facility metric) and NSP (40 % any client/facility metric) reported such metrics in fewer than half of countries with documented availability. Both HIV testing (51 % any client/facility metric), HCV treatment (52 % any client/facility metric) and HCV testing (55 % any client/facility metric) reported such metrics in about half of countries with documented availability. Facility counts were more frequently reported than client counts for testing and vaccination. However, many of these facility estimates came from countries that reported provision in all facilities.

Overall, the strongest and most up-to-date evidence was available for OAT, HCV testing and treatment, and HIV testing and treatment, which combined high proportions of national data with post-2020 collection. In contrast, tuberculosis treatment and HBV treatment were more often informed by older or subnational data and had more limited reporting of programmatic metrics. For each service, we present the recency of data, source scope (national vs. subnational), and availability of programmatic metrics by country in Appendix 10.

## Discussion

Incarcerated populations experience a disproportionately high burden of HIV, viral hepatitis, and tuberculosis, yet data on the availability and coverage of prevention, testing, and treatment services are largely absent and existing services remain inconsistently delivered. Based on documented sources, these interventions have been implemented in one or more carceral facility in countries accounting for 171,853 incarcerated individuals (<2 % of the global prison population), while availability remains unknown for many countries; therefore, these figures represent minimum documented availability. Our country classifications therefore differ from surveillance-based summaries (Martinez et al., 2023), which compile notifications among incarcerated people but do not document implementation of WHO-recommended screening programs in prisons. Of the 11.3 million people incarcerated worldwide, there are no people who are incarcerated in a country documenting availability of all six interventions (OAT, NSP, and treatment for HIV, HCV, HBV and tuberculosis) in every carceral facility (see Fig. 3). We provide clear evidence of a systemic global failure to provide essential services to a critical population, which highlights major deficiencies in healthcare access and human rights. This failure not only exacerbates social and health inequalities and perpetuates infectious disease transmission in carceral settings but also undermines efforts to reduce the transmission of these infections and is in contrast with WHO and UN recommendations (Larney et al., 2017; World Health Organization, 2022a).

In the community, OAT is available in 86 countries and NSPs in 92, compared to only 59 countries providing OAT and ten countries offering NSPs in one or more carceral facility (Colledge-Frisby et al., 2023), Limited programmatic coverage data largely precluded estimation of coverage using client numbers or needle distribution data as done in previous community-based reviews (Colledge-Frisby et al., 2023; Larney et al., 2017). Although coverage data for OAT are relatively more available than for other interventions, the information remains very sparse and, where documented, indicates coverage is low.

We have quantified elsewhere that the prevalence of infectious disease among people in carceral settings is also substantially higher than in the general population (Degenhardt, 2025), yet prevention, testing and treatment services are not available in many countries (Hajarizadeh et al., 2023). More than 190 countries have established community-based HBV vaccination programs with available programmatic data (Smith et al., 2017; World Health Organization & UNICEF, 2023); however, our review found limited evidence of their implementation in carceral settings, despite WHO guidelines identifying incarcerated populations as a priority group (World Health Organization, 2016).

Many individuals enter carceral settings already receiving OAT, ART, DAA and/or tuberculosis treatment in the community, yet access barriers during incarceration frequently result in treatment discontinuation and thus increase the risk of risky drug use, overdose, disease resistance, morbidity, and transmission (Rich et al., 2016). The impact of co-infections is exacerbated by these disruptions, as sustained treatment adherence is critical for disease management (Altice et al., 2016). Although updated guidelines provide improved clinical strategies (Godin et al., 2021; Hajarizadeh et al., 2021), limited programmatic data make it unclear whether these are being implemented in carceral settings.

### Global disparities in coverage

Access to services in carceral settings varies substantially across regions. Western Europe had the highest levels of harm reduction services documented in one or more carceral setting within countries for that region, with OAT and NSPs documented in one or more carceral facilities in countries comprising 100 % and 26 %, respectively, of the incarcerated population in Western Europe. Eastern Europe and Central Asia, where injecting drug use and HCV prevalence are among the highest globally among people who are incarcerated (Degenhardt, 2025), have OAT availability documented in all carceral settings in only 6 % and 0 % of countries, respectively (covering 1 % of people incarcerated in the Eastern Europe and 0 % of people incarcerated in Central Asia). In Latin America and East and Southeast Asia, where tuberculosis prevalence is high (Altice et al., 2016; Liu et al., 2024), evidence of tuberculosis treatment is not available in many countries, highlighting the urgent need for expanded services (Casper & Fitzmaurice, 2016). In Sub-Saharan Africa and the Middle East & North Africa, where HIV prevalence in prisons is among the highest globally (9.7 % and 6.8 %, respectively), there is minimal documented access to HIV testing and treatment (World Health Organization, 2022a).

Regional disparities in available data may reflect differences in how monitoring systems are established, maintained, and resourced, with timely and representative data collection depending on sustained investment and infrastructure (Colledge-Frisby et al., 2023). Countries with established NGO networks and regional collaborations (European Union Drug Agency, 2024, 2024) often have more comprehensive data on prison healthcare, even though far from exhaustive (Tavoschi et al., 2024; Winkelman et al., 2022), whereas under-resourced settings face structural limitations that constrain accurate reporting. Addressing these inequalities requires sustained investment and political will to better integrate healthcare services in carceral settings with those in the community, helping to reduce global disparities and accelerate progress toward disease elimination targets (Larney et al., 2017).

### Barriers to implementation and scale-up of services

This study reveals striking epidemiological and implementation-related gaps in the availability and reach of essential health services in carceral settings globally. People in prisons carry a disproportionately high burden of HIV, viral hepatitis, and tuberculosis (Degenhardt, 2025), yet access to prevention and treatment remains woefully inadequate. Our global review found that fewer than half of countries provide evidence for tuberculosis screening in prisons, and just over one-third provide evidence of treatment. For HBV and HCV, the gaps are even wider, with evidence of vaccination, testing, and treatment available in fewer than a quarter of countries. This systemic neglect not only threatens the health of incarcerated individuals but also undermines broader public health goals, as infectious diseases in prisons readily spread to communities.

Our findings underscore the chasm between intervention adoption and extended reach. We categorised countries with evidence of a service in “one or more” carceral facilities as having adopted the intervention. In contrast, countries where the service is available in all carceral facilities are considered to have achieved extended reach or comprehensive implementation. This distinction helps illuminate where opportunities for expansion remain.

The limited diffusion of evidence-based interventions like OAT and NSPs is particularly stark. Despite overwhelming evidence that OAT reduces HIV and HCV transmission, overdose and recidivism (Degenhardt et al., 2019), only 59 countries (comprising 40 % of the global incarcerated population) have confirmed provision in one or more carceral setting, and a mere 20 (comprising 5 % of the global incarcerated population) have extended it to all. NSPs, despite being recommended by WHO, are documented in one or more carceral setting in only 10 countries and are functionally non-existent for the vast majority of the 11.3 million people incarcerated worldwide.

These adoption-reach discrepancies mirror the epidemiological disconnect between disease burden and intervention availability, providing policy makers and practitioners guidance to address the implementation gap. Tuberculosis, for example, is often 10 to 30 times more prevalent in prisons than in the general population (Degenhardt, 2025) and in Latin America (Liu et al., 2024; Utpatel et al., 2024; Walter et al., 2022) and Southeast Asia, tuberculosis incidence in prisons is a significant driver of community transmission. Mathematical studies from Ukraine similarly support this finding (Altice et al., 2016). Despite this, tuberculosis interventions remain among the least available. Similarly, while Sub-Saharan Africa and the Middle East and North Africa have the highest HIV prevalence among incarcerated populations globally (Degenhardt, 2025), documented access to testing and treatment services remains minimal. Although 86 countries (covering 65 % of the global incarcerated population) have documented HIV testing in one or more prison, only 38 (covering 31 % of the global incarcerated population) have provision confirmed across all facilities. Tuberculosis screening is documented in 96 countries (comprising 87 % of the global incarcerated population), yet only 42 have reported full reach. Similarly, tuberculosis treatment was documented in 81 countries (covering 76 % of the global incarcerated population), with provision confirmed across all carceral settings in 7 countries; availability was unknown in 126 countries. These figures reflect weak institutionalisation of services and limited health system integration. The result is highly fragmented access, with incarcerated people’s ability to receive care often depending on which facility they are held in. These mismatches between disease burden and intervention coverage highlight critical implementation failures.

Moreover, the near-total absence of programmatic coverage data—such as the number of incarcerated individuals tested, treated, or vaccinated—signals the absence of basic monitoring and evaluation infrastructure (Colledge-Frisby et al., 2023). This not only limits accountability but also precludes meaningful assessments of fidelity, penetration, and health outcomes, all of which are core elements of effective implementation.

The difference between countries that have adopted services and those that have achieved extended reach is not trivial. For instance, a country may have a pilot tuberculosis program in a single carceral facility, but without system-wide policy mandates, funding, and integration into national health reporting systems, such initiatives cannot meaningfully curb transmission. These findings underscore the urgent need to shift from fragmented, pilot-level programming to institutionalised, system-wide implementation of essential health services in carceral settings. From an implementation science perspective, this requires more than adoption; it demands scale-up, policy reinforcement, workforce training, financing, and inclusion of prison health metrics in national health information systems.

Furthermore, recent reductions in United States funding for global health programmes have intensified concerns about sustaining essential services in low- and middle- income countries (Apeagyei et al., 2025). These reductions, including permanent discontinuation of funding to United States Agency on International Development (Cavalcanti et al., 2025), and short term freezes that affected multilateral channels and Global Fund supported activities, risk widening gaps in availability of HIV, tuberculosis, and harm reduction services in prisons and jeopardise progress towards 2030 elimination targets (Clark et al., 2025; Zumla et al., 2025).In sum, the global neglect of health service implementation in carceral settings constitutes both a public health failure and a human rights crisis. Epidemiologically, the high burden of communicable diseases in prisons (Degenhardt, 2025) makes these settings critical nodes for global disease control efforts. For implementation, however, the failure to move from adoption to extended reach reveals systemic underinvestment in carceral health. To meet 2030 targets for the elimination of HIV, viral hepatitis, and tuberculosis (United Nations, 2025), national governments and global health stakeholders must prioritise full adoption, system-wide implementation, and routine monitoring of these essential services within prison health systems.

## Limitations

This review documented clear limitations in available data. Despite extensive searches of grey literature, direct engagement with EUDA, UNAIDS, UNODC, WHO, HRI and WHO Regional Office for Europe staff, and consultation with international experts globally, data on the extent of service provision are sorely lacking. National reports rarely detail the scale or reach of interventions, and existing data often document service availability without information on actual implementation or estimates of the number of people reached by each service. This likely explains observations such as more countries having documented tuberculosis screening or diagnosis than documented treatment, which is likely provided in several of these settings but not reported or publicly accessible. Furthermore, services operating outside formal reporting structures and differences in data frameworks between community and carceral settings complicate direct comparisons.

This complexity is further compounded by the varied nature of carceral settings, including short-term detention facilities, where health services may be provided externally through transfer to hospital or municipal health departments, other community-based arrangements, making a precise and universally consistent definition of “service availability within carceral settings” challenging.

Healthcare delivery models differ markedly across the carceral settings included in our review prisons, jails and remand centres. Services may be delivered on-site by correctional health providers, or off-site through transfer or escorted attendance at public hospitals or municipal clinics, including via contracted providers. This heterogeneity makes a universal definition of ‘service availability within carceral settings’ challenging. A lack of on-site documentation may reflect externally delivered care. We therefore interpret availability as minimum documented provision, which also helps explain instances where screening is documented without explicit documentation of treatment.

Regional discrepancies in data availability further constrained the ability to generate coverage estimates. In some regions, detailed national surveillance systems provided comprehensive data, whereas in others, particularly low- and middle-income countries, available data were fragmented or absent. Additionally, despite the standardised global indicators for harm reduction and infectious disease interventions in prisons (WHO UNODC UNAIDS, 2009) there is considerable heterogeneity in reporting of services, which limits comparability across settings and prevents direct benchmarking against community services. Future efforts would benefit from the development of standardised monitoring frameworks to improve data consistency, extraction and synthesis and enhance the ability to track intervention scale-up over time.

Despite these challenges, this review collated the best available evidence, with some limitations. For example, we did not contact every ministry of health or correctional authority worldwide, which would have likely resulted in additional information on availability and coverage. Accordingly, our estimates should be interpreted as minimum documented availability rather than confirmed absence. We welcome feedback at global.reviews@unsw.edu.au, including collaboration enquiries and data contributions to strengthen future assessments.

## Conclusions

Regional priorities differ based on documented availability, noting that unknown status does not imply non-provision. In Western Europe, OAT is widely implemented; additional data on the availability of prison NSPs and on HBV testing, vaccination and treatment would clarify remaining gaps. In Eastern Europe and Central Asia, clearer country-level evidence on OAT availability across facilities and on the presence of prison NSPs would guide policy. In Latin America and in East and Southeast Asia, further facility-level information on the availability of tuberculosis screening and treatment may help interpret regional differences. In Sub-Saharan Africa and the Middle East and North Africa, improved documentation of HIV testing and ART availability is needed. In Pacific Island states and territories, baseline documentation across all core services remains limited and further evidence on availability and programme reach is required.

Our evidence shows clearly the urgent need for a concerted global effort to enhance harm reduction and infectious disease services in carceral settings. Despite the high prevalence of injecting drug use and infectious diseases among incarcerated populations, the availability of essential interventions remains alarmingly low. The evidence presented highlights significant regional disparities, with many countries failing to meet even the most basic standards of care. The review also illustrates the critical interplay between prison health and public health, emphasising that addressing the health needs of incarcerated individuals is essential for reducing overall community transmission rates and improve public health reducing health inequalities.

Our findings highlight the urgent and large-scale need for policy reform, sustained investment, and international accountability to provide adequate health services for incarcerated populations (Joint United Nations Programme on HIV/AIDS, 2021; United Nations, 2025; World Health Organization, 2022b). Governments and international organisations must prioritise: 1) implementing minimum packages of infectious disease services in prisons, including routine screening and treatment for HIV, hepatitis B and C, and tuberculosis screening and diagnosis using validated approaches; 2) expanding availability of OAT in prisons, particularly in regions with high rates of injecting drug use and HIV/HCV prevalence; 3) introduction of needle and syringe programs (NSPs) where injecting drug use is documented in prisons, following WHO recommendations; 4) strengthening health system accountability for incarcerated populations by integrating prison health data and programming into national health systems; and 5) ensuring continuity of care for individuals with HIV, viral hepatitis or tuberculosis at the time of arrest, incarceration, and release, including linkage to community services and treatment programs. Without urgent efforts to expand testing and treatment access in carceral settings through policy and funding commitments the 2030 targets for ending AIDS, viral hepatitis and tuberculosis will remain out of reach (Stone et al., 2023; World Health Organization, 2023b, 2025).

## Supplementary Material

mmc1

Supplementary material associated with this article can be found, in the online version, at doi:10.1016/j.drugpo.2025.105069.

## Figures and Tables

**Fig. 2a.**
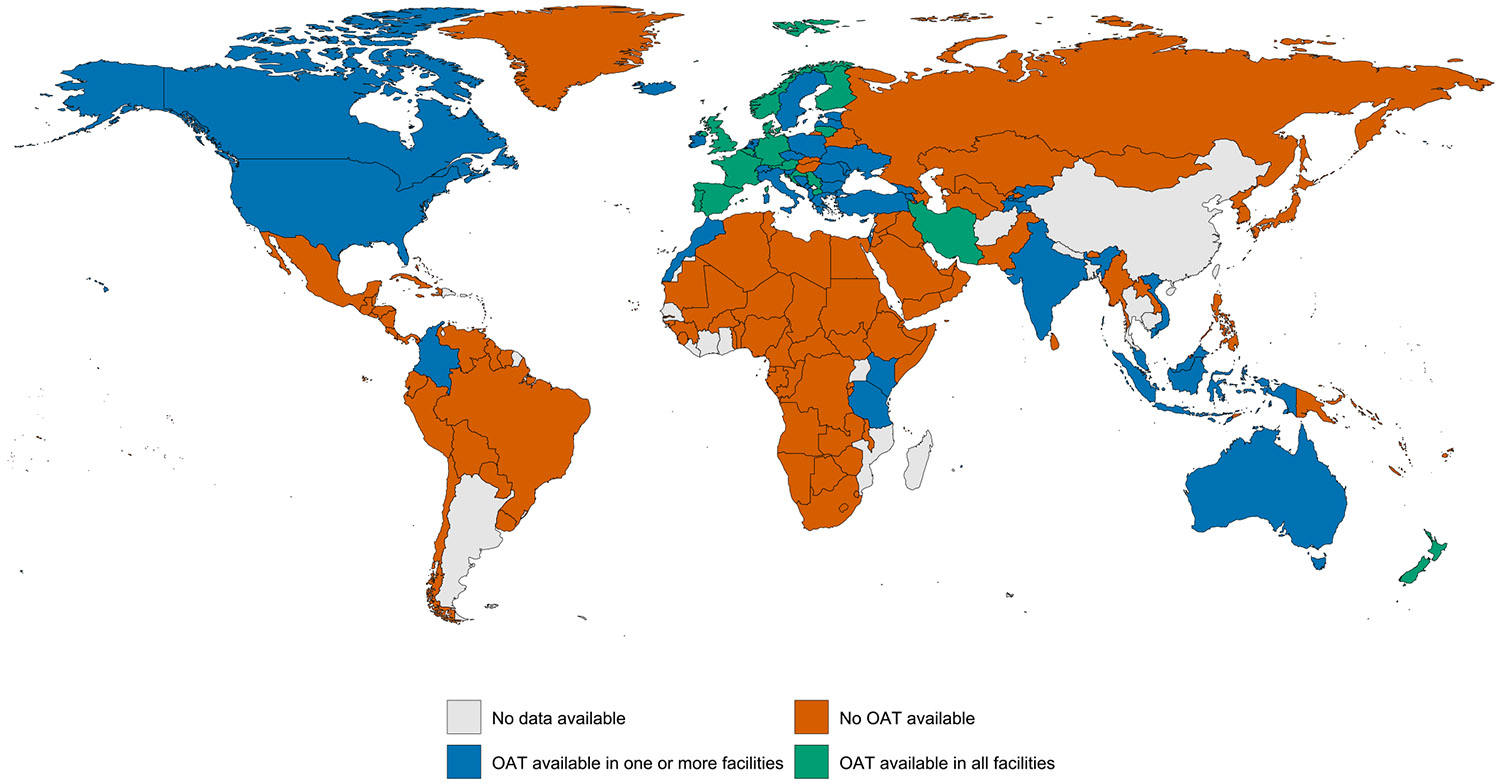
Global availability of OAT in carceral settings. Notes: Carceral Settings: Including prisons, jails or other carceral setting; OAT: Opioid Agonist Treatment.

**Fig. 2b.**
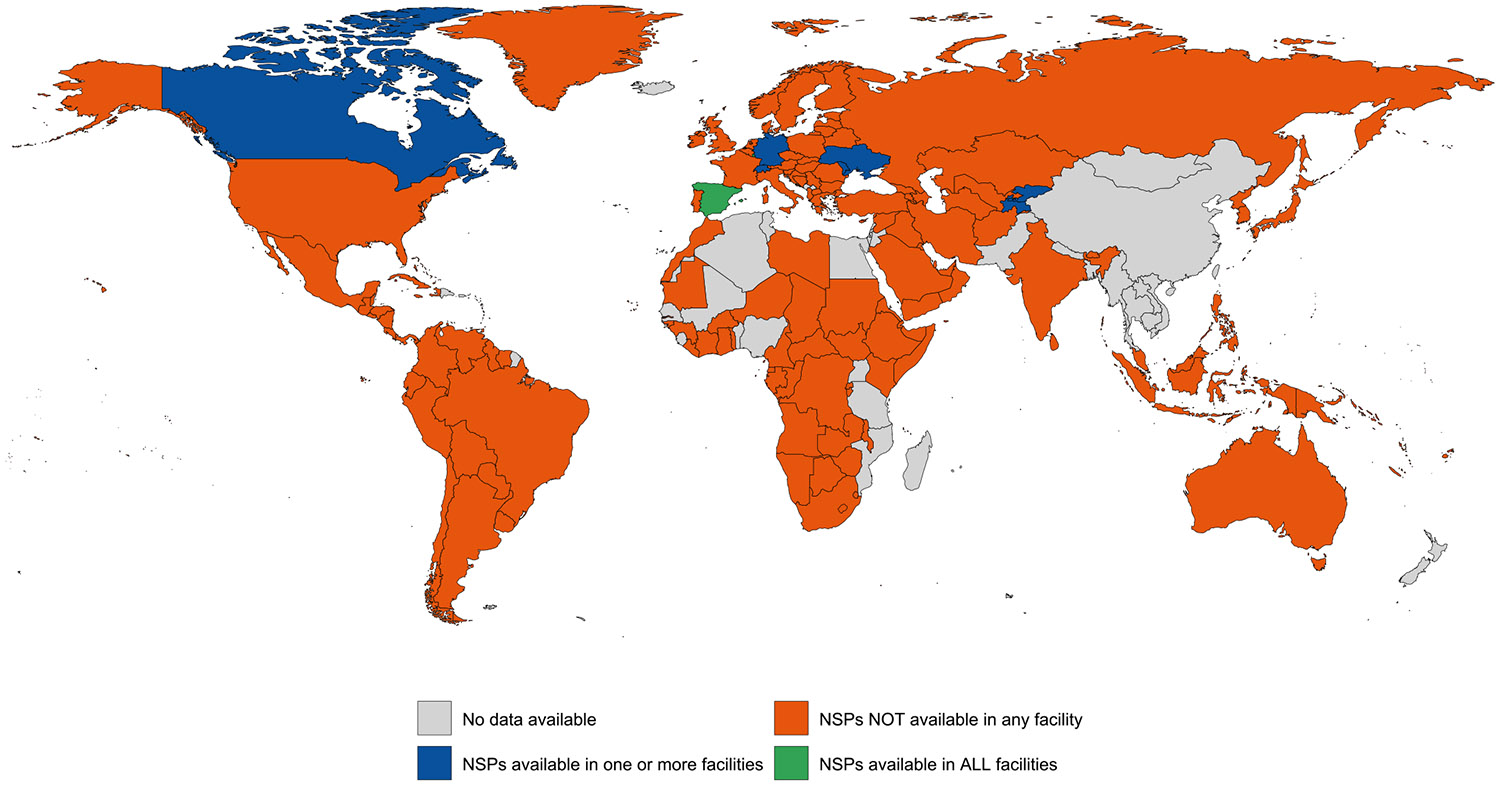
Global availability of NSPs in carceral settings. Notes: There have been reports (World Health Organization, 2023a) of some NSP distribution in Montpellier, but these do not seem to have delivered or sanctioned by the French government. Carceral Settings: Including prisons, jails or other carceral setting; NSP: Needle & Syringe Programme(s).

**Fig. 2c.**
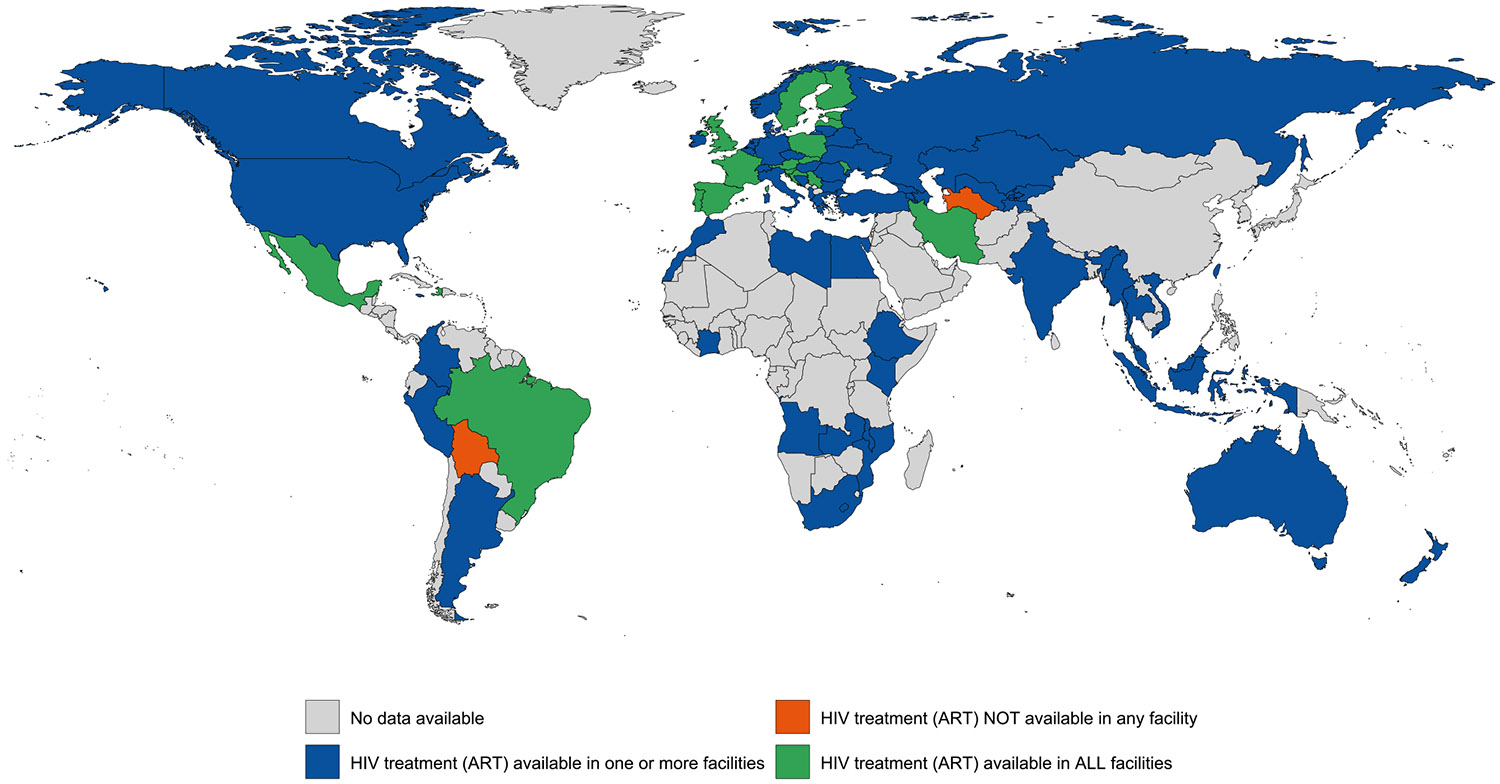
Global availability of HIV treatment (ART) in carceral settings. Notes: Carceral Settings: Including prisons, jails or other carceral setting; HIV: Human Immunodeficiency Virus; ART: Antiretroviral Therapy.

**Fig. 2d.**
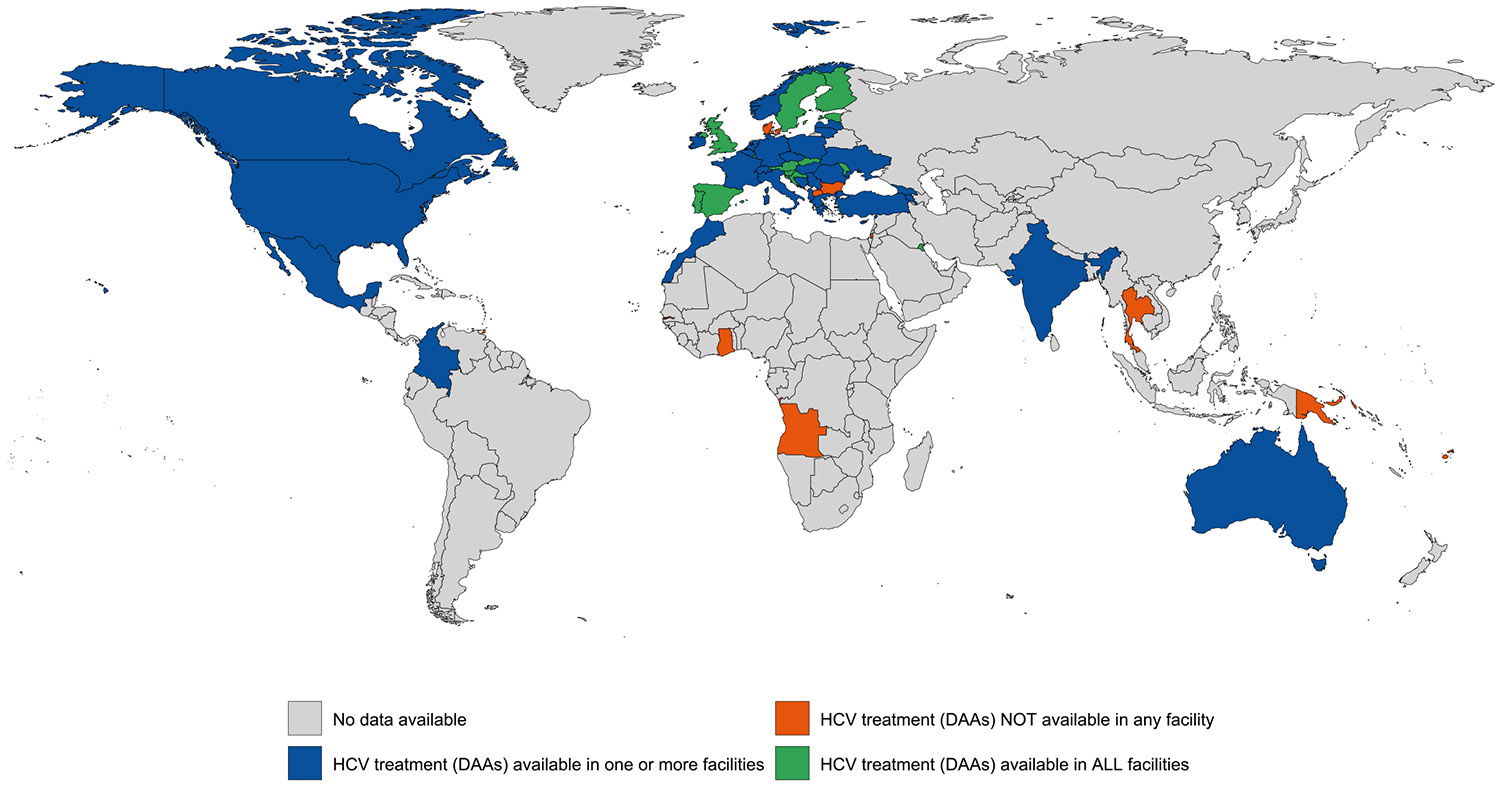
Global availability of HCV treatment (DAAs) in carceral settings. Notes: Carceral Settings: Including prisons, jails or other carceral setting; HCV: Hepatitis C; DAA: Direct-Acting Antiviral.

**Fig. 2e.**
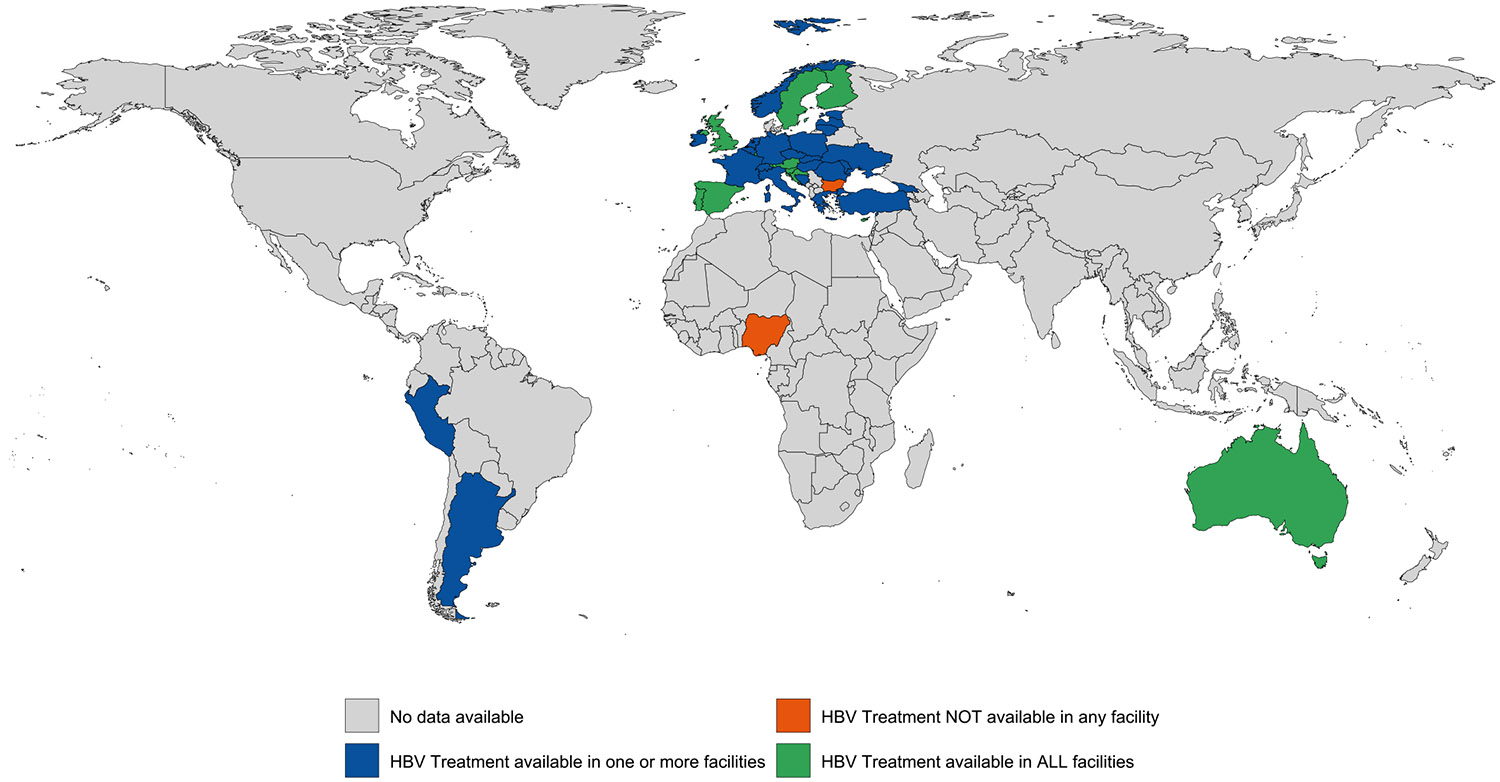
Global availability of HBV treatment in carceral settings.Notes: Carceral Settings: Including prisons, jails or other carceral setting; HBV: Hepatitis B.

**Fig. 2f.**
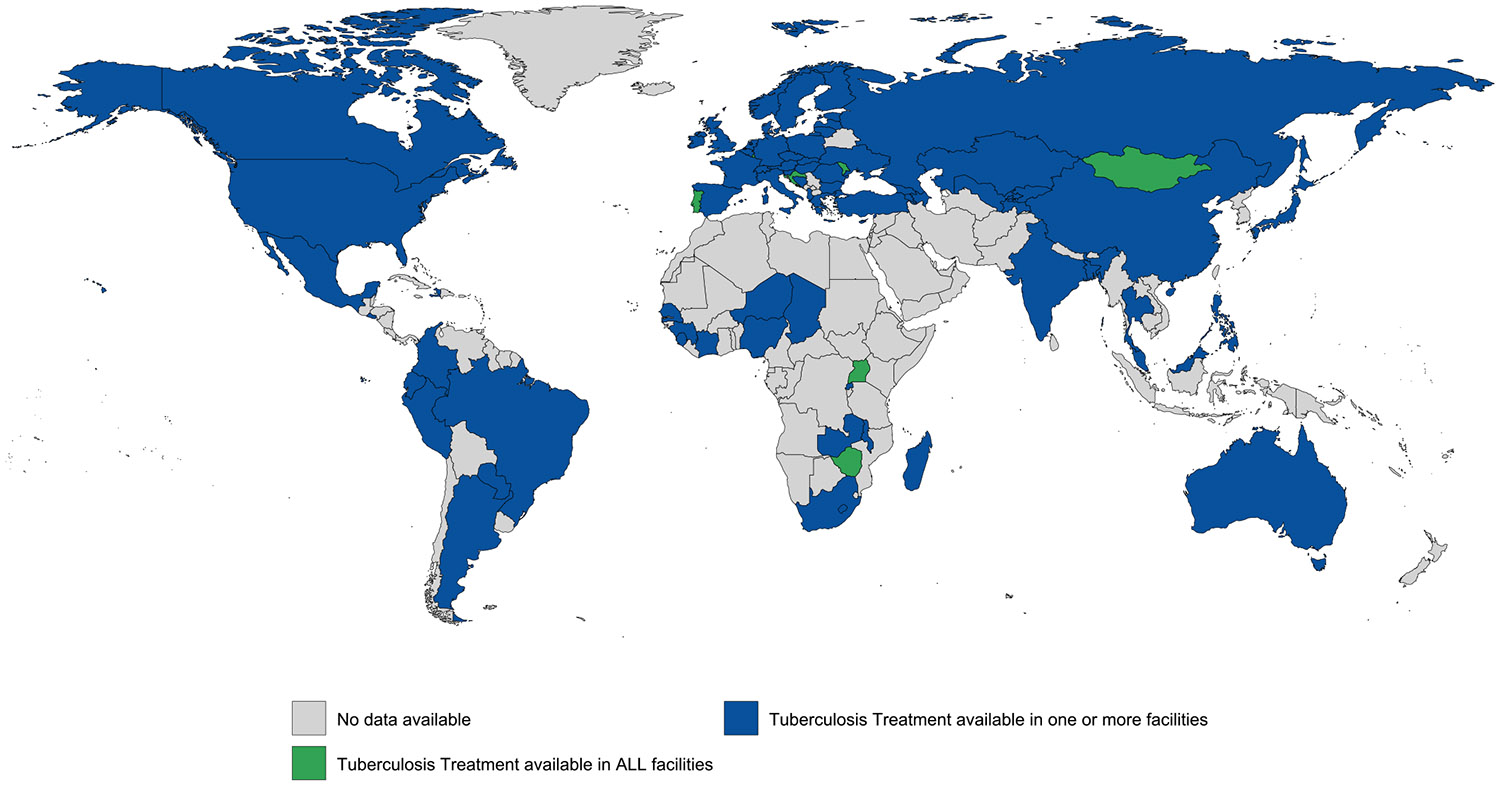
Global availability of tuberculosis treatment in carceral settings. Notes: Carceral Settings: Including prisons, jails or other carceral setting.

**Table 1:** Regional Availability of Harm Reduction and Treatment Services for Infectious Diseases and Injecting Drug Use in Carceral Settings.

		Service Type																							
		Opioid Agonist Treatment												Needle Syringe Programs (NSPs)											
		
Regional Information		≤1 setting, n (% of countries)			ALL carceral settings, n (% countries)			NO carceral settings, n (% countries)			UNKNOWN availability, n (% countries)			≤1 setting, n (% of countries)			ALL carceral settings, n (% countries)			NO carceral settings, n (% countries)			UNKNOWN availability, n (% countries)		
								
Region	Total Countries	n	%	% carceral population	n	%	% carceral population	n	%	% carceral population	n	%	% carceral population	n	%	% carceral population	n	%	% carceral population	n	%	% carceral population	n	%	% carceral population
Eastern Europe	17	12	70.6	27.4	1	5.9	0.6	5	29.4	72.6	0	0.0	0.0	2	11.8	7.0	0	0.0	0.0	15	88.2	93.0	0	0.0	0.0
Western Europe	33	28	84.8	99.9	16	48.5	75.4	3	9.1	0.0	2	6.1	0.0	5	15.2	26.1	3	9.1	12.3	25	75.8	72.7	3	9.1	1.2
East & South-East Asia	18	3	16.7	16.2	0	0.0	0.0	10	55.6	16.1	5	27.8	67.7	0	0.0	0.0	0	0.0	0.0	10	55.6	24.3	8	44.4	75.7
South Asia	9	2	22.2	75.9	1	11.1	18.8	3	33.3	13.9	4	44.4	10.2	0	0.0	0.0	0	0.0	0.0	6	66.7	81.1	3	33.3	18.9
Central Asia	5	2	40.0	18.0	0	0.0	0.0	3	60.0	82.0	0	0.0	0.0	2	40.0	18.0	0	0.0	0.0	3	60.0	82.0	0	0.0	0.0
Caribbean	15	0	0.0	0.0	0	0.0	0.0	13	86.7	77.5	2	13.3	22.5	0	0.0	0.0	0	0.0	0.0	13	86.7	77.5	2	13.3	22.5
Latin America	20	1	5.0	5.5	0	0.0	0.0	18	90.0	87.9	1	5.0	6.6	0	0.0	0.0	0	0.0	0.0	20	100.0	100.0	0	0.0	0.0
North America*^†^	2	2	100.0	100.0	1	50.0	1.9	0	0.0	0.0	0	0.0	0.0	1	50.0	1.9	0	0.0	0.0	1	50.0	98.1	0	0.0	0.0
Pacific Islands**	17	0	0.0	0.0	0	0.0	0.0	17	100.0	100.0	0	0.0	0.0	0	0.0	0.0	0	0.0	0.0	17	100.0	100.0	0	0.0	0.0
Australasia	2	2	100.0	100.0	1	50.0	18.4	0	0.0	0.0	0	0.0	0.0	0	0.0	0.0	0	0.0	0.0	1	50.0	81.6	1	50.0	18.4
Sub-Saharan Africa	47	3	6.4	9.4	0	0.0	0.0	36	76.6	72.1	8	17.0	18.5	0	0.0	0.0	0	0.0	0.0	36	76.6	70.9	11	23.4	29.1
Middle East & North Africa	22	4	18.2	50.0	0	0.0	0.0	16	72.7	49.0	2	9.1	0.9	0	0.0	0.0	0	0.0	0.0	15	68.2	71.1	7	31.8	28.9
**Global**	**207**	59	28.5	40.0	20	9.7	5.2	124	59.9	37.6	24	11.6	22.4	10	4.8	2.0	3	1.4	0.5	162	78.3	70.3	35	16.9	27.7
		HIV Testing												HIV Treatment (ART)											
Regional Information		≥1 setting, n (% of countries)			ALL carceral settings, n (% countries)			NO carceral settings, n (% countries)			UNKNOWN availability, n (% countries)			≥1 setting, n (% of countries)			ALL carceral settings, n (% countries)			NO carceral settings, n (% countries)			UNKNOWN availability, n (% countries)		
								
Region	Total Countries	n	%	% carceral population	n	%	% carceral population	n	%	% carceral population	n	%	% carceral population	n	%	% carceral population	n	%	% carceral population	n	%	% carceral population	n	%	% carceral population
Eastern Europe	17	16	94.1	95.4	10	58.8	24.2	0	0.0	0.0	1	5.9	4.6	17	100.0	100.0	5	29.4	12.6	0	0.0	0.0	0	0.0	0.0
Western Europe	33	27	81.8	99.2	20	60.6	80.2	0	0.0	0.0	6	18.2	0.8	24	72.7	98.7	12	36.4	58.7	0	0.0	0.0	9	27.3	1.3
East & South-East Asia	18	7	38.9	30.1	1	5.6	2.9	0	0.0	0.0	11	61.1	69.9	6	33.3	30.3	0	0.0	0.0	0	0.0	0.0	12	66.7	69.7
South Asia	9	5	55.6	81.8	0	0.0	0.0	1	11.1	10.8	3	33.3	7.4	2	22.2	75.9	1	11.1	18.8	0	0.0	0.0	7	77.8	24.1
Central Asia	5	5	100.0	100.0	0	0.0	0.0	0	0.0	0.0	0	0.0	0.0	4	80.0	71.1	0	0.0	0.0	1	20.0	28.9	0	0.0	0.0
Caribbean	15	0	0.0	0.0	0	0.0	0.0	0	0.0	0.0	15	100.0	100.0	2	13.3	7.8	1	6.7	5.3	0	0.0	0.0	13	86.7	92.2
Latin America	20	8	40.0	76.3	1	5.0	46.9	0	0.0	0.0	12	60.0	23.7	5	25.0	76.6	2	10.0	59.3	1	5.0	1.6	14	70.0	21.8
North America*,^†^	2	2	100.0	100.0	1	50.0	98.1	0	0.0	0.0	0	0.0	0.0	2	100.0	100.0	1	50.0	98.1	0	0.0	0.0	0	0.0	0.0
Pacific Islands	17	0	0.0	0.0	0	0.0	0.0	0	0.0	0.0	17	100.0	100.0	0	0.0	0.0	0	0.0	0.0	0	0.0	0.0	17	100.0	100.0
Australasia	2	1	50.0	81.6	0	0.0	0.0	0	0.0	0.0	1	50.0	18.4	2	100.0	100.0	0	0.0	0.0	0	0.0	0.0	0	0.0	0.0
Sub-Saharan Africa	47	7	14.9	33.3	2	4.3	10.5	0	0.0	0.0	40	85.1	66.7	10	21.3	44.1	0	0.0	0.0	0	0.0	0.0	37	78.7	55.9
Middle East & North Africa	22	8	36.4	72.5	3	13.6	12.5	0	0.0	0.0	14	63.6	27.5	5	22.7	62.1	0	0.0	0.0	0	0.0	0.0	17	77.3	37.9
**Global**	**207**	86	41.5	65.3	38	18.4	31.4	1	0.5	1.0	120	58.0	33.7	79	38.2	65.1	22	10.6	30.8	2	1.0	0.6	126	60.9	34.3
		Service Type																							
		HCV Testing												HCV Treatment (DAAs)											
		
Regional Information		≥1 setting, n (% of countries)			ALL carceral settings, n (% countries)			NO carceral settings, n (% countries)			UNKNOWN availability, n (% countries)			≥1 setting, n (% of countries)			ALL carceral settings, n (% countries)			NO carceral settings, n (% countries)			UNKNOWN availability, n (% countries)		
								
Region	Total Countries	n	%	% carceral population	n	%	% carceral population	n	%	% carceral population	n	%	% carceral population	n	%	% carceral population	n	%	% carceral population	n	%	% carceral population	n	%	% carceral population
Eastern Europe	17	14	82.4	31.2	9	52.9	23.3	0	0.0	0.0	3	17.6	68.8	13	76.5	30.3	3	17.6	2.2	1	5.9	0.9	3	17.6	68.8
Western Europe	33	27	81.8	99.2	18	54.5	77.9	0	0.0	0.0	6	18.2	0.8	24	72.7	98.3	12	36.4	41.6	2	6.1	1.4	7	21.2	0.3
East & South-East Asia	18	1	5.6	1.9	0	0.0	0.0	0	0.0	0.0	17	94.4	98.1	0	0.0	0.0	0	0.0	0.0	2	11.1	17.9	16	88.9	82.1
South Asia	9	2	22.2	57.3	0	0.0	0.0	0	0.0	0.0	7	77.8	42.7	1	11.1	57.1	0	0.0	0.0	0	0.0	0.0	8	88.9	42.9
Central Asia	5	0	0.0	0.0	0	0.0	0.0	0	0.0	0.0	5	100.0	100.0	0	0.0	0.0	0	0.0	0.0	0	0.0	0.0	5	100.0	100.0
Caribbean	15	0	0.0	0.0	0	0.0	0.0	0	0.0	0.0	15	100.0	100.0	0	0.0	0.0	0	0.0	0.0	2	13.3	3..0	13	86.7	97.0
Latin America	20	3	15	64.8	1	5.0	46.9	0	0.0	0.0	17	85.0	35.2	2	10.0	17.9	0	0.0	0.0	0	0.0	0.0	18	90.0	82.1
North America*^†^	2	2	100.0	100.0	0	0.0	0.0	0	0.0	0.0	0	0.0	0.0	2	100.0	100.0	0	0.0	0.0	0	0.0	0.0	0	0.0	0.0
Pacific Islands**	17	0	0.0	0.0	0	0.0	0.0	0	0.0	0.0	17	100.0	100.0	0	0.0	0.0	0	0.0	0.0	2	11.8	62.6	15	88.2	37.4
Australasia	2	1	50.0	81.6	0	0.0	0.0	0	0.0	0.0	1	50.0	18.4	1	50.0	81.6	0	0.0	0.0	0	0.0	0.0	1	50.0	18.4
Sub-Saharan Africa	47	1	2.1	3.4	0	0.0	0.0	0	0.0	0.0	46	97.9	96.6	0	0.0	0.0	0	0.0	0.0	3	6.4	3.8	44	93.6	96.2
Middle East & North Africa	22	4	18.2	50.3	2	9.1	0.6	0	0.0	0.0	18	81.8	49.7	4	18.2	48.6	1	4.5	0.5	1	4.5		17	77.3	51.4
**Global**	**207**	55	26.6	43.9	30	14.5	12.6	0	0.0	0.0	152	73.4	56.2	47	22.7	34.9	16	7.7	1.9	13	6.3	3.0	147	71.0	62.1
		HBV Testing												HBV Treatment											
Regional Information		≥1 setting, n (% of countries)			ALL carceral settings, n (% countries)			NO carceral settings, n (% countries)			UNKNOWN availability, n (% countries)			≥1 setting, n (% of countries)			ALL carceral settings, n (% countries)			NO carceral settings, n (% countries)			UNKNOWN availability, n (% countries)		
								
Region	Total Countries	n	%	% carceral population	n	%	% carceral population	n	%	% carceral population	n	%	% carceral population	n	%	% carceral population	n	%	% carceral population	n	%	% carceral population	n	%	% carceral population
Eastern Europe	17	14	82.4	31.2	8	47.1	22.9	0	0.0	0.0	3	17.6	68.8	12	70.6	29.9	0	0.0	0.0	1	5.9	0.9	4	23.5	69.2
Western Europe^‡^	33	25	75.8	96.8	17	51.5	76.9	0	0.0	0.0	8	24.2	3.2	19	57.6	92.8	9	27.3	39.4	0	0.0	0.0	14	42.4	7.2
East & South-East Asia	18	0	0.0	0.0	0	0.0	0.0	0	0.0	0.0	18	100.0	100.0	0	0.0	0.0	0	0.0	0.0	0	0.0	0.0	18	100.0	100.0
South Asia	9	1	11.1	0.2	0	0.0	0.0	1	11.1	10.8	7	77.8	89.0	0	0.0	0.0	0	0.0	0.0	0	0.0	0.0	9	100.0	100.0
Central Asia	5	0	0.0	0.0	0	0.0	0.0	0	0.0	0.0	5	100.0	100.0	0	0.0	0.0	0	0.0	0.0	0	0.0	0.0	5	100.0	100.0
Caribbean	15	0	0.0	0.0	0	0.0	0.0	0	0.0	0.0	15	100.0	100.0	0	0.0	0.0	0	0.0	0.0	0	0.0	0.0	15	100.0	100.0
Latin America	20	3	15	65.9	0	0.0	0.0	0	0.0	0.0	17	85.0	34.1	2	10.0	11.8	0	0.0	0.0	0	0.0	0.0	18	90.0	88.2
North America*^†^	2	2	100.0	100.0	0	0.0	0.0	0	0.0	0.0	0	0.0	0.0	0	0.0	0.0	0	0.0	0.0	0	0.0	0.0	2	100.0	100.0
Pacific Islands**	17	0	0.0	0.0	0	0.0	0.0	0	0.0	0.0	17	100.0	100.0	0	0.0	0.0	0	0.0	0.0	0	0.0	0.0	17	100.0	100.0
Australasia	2	1	50.0	81.6	0	0.0	0.0	0	0.0	0.0	1	50.0	18.4	1	50.0	81.6	1	50.0	81.6	0	0.0	0.0	1	50.0	18.4
Sub-Saharan Africa	47	1	2.1	3.4	0	0.0	0.0	1	2.1	8.2	45	95.7	88.4	0	0.0	0.0	0	0.0	0.0	1	2.1	8.2	46	97.9	91.8
Middle East & North Africa	22	4	18.2	50.7	1	4.5	0.1	0	0.0	0.0	18	81.8	49.3	2	9.1	37.7	1	4.5	0.1	0	0.0	0.0	20	90.9	62.3
**Global**	**207**	51	24.6	38.4	26	12.6	4.6	2	1.0	1.7	154	74.4	59.9	36	17.4	11.4	11	5.3	2.0	2	1.0	0.8	169	81.6	87.8
		Service Type																							
		Tuberculosis Screening												Tuberculosis Treatment											
		
Regional Information		≥1 setting, n (% of countries)			ALL carceral settings, n (% countries)			NO carceral settings, n (% countries)			UNKNOWN availability, n (% countries)			≥1 setting, n (% of countries)			ALL carceral settings, n (% countries)			NO carceral settings, n (% countries)			UNKNOWN availability, n (% countries)		
								
Region	Total Countries	n	%	% carceral population	n	%	% carceral population	n	%	% carceral population	n	%	% carceral population	n	%	% carceral population	n	%	% carceral population	n	%	% carceral population	n	%	% carceral population
Eastern Europe	17	16	94.1	95.4	14	82.4	31.2	0	0.0	0.0	1	5.9	4.6	16	94.1	95.4	1	5.9	0.8	0	0.0	0.0	1	5.9	4.6
Western Europe	33	24	72.7	94.0	23	69.7	93.9	0	0.0	0.0	9	27.3	6.0	24	72.7	96.8	3	9.1	3.7	0	0.0	0.0	9	27.3	3.2
East & South-East Asia	18	14	77.8	95.1	2	11.1	3.0	0	0.0	0.0	4	22.2	4.9	7	38.9	74.3	1	5.6	0.2	0	0.0	0.0	11	61.1	25.7
South Asia	9	5	55.6	95.1	0	0.0	0.0	1	11.1	0.2	3	33.3	4.7	2	22.2	62.5	0	0.0	0.0	0	0.0	0.0	7	77.8	37.5
Central Asia	5	1	20.0	6.4	0	0.0	0.0	0	0.0	0.0	4	80.0	93.6	4	80.0	71.1	0	0.0	0.0	0	0.0	0.0	1	20.0	28.9
Caribbean	15	1	6.7	18.4	0	0.0	0.0	0	0.0	0.0	14	93.3	81.6	1	6.7	5.3	0	0.0	0.0	0	0.0	0.0	14	93.3	94.7
Latin America	20	9	45.0	86.3	0	0.0	0.0	0	0.0	0.0	11	55.0	13.7	8	40.0	85.1	0	0.0	0.0	0	0.0	0.0	12	60.0	14.9
North America*^†^	2	2	100.0	100.0	0	0.0	0.0	0	0.0	0.0	0	0.0	0.0	2	100.0	100.0	0	0.0	0.0	0	0.0	0.0	0	0.0	0.0
Pacific Islands**	17	0	0.0	0.0	0	0.0	0.0	0	0.0	0.0	17	100.0	100.0	0	0.0	0.0	0	0.0	0.0	0	0.0	0.0	17	100.0	100.0
Australasia	2	1	50.0	81.6	0	0.0	0.0	0	0.0	0.0	1	50.0	18.4	1	50.0	81.6	0	0.0	0.0	0	0.0	0.0	1	50.0	18.4
Sub-Saharan Africa	47	20	42.6	75.4	2	4.3	23.1	0	0.0	0.0	27	57.4	24.6	14	29.8	56.5	2	4.3	9.8	0	0.0	0.0	33	70.2	43.5
Middle East & North Africa	22	3	13.6	49.8	1	4.5	0.1	0	0.0	0.0	19	86.4	50.2	2	9.1	37.7	0	0.0	0.0	0	0.0	0.0	20	90.9	62.3
**Global**	**207**	96	46.4	86.6	42	20.3	8.7	1	0.5	0.0	110	53.1	13.4	81	39.1	75.8	7	3.4	1.1	0	0.0	0.0	126	60.9	24.2

**Notes:** Country level data that informed these regional and global estimates were sourced from the World Prison Brief, collated by the Institute for Crime and Justice Policy Research at Burbeck University. **See:** https://www.prisonstudies.org/world-prison-brief-data; Country Level estimates for these interventions and their corresponding sources can be seen in Appendix 6**. Carceral Settings:** Including prisons, jails or other carceral setting; **OAT:** Opioid Agonist Treatment; **NSP**: Needle & Syringe Programme(s); **HIV**: Human Immunodeficiency Virus; **ART**: Antiretroviral Therapy; **HCV**: Hepatitis C; **DAA**: Direct-Acting Antiviral; **HBV**: Hepatitis B.

·· Indicates there were no data to inform a region’s estimate. For example, we did not have an incarcerated population estimate for Occupied Palestinian territories and therefore could not calculate the % of incarcerated population covered.

* For the United States, we excluded 663 100 people who are incarcerated in local jails from the total population calculations included in regional availability tables as we had insufficient data on nationwide coverage of interventions from this carceral category. See: https://www.prisonstudies.org/country/united-states-america. See Appendix 5.2 for further details.

† For Canada and the United States, availability was assessed separately for federal and for provincial/territorial (Canada) or state (United States) prison systems (Appendix 6). “Number of countries” counts include a country once in “one or more facilities” if at least one system provides the service. “% of incarcerated population” figures include only the proportion in systems with the service; populations in systems without it are counted as “not available. For example, for North America the NSP availability in “One or More Carceral Setting is reported as 1 country (50%), accounting for 1% of the incarcerated population. We found evidence of NSP availability only in Canadian Federal facilities, which is therefore reflected in the percentage of incarcerated population covered.

‡ For England and Wales, the World Prison Brief only reports a combined incarcerated population, therefore we reported a combined availability for HBV treatment as available in “One or More Carceral Setting” to enable calculation of % of incarcerated population. See Appendix 5.2 for further details.

** Full GBD region name: Pacific Islands States & Territories
