# Supplementary material for: Global coverage of interventions for reduction of injecting drug use-related harm, HIV, viral hepatitis and tuberculosis in prisons and other carceral settings: A systematic review
Source: Int J Drug Policy. Author manuscript; Available in PMC 2026 Apr 6. (PMC13051234; doi:10.1016/j.drugpo.2025.105069)
Supplement: mmc1 [file NIHMS2157185-supplement-mmc1.docx]

**Table of contents**

[Appendix 1: GATHER and PRISMA checklist 4](#_Toc213424860)

[1.1 GATHER checklist 4](#_Toc213424861)

[1.2 PRISMA checklist 5](#_Toc213424862)

[Appendix 2: Peer Reviewed Literature Search 6](#_Toc213424863)

[2.1 Medline Search Strategy 6](#_Toc213424864)

[2.2. Embase Search Strategy 7](#_Toc213424865)

[2.3. PsycINFO Search Strategy 10](#_Toc213424866)

[2.4. Web of Science Search Strategy 11](#_Toc213424867)

[2.5. CINAHL Search Strategy 12](#_Toc213424868)

[Appendix 3: Grey Literature Search 15](#_Toc213424869)

[Table 3.1: Websites searched in the grey literature search 16](#_Toc213424870)

[Appendix 4: Additional requests for information 29](#_Toc213424871)

[4.1 Example email 29](#_Toc213424872)

[4.2.1 Facebook advertisement example 30](#_Toc213424873)

[4.2.2 Twitter (X) advertisement example 31](#_Toc213424874)

[Appendix 5.1: Screening Process and Criteria 32](#_Toc213424875)

[Appendix 5.2: Summary of decision rules for data extraction and estimation processes 33](#_Toc213424876)

[Appendix 6: Country level availability of interventions and programmatic data for NSPs, OAT, HIV, HCV, HBV and tuberculosis in Carceral Settings 37](#_Toc213424877)

[*Appendix Table 6.1*: Country-level availability of interventions and programmatic data for any Opioid Agonist Treatment (OAT) 37](#_Toc213424878)

[*Appendix Table 6.2*: Country-level availability of interventions and programmatic data for Needle Syringe Programs 49](#_Toc213424879)

[*Appendix Table 6.3*: Country-level availability of interventions and programmatic data for HIV Testing 60](#_Toc213424880)

[*Appendix Table 6.4*: Country-level availability of interventions and programmatic data for HIV Treatment (ART) 73](#_Toc213424881)

[*Appendix Table 6.5*: Country-level availability of interventions and programmatic data for HCV Testing 86](#_Toc213424882)

[*Appendix Table 6.6*: Country-level availability of interventions and programmatic data for HCV Treatment (DAAs) 98](#_Toc213424883)

[*Appendix Table 6.7*: Country-level availability of interventions and programmatic data for HBV Testing 109](#_Toc213424884)

[*Appendix Table 6.8*: Country-level availability of interventions and programmatic data for HBV Treatment 121](#_Toc213424885)

[*Appendix 6.9*: Regional and Country-level availability of interventions and programmatic data for HBV Vaccination 131](#_Toc213424886)

[*Appendix Table 6.9.1*: Regional availability of interventions for HBV Vaccination 131](#_Toc213424887)

[*Appendix Table 6.9.2*: Country-level availability of interventions and programmatic data for HBV Vaccination 132](#_Toc213424888)

[*Appendix Table 6.10*: Country-level availability of interventions and programmatic data for Tuberculosis Screening 142](#_Toc213424889)

[*Appendix Table 6.11*: Country-level availability of interventions and programmatic data for tuberculosis treatment 155](#_Toc213424890)

[Appendix 7: Additional maps of service availability 168](#_Toc213424891)

[*Figure 7a*: Global availability of HIV testing in carceral settings 168](#_Toc213424892)

[*Figure 7b*: Global availability of HCV testing in carceral settings 168](#_Toc213424893)

[*Figure 7c*: Global availability of HBV testing in carceral settings 169](#_Toc213424894)

[*Figure 7d*: Global availability of HBV Vaccination availability in carceral settings 169](#_Toc213424895)

[*Figure 7e*: Global availability of Tuberculosis Screening in carceral settings 170](#_Toc213424896)

[Appendix 8: Country-level availability of all outcomes 172](#_Toc213424897)

[Appendix 9: People who provided data or advice regarding the reviews 180](#_Toc213424898)

[Appendix 10: Study quality tables for included studies for each indicator: NSPs, OAT, HIV, HCV, HBV and tuberculosis 181](#_Toc213424899)

[*Appendix Table 10.1: Source characteristics for any Opioid Agonist Treatment (OAT) coverage in carceral settings by country* 181](#_Toc213424900)

[*Appendix Table 10.2*: Source characteristics for Needle Syringe Programs (NSPs) coverage in carceral settings by country 185](#_Toc213424901)

[*Appendix Table 10.3* Source characteristics for HIV Testing coverage in carceral settings by country 189](#_Toc213424902)

[*Appendix Table 10.4*: Source characteristics for HIV Treatment coverage in carceral settings by country 193](#_Toc213424903)

[*Appendix Table 10.5*: Source characteristics for HCV Testing coverage in carceral settings by country 197](#_Toc213424904)

[*Appendix Table 10.6*: Source characteristics for HCV Treatment (DAAs) coverage in carceral settings by country 201](#_Toc213424905)

[*Appendix Table 10.7*: Source characteristics for HBV Testing coverage in carceral settings by country 205](#_Toc213424906)

[*Appendix Table 10.8*: Source characteristics for HBV Treatment coverage in carceral settings by country 209](#_Toc213424907)

[*Appendix Table 10.9*: Source characteristics for HBV vaccination coverage in carceral settings by country 213](#_Toc213424908)

[*Appendix Table 10.10: Source characteristics for Tuberculosis Screening coverage in carceral settings by country* 217](#_Toc213424909)

[*Appendix Table 10.11: Source characteristics for Tuberculosis Treatment coverage in carceral settings by country* 221](#_Toc213424910)

# Appendix 1: GATHER and PRISMA checklist

## GATHER checklist

| **#** | **GATHER checklist item** | **Description of compliance** | **Reference** |
| --- | --- | --- | --- |
| **Objectives and funding** | | | |
| 1 | Define the indicators, populations, and time periods for which estimates were made. | Narrative provided in paper and methods appendix describing indicators, definitions, and populations | Manuscript (Methods) and methods appendix |
| 2 | List the funding sources for the work. | Funding sources listed in paper | Summary (Funding) |
| **Data Inputs** | | | |
| *For all data inputs from multiple sources that are synthesized as part of the study:* | | | |
| 3 | Describe how the data were identified and how the data were accessed. | Narrative description of data seeking  methods provided | Manuscript (Methods) and appendix |
| 4 | Specify the inclusion and exclusion criteria. Identify all ad-hoc exclusions. | Narrative about inclusion and exclusion criteria by data type provided | Methods appendix |
| 5 | Provide information on all included data sources and their main characteristics. For each data source used, report reference information or contact name/institution, population represented, data collection method, year(s) of data collection, sex and age range, diagnostic criteria or measurement method, and sample size, as relevant. | Included in appendix and will be accessible online | Appendix and online web page |
| 6 | Identify and describe any categories of input data that have potentially important biases (e.g., based on characteristics listed in item 5). | Discussed in limitations section and in methods appendix | Manuscript and appendix |
| *For data inputs that contribute to the analysis but were not synthesized as part of the study:* | | | |
| 7 | Describe and give sources for any other data inputs. | N/A | N/A |
| *For all data inputs* | | | |

## PRISMA checklist

| **Section/topic** | **#** | **Checklist item** | **Reported on page #** |
| --- | --- | --- | --- |
| **TITLE** | | |  |
| Title | 1 | Identify the report as a systematic review, meta-analysis, or both. | 1 |
| **ABSTRACT** | | |  |
| Structured summary | 2 | Provide a structured summary including, as applicable: background; objectives; data sources; study eligibility criteria, participants, and interventions; study appraisal and synthesis methods; results; limitations; conclusions and implications of key findings; systematic review registration number. | 2 |
| **INTRODUCTION** | | |  |
| Rationale | 3 | Describe the rationale for the review in the context of what is already known. | 3-4 |
| Objectives | 4 | Provide an explicit statement of questions being addressed with reference to participants, interventions, comparisons, outcomes, and study design (PICOS). | 3-4 |
| **METHODS** | | |  |
| Protocol and registration | 5 | Indicate if a review protocol exists, where it can be accessed, and, if available, provide registration information including registration number. | 5 |
| Eligibility criteria | 6 | Specify study characteristics (e.g., PICOS, length of follow-up) and report characteristics used as criteria for eligibility, giving rationale. | 5-6, Appendix 2 |
| Information sources | 7 | Describe all information sources (e.g., databases with dates of coverage, contact with study authors to identify additional studies) in the search and date last searched. | 5-6, Appendix 2 |
| Search | 8 | Present full electronic search strategy for at least one database, including any limits used, such that it could be repeated. | 5-6, Appendix 2 |
| Study selection | 9 | State the process for selecting studies (i.e., screening, eligibility, included in systematic review, and, if applicable, included in the meta-analysis). | 6-7, Appendix 5.1 |
| Data collection process | 10 | Describe method of data extraction (e.g., piloted forms, independently, in duplicate) and any processes for obtaining and confirming data from investigators. | 6-7, Appendix 5.1 |
| Data items | 11 | List and define all variables for which data were sought (e.g., PICOS, funding sources) and any assumptions and simplifications made. | 6-7, Appendix 5.1 |
| Risk of bias in individual studies | 12 | Describe methods used for assessing risk of bias of individual studies (including specification of whether this was done at the study or outcome level), and how this information is to be used in any data synthesis. | 8-9 |
| Summary measures | 13 | State the principal summary measures (e.g., risk ratio, difference in means). | 9-10, Appendix 5.2 |
| Synthesis of results | 14 | Describe the methods of handling data and combining results of studies, if done, including measures of consistency (e.g., I^2^) for each meta-analysis. | 9-10, Appendix 5.2 |
| Risk of bias across studies | 15 | Specify any assessment of risk of bias that may affect the cumulative evidence (e.g., publication bias, selective reporting within studies). | 8-9 |
| Additional analyses | 16 | Describe methods of additional analyses (e.g., sensitivity or subgroup analyses, meta-regression), if done, indicating which were pre-specified. | 9-10 |
| **RESULTS** | | |  |
| Study selection | 17 | Give no. studies screened, assessed for eligibility, and included in the review, with reasons for exclusions at each stage, ideally with a flow diagram. | 11, 33 |
| Study characteristics | 18 | For each study, present characteristics for which data were extracted (e.g., study size, PICOS, follow-up period) and provide the citations. | N/A |
| Risk of bias within studies | 19 | Present data on risk of bias of each study and, if available, any outcome level assessment (see item 12). | 15-16, Appendix 10 |
| Results of individual studies | 20 | For all outcomes considered (benefits or harms), present, for each study: (a) simple summary data for each intervention group (b) effect estimates and confidence intervals, ideally with a forest plot. | 34-36 |
| Synthesis of results | 21 | Present results of each meta-analysis done, including confidence intervals and measures of consistency. | 34-36 |
| Risk of bias across studies | 22 | Present results of any assessment of risk of bias across studies (see Item 15). | 15-16, Appendix 10 |
| Additional analysis | 23 | Give results of additional analyses, if done (e.g., sensitivity or subgroup analyses, meta-regression [see Item 16]). | N/A |
| **DISCUSSION** | | |  |
| Summary of evidence | 24 | Summarize the main findings including the strength of evidence for each main outcome; consider their relevance to key groups | 17 |
| Limitations | 25 | Discuss limitations at study and outcome level (e.g., risk of bias), and at review-level (e.g., incomplete retrieval of identified research, reporting bias). | 22-23 |
| Conclusions | 26 | Provide a general interpretation of the results in the context of other evidence, and implications for future research. | 17-21 |
| **FUNDING** | | |  |
| Funding | 27 | Describe sources of funding for the systematic review and other support (e.g., supply of data); role of funders for the systematic review. | 2, 10 |

# Appendix 2: Peer Reviewed Literature Search

Five peer reviewed literature databases were searched in this exercise: Medline, EMBASE, PsycINFO, Web of Science, and CINAHL.

Listed below are the different literature and search strategies and terminologies used for each of the different databases. Because different databases use different terminologies and require searching in different ways it was necessary to develop search strategies for each database.

These searches were developed in close consultation with a specialist drug and alcohol archivist and with a generalist university librarian with expertise across all the databases searched.

## 2.1 Medline Search Strategy

The search strategy was as follows:

Results were restricted to those articles containing terms from search 1 and terms from one or more results of searches 2-8, combined as 1 AND (2 OR 3 OR 4 OR 5 OR 6 OR 7 OR 8). Results were restricted to articles published 2000-current and limited to population group: humans.

Original searches were conducted in March 2023, the searches were then updated to 2^nd^ June 2025. Medline was used in the original search and the updated search was done in PubMed.

Key-words are noted below in regular type, ‘MESH’ (Medical subject heading) terms in **bold**.

| **Search 1: Incarceration settings** |
| --- |
| Prison* OR Jail* or Detention* OR "Compulsory Drug Treatment" OR "Closed Setting" OR Prisoners* OR Criminal* OR Offender* OR Post-Release* OR Incarerat* OR **prisoners** |

| **Search 2: Injecting drug use** |
| --- |
| "IDU" OR "IDUs" OR "PWID" OR "injecting drug*" OR "intravenous drug*" OR "injecting substance*" OR "intravenous substance*" OR "people who inject*" OR "injection drug*" OR **substance abuse, intravenous** |

| **Search 3. HIV/AIDS** |
| --- |
| "HIV" OR "AIDS" OR "HIV/AIDS" OR “Human Immunodeficiency Virus” OR “Human Immune Deficiency Virus” OR “Acquired Immunodeficiency Syndrome” OR “Acquired Immune Deficiency Syndrome” OR "anti?retroviral" OR "anti?HIV agents" OR “highly active anti?retroviral therapy” OR “combination anti?retroviral therapy” OR "HAART" OR "cART" |

| **Search 4: Hepatitis** |
| --- |
| Hepatitis OR “hepatitis C” OR “hep C” OR HCV OR DAA OR "hepatitis B" or "hep B" or "HBV" OR **hepatitis b** OR **hepatitis c** |

| **Search 5: Needle syringe program** |
| --- |
| ("needle" AND ("exchange" OR "program*")) OR ("syringe" AND ("exchange" OR "program*")) OR **needle-exchange programs** OR **harm reduction** |

| **Search 6: Opioid agonist treatment** |
| --- |
| OST OR OAT OR “opioid substitution treatment” OR “methadone” OR “buprenorphine” OR “opioid replacement” OR “opioid substitution” OR “opiate substitution” OR “opioid agonist” OR “opiate agonist” OR “drug treatment” OR “medication assisted” OR “medication for opioid use disorder” OR “heroin assisted” OR “oral morphine” OR hydromorphone OR naltrexone OR "MAT" OR MOUD OR HAT OR **buprenorphine** OR **methadone** OR **opiate substitution treatment** OR **buprenorphine, naloxone drug combination** |

| **Search 7: Naloxone** |
| --- |
| Naloxone OR Narcan OR “take home naloxone” OR “take-home naloxone” OR THN OR **Naloxone** |

| **Search 8: COVID or HBV vaccinations** |
| --- |
| "COVID*" OR “SARS-CoV-2” OR **COVID-19 Vaccines** OR **Hepatitis B Vaccines** OR **COVID-19** |

| **Search 9: TB** |
| --- |
| **Tuberculosis** OR **“tuberculosis, multidrug-resistant”** OR **"Mycobacterium tuberculosis"** OR "Mycobacterium tuberculosis" OR “MDR-TB” OR “XDR-TB” OR “Mtb” OR (extensively AND **("drug resistance"** OR drug-resistant)) |

## 2.2. Embase Search Strategy

The search strategy was as follows:

Results were restricted to those articles containing terms from search 1 and terms from one or more results of searches 2-8, combined as 1 AND (2 OR 3 OR 4 OR 5 OR 6 OR 7 OR 8). Results were restricted to articles published 2000-current and limited to population group: humans.

Original searches were conducted in March 2023, the searches were then updated to 2^nd^ June 2025.

Key-words are noted in regular type, ‘EMTREE’ terms in **bold**.

| **Search 1: Incarceration settings** |
| --- |
| prison* OR jail OR gaol OR incarcerat* OR carceral OR detention OR compulsory and (drug OR treat* OR rehab*) OR offend* OR post-release OR custod* OR parole* OR probation OR crim* OR convict* OR detain* OR **prisons** OR **incarceration** OR **prisoners** OR **criminal** OR **parole** |

| **Search 2: Injecting drug use** |
| --- |
| IDU OR IDUs OR PWID OR "injecting drug*" OR "injecting substance*" OR "people who inject*" OR "injection drug*" OR **intravenous drug abuse/** OR **intravenous drug administration/** |

| **Search 3: HIV** |
| --- |
| HIV OR **HIV/** |

| **Search 4: Hepatitis** |
| --- |
| hepatitis OR "HBV" OR "hepatitis B" OR "hep B" OR "hep C" OR "HCV" OR "hepatitis C" OR **hepatitis B/** OR **hepatitis C/** |

| **Search 5: Needle syringe program** |
| --- |
| ("needle" AND ("exchange" OR "program*")) OR ("syringe" AND ("exchange" OR "program*")) OR “harm reduction” OR **needle-exchange programs/** OR **harm reduction/** |

| **Search 6: Opioid agonist treatment** |
| --- |
| "OST" OR "OAT" OR "MMT" OR "BMT" OR "MAT" OR "OUD" OR methadone OR buprenorphine* OR "opioid replacement" OR "opioid substitution" OR "opiate substitution" OR "opioid agonist" OR agonist OR "drug treatment" OR "medication assisted" OR "medication for opioid use disorder" OR "heroin assisted" OR "oral morphine" OR hydromorphone OR naltrexone OR "MOUD" OR "HAT" OR **buprenorphine** OR **methadone** OR **opiate substitution treatment** OR **buprenorphine, naloxone drug combination** |

| **Search 7: Naloxone** |
| --- |
| Naloxone OR Narcan OR THN OR **naloxone/** |

| **Search 8: COVID-19 or HBV vaccinations** |
| --- |
| "COVID*" OR “SARS-CoV-2” OR **COVID-19 Vaccines/** OR **Hepatitis B Vaccines/** OR **COVID-19/** |

| **Search 9: TB** |
| --- |
| **Tuberculosis/** OR'multidrug resistance' OR **Mycobacterium tuberculosis/ OR** 'mdr -tb' OR 'xdr -tb' OR 'mtb' OR 'tb' |

## 2.3. PsycINFO Search Strategy

The search strategy was as follows:

Results were restricted to those articles containing terms from search 1 and terms from one or more results of searches 2-8, combined as 1 AND (2 OR 3 OR 4 OR 5 OR 6 OR 7 OR 8). Results were restricted to articles published 2000-current and limited to population group: humans.

Original searches were conducted in March 2023, the searches were then updated to 2^nd^ June 2025.

Key-words in regular type, ‘PsycINFO thesaurus’ terms in **bold**.

| **Search 1: Incarceration settings** |
| --- |
| prison* OR jail OR gaol OR incarcerat* OR carceral OR detention OR compulsory and (drug OR treat* OR rehab*) OR offend* OR post-release OR custod* OR parole* OR probation OR crim* OR convict* OR detain* OR **prisons** OR **incarceration** OR **prisoners** OR **criminals** OR **parole** |

| **Search 2: Injecting drug use** |
| --- |
| IDU OR IDUs OR PWID OR “injecting drug*” OR “injecting substance*” OR "people who inject*" OR "injection drug*" OR (**"Intravenous Drug Usage"** OR **"Intravenous Injections"**) |

| **Search 3: HIV** |
| --- |
| HIV OR **"HIV"** |

| **Search 4: Hepatitis** |
| --- |
| hepatitis OR "HBV" OR "hepatitis B" OR "hep B" OR "hep C" OR "HCV" OR "hepatitis C" OR (**"Hepatitis"**) |

| **Search 5: Needle syringe program** |
| --- |
| ("needle" AND ("exchange" OR "program*")) OR ("syringe" AND ("exchange" OR "program*")) OR “harm reduction” OR (**"Needle-Exchange Programs"** OR **"Harm Reduction"**) |

| **Search 6: Opioid agonist treatment** |
| --- |
| ("OST" OR "OAT" OR "MMT" OR "BMT" OR "MAT" OR "OUD" “opioid substitution treatment” OR “methadone” OR “buprenorphine” OR “opioid replacement” OR “opioid substitution” OR “opiate substitution” OR “opioid agonist” OR “opiate agonist” OR “drug treatment” OR “medication assisted” OR “medication for opioid use disorder” OR “heroin assisted” OR “oral morphine” OR hydromorphone OR naltrexone OR "MOUD" OR "HAT").ti,ab. OR (**Buprenorphine** OR **Methadone** OR **opiate substitution treatment** OR **buprenorphine, naloxone drug combination**) |

| **Search 7: Naloxone** |
| --- |
| Naloxone OR Narcan OR THN OR (**"Naloxone"**) |

| **Search 8: COVID-19 and HBV vaccinations** |
| --- |
| "COVID*" OR “SARS-CoV-2” OR **COVID-19 Vaccines/** OR **Hepatitis B Vaccines/** OR **COVID-19/** |

| **Search 9: TB** |
| --- |
| **Tuberculosis/** OR 'multidrug resistance' OR 'mycobacterium tuberculosis' OR 'mdr-tb' OR 'xdr-tb' OR 'mtb' OR 'tb' |

## 2.4. Web of Science Search Strategy

The search strategy was as follows:

Results were restricted to those articles containing terms from search 1 and terms from one or more results of searches 2-8, combined as 1 AND (2 OR 3 OR 4 OR 5 OR 6 OR 7 OR 8). Results were restricted to articles published 2000-current and limited to population group: humans.

Original searches were conducted in March 2023, the searches were then updated to 2^nd^ June 2025.

Key-words in regular type.

| **Search 1: Incarceration settings** |
| --- |
| prison* or jail or gaol or incarcerat* or "carceral system" or detention or compulsory and (drug or treat* or rehab*) or "offend*" or post-release or custod* or parole* or probation or crim* or convict* OR prisons OR incarceration OR prisoners OR "Criminal Offenders" OR "Criminal Conviction" OR Parole |

| **Search 2: Injecting drug use** |
| --- |
| "IDU" or "IDUs" or "PWID" or "injecting drug*" or "injecting substance*" or "people who inject*" or "injection drug*" or "intravenous drug" OR "intravenous drug abuse" or "intravenous drug administration" |

| **Search 3: HIV** |
| --- |
| “HIV” OR HIV |

| **Search 4: Hepatitis** |
| --- |
| hepatitis or "HBV" or "hepatitis B" or "hep B" or "hep C" or "HCV" or "hepatitis C" OR "hepatitis c" or "hepatitis b" |

| **Search 5: Needle syringe program** |
| --- |
| "needle" AND ("exchange" OR "program*") OR "harm reduction" OR "needle-exchange programs" OR "harm reduction" |

| **Search 6: Opioid agonist treatment** |
| --- |
| "OST" OR "OAT" OR "MMT" OR "BMT" OR "MAT" OR "OUD" “opioid substitution treatment” OR “methadone” OR “buprenorphine” OR “opioid replacement” OR “opioid substitution” OR “opiate substitution” OR “opioid agonist” OR “opiate agonist” OR “drug treatment” OR “medication assisted” OR “medication for opioid use disorder” OR “heroin assisted” OR “oral morphine” OR hydromorphone OR naltrexone OR "MOUD" OR "HAT" OR Methadone or Buprenorphine or "opiate substitution treatment" or "naloxone drug combination" |

| **Search 7: Naloxone** |
| --- |
| Naloxone OR Narcan OR “take home naloxone” OR “take-home naloxone” OR "THN" OR Naloxone |

| **Search 8: COVID-19 and HBV vaccinations** |
| --- |
| "COVID*" OR “SARS-CoV-2” OR "COVID-19 Vaccines" OR "Hepatitis B Vaccines" OR COVID-19 |

| **Search 9: TB** |
| --- |
| Tuberculosis OR 'mycobacterium tuberculosis' OR TB OR “MDR-TB” OR “XDR-TB” |

## 2.5. CINAHL Search Strategy

The search strategy was as follows:

Results were restricted to those articles containing terms from search 1 and terms from one or more results of searches 2-8, combined as 1 AND (2 OR 3 OR 4 OR 5 OR 6 OR 7 OR 8). Results were restricted to articles published 2000-current and limited to population group: humans.

Original searches were conducted in March 2023, the searches were then updated to 2^nd^ June 2025.

Key-words in regular type, ‘CINAHL Subject Headings’ terms in **bold**.

| **Search 1: Incarceration settings** |
| --- |
| Prisoners OR Prison* OR Jail* OR Detention* OR "Compulsory drug treatment" OR "Closed setting" OR Prisoners* OR Criminal* OR Offender* OR Post-release* |

| **Search 2: Injecting drug use** |
| --- |
| "IDU" OR "IDUs" OR PWID OR "injecting drug*" OR "intravenous drug*" OR "injecting substance*" OR "intravenous substance*" OR "people who inject*" OR "injection drug*" OR "substance abuse" OR intravenous |

| **Search 3: HIV** |
| --- |
| "HIV" OR "AIDS" OR "HIV/AIDS" OR “Human Immunodeficiency Virus” OR “Human Immune Deficiency Virus” OR “Acquired Immunodeficiency Syndrome” OR “Acquired Immune Deficiency Syndrome” |

| **Search 4: Hepatitis** |
| --- |
| (HCV or hepatitis C) and (test* or incidence* or prevalence*) |

| **Search 5: Needle syringe program** |
| --- |
| "needle" and ("exchange" OR "program*") OR "syringe" and "exchange" OR "program*") OR "harm reduction" OR "NSP" |

| **Search 6: Opioid agonist treatment** |
| --- |
| (((opioid OR opiate) AND (subtitut* OR maintenance) AND treatment) OR methadone OR buprenorphinie OR ((medication* adj3 ("OUD" or ((opiate or opioid or heroin) and (dependen* or addict*)))) or "medication-assisted treatment" or "LAAM") OR "Narcotic Agonists" OR ((Opioid OR Opiate) AND "agonist treatment") OR ("OAT" OR "OST" OR "MMT" OR "BMT" OR "MAT")) |

| **Search 7: Naloxone** |
| --- |
| Naloxone or (naloxone and (provision or program or programme)) or "THN" |

| **Search 8: COVID-19 and HBV vaccinations** |
| --- |
| ("COVID*" or "SARS-CoV-2" or ("Hepatitis B" or "Hep B" or "HBV")) and (Vaccin* or immunisation or immunization or "vaccination coverage") |

| **Search 9: TB** |
| --- |
| Tuberculosis OR ‘mycobacterium tuberculosis’ OR “TB” OR “mdr-tb” OR “xdr-tb OR “mtb” |

# Appendix 3: Grey Literature Search

A wide range of online databases and websites were searched for additional information to that collected in the peer reviewed literature (see Table 3.1 below for full list). These databases are crucial sources of information on the epidemiology of injecting drug use and blood-borne virus among injecting drug users because so much of the work in this area appear to be published only in the form of reports. In total, we searched 145 websites and databases, including: government and substance use organisation websites, ongoing study sites, harm reduction organisation websites and infectious disease databases. The full technical report of grey literature sources is published elsewhere. ^1^

The search terms used in the grey literature search were similar to those used in the peer review search and are listed below. The ‘Google Advanced Search’ option was used to search for these terms within each website. Manual searching, involving clicking through relevant sections of the website in order to find relevant documents, was also conducted on some websites, particularly when the Google Advanced Search produced no results. Websites in languages other than English were also searched by translating the below search terms into English through ‘Google Translate’. All searches were conducted in October 2023, with additional searches up until December 2024.

**Grey Literature Search Strategy:**

| **Search 1: Carceral Settings** |
| --- |
| prison OR criminal OR justice OR jail OR correctional OR detention OR incarcerated OR incarceration OR custody |

| **Search 2: Health** |
| --- |
| hiv OR "human immunodeficiency virus" OR hepatitis OR hcv OR hbv OR bbv OR inject OR needle OR nsp OR intravenous OR pwid OR condom OR opioid OR opiate OR heroin OR narcotic OR methadone OR buprenorphine OR fentanyl OR "OST" OR methamphetamine OR stimulant OR amphetamine OR cocaine OR "crack" OR naloxone OR naltrexone OR tuberculosis |

## Table 3.1: Websites searched in the grey literature search

| **Region** | **Website Name and Link** | **Search Strategy** | **Search Results (N)^1^** | **Sources/Web Pages with Relevant Data (N)**^2^ |
| --- | --- | --- | --- | --- |
| **Global** | Harm Reduction International  <https://hri.global/> | Search 1: Carceral Settings | 291 | 3 |
| **Global** | National Drug & Alcohol Research Centre  <https://ndarc.med.unsw.edu.au/> | Search 1: Carceral Settings | 256 | 1 |
| **Global** | International Narcotics Control Board  <https://www.incb.org/> | Search 1: Carceral Settings | 0 | 0 |
| **Global** | International Narcotics Control Board  <https://www.incb.org/> | Search 1: Carceral Settings | 290 | 0 |
| **Global** | SALIS (Substance Abuse Librarians & Information Specialists)  <https://salis.org> | Search 1: Carceral Settings | 1 | 0 |
| **Global** | International Treatment Preparedness Coalition (ITPC)  <https://itpcglobal.org> | Search 1: Carceral Settings  Manual | 0  24 | 0  2 |
| **Global** | United Nations Office on Drugs and Crime  <http://www.unodc.org> | Manual | 4340 | 0 |
| **Global** | United Nations Office on Drugs and Crime  <https://dataunodc.un.org> | Search 1: Carceral Settings | 3 | 0 |
| **Global** | United Nations Office on Drugs and Crime  <https://www.unodc.org/unodc/en/treatment-and-care/publications.html> |  | n/a | n/a |
| **Global** | United Nations Office on Drugs and Crime  <https://www.unodc.org/unodc/en/justice-and-prison-reform/cpcj-tools-prisonreform.html> |  | n/a | n/a |
| **Global** | United Nations Office on Drugs and Crime  <https://www.unodc.org/unodc/en/crimecongress/about.html> |  | n/a | n/a |
| **Global** | United Nations Office on Drugs and Crime  <https://www.unodc.org/unodc/en/commissions/CND/index.html> | Search 1: Carceral Settings | 1 | 0 |
| **Global** | United Nations Office on Drugs and Crime  <https://www.unodc.org/unodc/en/commissions/CCPCJ/index.html> | Search 1: Carceral Settings | 1 | 0 |
| **Global** | United Nations Office on Drugs and Crime  <https://www.unodc.org/unodc/en/commissions/CCPCJ/PNI/institutes-UNAFEI.html> | Search 1: Carceral Settings | 1 | 0 |
| **Global** | United Nations Interregional Crime and Justice Research Institute  <https://unicri.it/> | Search 1: Carceral Settings | 302 | 0 |
| **Global** | World Health Organization  <https://www.who.int/europe/home> | Search 1: Carceral Settings | 0 | 0 |
| **Global** | World Health Organization  <https://www.who.int/data/gho> | Search 1: Carceral Settings | 1 | 0 |
| **Global** | World Health Organization  <https://www.who.int/teams/mentalhealth-and-substance-use/alcohol-drugsand-addictive-behaviours/overview> | Search 1: Carceral Settings | 276 | 0 |
| **Global** | United Nations Statistics Division  <https://unstats.un.org/home/nso_sites> | Search 1: Carceral Settings | 280 | 0 |
| **Global** | National Commission on Correctional Health Care  <https://www.ncchc.org/> | Search 1: Carceral Settings | 300 | 0 |
| **Global** | Penal Reform International  <https://www.penalreform.org/globalprison-trends-2022> | Manual | 1 | 1 |
| **Global** | International Committee of the Red Cross  <https://www.icrc.org/en> | Manual | 271 | 0 |
| **Global** | The Worldwide Prison Health Research & Engagement Network (WEPHREN)  <https://wephren.tghn.org> | Search 2: Health | 186 | 0 |
| **Global** | Coalition for Global Hepatitis Elimination  <https://www.globalhep.org/data-dashboards/national-hepatitis-elimination-profiles> | Manual | 1 | 1 |
| **Global** | HIV and AIDS Data Hub  <https://www.aidsdatahub.org> | Search 1: Carceral Settings | 70 | 0 |
| **Global** | Eurasian Harm Reduction Association  <https://harmreductioneurasia.org/drug-policy/criminalization-costs-2> | Search 1: Carceral Settings | 244 | 2 |
| **Global** | Virtual AIDS Office of Hong Kong  <https://www.aids.gov.hk/english/surveillance/off_surreport.html> | Search 1: Carceral Settings | 100 | 0 |
| **Global** | Narcotics Division, Security Bureau – The Government of the Hong Kong Special Administrative Region of the People’s Republic of China  <https://www.nd.gov.hk/en/index.html> | Manual | 308 | 0 |
| **Global** | Nai Zindagi Trust  <https://www.naizindagi.org/> | Search 1: Carceral Settings | 30 | 0 |
| **Global** | Recovering Nepal  [www.recoveringnepal.org.np](http://www.recoveringnepal.org.np) | Search 1: Carceral Settings | 20 | 0 |
| **Global** | Be in the Know  <https://www.beintheknow.org/understanding-hiv-epidemic/community/hiv-and-prisoners> | Search 1: Carceral Settings  Manual | 0  0 | 0  0 |
| **Global** | FHI360  <https://www.fhi360.org> | Search 1: Carceral Settings | 200 | 0 |
| **Global** | Global Network of People Living with HIV  <https://gnpplus.net/> | Search 1: Carceral Settings | 192 | 0 |
| **Global** | Open Society Foundations  <https://www.opensocietyfoundations.org/voices/%20topics/criminal-justice> | Search 1: Carceral Settings | 1 | 0 |
| **Global** | HIV and AIDS Data Hub  <https://www.aidsdatahub.org/resource/technicalbrief-addressing-hiv-and-tb-prisons-pre-trialdetention-and-other-closed> | Search 1: Carceral Settings | 2 | 0 |
| **Global** | The Global Fund  <https://www.theglobalfund.org/en> | Search 1: Carceral Settings  Manual | 0  1120 | 0  1 |
| **Global** | United Nations Development Programme  <https://www.undp.org> | Search 1: Carceral Settings  Manual | 0  4083 | 0  0 |
| **Global** | United Nations Population Fund  <https://www.unfpa.org> | Search 1: Carceral Settings | 2 | 0 |
| **Asia** | HIV and AIDS Data Hub  <https://www.aidsdatahub.org/> | Search 1: Carceral Settings | 295 | 1 |
| **Asia** | Eurasian Harm Reduction Association  [https://harmreductioneurasia.org/dru g-policy/criminalization-costs-2](https://harmreductioneurasia.org/dru%20g-policy/criminalization-costs-2) | Manual | 29 | 29 |
| **Asia** | Virtual AIDS Office of Hong Kong  [https://www.aids.gov.hk/english/surv eillance/off_surreport.html](https://www.aids.gov.hk/english/surv%20eillance/off_surreport.html) | Search 1: Carceral Settings | 0 | 0 |
| **Asia** | Virtual AIDS Office of Hong Kong  [https://www.aids.gov.hk/english/surv eillance/off_surreport.html](https://www.aids.gov.hk/english/surv%20eillance/off_surreport.html) | Manual | 23 | 1 |
| **Asia** | Narcotics Division, Security Bureau – The Government of the Hong Kong Special Administrative Region of the People’s Republic of China  <https://www.nd.gov.hk/en/index.html> | Search 1: Carceral Settings  Manual | 0  309 | 0  0 |
| **Asia** | Nai Zindagi Trust  <https://www.naizindagi.org> | Search 1: Carceral Settings | 30 | 0 |
| **Asia** | Recovering Nepal  [www.recoveringnepal.org.np](http://www.recoveringnepal.org.np) | Search 1: Carceral Settings | 26 | 0 |
| **Asia** | The Asia Foundation  <https://asiafoundation.org> | Search 1: Carceral Settings  Manual | 0  1 | 0  0 |
| **Asia** | International Drug Policy Consortium  <https://idpc.net> | Manual | 107 | 0 |
| **Asia** | National AIDS Control Organisation  <http://naco.gov.in/> | Search 1: Carceral Settings | 251 | 2 |
| **Asia** | National Center for AIDS and STD Control – Government of Nepal – Ministry of Health and Population  <https://www.ncasc.gov.np> | Search 1: Carceral Settings  Manual | 0  1 | 0  1 |
| **Asia** | Bureau of Jail Management and Penology  <https://www.bjmp.gov.ph> | Search 2: Health | 2 | 0 |
| **Asia** | Correctional Services Department Hong Kong  <https://www.csd.gov.hk/tc_chi/home/home.html> | Search 2: Health  Manual | 0  0 | 0  0 |
| **Asia** | Ministry of Justice – Agency of Corrections  <https://www.mjac.moj.gov.tw> | Search 2: Health | 104 | 2 |
| **Asia** | Singapore Prison Service  <https://www.sps.gov.sg> | Search 2: Health | 6 | 1 |
| **Asia** | Malaysian Prison Department  <https://www.prison.gov.my/ms> | Search 2: Health | 23 | 0 |
| **Asia** | Prisons Organization of Iran  <https://www.prisons.ir> | Search 2: Health | 8 | 1 |
| **Australasia** | Australian Institute of Health and Welfare  <https://www.aihw.gov.au> | Search 1: Carceral Settings | 256 | 1 |
| **Australasia** | Australian Government – Attorney-General’s Department  <https://www.ag.gov.au/crime/federaloffenders/state-and-territorycorrective-services-websites> | Search 2: Health | 86 | 1 |
| **Australasia** | Department of Health and Aged Care  http://www.health.gov.au | Search 1: Carceral Settings | 249 | 0 |
| **Australasia** | Alcohol and Drug Foundation  <https://adf.org.au> | Search 1: Carceral Settings | 247 | 0 |
| **Australasia** | Ministry of Health Manatu Hauora  <https://www.health.govt.nz/nzhealth-statistics/health-statistics-anddata-sets/prisoner-health-data-andstats> | Search 1: Carceral Settings | 1 | 0 |
| **Australasia** | Kirby Institute  <http://kirby.unsw.edu.au/> | Search 1: Carceral Settings  Manual | 0  11 | 0  2 |
| **Australasia** | Justice and Community Safety Directorate  <https://justice.act.gov.au> | Search 2: Health | 2 | 0 |
| **Australasia** | Department of Justice – Government of Western Australia  <https://www.wa.gov.au/organisation/department-of-justice> | Search 2: Health | 1 | 0 |
| **Australasia** | Justice and Community Safety – Victoria State Government  <https://www.justice.vic.gov.au> | Search 2: Health | 15 | 0 |
| **Australasia** | Department of Corrections – Ara Poutama Aotearoa  <http://www.corrections.govt.nz> | Search 2: Health | 20 | 0 |
| **Europe** | European Union Drugs Agency  <https://www.emcdda.europa.eu/topics/prison_en> | Search 1: Carceral Settings  Manual | 1  71 | 0  26 |
| **Europe** | Department of Health & Social Care United Kingdom Government  <https://www.gov.uk/health-andsocial-care/drug-misuse-and-dependency> | Search 1: Carceral Settings  Manual | 0  27 | 0  2 |
| **Europe** | Federal Ministry Republic of Austria – Social Affairs, Health, Care and Consumer Protection  <https://www.sozialministerium.at/en.html> | Search 1: Carceral Settings  Manual | 0  0 | 0  0 |
| **Europe** | Sciensano  <https://www.sciensano.be/en> | Search 1: Carceral Settings | 85 | 0 |
| **Europe** | Ministry of Health Bulgaria  <https://www.mh.government.bg/%20bg/novini/aktualno/v-sofiiskiiazatvor-shche-uchat-kak-da-sepaziat-o/> | Search 1: Carceral Settings  Manual | 0  1 | 0  1 |
| **Europe** | National Monitoring Center for Drugs and Addiction [https://www.drogyinfo.cz/data/obj_files/33369/107 3/VZdrogy2019_www_fin.pdf](https://www.drogyinfo.cz/data/obj_files/33369/107%203/VZdrogy2019_www_fin.pdf) | Search 1: Carceral Settings  Manual | 0  0 | 0  0 |
| **Europe** | Statens Serum Institut  <https://en.ssi.dk/news/epinews/2016/no-9---2016> | Search 1: Carceral Settings | 8 | 0 |
| **Europe** | DrugWise  <http://www.drugwise.org.uk/> | Search 1: Carceral Settings | 296 | 0 |
| **Europe** | United Kingdom Government  <https://www.gov.uk/government%20/organisations/public-healthengland> | Search 1: Carceral Settings  Manual | 0  0 | 0  0 |
| **Europe** | The French Monitoring Centre for Drugs and Addiction (OFDT)  [https://en.ofdt.fr/publications/m emo-posters-maps/drug-useprison-practices-consequencesand-responses-summary/](https://en.ofdt.fr/publications/m%20emo-posters-maps/drug-useprison-practices-consequencesand-responses-summary/) | Search 1: Carceral Settings | 1 | 1 |
| **Europe** | Sante Publique France  <https://www.santepubliquefrance.fr/recherche/#search=prison> |  | n/a | n/a |
| **Europe** | Robert Koch Institut  <https://www.rki.de/SiteGlobals/Forms/Suche/en/serviceSucheFormen> | Search 1: Carceral Settings  Manual | 0  24 | 0  0 |
| **Europe** | Public Health Scotland  <https://www.hps.scot.nhs.uk/search?q=prison> | Search 1: Carceral Settings | 9 | 0 |
| **Europe** | Public Health Scotland  <https://www.isdscotland.org/search/?q=prison> | Search 1: Carceral Settings | 229 | 2 |
| **Europe** | The Scottish Public Health Observatory  <https://www.scotpho.org.uk/search> | Search 1: Carceral Settings | 153 | 1 |
| **Europe** | National Institute of Health Italy  http://www.iss.it | Search 1: Carceral Settings | 216 | 0 |
| **Europe** | Ministry of Health of the Republic of Lithuania  <http://sam.lrv.lt/> | Search 1: Carceral Settings | 63 | 1 |
| **Europe** | National Institute for Public Health and the Environment Netherlands  <https://www.rivm.nl/en/search?search=prison> | Search 1: Carceral Settings | 19 | 0 |
| **Europe** | Instituto de Salud Carlos III  [https://www.isciii.es/en/layouts/15/osssearchresults.aspx#k=prisi%C%203%B3n#s=11](https://www.isciii.es/en/layouts/15/osssearchresults.aspx#k=prisi%C%203%B3n) | Search 1: Carceral Settings | 21 | 0 |
| **Europe** | Federal Office of Public Health Switzerland  [https://www.bag.admin.ch/bag/e n/home/strategie-undpolitik/nationalegesundheitsstrategien/nationales-programm-hiv-und-anderesexuell-uebertragbareinfektionen/zielgruppe-miterhoehtem-expositionsrisikoachse2/bekampfung-voninfektionskrankheiten-imgefangnis.html](https://www.bag.admin.ch/bag/e%20n/home/strategie-undpolitik/nationalegesundheitsstrategien/nationales-programm-hiv-und-anderesexuell-uebertragbareinfektionen/zielgruppe-miterhoehtem-expositionsrisikoachse2/bekampfung-voninfektionskrankheiten-imgefangnis.html) | Search 1: Carceral Settings | 23 | 0 |
| **Europe** | Eurasian Harm Reduction Association  <https://harmreductioneurasia.org/> | Search 1: Carceral Settings | 244 | 0 |
| **Europe** | French National AIDS Council  <https://cns.sante.fr/> | Search 1: Carceral Settings | 205 | 1 |
| **Europe** | Health Protection Surveillance Centre  <https://www.hpsc.ie> | Search 1: Carceral Settings | 233 | 1 |
| **Europe** | Country Coordinating Mechanism of Moldova  <http://www.ccm.md/index.php/> | Search 1: Carceral Settings | 8 | 0 |
| **Europe** | UK Harm Reduction Alliance  <https://www.ukhra.org/index.html> | Search 1: Carceral Settings  Manual | 0  0 | 0  0 |
| **Europe** | Ministry of Justice Republic Moldova  <http://anp.gov.md/> | Search 2: Health | 50 | 1 |
| **Europe** | Prison Service of the Czech Republic  <https://www.vscr.cz/> | Search 2: Health | 77 | 1 |
| **Europe** | National Penitentiary Administration Romania  <https://anp.gov.ro/> | Search 2: Health | 290 | 0 |
| **Europe** | Prison Service Estonia  <https://www.vangla.ee> | Search 2: Health | 3 | 1 |
| **Europe** | Prison Service Poland  <https://sw.gov.pl> | Search 2: Health | 229 | 0 |
| **Europe** | Special Penitentiary Service of Georgia  <http://www.sps.gov.ge> | Search 2: Health | 44 | 0 |
| **Europe** | Service Public Federal Justice  <https://justice.belgium.be/fr/themes_et_dossiers/prisons> | Search 2: Health | 1 | 0 |
| **Europe** | Directorate-General for Reintegration and Prison Services Portugal  <https://dgrsp.justica.gov.pt> | Search 2: Health | 35 | 0 |
| **Europe** | Norwegian Correctional Service  <https://www.kriminalomsorgen.no> | Search 2: Health | 23 | 0 |
| **Europe** | Irish Prison Service  <https://www.irishprisons.ie> | Search 2: Health | 92 | 0 |
| **Europe** | Ministry of Justice France  <http://www.justice.gouv.fr> | Search 2: Health  Manual | 1  8 | 0  0 |
| **Europe** | Department of Justice Northern Ireland  <https://www.justice-ni.gov.uk/topics/prisons> | Search 2: Health  Manual | 0  0 | 0  0 |
| **Europe** | Ministry of Justice Albania  <https://dpbsh.gov.al> | Search 2: Health | 18 | 0 |
| **Europe** | Scottish Prison Service  <https://www.sps.gov.uk/> | Search 2: Health | 2 | 0 |
| **Europe** | Directorate of the Danish Prison and Probation Service  <https://www.kriminalforsorgen.dk/> | Search 1: Carceral Settings | 188 | 0 |
| **Europe** | Criminal Sanctions Agency Finland  <https://rikosseuraamus.fi/> | Search 1: Carceral Settings | 293 | 0 |
| **Europe** | Ministry of Justice – Directorate for the Execution of Criminal Sanctions Serbia  <http://www.uiks.mpravde.gov.rs/> | Search 1: Carceral Settings | 3 | 0 |
| **Europe** | HM Prison Service – Public Sector Prisons UK  <https://www.gov.uk/government/organisations/hm-prison-service> | Search 2: Health | 2 | 0 |
| **Europe** | Ministry of Justice Italy  <https://www.giustizia.it/giustizia/> | Search 2: Health | 40 | 0 |
| **Africa** | Ministry of Health and Wellness Mauritius  <https://health.govmu.org/Pages/default.aspx> | Search 1: Carceral Settings  Manual | 0  2 | 0  1 |
| **Africa** | Ghana AIDS Commission  <https://www.ghanaids.gov.gh/> | Search 1: Carceral Settings  Manual | 0  6 | 0  1 |
| **Africa** | The National Syndemic Diseases Control Council (NSDCC)  <https://nsdcc.go.ke/> | Search 1: Carceral Settings  Manual | 0  11 | 0  0 |
| **Africa** | National AIDS Council Zimbabwe  <https://www.nac.org.zw/> | Search 1: Carceral Settings  Manual | 0  8 | 0  0 |
| **Africa** | The South African National AIDS Council  <http://www.sanac.org.za/> | Search 1: Carceral Settings  Manual | 0  7 | 0  0 |
| **Africa** | Ministry of Justice - Prisons Directorate Lebanon  <http://pa.justice.gov.lb/index.php> | Search 1: Carceral Settings  Manual | 0  0 | 0  0 |
| **Africa** | Sierra Leone Correctional Service  <https://slcs.gov.sl/> | Search 2: Health  Manual | 0  0 | 0  0 |
|  | Nigerian Correctional Service  <https://corrections.gov.ng/> | Search 2: Health | 4 |  |
| **Africa** | Malawi Prisons Service  <https://www.mps.gov.mw/> | Search 1: Carceral Settings | 30 | 0 |
| **Africa** | Seychelles Prison Service  <http://www.prisonservice.gov.sc/> | Search 1: Carceral Settings | 20 | 0 |
| **Americas** | Correctional Service of Canada  <https://www.csc-scc.gc.ca/health/002006index-en.shtml> | Search 1: Carceral Settings  Manual | 118  407 | 1  2 |
| **Americas** | Canadian Centre on Substance Use and Addiction  <https://www.ccsa.ca/> | Search 1: Carceral Settings  Manual | 59  401 | 0  0 |
| **Americas** | Government of Canada  <https://www.canada.ca/en/publichealth.html> | Search 1: Carceral Settings | 374 | 3 |
| **Americas** | Substance Abuse and Mental Health Services Administration  <https://www.samhsa.gov/> | Search 1: Carceral Settings | 684 | 0 |
| **Americas** | Bureau of Justice Statistics  <https://bjs.ojp.gov> | Search 2: Health  Manual | 120 | 3  0 |
| **Americas** | Correctional Health – Centers for Disease Control and Prevention  <https://www.cdc.gov/correctionalhealth/> | Search 2: Health | 120 | 0 |
| **Americas** | Partnership to End Addiction  <https://drugfree.org/> | Search 1: Carceral Settings | 10 | 0 |
| **Americas** | National Center for Health Statistics - Centers for Disease Control and Prevention  <https://www.cdc.gov/nchs/index.htm> | Search 1: Carceral Settings  Manual | 284  621 | 1  7 |
| **Americas** | The White House  <https://www.whitehouse.gov/ondcp> | Search 1: Carceral Settings | 96 | 0 |
| **Americas** | Inter-American Drug Abuse Control Commission  <http://www.cicad.oas.org/main/default_eng.asp> | n/a | n/a | n/a |
| **Americas** | National Prevention Information Network – Centers for Disease Control and Prevention  <https://npin.cdc.gov/> | n/a | n/a | n/a |
| **Americas** | National Harm Reduction Coalition  <https://harmreduction.org/> | Search 1: Carceral Settings | 142 | 0 |
| **Americas** | Department of HIV, AIDS, Tuberculosis, Viral Hepatitis and Sexually Transmitted Infections Brazil  <http://www.aids.gov.br> | Search 1: Carceral Settings | 50 | 0 |
| **Americas** | HIV Legal Network Canada  <https://www.hivlegalnetwork.ca/site/?lang=en> | Search 1: Carceral Settings | 295 | 0 |
| **Americas** | The National Council for Comprehensive HIV AIDS Care (CONASIDA)  <http://www.conasida.go.cr/> | Search 1: Carceral Settings | 172 | 0 |
| **Americas** | Ministry of Health and Wellness Jamaica  <https://www.moh.gov.jm/> | Search 1: Carceral Settings | 72 | 0 |
| **Americas** | Department of Correctional Services Jamaica  <https://www.dcs.gov.jm/> | Search 2: Health | 5 | 0 |
| **Americas** | Department of Corrections and Rehabilitation Puerto Rico  <http://dcr.pr.gov/> | Search 1: Carceral Settings | n/a | n/a |
| **Americas** | Ministry of Home Affairs, Justice and National Security  <https://homeaffairs.govt.lc/> | Search 1: Carceral Settings | 20 | 0 |
| **Americas** | National Secretariat for Penal Policies – Ministry of Justice and Public Security Brazil  <https://www.gov.br/depen/pt-br> | Manual | 173 | 0 |
| **Americas** | INPEC National Penitentiary and Prison Institute Colombia  <https://www.inpec.gov.co/> | Search 1: Carceral Settings | 294 | 0 |
| **Americas** | General Directorate of the Penitentiary System Guatemala  https://dgsp.gob.gt | Search 1: Carceral Settings | 201 | 0 |
| **Americas** | Guyana Prison Service  <https://gps.moha.gov.gy/> | Search 1: Carceral Settings | 51 | 0 |
| **Americas** | General Directorate of the Penitentiary System Panama  <https://www.sistemapenitenciario.gob.pa/> | Search 1: Carceral Settings | 299 | 0 |
| **Americas** | Ministry of People's Power for the Penitentiary Service Venezuela  <https://www.mppsp.gob.ve/> | Search 1: Carceral Settings | 283 | 0 |
| **Americas** | Ministry of Justice and Public Security El Salvador  <https://www.seguridad.gob.sv/> | Search 1: Carceral Settings | 282 | 0 |
| **Americas** | Statistics Canada  <https://www.statcan.gc.ca/en/subjectsstart/crime_and_justice/correctional_services> | Search 1: Carceral Settings | 256 | 0 |

**Notes: 1. Search results** refers to sources/web pages found in search results that had potential data for inclusion **2. Sources/Web Pages with Relevant Data** refers to sources that were included for full text review

# Appendix 4: Additional requests for information

## 4.1 Example email

*“We hope this message finds you well.*

*We are conducting a global review of the prevalence and treatment practices related to blood-borne viruses and injecting drug use among incarcerated populations.*

*Perhaps you recall our previous work on the epidemiology of injecting drug use, the prevalence of injecting-related harm, and exposure to behavioural and environmental risks among people who inject drugs (see attached). That research was intended to inform the provision of services, such as blood-borne virus treatment, for people who inject drugs globally.*

*In our current review, we aim to extend our work to estimate the prevalence of injecting drug use, blood-borne viruses, and related harms, and summarise the state of treatment coverage within prisons for injecting drug use and related harms. A summary of these reviews is attached for your reference.*

*We are keen to gain access particularly to reports and papers that may not be accessible online. Given your expertise, we were hoping you could assist by providing any reports or data you may be aware of related to injecting drug use, HIV, HCV, other infectious diseases and treatment among people who are incarcerated?*

*We will acknowledge all people who submit literature for our consideration in our review.*

*Thank you for considering this request.”*

**4.2 Social media advertising**

An advertisement was posted on Facebook and Twitter to request relevant information during April-June 2024.

### 4.2.1 Facebook advertisement example

**
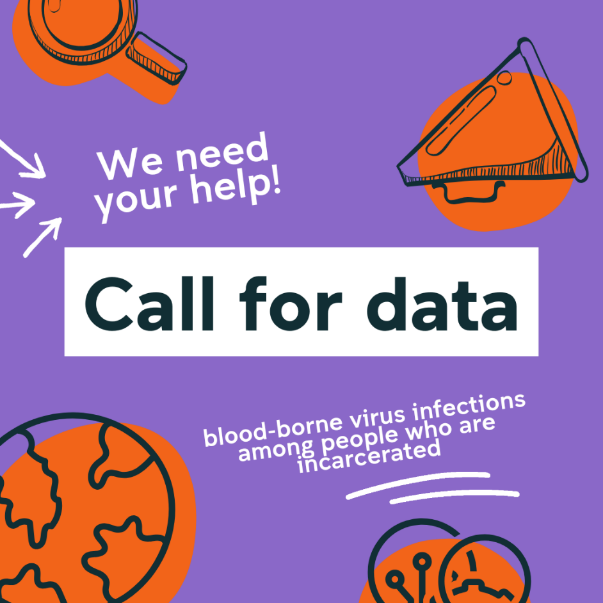
**

### 4.2.2 Twitter (X) advertisement example


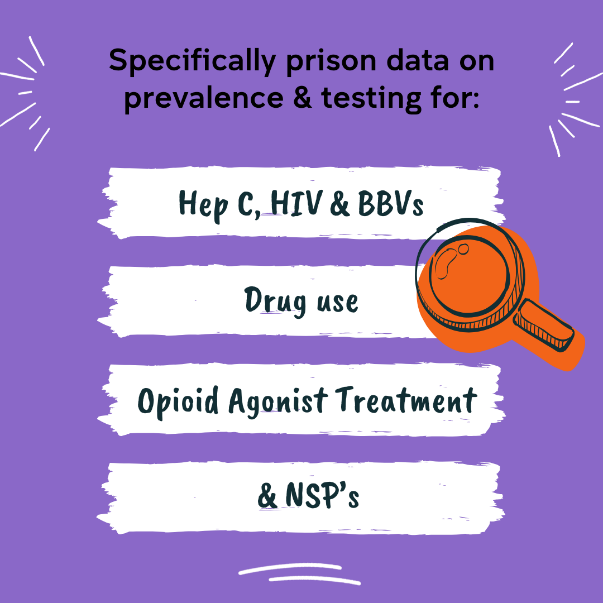


# Appendix 5.1: Screening Process and Criteria

Screening was conducted in two stages using Covidence (Veritas Health Innovation, 2024). The first stage involved title and abstract screening, which was conducted independently by two reviewers for each record. Reviewers contributing to this stage were Sophia Taylor (ST), Louisa Degendardt (LD), Michelle Lynch (ML), Sophie Ottaviano (SO), Aleksa Kamenjaš (AK), Paige Webb (PW), Benson Yiu (BY), Brodie Clark (BC), Marion Barault (MB), Jason Grebely (JG), Thomas Santo Jr (TS), Rachel Jenkins (RJ), Olivia Price (OP), Behzad Hajarizadeh (BH), and Evan Cunningham (EC). Discrepancies were resolved through discussion, and when consensus could not be reached, a third reviewer was consulted.

The second stage involved full-text review of all records deemed potentially eligible, which was also conducted independently by two reviewers for each record. Reviewers contributing to this stage were AK, ST, BY, Jack Marsden (JM), ML, LD, Jonathan Wu (JW), Noni O’Dea (NO), MB, TS, BC, Eunice Ling (EL), OP, and Christopher Manning (CM). Discrepancies were resolved by consensus, with a third reviewer involved when necessary. While most reviewers contributed to both title and abstract screening and full-text review, some additional reviewers participated in only one of these stages due to changes in team personnel during the course of the review.

**Inclusion criteria**

- Primary observational studies of people in prisons or other carceral settings that are likely to include one of the following outcomes:
  - Any study reporting on the correct sample, that is people in incarceration settings and reporting on infectious diseases or drug use
- Papers with information on imprisonment rates or estimated prison population sizes
- Relevant 100% subpopulation samples (e.g. 100% BBV, 100% Tuberculosis, 100% IDU etc.)
- Relevant systematic reviews for reference checking

**Exclusion Criteria**

- Commentary, editorial, or review
- Case study, or sample size < 40 incarcerated people
- No primary data presented
- Modelling studies
- Methodology not reported
- Delphi, consensus, or government estimate with no methodology reported
- Duplicate, or conference abstract where full report is not available
- Registration or notification of cases as an indicator of # of people positive
- Non-human sample
- People in closed psychiatric settings
- Arrestee samples (exclude ADAM samples)
- Samples of people post-release
- Subpopulations of people who are incarcerated
  - Samples of specific race groups (e.g. African American incarcerated population)
  - Samples of offenders who are referred to mental health or hospital services whilst admitted
  - Samples of specific offence types (e.g. Sexual offenders, violent offenders), with the exemption of people incarcerated for drug offences
  - 100% negative infectious disease sample (e.g. All inmates were anti-HCV ab negative)
  - Samples of people incarcerated who have never used illicit drugs.

# Appendix 5.2: Summary of decision rules for data extraction and estimation processes

*Decision rules for identifying data for extraction:*

1. Preference is for the most recent, national data for each outcome. Recency is based on the year for which the data of each outcome was generated rather than year of publication.
2. If only sub-national data are available, extraction noted as sub-national sample with designation of the area that it is referring to. Priority is placed on lager sub-national samples (i.e. A state sample over a city sample).
3. If both national data and sub-national data are available (and in cases where sub-national data may be more recent), extract the sub-national data in the notes field of the extraction on REDCap.
4. The number of unique individual clients accessing a service over a designated period and the total number of service events (i.e. number of tests) over a designated period are extracted as separate outcomes.
5. If only one piece of data on the number of clients accessing a service is available and does not explicitly state whether it is referring to ‘individual clients’ or ‘service events’ then this data can be taken as ‘service events’ and the ambiguity of the data and the extraction made is noted as such.

*General extraction instructions:*

1. We are developing coverage estimates by most recent year of data available and different time periods for estimates are to be noted (i.e. 12 months, a range e.g. June to December 2022, or a snapshot of a specific point in time).
2. If there are multiple years or time-periods of data available for a service, the most recent is to be extracted and the prior periods to be noted on the REDCap form.
3. If the year of estimate has not been specified in a paper, then the year of publication is to be used and this is to be noted on the REDCap form.
4. If the timeframe of data is presented as a range, e.g. 2020-2023, then the most recent year of data will be extracted as the year of estimate, this would be 2023 from this example, and the full range noted on the REDCap form.
5. The number of facilities and the type of facilities offering a service is to be extracted if available

*OAT:*

1. Only data for opioid agonist treatment were included under OAT. Opioids provided for the purposes of detoxification as opposed to maintenance were excluded.
2. OAT types included in this review:
3. Any unspecified OAT
4. Methadone
5. Buprenorphine
6. Buprenorphine/naloxone
7. Depot long-acting buprenorphine
8. If a source(s) state that one type of OAT is available and another is states a type not being available, extract the information for the type(s) that is available and extract ‘No not present’ for the type(s) that are not available.

*NSPs:*

1. Different terminology was used in the literature – the term ‘needle syringe programme’ or ‘NSP’ can refer to a single site or may refer to multiple sites within the carceral system of a given country
2. In most cases the number of sterile needles and syringes distributed will be reported as a single number.
3. In some cases, the total number of syringes and the total number of needles and/or clients may be provided separately – in such cases enter both separately into the relevant fields.

*HIV Testing:*

1. Any type of HIV testing in carceral settings can be used to indicate availability of HIV testing in carceral settings within that country.

*HIV Treatment /* Antiretroviral treatment:

1. Only data on the availability of antiretroviral therapy should be included for extraction of HIV treatment availability. Any other types of HIV treatment should not be used to indicate the availability of HIV treatment in carceral settings within that country.

*HCV Testing:*

1. When extracting the availability of HCV testing in carceral settings within each country, extract both HCV antibody and RNA testing availability into the relevant fields.
2. In the case that HCV testing type is not specified within the record, extract availability data into the ‘Any/Unspecified HCV Testing’ fields.

*DAA Treatment:*

1. Only data for HCV treatment were included if it was clear the treatment was a direct acting antiviral therapy. Other treatments available for HCV were excluded.

*Tuberculosis Screening:*

1. For Tuberculosis Screening, extract the availability of services for ‘Screening for symptoms’, ‘Chest X-Ray’, ‘Tuberculin skin test’, ‘Blood Assay’, ‘Tuberculosis confirmation using culture’, ‘Tuberculosis confirmation using Gene Xpert‘ and ‘Tuberculosis confirmation using smear’ into the relevant fields.
2. If there is evidence of Tuberculosis Screening availability that do not align with these categories, extract into either ‘Other specified tuberculosis confirmation’ and note the type of testing, or ‘Unspecified screening’.

*Tuberculosis Treatment:*

1. Any type of tuberculosis treatment, which includes prescription of antibiotics (e.g., Isoniazid and Rifampicin) in carceral settings was be used to indicate availability of tuberculosis treatment in carceral settings within that country.

*HBV Testing:*

1. When extracting the availability of HBV testing in carceral settings within each country, extract only HBsAg testing for current infection.

*HBV Treatment & Vaccination:*

1. Any type of HBV treatment in carceral settings can be used to indicate availability of HBV treatment in carceral settings within that country.
2. Any type of HBV vaccination in carceral settings can be used to indicate availability of HBV vaccination in carceral settings within that country.

*Additional methodological decision rules:*

The availability of programmatic data was determined by whether countries reported information at the client level for each service. Countries were considered to have provided facility level data if they reported the specific number of facilities with a service or if they reported services in all facilities. In cases where services were reported in all facilities, WPB data informed the number of facilities for each service. For client-level data, this was based on the number of clients receiving OAT, the number of needles distributed, or the total number of tests or individuals tested for other interventions. Some countries also reported the number of facilities where these services were implemented.

When reporting on client level data for each intervention for Canada and the United States, we only included client level data availability if it was available for both regions in the country (i.e. state and federal), or if the outcome is only available in one state (i.e. TB available in Canada Federal but not Canada Provincial/Territorial) and the other region is not available (unknown does not count in this) than we would include this as availability of client level data.

For countries where no other data were available on the availability of OAT and NSPs in carceral settings, if provision of these services were listed as ‘not available’ at a community level in the global review by Colledge-Frisby et al.,^1^ we assumed and reported them as also not providing these services in carceral settings.

For Canada and the United States, national data were only available in the form of separate reporting for federal and state/provincial/territorial systems. Federal data alone are not representative of national service availability, as state and provincial/territorial systems account for a substantial proportion of the incarcerated population.^2^ Therefore, we collected and present data at both levels in **Appendix 6** to capture the full scope of intervention availability. However, for reporting in Table 1, we combined Canada provincial/territorial and federal and United States state and federal into Canada and the United States, respectively, to report at a regional level for both countries. For each intervention regionally presented in Table 1, we reported that these countries may have 0 in an availability column (for example in the column ‘Countries with UNKNOWN Availability in Carceral Settings (% of countries)’) but still maintain a percentage of the total prison population covered in the same column. As we may have data availability at the federal level but not at the state/provincial/territorial level. For example, for OAT in Canada, for Table 1 we reported this as available in ‘One or More’ carceral settings as Canada (provincial/territorial) reports availability in one or more prisons, however Canada (Federal) reports availability in ‘All facilities’. Thus, this enables us to calculate the percentage of the incarcerated population in North America, where each intervention is available at both a state/provincial/territorial and federal level. Additionally, for the United States, we excluded 663 100 people who are incarcerated in local jails^3^ from the total population calculations included in regional availability tables as we had insufficient data on interventions from this carceral category.

For England and Wales, the World Prison Brief only reports a combined incarcerated population^4^, therefore we reported a combined availability for HBV treatment as available in “One or More Carceral Setting” to enable calculation of the percentage of incarcerated population covered by each intervention. The data in **Appendix 6** reports that HBV treatment is available in all facilities in England, however HBV treatment availability is unknown in Wales. Therefore, we combined these availabilities to calculate the percentage of population covered by HBV treatment in carceral settings for Western Europe and globally using the combined England and Wales prison population.

**Appendix 5 References:**

1. Colledge-Frisby S, Ottaviano S, Webb P, et al. Global coverage of interventions to prevent and manage drug-related harms among people who inject drugs: a systematic review. . *Lancet Global Health* 2023.

2. Fair H, Walmsley R. World prison population list. 2024: ICPR; 2024.

3. World Prison Brief. United States of America. 2024. Available at: <https://www.prisonstudies.org/country/united-states-america>

4. World Prison Brief. United Kingdom: England & Wales. 2024. Available at:

<https://www.prisonstudies.org/country/united-kingdom-england-wales>

# Appendix 6: Country level availability of interventions and programmatic data for NSPs, OAT, HIV, HCV, HBV and tuberculosis in Carceral Settings

## *Appendix Table 6.1*: Country-level availability of interventions and programmatic data for any Opioid Agonist Treatment (OAT)

|  | | | | **Number of Carceral Sites with OAT** | | | | **Number of People accessing OAT in Carceral Settings** | | | |  |
| --- | --- | --- | --- | --- | --- | --- | --- | --- | --- | --- | --- | --- |
| **Country** | **Available** | **Year** | **Ref** | **Total sites*** | **N sites with OAT** | **Year** | **Ref** | **N People Accessing OAT in Carceral Settings** | **Timeframe** | **Year** | **Ref** | **Number of OAT recipients per 100 PWID** |
| **Eastern Europe** |  |  |  |  |  |  |  |  |  |  |  |  |
| Armenia | Yes | 2024 | 1,2 | 12 | ·· | ·· |  | ·· | ·· | ·· |  | ·· |
| Azerbaijan | No | 2024 | 1 | 53 | ·· | ·· |  | ·· | ·· | ·· |  | ·· |
| Belarus | No | 2024 | 1 | 67 | ·· | ·· |  | ·· | ·· | ·· |  | ·· |
| Bosnia & Herzegovina | Yes | 2015 | 3 | 13 | 6 | 2015 | 3 | ·· | ·· | ·· |  | ·· |
| Bulgaria | Yes | 2022 | 4,5 | 55 | ·· | ·· |  | ·· | ·· | ·· |  | ·· |
| Czechia | Yes | 2024 | 4,6 | 35 | ·· | ·· |  | 113 | Snapshot | 2021 | 6 | ·· |
| Estonia | Yes | 2024 | 4 | 3 | ·· | ·· |  | ·· | ·· | ·· |  | ·· |
| Georgia | Yes | 2013 | 7 | 14 | ·· | ·· |  | ·· | ·· | ·· |  | ·· |
| Hungary | No | 2024 | 1 | 30 | ·· | ·· |  | ·· | ·· | ·· |  | ·· |
| Latvia | Yes | 2022 | 4,8 | 9 | ·· | ·· |  | 40 | Snapshot | 2016 | 9 | 4 |
| Lithuania | All facilities | 2022 | 10 | 8 | 8 | 2022 | 10 | ·· | ·· | ·· |  | ·· |
| Republic of Moldova | Yes | 2022 | 11,12 | 17 | 13 | 2022 | 11 | 106 | Snapshot | 2022 | 11 | ·· |
| Poland | Yes | 2024 | 4 | 215 | ·· | ·· |  | 159 | Snapshot | 2017 | 13 | ·· |
| Romania | Yes | 2024 | 4,14 | 45 | ·· | ·· |  | 68 | Snapshot | 2017 | 14 | ·· |
| Russian Federation | No | 2023 | 15 | 872 | ·· | ·· |  | ·· | ·· | ·· |  | ·· |
| Slovakia | No | 2022 | 4 | 18 | ·· | ·· |  | ·· | ·· | ·· |  | ·· |
| Ukraine | Yes | 2024 | 1 | 110 | ·· | ·· |  | ·· | ·· | ·· |  | ·· |
| **Western Europe** |  |  |  |  |  |  |  |  |  |  |  |  |
| Albania | Yes | 2017 | 16 | 23 | ·· | ·· |  | ·· | ·· | ·· |  | ·· |
| Andorra | No | 2023 | 15 | 1 | ·· | ·· |  | ·· | ·· | ·· |  | ·· |
| Austria | All facilities | 2022 | 17 | 27 | 27 | 2022 | 17 | 862 | Snapshot | 2022 | 17 | 221 |
| Belgium | All facilities | 2022 | 4,18 | 35 | 35 | 2022 | 4 | 665 | Past year | 2016 | 9 | 26 |
| Croatia | All facilities | 2022 | 19,20 | 24 | 24 | 2022 | 19 | 835 | Past year | 2016 | 9 | 76 |
| Denmark | All facilities | 2022 | 4,21 | 54 | 54 | 2022 | 4 | 450 | Past year | 2015 | 9 | 26 |
| England** | All facilities | 2023 | 22,23 | 120 | 120 | 2023 | 22 | 16826 | Snapshot | 2023 | 22 | ·· |
| Finland | All facilities | 2022 | 4,21 | 26 | 26 | 2022 | 4 | 80 | Past year | 2016 | 9 | 5 |
| France | All facilities | 2022 | 4,24 | 187 | 187 | 2022 | 4 | 14900 | Past year | 2013 | 9 | 98 |
| Germany | All facilities | 2022 | 4 | 179 | 179 | 2022 | 4 | 1700 | Snapshot | 2011 | 9 | 10 |
| Greece | Yes | 2024 | 4 | 34 | ·· | ·· |  | 123 | Past year | 2016 | 9 | 3 |
| Greenland | No | 2023 | 15 | 6 | ·· | ·· |  | ·· | ·· | ·· |  | ·· |
| Iceland | Yes | 2024 | 25 | 5 | ·· | ·· |  | ·· | ·· | ·· |  | ·· |
| Ireland | Yes | 2022 | 4,26 | 12 | ·· | ·· |  | 465 | Past year | 2016 | 9 | 30 |
| Italy | Yes | 2022 | 4 | 206 | ·· | ·· |  | ·· | ·· | ·· |  | ·· |
| Liechtenstein | No | 2023 | 15 | 1 | ·· | ·· |  | ·· | ·· | ·· |  | ·· |
| Luxembourg | Yes | 2022 | 27,28 | 3 | ·· | ·· |  | 132 | Snapshot | 2021 | 28 | 96 |
| Malta | Yes | 2022 | 4 | 1 | ·· | ·· |  | ·· | ·· | ·· |  | ·· |
| Monaco | Unknown | ·· |  | 2 | ·· | ·· |  | ·· | ·· | ·· |  | ·· |
| Montenegro | Yes | 2010 | 2 | 3 | ·· | ·· |  | ·· | ·· | ·· |  | ·· |
| Netherlands | Yes | 2021 | 9 | 49 | ·· | ·· |  | 978 | Past year | 2014 | 9 | 99 |
| North Macedonia | All facilities | 2017 | 29 | 13 | 13 | 2017 | 29 | 239 | Snapshot | 2010 | 2 | ·· |
| Northern Ireland | All facilities | 2018 | 29 | 3 | 3 | 2018 | 29 | 30 | Past year | 2016 | 9 | 12 |
| Norway | All facilities | 2022 | 4 | 33 | 33 | 2022 | 4 | 271 | Snapshot | 2016 | 9 | 17 |
| Portugal | All facilities | 2022 | 21,30 | 49 | 49 | 2022 | 30 | 907 | Past year | 2016 | 9 | 48 |
| San Marino | Unknown | ·· |  | 1 | ·· | ·· |  | ·· | ·· | ·· |  | ·· |
| Scotland | All facilities | 2021 | 29,31 | 15 | 15 | 2017 | 29 | 2127 | Snapshot | 2021 | 31 | 129 |
| Serbia | All facilities | 2017 | 29,32 | 30 | 30 | 2017 | 29 | 143 | Snapshot | 2013 | 32 | ·· |
| Slovenia | Yes | 2022 | 4 | 7 | ·· | ·· |  | ·· | ·· | ·· |  | ·· |
| Spain | All facilities | 2022 | 4,33,34 | 82 | 82 | 2022 | 4 | 3404 | Snapshot | 2016 | 9 | 24 |
| Sweden | Yes | 2022 | 4 | 79 | ·· | ·· |  | ·· | ·· | ·· |  | ·· |
| Switzerland | Yes | 2013 | 35 | 88 | ·· | ·· |  | ·· | ·· | ·· |  | ·· |
| Wales** | All facilities | 2018 | 29 | 120 | 120 | 2018 | 29 | ·· | ·· | ·· |  | ·· |
| **East and South East Asia** |  |  |  |  |  |  |  |  |  |  |  |  |
| Brunei Darussalam | No | 2023 | 15 | 3 | ·· | ·· |  | ·· | ·· | ·· |  | ·· |
| Cambodia | Unknown | ·· |  | 29 | ·· | ·· |  | ·· | ·· | ·· |  | ·· |
| China | Unknown | ·· |  | 683 | ·· | ·· |  | ·· | ·· | ·· |  | ·· |
| Hong Kong† | Unknown | ·· |  | 24 | ·· | ·· |  | ·· | ·· | ·· |  | ·· |
| Indonesia | Yes | 2018 | 36,37 | 526 | 4 | 2018 | 36 | ·· | ·· | ·· |  | ·· |
| Japan | No | 2023 | 15 | 184 | ·· | ·· |  | ·· | ·· | ·· |  | ·· |
| Lao People's Democratic Republic | No | 2023 | 15 | 19 | ·· | ·· |  | ·· | ·· | ·· |  | ·· |
| Malaysia | Yes | 2014 | 38 | 43 | 18 | 2014 | 38 | ·· | ·· | ·· |  | ·· |
| Mongolia | No | 2023 | 15 | 50 | ·· | ·· |  | ·· | ·· | ·· |  | ·· |
| Myanmar | No | 2020 | 39 | 96 | ·· | ·· |  | ·· | ·· | ·· |  | ·· |
| Democratic People's Republic of Korea | No | 2023 | 15 | N/A | ·· | ·· |  | ·· | ·· | ·· |  | ·· |
| Philippines | No | 2023 | 15 | 440 | ·· | ·· |  | ·· | ·· | ·· |  | ·· |
| Singapore | No | 2023 | 15 | 13 | ·· | ·· |  | ·· | ·· | ·· |  | ·· |
| Republic of Korea | No | 2023 | 15 | 54 | ·· | ·· |  | ·· | ·· | ·· |  | ·· |
| Taiwan† | Unknown | ·· |  | 49 | ·· | ·· |  | ·· | ·· | ·· |  | ·· |
| Thailand | Unknown | ·· |  | 143 | ·· | ·· |  | ·· | ·· | ·· |  | ·· |
| Timor-Leste | No | 2023 | 15 | 3 | ·· | ·· |  | ·· | ·· | ·· |  | ·· |
| Viet Nam | Yes | 2020 | 40 | 54 | ·· | ·· |  | ·· | ·· | ·· |  | ·· |
| **South Asia** |  |  |  |  |  |  |  |  |  |  |  |  |
| Afghanistan | Unknown | ·· |  | 251 | ·· | ·· |  | ·· | ·· | ·· |  | ·· |
| Bangladesh | Unknown | ·· |  | 68 | ·· | ·· |  | ·· | ·· | ·· |  | ·· |
| Bhutan | No | 2023 | 15 | 7 | ·· | ·· |  | ·· | ·· | ·· |  | ·· |
| India | Yes | 2024 | 1 | 1330 | ·· | ·· |  | ·· | ·· | ·· |  | ·· |
| Islamic Republic of Iran | All facilities | 2024 | 1,41 | 253 | 253 | 2024 | 1 | 62743 | Past year | 2019 | 42 | 204 |
| Maldives | Unknown | ·· |  | 11 | ·· | ·· |  | ·· | ·· | ·· |  | ·· |
| Nepal | Unknown | ·· |  | 74 | ·· | ·· |  | ·· | ·· | ·· |  | ·· |
| Pakistan | No | 2023 | 15 | 116 | ·· | ·· |  | ·· | ·· | ·· |  | ·· |
| Sri Lanka | No | 2023 | 15 | 60 | ·· | ·· |  | ·· | ·· | ·· |  | ·· |
| **Central Asia** |  |  |  |  |  |  |  |  |  |  |  |  |
| Kazakhstan | No | 2024 | 1,43 | 80 | ·· | ·· |  | ·· | ·· | ·· |  | ·· |
| Kyrgyzstan | Yes | 2023 | 44,45 | 28 | 9 | 2023 | 44 | 208 | Snapshot | 2023 | 44 | 6 |
| Tajikistan | Yes | 2024 | 1 | 19 | ·· | ·· |  | 33 | Snapshot | 2024 | 1 | 6 |
| Turkmenistan | No | 2023 | 15 | 27 | ·· | ·· |  | ·· | ·· | ·· |  | ·· |
| Uzbekistan | No | 2014 | 46 | 54 | ·· | ·· |  | ·· | ·· | ·· |  | ·· |
| **Caribbean** |  |  |  |  |  |  |  |  |  |  |  |  |
| Antigua & Barbuda | No | 2023 | 15 | 1 | ·· | ·· |  | ·· | ·· | ·· |  | ·· |
| Bahamas | No | 2023 | 15 | 1 | ·· | ·· |  | ·· | ·· | ·· |  | ·· |
| Barbados | No | 2023 | 15 | 1 | ·· | ·· |  | ·· | ·· | ·· |  | ·· |
| Bermuda | No | 2023 | 15 | 4 | ·· | ·· |  | ·· | ·· | ·· |  | ·· |
| Cuba | No | 2023 | 15 | 200 | ·· | ·· |  | ·· | ·· | ·· |  | ·· |
| Dominica | No | 2023 | 15 | 1 | ·· | ·· |  | ·· | ·· | ·· |  | ·· |
| Dominican Republic | Unknown | ·· |  | 41 | ·· | ·· |  | ·· | ·· | ·· |  | ·· |
| Grenada | No | 2023 | 15 | 1 | ·· | ·· |  | ·· | ·· | ·· |  | ·· |
| Haiti | No | 2023 | 15 | 17 | ·· | ·· |  | ·· | ·· | ·· |  | ·· |
| Jamaica | No | 2023 | 15 | 11 | ·· | ·· |  | ·· | ·· | ·· |  | ·· |
| Commonwealth of Puerto Rico | Unknown | ·· |  | 34 | ·· | ·· |  | ·· | ·· | ·· |  | ·· |
| Saint Kitts & Nevis | No | 2023 | 15 | 2 | ·· | ·· |  | ·· | ·· | ·· |  | ·· |
| Saint Lucia | No | 2023 | 15 | 1 | ·· | ·· |  | ·· | ·· | ·· |  | ·· |
| Saint Vincent & the Grenadines | No | 2023 | 15 | 2 | ·· | ·· |  | ·· | ·· | ·· |  | ·· |
| Trinidad & Tobago | No | 2023 | 15 | 9 | ·· | ·· |  | ·· | ·· | ·· |  | ·· |
| **Latin America** |  |  |  |  |  |  |  |  |  |  |  |  |
| Argentina | Unknown | ·· |  | 338 | ·· | ·· |  | ·· | ·· | ·· |  | ·· |
| Belize | No | 2023 | 15 | 1 | ·· | ·· |  | ·· | ·· | ·· |  | ·· |
| Plurinational State of Bolivia | No | 2023 | 15 | 40 | ·· | ·· |  | ·· | ·· | ·· |  | ·· |
| Brazil | No | 2023 | 15 | 1386 | ·· | ·· |  | ·· | ·· | ·· |  | ·· |
| Chile | No | 2023 | 15 | 113 | ·· | ·· |  | ·· | ·· | ·· |  | ·· |
| Colombia | Yes | 2024 | 47 | 125 | ·· | ·· |  | ·· | ·· | ·· |  | ·· |
| Costa Rica | No | 2023 | 15 | 32 | ·· | ·· |  | ·· | ·· | ·· |  | ·· |
| Ecuador | No | 2023 | 15 | 52 | ·· | ·· |  | ·· | ·· | ·· |  | ·· |
| El Salvador | No | 2023 | 15 | 25 | ·· | ·· |  | ·· | ·· | ·· |  | ·· |
| Guatemala | No | 2023 | 15 | 21 | ·· | ·· |  | ·· | ·· | ·· |  | ·· |
| Guyana | No | 2023 | 15 | 7 | ·· | ·· |  | ·· | ·· | ·· |  | ·· |
| Honduras | No | 2023 | 15 | 26 | ·· | ·· |  | ·· | ·· | ·· |  | ·· |
| Mexico | No | 2024 | 48 | 279 | ·· | ·· |  | ·· | ·· | ·· |  | ·· |
| Nicaragua | No | 2023 | 15 | 9 | ·· | ·· |  | ·· | ·· | ·· |  | ·· |
| Panama | No | 2023 | 15 | 23 | ·· | ·· |  | ·· | ·· | ·· |  | ·· |
| Paraguay | No | 2023 | 15 | 18 | ·· | ·· |  | ·· | ·· | ·· |  | ·· |
| Peru | No | 2023 | 15 | 68 | ·· | ·· |  | ·· | ·· | ·· |  | ·· |
| Suriname | No | 2023 | 15 | 21 | ·· | ·· |  | ·· | ·· | ·· |  | ·· |
| Uruguay | No | 2023 | 15 | 27 | ·· | ·· |  | ·· | ·· | ·· |  | ·· |
| Bolivarian Republic of Venezuela | No | 2023 | 15 | 41 | ·· | ·· |  | ·· | ·· | ·· |  | ·· |
| **North America** |  |  |  |  |  |  |  |  |  |  |  |  |
| Canada (Federal) | All facilities | 2023 | 49 | 43 | 43 | 2023 | 49 | 3287 | Snapshot | 2023 | 49 | 87 |
| Canada (Provincial/Territorial) | Yes | 2020 | 50 | 173 | 49 | 2020 | 50 | ·· | ·· | ·· |  | ·· |
| United States of America (Federal) | Unknown | ·· |  | 102 | ·· | ·· |  | ·· | ·· | ·· |  | ·· |
| United States of America (State) | Yes | 2021 | 51,52 | 1190 | ·· | ·· |  | ·· | ·· | ·· |  | ·· |
| **Pacific Island States & Terr.** |  |  |  |  |  |  |  |  |  |  |  |  |
| American Samoa | No | 2023 | 15 | 1 | ·· | ·· |  | ·· | ·· | ·· |  | ·· |
| Federated States of Micronesia | No | 2023 | 15 | 4 | ·· | ·· |  | ·· | ·· | ·· |  | ·· |
| Fiji | No | 2023 | 15 | 15 | ·· | ·· |  | ·· | ·· | ·· |  | ·· |
| French Polynesia | No | 2023 | 15 | 2 | ·· | ·· |  | ·· | ·· | ·· |  | ·· |
| Guam | No | 2023 | 15 | 2 | ·· | ·· |  | ·· | ·· | ·· |  | ·· |
| Kiribati | No | 2023 | 15 | 4 | ·· | ·· |  | ·· | ·· | ·· |  | ·· |
| Marshall Islands | No | 2023 | 15 | 2 | ·· | ·· |  | ·· | ·· | ·· |  | ·· |
| Nauru | No | 2023 | 15 | 1 | ·· | ·· |  | ·· | ·· | ·· |  | ·· |
| New Caledonia | No | 2023 | 15 | 1 | ·· | ·· |  | ·· | ·· | ·· |  | ·· |
| Northern Mariana Islands | No | 2023 | 15 | 1 | ·· | ·· |  | ·· | ·· | ·· |  | ·· |
| Palau | No | 2023 | 15 | 1 | ·· | ·· |  | ·· | ·· | ·· |  | ·· |
| Papua New Guinea | No | 2023 | 15 | 18 | ·· | ·· |  | ·· | ·· | ·· |  | ·· |
| Samoa | No | 2023 | 15 | 3 | ·· | ·· |  | ·· | ·· | ·· |  | ·· |
| Solomon Islands | No | 2023 | 15 | 6 | ·· | ·· |  | ·· | ·· | ·· |  | ·· |
| Tonga | No | 2023 | 15 | 4 | ·· | ·· |  | ·· | ·· | ·· |  | ·· |
| Tuvalu | No | 2023 | 15 | 2 | ·· | ·· |  | ·· | ·· | ·· |  | ·· |
| Vanuatu | No | 2023 | 15 | 4 | ·· | ·· |  | ·· | ·· | ·· |  | ·· |
| **Australasia** |  |  |  |  |  |  |  |  |  |  |  |  |
| Australia | Yes | 2023 | 53 | 111 | ·· | ·· |  | 5841 | Snapshot | 2023 | 53 | 26 |
| New Zealand | All facilities | 2021 | 54 | 18 | 18 | 2021 | 54 | 55 | Past year | 2021 | 54 | ·· |
| **Sub Saharan Africa** |  |  |  |  |  |  |  |  |  |  |  |  |
| Angola | No | 2023 | 15 | 47 | ·· | ·· |  | ·· | ·· | ·· |  | ·· |
| Benin | No | 2023 | 15 | 11 | ·· | ·· |  | ·· | ·· | ·· |  | ·· |
| Botswana | No | 2023 | 15 | 23 | ·· | ·· |  | ·· | ·· | ·· |  | ·· |
| Burkina Faso | No | 2023 | 15 | 27 | ·· | ·· |  | ·· | ·· | ·· |  | ·· |
| Burundi | No | 2023 | 15 | 13 | ·· | ·· |  | ·· | ·· | ·· |  | ·· |
| Cameroon | No | 2023 | 15 | 76 | ·· | ·· |  | ·· | ·· | ·· |  | ·· |
| Cabo Verde | No | 2023 | 15 | 5 | ·· | ·· |  | ·· | ·· | ·· |  | ·· |
| Central African Republic | No | 2023 | 15 | 15 | ·· | ·· |  | ·· | ·· | ·· |  | ·· |
| Chad | No | 2023 | 15 | 41 | ·· | ·· |  | ·· | ·· | ·· |  | ·· |
| Comoros | No | 2023 | 15 | 3 | ·· | ·· |  | ·· | ·· | ·· |  | ·· |
| Côte d'Ivoire | Unknown | ·· |  | 34 | ·· | ·· |  | ·· | ·· | ·· |  | ·· |
| Democratic Republic of the Congo | No | 2023 | 15 | 142 | ·· | ·· |  | ·· | ·· | ·· |  | ·· |
| Djibouti | No | 2023 | 15 | 1 | ·· | ·· |  | ·· | ·· | ·· |  | ·· |
| Equatorial Guinea | No | 2023 | 15 | 15 | ·· | ·· |  | ·· | ·· | ·· |  | ·· |
| Eritrea | No | 2023 | 15 | N/A | ·· | ·· |  | ·· | ·· | ·· |  | ·· |
| Eswatini | No | 2023 | 15 | 12 | ·· | ·· |  | ·· | ·· | ·· |  | ·· |
| Ethiopia | No | 2023 | 15 | 126 | ·· | ·· |  | ·· | ·· | ·· |  | ·· |
| Gabon | No | 2023 | 15 | 9 | ·· | ·· |  | ·· | ·· | ·· |  | ·· |
| Gambia | No | 2023 | 15 | 3 | ·· | ·· |  | ·· | ·· | ·· |  | ·· |
| Ghana | Unknown | ·· |  | 46 | ·· | ·· |  | ·· | ·· | ·· |  | ·· |
| Guinea | No | 2023 | 15 | 31 | ·· | ·· |  | ·· | ·· | ·· |  | ·· |
| Guinea-Bissau | No | 2023 | 15 | 3 | ·· | ·· |  | ·· | ·· | ·· |  | ·· |
| Kenya | Yes | 2024 | 55 | 134 | 3 | 2024 | 55 | ·· | ·· | ·· |  | ·· |
| Lesotho | No | 2023 | 15 | 14 | ·· | ·· |  | ·· | ·· | ·· |  | ·· |
| Liberia | Unknown | ·· |  | 16 | ·· | ·· |  | ·· | ·· | ·· |  | ·· |
| Madagascar | Unknown | ·· |  | 82 | ·· | ·· |  | ·· | ·· | ·· |  | ·· |
| Malawi | No | 2023 | 15 | 30 | ·· | ·· |  | ·· | ·· | ·· |  | ·· |
| Mali | No | 2023 | 15 | 60 | ·· | ·· |  | ·· | ·· | ·· |  | ·· |
| Mauritania | No | 2023 | 15 | 23 | ·· | ·· |  | ·· | ·· | ·· |  | ·· |
| Mauritius | Yes | 2019 | 56,57 | 11 | 4 | 2019 | 56 | 355 | Snapshot | 2019 | 56 | ·· |
| Mozambique | Unknown | ·· |  | 157 | ·· | ·· |  | ·· | ·· | ·· |  | ·· |
| Namibia | No | 2023 | 15 | 13 | ·· | ·· |  | ·· | ·· | ·· |  | ·· |
| Niger | No | 2023 | 15 | 41 | ·· | ·· |  | ·· | ·· | ·· |  | ·· |
| Nigeria | No | 2023 | 15 | 240 | ·· | ·· |  | ·· | ·· | ·· |  | ·· |
| Congo | No | 2023 | 15 | 12 | ·· | ·· |  | ·· | ·· | ·· |  | ·· |
| Rwanda | No | 2023 | 15 | 13 | ·· | ·· |  | ·· | ·· | ·· |  | ·· |
| Sao Tome & Principe | No | 2023 | 15 | 1 | ·· | ·· |  | ·· | ·· | ·· |  | ·· |
| Senegal | Unknown | ·· |  | 37 | ·· | ·· |  | ·· | ·· | ·· |  | ·· |
| Seychelles | Unknown | ·· |  | 3 | ·· | ·· |  | ·· | ·· | ·· |  | ·· |
| Sierra Leone | No | 2023 | 15 | 21 | ·· | ·· |  | ·· | ·· | ·· |  | ·· |
| Somalia | No | 2023 | 15 | 14 | ·· | ·· |  | ·· | ·· | ·· |  | ·· |
| South Africa | No | 2020 | 58 | 235 | ·· | ·· |  | ·· | ·· | ·· |  | ·· |
| United Republic of Tanzania | Yes | 2024 | 59 | 126 | ·· | ·· |  | ·· | ·· | ·· |  | ·· |
| Togo | No | 2023 | 15 | 13 | ·· | ·· |  | ·· | ·· | ·· |  | ·· |
| Uganda | Unknown | ·· |  | 254 | ·· | ·· |  | ·· | ·· | ·· |  | ·· |
| Zambia | No | 2023 | 15 | 90 | ·· | ·· |  | ·· | ·· | ·· |  | ·· |
| Zimbabwe | No | 2023 | 15 | 72 | ·· | ·· |  | ·· | ·· | ·· |  | ·· |
| **Middle East & North Africa** |  |  |  |  |  |  |  |  |  |  |  |  |
| Algeria | No | 2023 | 15 | 162 | ·· | ·· |  | ·· | ·· | ·· |  | ·· |
| Bahrain | No | 2023 | 15 | 4 | ·· | ·· |  | ·· | ·· | ·· |  | ·· |
| Cyprus | Yes | 2022 | 4 | 1 | ·· | ·· |  | ·· | ·· | ·· |  | ·· |
| Egypt | No | 2024 | 60 | 78 | ·· | ·· |  | ·· | ·· | ·· |  | ·· |
| Iraq | No | 2023 | 15 | 55 | ·· | ·· |  | ·· | ·· | ·· |  | ·· |
| Israel | Yes | 2015 | 61 | 30 | 40 | 2015 | 61 | 600 | Past year | 2015 | 61 | ·· |
| Jordan | No | 2023 | 15 | 17 | ·· | ·· |  | ·· | ·· | ·· |  | ·· |
| Kuwait | No | 2023 | 15 | 3 | ·· | ·· |  | ·· | ·· | ·· |  | ·· |
| Lebanon | Unknown | ·· |  | 25 | ·· | ·· |  | ·· | ·· | ·· |  | ·· |
| Libya | No | 2023 | 15 | 26 | ·· | ·· |  | ·· | ·· | ·· |  | ·· |
| Morocco | Yes | 2021 | 62 | 75 | ·· | ·· |  | 100 | Snapshot | 2019 | 63 | ·· |
| Oman | No | 2023 | 15 | 3 | ·· | ·· |  | ·· | ·· | ·· |  | ·· |
| Occupied Palestinian territories | Unknown | ·· |  | N/A | ·· | ·· |  | ·· | ·· | ·· |  | ·· |
| Qatar | No | 2023 | 15 | 1 | ·· | ·· |  | ·· | ·· | ·· |  | ·· |
| Saudi Arabia | No | 2023 | 15 | 110 | ·· | ·· |  | ·· | ·· | ·· |  | ·· |
| South Sudan | No | 2023 | 15 | 80 | ·· | ·· |  | ·· | ·· | ·· |  | ·· |
| Sudan | No | 2023 | 15 | 125 | ·· | ·· |  | ·· | ·· | ·· |  | ·· |
| Syrian Arab Republic | No | 2023 | 15 | 35 | ·· | ·· |  | ·· | ·· | ·· |  | ·· |
| Tunisia | No | 2023 | 15 | 32 | ·· | ·· |  | ·· | ·· | ·· |  | ·· |
| Türkiye | Yes | 2022 | 4 | 403 | ·· | ·· |  | ·· | ·· | ·· |  | ·· |
| United Arab Emirates | No | 2023 | 15 | 21 | ·· | ·· |  | ·· | ·· | ·· |  | ·· |
| Yemen | No | 2023 | 15 | 43 | ·· | ·· |  | ·· | ·· | ·· |  | ·· |

**Notes:**

**Ref:** Reference; **OAT**: Opioid Agonist Treatment; **PWID**: people who inject drugs; **M**: Methadone; **B**: Buprenorphine

***Total sites:** Including prisons, jails or other carceral setting, Country level data that informed these regional and global estimates were sourced from the World Prison Brief, collated by the Institute for Crime and Justice Policy Research at Burbeck University. See: <https://www.prisonstudies.org/world-prison-brief-data>;

** The total number of sites for England and Wales was reported together in the World Prison Brief, therefore the total number of sites for both England and Wales is reported as 120.

† For reporting purposes, these countries or territories are reported separately due to differences in service provision.

·· Indicates there were no data to inform a country’s estimate.

**References Table 6.1**

1. Altice FL. OAT and NSP Coverage in Prisons in Eastern Europe and Central Asia. 2024.

2. Latypov A, Bidordinova A, Khachatrian A. Opioid substitution therapy in Eurasia: How to increase the access and improve the quality. IDPC Briefing Paper, 2012.

3. Network EHR. The Impact of Transition from Global Fund support to Governmental Funding on the Sustainability of Harm Reduction Programs, 2016.

4. European Union Drug Agency. EUDA Prison Data 2024. Lisbon; 2024.

5. Marotta PL, McCullagh CA. A cross-national analysis of the association between years of implementation of opioid substitution treatments and drug-related deaths in Europe from 1995 to 2013. *Eur J Epidemiol* 2018.

6. P Chomynova KG, Z Dvorakova, T Cernikova, B Orlikova, Z Rous, H Jarosikova, E Frankova, L Dekany, H Fidesova, J Vopravil. Zprava o nelegalnich drogach v Ceske republice, 2022.

7. Global A. Response Progress Report–GEORGIA Country Progress Report. *Reporting Period January* 2014.

8. Kurcalte O SM, Manson E, Karadzhan J, Krastiÿš I, & Zalans O. Analysis of trends in the use of addictive substances in prisons in Latvia in 2022": Final report, 2023.

9. Stöver H, Tarján A, Horváth G, Montanari L. The state of harm reduction in prisons in 30 European countries with a focus on people who inject drugs and infectious diseases. *Harm Reduct J* 2021.

10. Rasimaite B. DRID in prisons Lituhania. 2024.

11. EHRA. Republic of Moldova Reass4essment of the sustainability of the opioid agonist therapy programme within the context of transition from donor support to domestic funding, 2023.

12. Harm Reduction International. Availability, accessibility, acceptability and quality of harm reduction services in Moldovan prisons. London, 2021.

13. EMCDDA. Poland Country Drug Report, 2019.

14. EMCDDA. Romania Country Drug Report 2019: EMCDDA, 2019.

15. Colledge-Frisby S, Ottaviano S, Webb P, Grebely J, Wheeler A, Cunningham E. Global coverage of interventions to prevent and manage drug-related harms among people who inject drugs: a systematic review. Lancet Glob Health. 2023.

16. EMCDDA. Albania National Drug Report 2017: EMCDDA, 2017.

17. Reitox Focal Point Austria, Gesundheit Österreich GmbH Vienna. Prison workbook 2023 - Austria. Austria; 2023.

18. EMCDDA. Belgium Country Drug Report 2019: EMCDDA, 2019.

19. Andreić J-L. Prison Workbook 2023 - Croatia, 2023.

20. EMCDDA. Croatia Country Drug Report 2019: EMCDDA, 2019.

21. Merino P. Inventory of European social and health policies, measures and actions concerning drug users in prisons. *European Monitoring Centre for Drugs and Drug Addiction* 2005.

22. Disparities OfHIa. Alcohol and drug treatment in secure settings 2022 to 2023: report. 25 January 2024. https://www.gov.uk/government/statistics/substance-misuse-treatment-in-secure-settings-2022-to-2023/alcohol-and-drug-treatment-in-secure-settings-2022-to-2023-report--2.

23. Marsden J, Stillwell G, Jones H, et al. Does exposure to opioid substitution treatment in prison reduce the risk of death after release? A national prospective observational study in England. *Addict* 2017.

24. EMCDDA. France Country Drug Report 2019: EMCDDA, 2019.

25. Kattau T. Review of treatment services for substance use disorders in Iceland, 2024.

26. EMCDDA. Ireland Country Drug Report 2019, 2019.

27. Kugener T, Berndt N, Origer A, Debacker M. Prison workbook 2023 Luxembourg. 2023.

28. Berndt N SR, Kugener T, Origer A. National Drug Report 2022, 2022.

29. Bielen R, Stumo, S. R., Halford, R., Werling, K., Reic, T., Stover, H., Robaeys, G., & Lazarus, J. V. Harm reduction and viral hepatitis C in European prisons: A cross-sectional survey of 25 countries. *Harm Reduct J* 2018.

30. de Castro MV, Mendes SCC, Marques JPA, Santos AS, Duarte O. Prison Workbook 2023 - Portugal. 2023.

31. Scotland PH. Prison Health Information Dashboard, 2022.

32. Kilibarda B, Simić D, Baroš S, Brandić I. National Report on Drug Situation in Serbia, 2014, 2014.

33. EMCDDA. Spain Country Drug Report 2019: EMCDDA, 2019.

34. Drugs GDftNPo. Survey on Health and drug use in the inmate population in penitentiary institutions Summary Report, 2022.

35. Zurhold H, Stöver H. Provision of harm reduction and drug treatment services in custodial settings – Findings from the European ACCESS study. *Drugs Educ Prev Policy* 2016.

36. Gunawan R. Submission to the UN Working Group on Arbitrary Detention on detention in the context of drug policies, pursuant to Human Rights Council Resolution 42/22. 2020.

37. National AIDS Commission. National HIV and AIDS Strategy and Action Plan 2010 – 2014, 2010.

38. Ministry of Health Malaysia. Global AIDS response progress. Report Malaysia 2015: Ministry of Health Malaysia Kuala Lumpur, 2015.

39. Myanmar Ministry of Health and Sports. National Strategic Framework on Health and Drugs: Myanmar Ministry of Health and Sports, 2020.

40. The Global Fund. Technical Brief: Addresing HIV and TB in Prisons, Pre-Trial Detention and Other Closed Settings, 2020.

41. Ekhtiari H, Noroozi A, Farhoudian A, et al. The evolution of addiction treatment and harm reduction programs in Iran: a chaotic response or a synergistic diversity? *Addict* 2020; **115**(7): 1395-403.

42. UNAIDS. Country progress report - Iran, Global AIDS Monitoring 2020: UNAIDS.

43. Amanda R. Liberman, Yelena Rozental, Roman Ivasiy, et al. Exploration of the Multi-level Barriers to Scaling Up Methadone for HIV Prevention among People Who Inject Drugs in Kazakhstan. In: Kamenjas A, editor.; 2024.

44. Frontline AIDS. Harm reduction services for drug users in Kyrgyzstan. 2024; 2024.

45. Ivasiy R, Madden LM, Farnum SO, et al. Implementation opportunities for scaling up methadone maintenance treatment in Kyrgyzstan: Methadone dosage and retention on treatment over two years. *Drug Alcohol Depend Rep* 2022; **4**.

46. Latypov A. Drug Dependence in Central Asia: Challenges and Responses. 2014.

47. Hincapie C. In: Degenhardt L, editor.; 2024.

48. Borquez A. Coverage data in Mexico prisons. In: Thomas Santo Jr., editor.; 2024.

49. Canada Go. Opioid Agonist Treatment: September 2023. 2023 (accessed 23/11/23 2023).

50. Kronfli N, Dussault C, Bartlett S, et al. Disparities in hepatitis C care across Canadian provincial prisons: Implications for hepatitis C micro-elimination. *Can Liver J* 2021.

51. Dadiomov D, Trotzky-Sirr R, Shooshtari A, Qato DM. Changes in the availability of medications for opioid use disorder in prisons and jails in the United States during the COVID-19 pandemic. *Drug Alcohol Depend* 2022.

52. Thakrar AP, Alexander GC, Saloner B. Trends in Buprenorphine Use in US Jails and Prisons From 2016 to 2021. *JAMA Netw Open* 2021.

53. Welfare AIoHa. National Opioid Pharmacotherapy Statistics Annual Data collection, 2024.

54. Health Mo. Office of the Director of Mental Health and Addiction Services: Regulatory Report 1 July 2020 to 30 June 2021: Wellington: Ministry of Health, 2022.

55. Wambui S. Confirmation of OAT Availability in Kenyan Prisons. In: Thomas Santo Jr., editor.; 2024.

56. 2020 GAM. Country progress report - Mauritius, 2020.

57. Office NDSPMs. National Drug Observatory Report 2019, 2019.

58. Harm Reduction International. Harm reduction for women in prison, 2020.

59. Mjindo R. Confirmation of OAT Availability in Tanzanian Prisons. In: Thomas Santo Jr., editor.; 2024.

60. Sonnan T. OAT coverage in prisons in Egypt. In: Degenhardt L, editor.; 2024.

61. EMCDDA. Israel country overview. 2017.

62. Royaume du Maroc Ministère de la Santé et de la Protection Sociale. Rapport National SIDA 2022, 2021.

63. Ministry of Health Directory of Epidemiology and Diseases Control DoNMHO. National Program on Prevention and care of Substance Use and Addictions. 2021; 2021.

## *Appendix Table 6.2*: Country-level availability of interventions and programmatic data for Needle Syringe Programs

|  | | | | **Number of Carceral Sites with NSPs** | | | | **Number of People Accessing NSPs in Carceral Settings** | | | | **Number of Needle Syringes Distributed** | | | |
| --- | --- | --- | --- | --- | --- | --- | --- | --- | --- | --- | --- | --- | --- | --- | --- |
| **Country** | **Available** | **Year** | **Ref** | **Total sites*** | **N NSP Sites** | **Year** | **Ref** | **N People Accessing NSPs in Carceral Settings** | **Timeframe** | **Year** | **Ref** | **N** | **Timeframe** | **Year** | **Ref** |
| **Eastern Europe** |  |  |  |  |  |  |  |  |  |  |  |  |  |  |  |
| Armenia | No | 2024 | 1 | 12 | ·· | ·· | ·· | ·· | ·· | ·· |  | ·· | ·· | ·· |  |
| Azerbaijan | No | 2024 | 2 | 53 | ·· | ·· | ·· | ·· | ·· | ·· |  | ·· | ·· | ·· |  |
| Belarus | No | 2024 | 2 | 67 | ·· | ·· | ·· | ·· | ·· | ·· |  | ·· | ·· | ·· |  |
| Bosnia & Herzegovina | No | 2020 | 3 | 13 | ·· | ·· | ·· | ·· | ·· | ·· |  | ·· | ·· | ·· |  |
| Bulgaria | No | 2022 | 4 | 55 | ·· | ·· | ·· | ·· | ·· | ·· |  | ·· | ·· | ·· |  |
| Czechia | No | 2022 | 4 | 35 | ·· | ·· | ·· | ·· | ·· | ·· |  | ·· | ·· | ·· |  |
| Estonia | No | 2022 | 4 | 3 | ·· | ·· | ·· | ·· | ·· | ·· |  | ·· | ·· | ·· |  |
| Georgia | No | 2020 | 3 | 14 | ·· | ·· | ·· | ·· | ·· | ·· |  | ·· | ·· | ·· |  |
| Hungary | No | 2024 | 4 | 30 | ·· | ·· | ·· | ·· | ·· | ·· |  | ·· | ·· | ·· |  |
| Latvia | No | 2022 | 4 | 9 | ·· | ·· | ·· | ·· | ·· | ·· |  | ·· | ·· | ·· |  |
| Lithuania | No | 2022 | 5 | 8 | ·· | ·· | ·· | ·· | ·· | ·· |  | ·· | ·· | ·· |  |
| Republic of Moldova | Yes | 2024 | 6 | 17 | 15 | 2024 | ·· | ·· | ·· | ·· |  | 2750015 | Snapshot | 2024 | 6 |
| Poland | No | 2022 | 4 | 215 | ·· | ·· | ·· | ·· | ·· | ·· |  | ·· | ·· | ·· |  |
| Romania | No | 2024 | 6 | 45 | ·· | ·· | ·· | ·· | ·· | ·· |  | ·· | ·· | ·· |  |
| Russian Federation | No | 2010 | 7 | 872 | ·· | ·· | ·· | ·· | ·· | ·· |  | ·· | ·· | ·· |  |
| Slovakia | No | 2022 | 4 | 18 | ·· | ·· | ·· | ·· | ·· | ·· |  | ·· | ·· | ·· |  |
| Ukraine | Yes | 2024 | 6 | 110 | 2 | 2024 | 1248 | 1248 | Past -year | 2024 | 6 | ·· | ·· | ·· |  |
| **Western Europe** |  |  |  |  |  |  |  |  |  |  |  |  |  |  |  |
| Albania | No | 2020 | 3 | 23 | ·· | ·· | ·· | ·· | ·· | ·· |  | ·· | ·· | ·· |  |
| Andorra | No | 2023 | 8 | 1 | ·· | ·· | ·· | ·· | ·· | ·· |  | ·· | ·· | ·· |  |
| Austria | No | 2022 | 4 | 27 | ·· | ·· | ·· | ·· | ·· | ·· |  | ·· | ·· | ·· |  |
| Belgium | No | 2024 | 4 | 35 | ·· | ·· | ·· | ·· | ·· | ·· |  | ·· | ·· | ·· |  |
| Croatia | Unknown | ·· |  | ·· | ·· | ·· | ·· | ·· | ·· | ·· |  | ·· | ·· | ·· |  |
| Denmark | No | 2024 | 4 | 54 | ·· | ·· | ·· | ·· | ·· | ·· |  | ·· | ·· | ·· |  |
| England** | No | 2020 | 3 | 120 | ·· | ·· | ·· | ·· | ·· | ·· |  | ·· | ·· | ·· |  |
| Finland | No | 2024 | 4 | 26 | ·· | ·· | ·· | ·· | ·· | ·· |  | ·· | ·· | ·· |  |
| France | No | 2024 | 4 | 187 | ·· | ·· | ·· | ·· | ·· | ·· |  | ·· | ·· | ·· |  |
| Germany | Yes | 2024 | 6 | 179 | 1 | 2024 | ·· | ·· | ·· | ·· |  | 1483 | Snapshot | 2023 | 6 |
| Greece | No | 2024 | 4 | 34 | ·· | ·· | ·· | ·· | ·· | ·· |  | ·· | ·· | ·· |  |
| Greenland | No | 2023 | 8 | 6 | ·· | ·· | ·· | ·· | ·· | ·· |  | ·· | ·· | ·· |  |
| Iceland | Unknown | ·· |  | 5 | ·· | ·· | ·· | ·· | ·· | ·· |  | ·· | ·· | ·· |  |
| Ireland | No | 2022 | 4 | 12 | ·· | ·· | ·· | ·· | ·· | ·· |  | ·· | ·· | ·· |  |
| Italy | No | 2022 | 4 | 206 | ·· | ·· | ·· | ·· | ·· | ·· |  | ·· | ·· | ·· |  |
| Liechtenstein | No | 2023 | 8 | 1 | ·· | ·· | ·· | ·· | ·· | ·· |  | ·· | ·· | ·· |  |
| Luxembourg | All facilities | 2024 | 6 | 3 | All facilities | 2024 | 383 | 383 | 2005-2022 | 2022 | 6 | 16600 | 2005-2022 | 2022 | 6 |
| Malta | No | 2022 | 4 | 1 | ·· | ·· | ·· | ·· | ·· | ·· |  | ·· | ·· | ·· |  |
| Monaco | No | 2023 | 8 | 2 | ·· | ·· | ·· | ·· | ·· | ·· |  | ·· | ·· | ·· |  |
| Montenegro | Unknown | ·· |  | 3 | ·· | ·· | ·· | ·· | ·· | ·· |  | ·· | ·· | ·· |  |
| Netherlands | No | 2022 | 4 | 49 | ·· | ·· | ·· | ·· | ·· | ·· |  | ·· | ·· | ·· |  |
| North Macedonia | No | 2024 | 6 | 13 | ·· | ·· | ·· | ·· | ·· | ·· |  | ·· | ·· | ·· |  |
| Northern Ireland | No | 2020 | 3 | 3 | ·· | ·· | ·· | ·· | ·· | ·· |  | ·· | ·· | ·· |  |
| Norway | No | 2024 | 4 | 33 | ·· | ·· | ·· | ·· | ·· | ·· |  | ·· | ·· | ·· |  |
| Portugal | No | 2024 | 6 | 49 | ·· | ·· | ·· | ·· | ·· | ·· |  | ·· | ·· | ·· |  |
| San Marino | All facilities | 2020 | 3 | 1 | All facilities | 2020 | ·· | ·· | ·· | ·· |  | ·· | ·· | ·· |  |
| Scotland | No | 2020 | 3 | 15 | ·· | ·· | ·· | ·· | ·· | ·· |  | ·· | ·· | ·· |  |
| Serbia | No | 2017 | 9 | 30 | ·· | ·· | ·· | ·· | ·· | ·· |  | ·· | ·· | ·· |  |
| Slovenia | No | 2022 | 4 | 7 | ·· | ·· | ·· | ·· | ·· | ·· |  | ·· | ·· | ·· |  |
| Spain | All facilities | 2024 | 6 | 82 | All facilities | 2024 | ·· | ·· | ·· | ·· |  | 914 | Snapshot | 2023 | 6 |
| Sweden | No | 2024 | 4 | 79 | ·· | ·· | ·· | ·· | ·· | ·· |  | ·· | ·· | ·· |  |
| Switzerland | Yes | 2024 | 6 | 88 | 15 | 2024 | ·· | ·· | ·· | ·· |  | ·· | ·· | ·· |  |
| Wales** | No | 2020 | 3 | 120 | ·· | ·· | ·· | ·· | ·· | ·· |  | ·· | ·· | ·· |  |
| **East and South East Asia** |  |  |  |  |  |  |  |  |  |  |  |  |  |  |  |
| Brunei Darussalam | No | 2023 | 8 | 3 | ·· | ·· | ·· | ·· | ·· | ·· |  | ·· | ·· | ·· |  |
| Cambodia | Unknown | ·· |  | 29 | ·· | ·· | ·· | ·· | ·· | ·· |  | ·· | ·· | ·· |  |
| China | Unknown | ·· |  | 683 | ·· | ·· | ·· | ·· | ·· | ·· |  | ·· | ·· | ·· |  |
| Hong Kong† | No | 2023 | 8 | 24 | ·· | ·· | ·· | ·· | ·· | ·· |  | ·· | ·· | ·· |  |
| Indonesia | No | 2018 | 10 | 526 | ·· | ·· | ·· | ·· | ·· | ·· |  | ·· | ·· | ·· |  |
| Japan | No | 2023 | 8 | 184 | ·· | ·· | ·· | ·· | ·· | ·· |  | ·· | ·· | ·· |  |
| Lao People's Democratic Republic | Unknown | ·· |  | 19 | ·· | ·· | ·· | ·· | ·· | ·· |  | ·· | ·· | ·· |  |
| Malaysia | No | 2023 | 11 | 43 | ·· | ·· | ·· | ·· | ·· | ·· |  | ·· | ·· | ·· |  |
| Mongolia | Unknown | ·· |  | 50 | ·· | ·· | ·· | ·· | ·· | ·· |  | ·· | ·· | ·· |  |
| Myanmar | Unknown | ·· |  | 96 | ·· | ·· | ·· | ·· | ·· | ·· |  | ·· | ·· | ·· |  |
| Democratic People's Republic of Korea | No | 2023 | 8 | N/A | ·· | ·· | ·· | ·· | ·· | ·· |  | ·· | ·· | ·· |  |
| Philippines | No | 2023 | 8 | 440 | ·· | ·· | ·· | ·· | ·· | ·· |  | ·· | ·· | ·· |  |
| Singapore | No | 2023 | 8 | 13 | ·· | ·· | ·· | ·· | ·· | ·· |  | ·· | ·· | ·· |  |
| Republic of Korea | No | 2023 | 8 | 54 | ·· | ·· | ·· | ·· | ·· | ·· |  | ·· | ·· | ·· |  |
| Taiwan† | Unknown | ·· |  | 49 | ·· | ·· | ·· | ·· | ·· | ·· |  | ·· | ·· | ·· |  |
| Thailand | Unknown | ·· |  | 143 | ·· | ·· | ·· | ·· | ·· | ·· |  | ·· | ·· | ·· |  |
| Timor-Leste | No | 2023 | 8 | 3 | ·· | ·· | ·· | ·· | ·· | ·· |  | ·· | ·· | ·· |  |
| Viet Nam | Unknown | ·· |  | 54 | ·· | ·· | ·· | ·· | ·· | ·· |  | ·· | ·· | ·· |  |
| **South Asia** |  |  |  |  |  |  |  |  |  |  |  |  |  |  |  |
| Afghanistan | No | 2024 | 12 | 251 | ·· | ·· | ·· | ·· | ·· | ·· |  | ·· | ·· | ·· |  |
| Bangladesh | Unknown | ·· |  | 68 | ·· | ·· | ·· | ·· | ·· | ·· |  | ·· | ·· | ·· |  |
| Bhutan | No | 2023 | 8 | 7 | ·· | ·· | ·· | ·· | ·· | ·· |  | ·· | ·· | ·· |  |
| India | No | 2024 | 2 | 1330 | ·· | ·· | ·· | ·· | ·· | ·· |  | ·· | ·· | ·· |  |
| Islamic Republic of Iran | No | 2024 | 6 | 253 | ·· | ·· | ·· | ·· | ·· | ·· |  | ·· | ·· | ·· |  |
| Maldives | No | 2023 | 8 | 11 | ·· | ·· | ·· | ·· | ·· | ·· |  | ·· | ·· | ·· |  |
| Nepal | Unknown | ·· |  | 74 | ·· | ·· | ·· | ·· | ·· | ·· |  | ·· | ·· | ·· |  |
| Pakistan | Unknown | ·· |  | 116 | ·· | ·· | ·· | ·· | ·· | ·· |  | ·· | ·· | ·· |  |
| Sri Lanka | No | 2023 | 8 | 60 | ·· | ·· | ·· | ·· | ·· | ·· |  | ·· | ·· | ·· |  |
| **Central Asia** |  |  |  |  |  |  |  |  |  |  |  |  |  |  |  |
| Kazakhstan | No | 2024 | 2 | 80 | ·· | ·· | ·· | ·· | ·· | ·· |  | ·· | ·· | ·· |  |
| Kyrgyzstan | Yes | 2024 | 6 | 28 | 9 | 2024 | 1150 | 1150 | Snapshot | 2020 | 6 | ·· | ·· | ·· |  |
| Tajikistan | Yes | 2024 | 6 | 19 | 3 | 2024 | ·· | ·· | ·· | ·· |  | 23018 | Snapshot | 2019 | 11 |
| Turkmenistan | No | 2023 | 8 | 27 | ·· | ·· | ·· | ·· | ·· | ·· |  | ·· | ·· | ·· |  |
| Uzbekistan | No | 2010 | 13 | 54 | ·· | ·· | ·· | ·· | ·· | ·· |  | ·· | ·· | ·· |  |
| **Caribbean** |  |  |  |  |  |  |  |  |  |  |  |  |  |  |  |
| Antigua & Barbuda | No | 2023 | 8 | 1 | ·· | ·· | ·· | ·· | ·· | ·· |  | ·· | ·· | ·· |  |
| Bahamas | No | 2023 | 8 | 1 | ·· | ·· | ·· | ·· | ·· | ·· |  | ·· | ·· | ·· |  |
| Barbados | No | 2023 | 8 | 1 | ·· | ·· | ·· | ·· | ·· | ·· |  | ·· | ·· | ·· |  |
| Bermuda | No | 2023 | 8 | 4 | ·· | ·· | ·· | ·· | ·· | ·· |  | ·· | ·· | ·· |  |
| Cuba | No | 2023 | 8 | 200 | ·· | ·· | ·· | ·· | ·· | ·· |  | ·· | ·· | ·· |  |
| Dominica | No | 2023 | 8 | 1 | ·· | ·· | ·· | ·· | ·· | ·· |  | ·· | ·· | ·· |  |
| Dominican Republic | Unknown | ·· |  | 41 | ·· | ·· | ·· | ·· | ·· | ·· |  | ·· | ·· | ·· |  |
| Grenada | No | 2023 | 8 | 1 | ·· | ·· | ·· | ·· | ·· | ·· |  | ·· | ·· | ·· |  |
| Haiti | No | 2023 | 8 | 17 | ·· | ·· | ·· | ·· | ·· | ·· |  | ·· | ·· | ·· |  |
| Jamaica | No | 2023 | 8 | 11 | ·· | ·· | ·· | ·· | ·· | ·· |  | ·· | ·· | ·· |  |
| Commonwealth of Puerto Rico | Unknown | ·· |  | 34 | ·· | ·· | ·· | ·· | ·· | ·· |  | ·· | ·· | ·· |  |
| Saint Kitts & Nevis | No | 2023 | 8 | 2 | ·· | ·· | ·· | ·· | ·· | ·· |  | ·· | ·· | ·· |  |
| Saint Lucia | No | 2023 | 8 | 1 | ·· | ·· | ·· | ·· | ·· | ·· |  | ·· | ·· | ·· |  |
| Saint Vincent & the Grenadines | No | 2023 | 8 | 2 | ·· | ·· | ·· | ·· | ·· | ·· |  | ·· | ·· | ·· |  |
| Trinidad & Tobago | No | 2023 | 8 | 9 | ·· | ·· | ·· | ·· | ·· | ·· |  | ·· | ·· | ·· |  |
| **Latin America** |  |  |  |  |  |  |  |  |  |  |  |  |  |  |  |
| Argentina | No | 2023 | 8 | 338 | ·· | ·· | ·· | ·· | ·· | ·· |  | ·· | ·· | ·· |  |
| Belize | No | 2023 | 8 | 1 | ·· | ·· | ·· | ·· | ·· | ·· |  | ·· | ·· | ·· |  |
| Plurinational State of Bolivia | No | 2023 | 8 | 40 | ·· | ·· | ·· | ·· | ·· | ·· |  | ·· | ·· | ·· |  |
| Brazil | No | 2023 | 8 | 1386 | ·· | ·· | ·· | ·· | ·· | ·· |  | ·· | ·· | ·· |  |
| Chile | No | 2023 | 8 | 113 | ·· | ·· | ·· | ·· | ·· | ·· |  | ·· | ·· | ·· |  |
| Colombia | No | 2024 | 14 | 125 | ·· | ·· | ·· | ·· | ·· | ·· |  | ·· | ·· | ·· |  |
| Costa Rica | No | 2023 | 8 | 32 | ·· | ·· | ·· | ·· | ·· | ·· |  | ·· | ·· | ·· |  |
| Ecuador | No | 2023 | 8 | 52 | ·· | ·· | ·· | ·· | ·· | ·· |  | ·· | ·· | ·· |  |
| El Salvador | No | 2023 | 8 | 25 | ·· | ·· | ·· | ·· | ·· | ·· |  | ·· | ·· | ·· |  |
| Guatemala | No | 2023 | 8 | 21 | ·· | ·· | ·· | ·· | ·· | ·· |  | ·· | ·· | ·· |  |
| Guyana | No | 2023 | 8 | 7 | ·· | ·· | ·· | ·· | ·· | ·· |  | ·· | ·· | ·· |  |
| Honduras | No | 2023 | 8 | 26 | ·· | ·· | ·· | ·· | ·· | ·· |  | ·· | ·· | ·· |  |
| Mexico | No | 2024 | 15 | 279 | ·· | ·· | ·· | ·· | ·· | ·· |  | ·· | ·· | ·· |  |
| Nicaragua | No | 2023 | 8 | 9 | ·· | ·· | ·· | ·· | ·· | ·· |  | ·· | ·· | ·· |  |
| Panama | No | 2023 | 8 | 23 | ·· | ·· | ·· | ·· | ·· | ·· |  | ·· | ·· | ·· |  |
| Paraguay | No | 2023 | 8 | 18 | ·· | ·· | ·· | ·· | ·· | ·· |  | ·· | ·· | ·· |  |
| Peru | No | 2023 | 8 | 68 | ·· | ·· | ·· | ·· | ·· | ·· |  | ·· | ·· | ·· |  |
| Suriname | No | 2023 | 8 | 21 | ·· | ·· | ·· | ·· | ·· | ·· |  | ·· | ·· | ·· |  |
| Uruguay | No | 2023 | 8 | 27 | ·· | ·· | ·· | ·· | ·· | ·· |  | ·· | ·· | ·· |  |
| Bolivarian Republic of Venezuela | No | 2023 | 8 | 41 | ·· | ·· | ·· | ·· | ·· | ·· |  | ·· | ·· | ·· |  |
| **North America** |  |  |  |  |  |  |  |  |  |  |  |  |  |  |  |
| Canada (Federal) | Yes | 2024 | 6 | 43 | 9 | 2024 | ·· | ·· | ·· | ·· |  | ·· | ·· | ·· |  |
| Canada (Provincial/Territorial) | No | 2024 | 6 | 173 | ·· | ·· | ·· | ·· | ·· | ·· |  | ·· | ·· | ·· |  |
| United States of America (Federal) | No | 2021 | 16 | 102 | ·· | ·· | ·· | ·· | ·· | ·· |  | ·· | ·· | ·· |  |
| United States of America (State) | No | 2021 | 16 | 1190 | ·· | ·· | ·· | ·· | ·· | ·· |  | ·· | ·· | ·· |  |
| **Pacific Island States & Terr.** |  |  |  |  |  |  |  |  |  |  |  |  |  |  |  |
| American Samoa | No | 2023 | 8 | 1 | ·· | ·· | ·· | ·· | ·· | ·· |  | ·· | ·· | ·· |  |
| Federated States of Micronesia | No | 2023 | 8 | 4 | ·· | ·· | ·· | ·· | ·· | ·· |  | ·· | ·· | ·· |  |
| Fiji | No | 2023 | 8 | 15 | ·· | ·· | ·· | ·· | ·· | ·· |  | ·· | ·· | ·· |  |
| French Polynesia | No | 2023 | 8 | 2 | ·· | ·· | ·· | ·· | ·· | ·· |  | ·· | ·· | ·· |  |
| Guam | No | 2023 | 8 | 2 | ·· | ·· | ·· | ·· | ·· | ·· |  | ·· | ·· | ·· |  |
| Kiribati | No | 2023 | 8 | 4 | ·· | ·· | ·· | ·· | ·· | ·· |  | ·· | ·· | ·· |  |
| Marshall Islands | No | 2023 | 8 | 2 | ·· | ·· | ·· | ·· | ·· | ·· |  | ·· | ·· | ·· |  |
| Nauru | No | 2023 | 8 | 1 | ·· | ·· | ·· | ·· | ·· | ·· |  | ·· | ·· | ·· |  |
| New Caledonia | No | 2023 | 8 | 1 | ·· | ·· | ·· | ·· | ·· | ·· |  | ·· | ·· | ·· |  |
| Northern Mariana Islands | No | 2023 | 8 | 1 | ·· | ·· | ·· | ·· | ·· | ·· |  | ·· | ·· | ·· |  |
| Palau | No | 2023 | 8 | 1 | ·· | ·· | ·· | ·· | ·· | ·· |  | ·· | ·· | ·· |  |
| Papua New Guinea | No | 2023 | 8 | 18 | ·· | ·· | ·· | ·· | ·· | ·· |  | ·· | ·· | ·· |  |
| Samoa | No | 2023 | 8 | 3 | ·· | ·· | ·· | ·· | ·· | ·· |  | ·· | ·· | ·· |  |
| Solomon Islands | No | 2023 | 8 | 6 | ·· | ·· | ·· | ·· | ·· | ·· |  | ·· | ·· | ·· |  |
| Tonga | No | 2023 | 8 | 4 | ·· | ·· | ·· | ·· | ·· | ·· |  | ·· | ·· | ·· |  |
| Tuvalu | No | 2023 | 8 | 2 | ·· | ·· | ·· | ·· | ·· | ·· |  | ·· | ·· | ·· |  |
| Vanuatu | No | 2023 | 8 | 4 | ·· | ·· | ·· | ·· | ·· | ·· |  | ·· | ·· | ·· |  |
| **Australasia** |  |  |  |  |  |  |  |  |  |  |  |  |  |  |  |
| Australia | No | 2020 | 17 | 111 | ·· | ·· | ·· | ·· | ·· | ·· |  | ·· | ·· | ·· |  |
| New Zealand | Unknown | ·· |  | 18 | ·· | ·· | ·· | ·· | ·· | ·· |  | ·· | ·· | ·· |  |
| **Sub Saharan Africa** |  |  |  |  |  |  |  |  |  |  |  |  |  |  |  |
| Angola | No | 2023 | 8 | 47 | ·· | ·· | ·· | ·· | ·· | ·· |  | ·· | ·· | ·· |  |
| Benin | Unknown | ·· |  | 11 | ·· | ·· | ·· | ·· | ·· | ·· |  | ·· | ·· | ·· |  |
| Botswana | No | 2023 | 8 | 23 | ·· | ·· | ·· | ·· | ·· | ·· |  | ·· | ·· | ·· |  |
| Burkina Faso | No | 2023 | 8 | 27 | ·· | ·· | ·· | ·· | ·· | ·· |  | ·· | ·· | ·· |  |
| Burundi | No | 2023 | 8 | 13 | ·· | ·· | ·· | ·· | ·· | ·· |  | ·· | ·· | ·· |  |
| Cameroon | No | 2023 | 8 | 76 | ·· | ·· | ·· | ·· | ·· | ·· |  | ·· | ·· | ·· |  |
| Cabo Verde | No | 2023 | 8 | 5 | ·· | ·· | ·· | ·· | ·· | ·· |  | ·· | ·· | ·· |  |
| Central African Republic | No | 2023 | 8 | 15 | ·· | ·· | ·· | ·· | ·· | ·· |  | ·· | ·· | ·· |  |
| Chad | No | 2023 | 8 | 41 | ·· | ·· | ·· | ·· | ·· | ·· |  | ·· | ·· | ·· |  |
| Comoros | No | 2023 | 8 | 3 | ·· | ·· | ·· | ·· | ·· | ·· |  | ·· | ·· | ·· |  |
| Côte d'Ivoire | No | 2023 | 8 | 34 | ·· | ·· | ·· | ·· | ·· | ·· |  | ·· | ·· | ·· |  |
| Democratic Republic of the Congo | No | 2023 | 8 | 142 | ·· | ·· | ·· | ·· | ·· | ·· |  | ·· | ·· | ·· |  |
| Djibouti | No | 2023 | 8 | 1 | ·· | ·· | ·· | ·· | ·· | ·· |  | ·· | ·· | ·· |  |
| Equatorial Guinea | No | 2023 | 8 | 15 | ·· | ·· | ·· | ·· | ·· | ·· |  | ·· | ·· | ·· |  |
| Eritrea | No | 2023 | 8 | N/A | ·· | ·· | ·· | ·· | ·· | ·· |  | ·· | ·· | ·· |  |
| Eswatini | No | 2023 | 8 | 12 | ·· | ·· | ·· | ·· | ·· | ·· |  | ·· | ·· | ·· |  |
| Ethiopia | No | 2023 | 8 | 126 | ·· | ·· | ·· | ·· | ·· | ·· |  | ·· | ·· | ·· |  |
| Gabon | No | 2023 | 8 | 9 | ·· | ·· | ·· | ·· | ·· | ·· |  | ·· | ·· | ·· |  |
| Gambia | No | 2023 | 8 | 3 | ·· | ·· | ·· | ·· | ·· | ·· |  | ·· | ·· | ·· |  |
| Ghana | No | 2023 | 8 | 46 | ·· | ·· | ·· | ·· | ·· | ·· |  | ·· | ·· | ·· |  |
| Guinea | No | 2023 | 8 | 31 | ·· | ·· | ·· | ·· | ·· | ·· |  | ·· | ·· | ·· |  |
| Guinea-Bissau | No | 2023 | 8 | 3 | ·· | ·· | ·· | ·· | ·· | ·· |  | ·· | ·· | ·· |  |
| Kenya | No | 2012 | 18 | 134 | ·· | ·· | ·· | ·· | ·· | ·· |  | ·· | ·· | ·· |  |
| Lesotho | No | 2023 | 8 | 14 | ·· | ·· | ·· | ·· | ·· | ·· |  | ·· | ·· | ·· |  |
| Liberia | No | 2023 | 8 | 16 | ·· | ·· | ·· | ·· | ·· | ·· |  | ·· | ·· | ·· |  |
| Madagascar | Unknown | ·· |  | 82 | ·· | ·· | ·· | ·· | ·· | ·· |  | ·· | ·· | ·· |  |
| Malawi | No | 2023 | 8 | 30 | ·· | ·· | ·· | ·· | ·· | ·· |  | ·· | ·· | ·· |  |
| Mali | Unknown | ·· |  | 60 | ·· | ·· | ·· | ·· | ·· | ·· |  | ·· | ·· | ·· |  |
| Mauritania | No | 2023 | 8 | 23 | ·· | ·· | ·· | ·· | ·· | ·· |  | ·· | ·· | ·· |  |
| Mauritius | Unknown | ·· |  | 11 | ·· | ·· | ·· | ·· | ·· | ·· |  | ·· | ·· | ·· |  |
| Mozambique | Unknown | ·· |  | 157 | ·· | ·· | ·· | ·· | ·· | ·· |  | ·· | ·· | ·· |  |
| Namibia | No | 2023 | 8 | 13 | ·· | ·· | ·· | ·· | ·· | ·· |  | ·· | ·· | ·· |  |
| Niger | No | 2023 | 8 | 41 | ·· | ·· | ·· | ·· | ·· | ·· |  | ·· | ·· | ·· |  |
| Nigeria | Unknown | ·· |  | 240 | ·· | ·· | ·· | ·· | ·· | ·· |  | ·· | ·· | ·· |  |
| Congo | No | 2023 | 8 | 12 | ·· | ·· | ·· | ·· | ·· | ·· |  | ·· | ·· | ·· |  |
| Rwanda | No | 2023 | 8 | 13 | ·· | ·· | ·· | ·· | ·· | ·· |  | ·· | ·· | ·· |  |
| Sao Tome & Principe | No | 2023 | 8 | 1 | ·· | ·· | ·· | ·· | ·· | ·· |  | ·· | ·· | ·· |  |
| Senegal | Unknown | ·· |  | 37 | ·· | ·· | ·· | ·· | ·· | ·· |  | ·· | ·· | ·· |  |
| Seychelles | Unknown | ·· |  | 3 | ·· | ·· | ·· | ·· | ·· | ·· |  | ·· | ·· | ·· |  |
| Sierra Leone | Unknown | ·· |  | 21 | ·· | ·· | ·· | ·· | ·· | ·· |  | ·· | ·· | ·· |  |
| Somalia | No | 2023 | 8 | 14 | ·· | ·· | ·· | ·· | ·· | ·· |  | ·· | ·· | ·· |  |
| South Africa | No | 2020 | 19 | 235 | ·· | ·· | ·· | ·· | ·· | ·· |  | ·· | ·· | ·· |  |
| United Republic of Tanzania | Unknown | ·· |  | 126 | ·· | ·· | ·· | ·· | ·· | ·· |  | ·· | ·· | ·· |  |
| Togo | No | 2023 | 8 | 13 | ·· | ·· | ·· | ·· | ·· | ·· |  | ·· | ·· | ·· |  |
| Uganda | Unknown | ·· |  | 254 | ·· | ·· | ·· | ·· | ·· | ·· |  | ·· | ·· | ·· |  |
| Zambia | No | 2023 | 8 | 90 | ·· | ·· | ·· | ·· | ·· | ·· |  | ·· | ·· | ·· |  |
| Zimbabwe | No | 2023 | 8 | 72 | ·· | ·· | ·· | ·· | ·· | ·· |  | ·· | ·· | ·· |  |
| **Middle East & North Africa** |  |  |  |  |  |  |  |  |  |  |  |  |  |  |  |
| Algeria | Unknown | ·· |  | 162 | ·· | ·· | ·· | ·· | ·· | ·· |  | ·· | ·· | ·· |  |
| Bahrain | No | 2023 | 8 | 4 | ·· | ·· | ·· | ·· | ·· | ·· |  | ·· | ·· | ·· |  |
| Cyprus | No | 2023 | 4 | 1 | ·· | ·· | ·· | ·· | ·· | ·· |  | ·· | ·· | ·· |  |
| Egypt | Unknown | ·· |  | 78 | ·· | ·· | ·· | ·· | ·· | ·· |  | ·· | ·· | ·· |  |
| Iraq | No | 2023 | 8 | 55 | ·· | ·· | ·· | ·· | ·· | ·· |  | ·· | ·· | ·· |  |
| Israel | Unknown | ·· |  | 30 | ·· | ·· | ·· | ·· | ·· | ·· |  | ·· | ·· | ·· |  |
| Jordan | Unknown | ·· |  | 17 | ·· | ·· | ·· | ·· | ·· | ·· |  | ·· | ·· | ·· |  |
| Kuwait | No | 2023 | 8 | 3 | ·· | ·· | ·· | ·· | ·· | ·· |  | ·· | ·· | ·· |  |
| Lebanon | Unknown | ·· |  | 25 | ·· | ·· | ·· | ·· | ·· | ·· |  | ·· | ·· | ·· |  |
| Libya | No | 2023 | 8 | 26 | ·· | ·· | ·· | ·· | ·· | ·· |  | ·· | ·· | ·· |  |
| Morocco | No | 2021 | 20 | 75 | ·· | ·· | ·· | ·· | ·· | ·· |  | ·· | ·· | ·· |  |
| Oman | No | 2023 | 8 | 3 | ·· | ·· | ·· | ·· | ·· | ·· |  | ·· | ·· | ·· |  |
| Occupied Palestinian territories | Unknown | ·· |  | N/A | ·· | ·· | ·· | ·· | ·· | ·· |  | ·· | ·· | ·· |  |
| Qatar | No | 2023 | 8 | 1 | ·· | ·· | ·· | ·· | ·· | ·· |  | ·· | ·· | ·· |  |
| Saudi Arabia | No | 2023 | 8 | 110 | ·· | ·· | ·· | ·· | ·· | ·· |  | ·· | ·· | ·· |  |
| South Sudan | No | 2023 | 8 | 80 | ·· | ·· | ·· | ·· | ·· | ·· |  | ·· | ·· | ·· |  |
| Sudan | No | 2023 | 8 | 125 | ·· | ·· | ·· | ·· | ·· | ·· |  | ·· | ·· | ·· |  |
| Syrian Arab Republic | No | 2023 | 8 | 35 | ·· | ·· | ·· | ·· | ·· | ·· |  | ·· | ·· | ·· |  |
| Tunisia | Unknown | ·· |  | 32 | ·· | ·· | ·· | ·· | ·· | ·· |  | ·· | ·· | ·· |  |
| Türkiye | No | 2022 | 4 | 403 | ·· | ·· | ·· | ·· | ·· | ·· |  | ·· | ·· | ·· |  |
| United Arab Emirates | No | 2023 | 8 | 21 | ·· | ·· | ·· | ·· | ·· | ·· |  | ·· | ·· | ·· |  |
| Yemen | No | 2023 | 8 | 43 | ·· | ·· | ·· | ·· | ·· | ·· |  | ·· | ·· | ·· |  |

**Notes:**

**Ref:** Reference; **NSP:** Needle and Syringe Program.

There have been reports of some NS distribution in Montpellier, but these do not seem to have delivered or sanctioned by the French government. See: <https://www.who.int/europe/news/item/18-07-2023-the-independence-of-doctors-is-essential--the-work-of-a-montpellier-prison-towards-eliminating-hepatitis-c>

***Total sites:** Including prisons, jails or other carceral setting, Country level data that informed these regional and global estimates were sourced from the World Prison Brief, collated by the Institute for Crime and Justice Policy Research at Burbeck University. See: <https://www.prisonstudies.org/world-prison-brief-data>**;**

** The total number of sites for England and Wales was reported together in the World Prison Brief, therefore the total number of sites for both England and Wales is reported as 120.

† For reporting purposes, these countries or territories are reported separately due to differences in service provision.

·· Indicates there were no data to inform a country’s estimate.

**References for Table 6.2**

1. Otiashvili D. NSPs in prisons in Armenia. In: Degenhardt L, editor.; 2024.

2. Altice FL. OAT and NSP Coverage in Prisons in Eastern Europe and Central Asia. 2024.

3. World Health Organization. Status report on prison health in the WHO European Region 2022. Copenhagen, 2023.

4. European Union Drug Agency. EUDA Prison Data 2024. Lisbon; 2024.

5. Rasimaite B. DRID in prisons Lituhania. 2024.

6. Kronfli N, Bromberg DJ, Wolff H, Montanari L, Vasyliev S, Altice FL. Improving implementation of needle and syringe programmes to expand, scale up, and sustain evidence-based prevention interventions for HIV and hepatitis C in prisons. *Lancet Pub Health* 2025; **10**(1): e63-e70.

7. Group WB. Preventing HIV infection in Russia : best practices in harm reduction programs (English), 2010.

8. Colledge-Frisby S, Ottaviano S, Webb P, Grebely J, Wheeler A, Cunningham E. Global coverage of interventions to prevent and manage drug-related harms among people who inject drugs: a systematic review. Lancet Glob Health. 2023.

9. Bielen R, Stumo, S. R., Halford, R., Werling, K., Reic, T., Stover, H., Robaeys, G., & Lazarus, J. V. Harm reduction and viral hepatitis C in European prisons: A cross-sectional survey of 25 countries. *Harm Reduct J* 2018.

10. Gunawan R. Submission to the UN Working Group on Arbitrary Detention on detention in the context of drug policies, pursuant to Human Rights Council Resolution 42/22. 2020.

11. Suan MAM, Zulkifli AM, Ani NH, et al. Feasibility of a prison-based test-and-treat model for enhancing hepatitis C care in Kedah, Malaysia. *BMC Public Health* 2025; **25**(1): 1152.

12. Afarin R. NSPs in prisons in Afghanistan. 2024.

13. United Nations Office on Drugs and Crime. Accessibility of HIV Prevention, Treatment and Care Services for People who Use Drugs and Incarcerated People in Azerbaijan, Kazakhstan, Kyrgyzstan, Tajikistan, Turkmenistan, and Uzbekistan: Legislative and Policy Recommentations for Reform. Ashgabat, 2010.

14. Hincapie C. In: Degenhardt L, editor.; 2024.

15. Borquez A. Coverage data in Mexico prisons. In: Thomas Santo Jr., editor.; 2024.

16. Armstrong-Mensah E, Dada D, Rupasinghe R, Whately H. Injecting substance use in prisons in the United States: a case for needle exchange programs. *Am J Drug Alcohol Abuse* 2021.

17. Simpson PL, Gardoll B, White L, Butler T. HIV policies in Australian prisons: a structured review assessing compliance with international guidelines. *Lancet Reg Health West Pac* 2023; **41**.

18. Ministry of Health Kenya. MARPs Surveillance Report. *Ministry of Health, National AIDS & STI Control Programme-NASCOP* 2012.

19. Harm Reduction International. Harm reduction for women in prison, 2020.

20. Royaume du Maroc Ministère de la Santé et de la Protection Sociale. Rapport National SIDA 2022, 2021.

## *Appendix Table 6.3*: Country-level availability of interventions and programmatic data for HIV Testing

|  | | | | **Number of Carceral Sites with HIV Testing** | | | | **Number of People Accessing HIV Testing in Carceral Settings** | | | | **Number of HIV Tests Distributed** | | | |
| --- | --- | --- | --- | --- | --- | --- | --- | --- | --- | --- | --- | --- | --- | --- | --- |
| **Country** | **Available** | **Year** | **Ref** | **Total sites*** | **N Sites with HIV Testing** | **Year** | **Ref** | **N People Accessing HIV Testing in Carceral settings** | **Timeframe** | **Year** | **Ref** | **N** | **Timeframe** | **Year** | **Ref** |
| **Eastern Europe** |  |  |  |  |  |  |  |  |  |  |  |  |  |  |  |
| Armenia | All facilities | 2020 | 1 | 12 | 12 | 2020 | 1 | ·· | ·· | ·· |  | ·· | ·· | ·· |  |
| Azerbaijan | Yes | 2013 | 2 | 53 | ·· | ·· |  | 5304 | Snapshot | 2013 | 2 | ·· | ·· | ·· |  |
| Belarus | Unknown | ·· |  | 67 | ·· | ·· |  | ·· | ·· | ·· |  | ·· | ·· | ·· |  |
| Bosnia & Herzegovina | Yes | 2020 | 1 | 13 | ·· | ·· |  | ·· | ·· | ·· |  | ·· | ·· | ·· |  |
| Bulgaria | All facilities | 2022 | 3 | 55 | 55 | 2022 | 3 | ·· | ·· | ·· |  | 499 | Snapshot | 2022 | 4 |
| Czechia | All facilities | 2020 | 1 | 35 | 35 | 2020 | 1 | ·· | ·· | ·· |  | ·· | ·· | ·· |  |
| Estonia | All facilities | 2022 | 3 | 3 | 3 | 2022 | 3 | ·· | ·· | ·· |  | 3112 | Census | 2015 | 5 |
| Georgia | All facilities | 2020 | 1 | 14 | 14 | 2020 | 1 | ·· | ·· | ·· |  | ·· | ·· | ·· |  |
| Hungary | Yes | 2022 | 3 | 30 | 15 | 2022 | 3 | ·· | ·· | ·· |  | ·· | ·· | ·· |  |
| Latvia | All facilities | 2022 | 3 | 9 | 9 | 2022 | 3 | ·· | ·· | ·· |  | 3035 | Snapshot | 2022 | 3 |
| Lithuania | Yes | 2022 | 3 | 8 | 2 | 2022 | 3 | ·· | ·· | ·· |  | 6824 | Past year | 2022 | 6 |
| Republic of Moldova | All facilities | 2021 | 7 | 17 | 17 | 2021 | 7 | ·· | ·· | ·· |  | 4169 | Past year | 2020 | 7 |
| Poland | All facilities | 2022 | 3 | 215 | 215 | 2022 | 3 | ·· | ·· | ·· |  | 4008 | Past year | 2012 | 8 |
| Romania | Yes | 2022 | 3 | 45 | ·· | ·· |  | ·· | ·· | ·· |  | ·· | ·· | ·· |  |
| Russian Federation | Yes | 2022 | 9 | 872 | ·· | ·· |  | ·· | ·· | ·· |  | ·· | ·· | ·· |  |
| Slovakia | All facilities | 2022 | 3 | 18 | 18 | 2022 | 3 | ·· | ·· | ·· |  | ·· | ·· | ·· |  |
| Ukraine | All facilities | 2020 | 1 | 110 | 110 | 2020 | 1 | 57200 | Snapshot | 2020 | 10 | ·· | ·· | ·· |  |
| **Western Europe** |  |  |  |  |  |  |  |  |  |  |  |  |  |  |  |
| Albania | Yes | 2020 | 1 | 23 | ·· | ·· |  | ·· | ·· | ·· |  | ·· | ·· | ·· |  |
| Andorra | Unknown | ·· |  | 1 | ·· | ·· |  | ·· | ·· | ·· |  | ·· | ·· | ·· |  |
| Austria | All facilities | 2022 | 3 | 27 | 27 | 2022 | 3 | ·· | ·· | ·· |  | ·· | ·· | ·· |  |
| Belgium | All facilities | 2020 | 1 | 35 | 35 | 2020 | 1 | ·· | ·· | ·· |  | ·· | ·· | ·· |  |
| Croatia | All facilities | 2022 | 11 | 24 | 24 | 2022 | 11 | ·· | ·· | ·· |  | ·· | ·· | ·· |  |
| Denmark | Yes | 2022 | 3 | 54 | ·· | ·· |  | ·· | ·· | ·· |  | ·· | ·· | ·· |  |
| England** | All facilities | 2023 | 12 | 120 | 120 | 2023 | 12 | ·· | ·· | ·· |  | ·· | ·· | ·· |  |
| Finland | All facilities | 2022 | 3 | 26 | 26 | 2022 | 3 | ·· | ·· | ·· |  | ·· | ·· | ·· |  |
| France | All facilities | 2022 | 3 | 187 | 187 | 2022 | 3 | ·· | ·· | ·· |  | ·· | ·· | ·· |  |
| Germany | Yes | 2022 | 3 | 179 | ·· | ·· |  | ·· | ·· | ·· |  | ·· | ·· | ·· |  |
| Greece | All facilities | 2020 | 1 | 34 | 34 | 2020 | 1 | ·· | ·· | ·· |  | ·· | ·· | ·· |  |
| Greenland | Unknown | ·· |  | 6 | ·· | ·· |  | ·· | ·· | ·· |  | ·· | ·· | ·· |  |
| Iceland | Unknown | ·· |  | 5 | ·· | ·· |  | ·· | ·· | ·· |  | ·· | ·· | ·· |  |
| Ireland | All facilities | 2022 | 3 | 12 | 12 | 2022 | 3 | ·· | ·· | ·· |  | ·· | ·· | ·· |  |
| Italy | All facilities | 2020 | 1 | 206 | 206 | 2020 | 1 | 6630 | Snapshot | 2013 | 13 | ·· | ·· | ·· |  |
| Liechtenstein | Unknown | ·· |  | 1 | ·· | ·· |  | ·· | ·· | ·· |  | ·· | ·· | ·· |  |
| Luxembourg | Yes | 2022 | 14 | 3 | 1 | 2022 | 14 | ·· | ·· | ·· |  | ·· | ·· | ·· |  |
| Malta | All facilities | 2020 | 1 | 1 | 1 | 2020 | 1 | ·· | ·· | ·· |  | ·· | ·· | ·· |  |
| Monaco | All facilities | 2020 | 1 | 2 | 2 | 2020 | 1 | ·· | ·· | ·· |  | ·· | ·· | ·· |  |
| Montenegro | Unknown | ·· |  | 3 | ·· | ·· |  | ·· | ·· | ·· |  | ·· | ·· | ·· |  |
| Netherlands | Yes | 2022 | 3 | 49 | ·· | ·· |  | ·· | ·· | ·· |  | ·· | ·· | ·· |  |
| North Macedonia | Unknown | ·· |  | 13 | ·· | ·· |  | ·· | ·· | ·· |  | ·· | ·· | ·· |  |
| Northern Ireland | All facilities | 2020 | 1 | 3 | 3 | 2020 | 1 | ·· | ·· | ·· |  | ·· | ·· | ·· |  |
| Norway | Yes | 2022 | 3 | 33 | ·· | ·· |  | ·· | ·· | ·· |  | ·· | ·· | ·· |  |
| Portugal | All facilities | 2022 | 15 | 49 | 49 | 2022 | 15 | ·· | ·· | ·· |  | ·· | ·· | ·· |  |
| San Marino | All facilities | 2020 | 1 | 1 | 1 | 2020 | 1 | ·· | ·· | ·· |  | ·· | ·· | ·· |  |
| Scotland | All facilities | 2022 | 16 | 15 | 15 | 2022 | 16 | ·· | ·· | ·· |  | ·· | ·· | ·· |  |
| Serbia | All facilities | 2013 | 17 | 30 | 30 | 2013 | 17 | ·· | ·· | ·· |  | ·· | ·· | ·· |  |
| Slovenia | All facilities | 2022 | 3 | 7 | 7 | 2022 | 3 | ·· | ·· | ·· |  | ·· | ·· | ·· |  |
| Spain | All facilities | 2022 | 3 | 82 | 82 | 2022 | 3 | ·· | ·· | ·· |  | ·· | ·· | ·· |  |
| Sweden | All facilities | 2022 | 3 | 79 | 79 | 2022 | 3 | ·· | ·· | ·· |  | ·· | ·· | ·· |  |
| Switzerland | Yes | 2020 | 1 | 88 | ·· | ·· |  | ·· | ·· | ·· |  | ·· | ·· | ·· |  |
| Wales** | All facilities | 2020 | 18 | 120 | 120 | 2020 | 18 | ·· | ·· | ·· |  | ·· | ·· | ·· |  |
| **East and South East Asia** |  |  |  |  |  |  |  |  |  |  |  |  |  |  |  |
| Brunei Darussalam | Unknown | ·· |  | 3 | ·· | ·· |  | ·· | ·· | ·· |  | ·· | ·· | ·· |  |
| Cambodia | Yes | 2013 | 19 | 29 | ·· | ·· |  | ·· | ·· | ·· |  | ·· | ·· | ·· |  |
| China | Unknown | ·· |  | 683 | ·· | ·· |  | ·· | ·· | ·· |  | ·· | ·· | ·· |  |
| Hong Kong† | Yes | 2021 | 20 | 24 | ·· | ·· |  | ·· | ·· | ·· |  | 1169 | Past year | 2021 | 20 |
| Indonesia | Yes | 2012 | 21 | 526 | 78 | 2012 | 21 | 4285 | Snapshot | 2009 | 22 | ·· | ·· | ·· |  |
| Japan | Unknown | ·· |  | 184 | ·· | ·· |  | ·· | ·· | ·· |  | ·· | ·· | ·· |  |
| Lao People's Democratic Republic | Unknown | ·· |  | 19 | ·· | ·· |  | ·· | ·· | ·· |  | ·· | ·· | ·· |  |
| Malaysia | All facilities | 2014 | 23 | 43 | 43 | 2014 | 23 | ·· | ·· | ·· |  | ·· | ·· | ·· |  |
| Mongolia | Unknown | ·· |  | 50 | ·· | ·· |  | ·· | ·· | ·· |  | ·· | ·· | ·· |  |
| Myanmar | Yes | 2019 | 24 | 96 | 45 | 2019 | 24 | ·· | ·· | ·· |  | ·· | ·· | ·· |  |
| Democratic People's Republic of Korea | Unknown | ·· |  | N/A | ·· | ·· |  | ·· | ·· | ·· |  | ·· | ·· | ·· |  |
| Philippines | Unknown | ·· |  | 440 | ·· | ·· |  | ·· | ·· | ·· |  | ·· | ·· | ·· |  |
| Singapore | Unknown | ·· |  | 13 | ·· | ·· |  | ·· | ·· | ·· |  | ·· | ·· | ·· |  |
| Republic of Korea | Unknown | ·· |  | 54 | ·· | ·· |  | ·· | ·· | ·· |  | ·· | ·· | ·· |  |
| Taiwan† | Unknown | ·· |  | 49 | ·· | ·· |  | ·· | ·· | ·· |  | ·· | ·· | ·· |  |
| Thailand | Yes | 2016 | 25 | 143 | ·· | ·· |  | 18624 | Past year | 2016 | 25 | ·· | ·· | ·· |  |
| Timor-Leste | Unknown | ·· |  | 3 | ·· | ·· |  | ·· | ·· | ·· |  | ·· | ·· | ·· |  |
| Viet Nam | Yes | 2020 | 26 | 54 | ·· | ·· |  | ·· | ·· | ·· |  | ·· | ·· | ·· |  |
| **South Asia** |  |  |  |  |  |  |  |  |  |  |  |  |  |  |  |
| Afghanistan | Unknown | ·· |  | 251 | ·· | ·· |  | ·· | ·· | ·· |  | ·· | ·· | ·· |  |
| Bangladesh | Unknown | ·· |  | 68 | ·· | ·· |  | ·· | ·· | ·· |  | ·· | ·· | ·· |  |
| Bhutan | Unknown | ·· |  | 7 | ·· | ·· |  | ·· | ·· | ·· |  | ·· | ·· | ·· |  |
| India | Yes | 2023 | 27 | 1330 | ·· | ·· |  | 1245209 | Past year | 2023 | 27 | ·· | ·· | ·· |  |
| Islamic Republic of Iran | Yes | 2019 | 28 | 253 | ·· | ·· |  | ·· | ·· | ·· |  | ·· | ·· | ·· |  |
| Maldives | Yes | 2017 | 29 | 11 | ·· | ·· |  | ·· | ·· | ·· |  | ·· | ·· | ·· |  |
| Nepal | Yes | 2022 | 30 | 74 | ·· | ·· |  | ·· | ·· | ·· |  | ·· | ·· | ·· |  |
| Pakistan | No | 2010 | 31 | 116 | ·· | ·· |  | ·· | ·· | ·· |  | ·· | ·· | ·· |  |
| Sri Lanka | Yes | 2014 | 32 | 60 | ·· | ·· |  | 13803 | Snapshot | 2014 | 32 | ·· | ·· | ·· |  |
| **Central Asia** |  |  |  |  |  |  |  |  |  |  |  |  |  |  |  |
| Kazakhstan | Yes | 2011 | 33 | 80 | ·· | ·· |  | ·· | ·· | ·· |  | ·· | ·· | ·· |  |
| Kyrgyzstan | Yes | 2014 | 34 | 28 | ·· | ·· |  | ·· | ·· | ·· |  | ·· | ·· | ·· |  |
| Tajikistan | Yes | 2019 | 35 | 19 | ·· | ·· |  | 7111 | Past year | 2019 | 35 | ·· | ·· | ·· |  |
| Turkmenistan | Yes | 2010 | 33 | 27 | ·· | ·· |  | ·· | ·· | ·· |  | ·· | ·· | ·· |  |
| Uzbekistan | Yes | 2010 | 33 | 54 | ·· | ·· |  | ·· | ·· | ·· |  | ·· | ·· | ·· |  |
| **Caribbean** |  |  |  |  |  |  |  |  |  |  |  |  |  |  |  |
| Antigua & Barbuda | Unknown | ·· |  | 1 | ·· | ·· |  | ·· | ·· | ·· |  | ·· | ·· | ·· |  |
| Bahamas | Unknown | ·· |  | 1 | ·· | ·· |  | ·· | ·· | ·· |  | ·· | ·· | ·· |  |
| Barbados | Unknown | ·· |  | 1 | ·· | ·· |  | ·· | ·· | ·· |  | ·· | ·· | ·· |  |
| Bermuda | Unknown | ·· |  | 4 | ·· | ·· |  | ·· | ·· | ·· |  | ·· | ·· | ·· |  |
| Cuba | Unknown | ·· |  | 200 | ·· | ·· |  | ·· | ·· | ·· |  | ·· | ·· | ·· |  |
| Dominica | Unknown | ·· |  | 1 | ·· | ·· |  | ·· | ·· | ·· |  | ·· | ·· | ·· |  |
| Dominican Republic | Unknown | ·· |  | 41 | ·· | ·· |  | ·· | ·· | ·· |  | ·· | ·· | ·· |  |
| Grenada | Unknown | ·· |  | 1 | ·· | ·· |  | ·· | ·· | ·· |  | ·· | ·· | ·· |  |
| Haiti | Unknown | ·· |  | 17 | ·· | ·· |  | ·· | ·· | ·· |  | ·· | ·· | ·· |  |
| Jamaica | Unknown*** | 2006 | 36 | 11 | ·· | ·· |  | ·· | ·· | ·· |  | ·· | ·· | ·· |  |
| Commonwealth of Puerto Rico | Unknown | ·· |  | 34 | ·· | ·· |  | ·· | ·· | ·· |  | ·· | ·· | ·· |  |
| Saint Kitts & Nevis | Unknown | ·· |  | 2 | ·· | ·· |  | ·· | ·· | ·· |  | ·· | ·· | ·· |  |
| Saint Lucia | Unknown | ·· |  | 1 | ·· | ·· |  | ·· | ·· | ·· |  | ·· | ·· | ·· |  |
| Saint Vincent & the Grenadines | Unknown | ·· |  | 2 | ·· | ·· |  | ·· | ·· | ·· |  | ·· | ·· | ·· |  |
| Trinidad & Tobago | Unknown | ·· |  | 9 | ·· | ·· |  | ·· | ·· | ·· |  | ·· | ·· | ·· |  |
| **Latin America** |  |  |  |  |  |  |  |  |  |  |  |  |  |  |  |
| Argentina | Yes | 2011 | 37 | 338 | ·· | ·· |  | ·· | ·· | ·· |  | ·· | ·· | ·· |  |
| Belize | Unknown | ·· |  | 1 | ·· | ·· |  | ·· | ·· | ·· |  | ·· | ·· | ·· |  |
| Plurinational State of Bolivia | Unknown | ·· |  | 40 | ·· | ·· |  | ·· | ·· | ·· |  | ·· | ·· | ·· |  |
| Brazil | All facilities | 2003 | 38 | 1386 | 1386 | 2003 | 38 | ·· | ·· | ·· |  | ·· | ·· | ·· |  |
| Chile | Unknown | ·· |  | 113 | ·· | ·· |  | ·· | ·· | ·· |  | ·· | ·· | ·· |  |
| Colombia | Yes | 2024 | 39 | 125 | ·· | ·· |  | ·· | ·· | ·· |  | ·· | ·· | ·· |  |
| Costa Rica | Unknown | ·· |  | 32 | ·· | ·· |  | ·· | ·· | ·· |  | ·· | ·· | ·· |  |
| Ecuador | Yes | 6806 |  | 52 | ·· | ·· |  | ·· | ·· | ·· |  | ·· | ·· | ·· |  |
| El Salvador | Unknown | ·· |  | 25 | ·· | ·· |  | ·· | ·· | ·· |  | ·· | ·· | ·· |  |
| Guatemala | Yes | 2021 | 40 | 21 | ·· | ·· |  | ·· | ·· | ·· |  | ·· | ·· | ·· |  |
| Guyana | Unknown | ·· |  | 7 | ·· | ·· |  | ·· | ·· | ·· |  | ·· | ·· | ·· |  |
| Honduras | Yes | 2025 | 41 | 26 | ·· | ·· |  | ·· | ·· | ·· |  | ·· | ·· | ·· |  |
| Mexico | Yes | 2024 | 42 | 279 | ·· | ·· |  | ·· | ·· | ·· |  | ·· | ·· | ·· |  |
| Nicaragua | Unknown | ·· |  | 9 | ·· | ·· |  | ·· | ·· | ·· |  | ·· | ·· | ·· |  |
| Panama | Unknown | ·· |  | 23 | ·· | ·· |  | ·· | ·· | ·· |  | ·· | ·· | ·· |  |
| Paraguay | Unknown | ·· |  | 18 | ·· | ·· |  | ·· | ·· | ·· |  | ·· | ·· | ·· |  |
| Peru | Unknown | ·· |  | 68 | ·· | ·· |  | ·· | ·· | ·· |  | ·· | ·· | ·· |  |
| Suriname | Unknown | ·· |  | 21 | ·· | ·· |  | ·· | ·· | ·· |  | ·· | ·· | ·· |  |
| Uruguay | Yes | 2016 | 43 | 27 | ·· | ·· |  | ·· | ·· | ·· |  | ·· | ·· | ·· |  |
| Bolivarian Republic of Venezuela | Unknown | ·· |  | 41 | ·· | ·· |  | ·· | ·· | ·· |  | ·· | ·· | ·· |  |
| **North America** |  |  |  |  |  |  |  |  |  |  |  |  |  |  |  |
| Canada (Federal) | All facilities | 2008 | 44 | 43 | 43 | 2008 | 44 | 6011 | Snapshot | 2008 | 44 | ·· | ·· | ·· |  |
| Canada (Provincial/ Territorial) | Unknown | ·· |  | 173 | ·· | ·· |  | ·· | ·· | ·· |  | ·· | ·· | ·· |  |
| United States of America (Federal) | All facilities | 2021 | 45 | 102 | 102 | 2021 | 45 | ·· | ·· | ·· |  | ·· | ·· | ·· |  |
| United States of America (State) | All facilities | 2021 | 45 | 1190 | 1190 | 2021 | 45 | ·· | ·· | ·· |  | ·· | ·· | ·· |  |
| **Pacific Island States & Terr.** |  |  |  |  |  |  |  |  |  |  |  |  |  |  |  |
| American Samoa | Unknown | ·· |  | 1 | ·· | ·· |  | ·· | ·· | ·· |  | ·· | ·· | ·· |  |
| Federated States of Micronesia | Unknown | ·· |  | 4 | ·· | ·· |  | ·· | ·· | ·· |  | ·· | ·· | ·· |  |
| Fiji | Unknown | ·· |  | 15 | ·· | ·· |  | ·· | ·· | ·· |  | ·· | ·· | ·· |  |
| French Polynesia | Unknown | ·· |  | 2 | ·· | ·· |  | ·· | ·· | ·· |  | ·· | ·· | ·· |  |
| Guam | Unknown | ·· |  | 2 | ·· | ·· |  | ·· | ·· | ·· |  | ·· | ·· | ·· |  |
| Kiribati | Unknown | ·· |  | 4 | ·· | ·· |  | ·· | ·· | ·· |  | ·· | ·· | ·· |  |
| Marshall Islands | Unknown | ·· |  | 2 | ·· | ·· |  | ·· | ·· | ·· |  | ·· | ·· | ·· |  |
| Nauru | Unknown | ·· |  | 1 | ·· | ·· |  | ·· | ·· | ·· |  | ·· | ·· | ·· |  |
| New Caledonia | Unknown | ·· |  | 1 | ·· | ·· |  | ·· | ·· | ·· |  | ·· | ·· | ·· |  |
| Northern Mariana Islands | Unknown | ·· |  | 1 | ·· | ·· |  | ·· | ·· | ·· |  | ·· | ·· | ·· |  |
| Palau | Unknown | ·· |  | 1 | ·· | ·· |  | ·· | ·· | ·· |  | ·· | ·· | ·· |  |
| Papua New Guinea | Unknown | ·· |  | 18 | ·· | ·· |  | ·· | ·· | ·· |  | ·· | ·· | ·· |  |
| Samoa | Unknown | ·· |  | 3 | ·· | ·· |  | ·· | ·· | ·· |  | ·· | ·· | ·· |  |
| Solomon Islands | Unknown | ·· |  | 6 | ·· | ·· |  | ·· | ·· | ·· |  | ·· | ·· | ·· |  |
| Tonga | Unknown | ·· |  | 4 | ·· | ·· |  | ·· | ·· | ·· |  | ·· | ·· | ·· |  |
| Tuvalu | Unknown | ·· |  | 2 | ·· | ·· |  | ·· | ·· | ·· |  | ·· | ·· | ·· |  |
| Vanuatu | Unknown | ·· |  | 4 | ·· | ·· |  | ·· | ·· | ·· |  | ·· | ·· | ·· |  |
| **Australasia** |  |  |  |  |  |  |  |  |  |  |  |  |  |  |  |
| Australia | Yes | 2020 | 46 | 111 | ·· | ·· |  | ·· | ·· | ·· |  | ·· | ·· | ·· |  |
| New Zealand | Unknown | ·· |  | 18 | ·· | ·· |  | ·· | ·· | ·· |  | ·· | ·· | ·· |  |
| **Sub Saharan Africa** |  |  |  |  |  |  |  |  |  |  |  |  |  |  |  |
| Angola | Unknown | ·· |  | 47 | ·· | ·· |  | ·· | ·· | ·· |  | ·· | ·· | ·· |  |
| Benin | Unknown | ·· |  | 11 | ·· | ·· |  | ·· | ·· | ·· |  | ·· | ·· | ·· |  |
| Botswana | Unknown | ·· |  | 23 | ·· | ·· |  | ·· | ·· | ·· |  | ·· | ·· | ·· |  |
| Burkina Faso | Unknown | ·· |  | 27 | ·· | ·· |  | ·· | ·· | ·· |  | ·· | ·· | ·· |  |
| Burundi | Unknown | ·· |  | 13 | ·· | ·· |  | ·· | ·· | ·· |  | ·· | ·· | ·· |  |
| Cameroon | Yes | 2021 | 47 | 76 | ·· | ·· |  | ·· | ·· | ·· |  | ·· | ·· | ·· |  |
| Cabo Verde | Unknown | ·· |  | 5 | ·· | ·· |  | ·· | ·· | ·· |  | ·· | ·· | ·· |  |
| Central African Republic | Unknown | ·· |  | 15 | ·· | ·· |  | ·· | ·· | ·· |  | ·· | ·· | ·· |  |
| Chad | Unknown | ·· |  | 41 | ·· | ·· |  | ·· | ·· | ·· |  | ·· | ·· | ·· |  |
| Comoros | Unknown | ·· |  | 3 | ·· | ·· |  | ·· | ·· | ·· |  | ·· | ·· | ·· |  |
| Côte d'Ivoire | Unknown | ·· |  | 34 | ·· | ·· |  | ·· | ·· | ·· |  | ·· | ·· | ·· |  |
| Democratic Republic of the Congo | Unknown | ·· |  | 142 | ·· | ·· |  | ·· | ·· | ·· |  | ·· | ·· | ·· |  |
| Djibouti | Unknown | ·· |  | 1 | ·· | ·· |  | ·· | ·· | ·· |  | ·· | ·· | ·· |  |
| Equatorial Guinea | Unknown | ·· |  | 15 | ·· | ·· |  | ·· | ·· | ·· |  | ·· | ·· | ·· |  |
| Eritrea | Unknown | ·· |  | N/A | ·· | ·· |  | ·· | ·· | ·· |  | ·· | ·· | ·· |  |
| Eswatini | Unknown | ·· |  | 12 | ·· | ·· |  | ·· | ·· | ·· |  | ·· | ·· | ·· |  |
| Ethiopia | Unknown | ·· |  | 126 | ·· | ·· |  | ·· | ·· | ·· |  | ·· | ·· | ·· |  |
| Gabon | Unknown | ·· |  | 9 | ·· | ·· |  | ·· | ·· | ·· |  | ·· | ·· | ·· |  |
| Gambia | Unknown | ·· |  | 3 | ·· | ·· |  | ·· | ·· | ·· |  | ·· | ·· | ·· |  |
| Ghana | Unknown | ·· |  | 46 | ·· | ·· |  | ·· | ·· | ·· |  | ·· | ·· | ·· |  |
| Guinea | Unknown | ·· |  | 31 | ·· | ·· |  | ·· | ·· | ·· |  | ·· | ·· | ·· |  |
| Guinea-Bissau | Unknown | ·· |  | 3 | ·· | ·· |  | ·· | ·· | ·· |  | ·· | ·· | ·· |  |
| Kenya | Unknown | ·· |  | 134 | ·· | ·· |  | ·· | ·· | ·· |  | ·· | ·· | ·· |  |
| Lesotho | Unknown | ·· |  | 14 | ·· | ·· |  | ·· | ·· | ·· |  | ·· | ·· | ·· |  |
| Liberia | Unknown | ·· |  | 16 | ·· | ·· |  | ·· | ·· | ·· |  | ·· | ·· | ·· |  |
| Madagascar | Unknown | ·· |  | 82 | ·· | ·· |  | ·· | ·· | ·· |  | ·· | ·· | ·· |  |
| Malawi | Yes | 2022 | 48 | 30 | ·· | ·· |  | 7289 | Past year | 2022 | 48 | ·· | ·· | ·· |  |
| Mali | Unknown | ·· |  | 60 | ·· | ·· |  | ·· | ·· | ·· |  | ·· | ·· | ·· |  |
| Mauritania | Unknown | ·· |  | 23 | ·· | ·· |  | ·· | ·· | ·· |  | ·· | ·· | ·· |  |
| Mauritius | Yes | 2022 | 49 | 11 | ·· | ·· |  | ·· | ·· | ·· |  | 8485 | Past year | 2022 | 49 |
| Mozambique | Yes | 2019 | 50 | 157 | ·· | ·· |  | 6022 | Other | 2019 | 50 | ·· | ·· | ·· |  |
| Namibia | Unknown | ·· |  | 13 | ·· | ·· |  | ·· | ·· | ·· |  | ·· | ·· | ·· |  |
| Niger | Unknown | ·· |  | 41 | ·· | ·· |  | ·· | ·· | ·· |  | ·· | ·· | ·· |  |
| Nigeria | Unknown | ·· |  | 240 | ·· | ·· |  | ·· | ·· | ·· |  | ·· | ·· | ·· |  |
| Congo | Unknown | ·· |  | 12 | ·· | ·· |  | ·· | ·· | ·· |  | ·· | ·· | ·· |  |
| Rwanda | Unknown | ·· |  | 13 | ·· | ·· |  | ·· | ·· | ·· |  | ·· | ·· | ·· |  |
| Sao Tome & Principe | Unknown | ·· |  | 1 | ·· | ·· |  | ·· | ·· | ·· |  | ·· | ·· | ·· |  |
| Senegal | Unknown | ·· |  | 37 | ·· | ·· |  | ·· | ·· | ·· |  | ·· | ·· | ·· |  |
| Seychelles | Unknown | ·· |  | 3 | ·· | ·· |  | ·· | ·· | ·· |  | ·· | ·· | ·· |  |
| Sierra Leone | Unknown | ·· |  | 21 | ·· | ·· |  | ·· | ·· | ·· |  | ·· | ·· | ·· |  |
| Somalia | Unknown | ·· |  | 14 | ·· | ·· |  | ·· | ·· | ·· |  | ·· | ·· | ·· |  |
| South Africa | Yes | 2017 | 51 | 235 | ·· | ·· |  | ·· | ·· | ·· |  | ·· | ·· | ·· |  |
| United Republic of Tanzania | Unknown | ·· |  | 126 | ·· | ·· |  | ·· | ·· | ·· |  | ·· | ·· | ·· |  |
| Togo | Unknown | ·· |  | 13 | ·· | ·· |  | ·· | ·· | ·· |  | ·· | ·· | ·· |  |
| Uganda | All facilities | 2024 | 52 | 254 | 254 | 2024 | 52 | ·· | ·· | ·· |  | ·· | ·· | ·· |  |
| Zambia | All facilities | 2023 | 53 | 90 | 90 | 2023 | 53 | ·· | ·· | ·· |  | ·· | ·· | ·· |  |
| Zimbabwe | Unknown | ·· |  | 72 | ·· | ·· |  | ·· | ·· | ·· |  | ·· | ·· | ·· |  |
| **Middle East & North Africa** |  |  |  |  |  |  |  |  |  |  |  |  |  |  |  |
| Algeria | Unknown | ·· |  | 162 | ·· | ·· |  | ·· | ·· | ·· |  | ·· | ·· | ·· |  |
| Bahrain | Unknown | ·· |  | 4 | ·· | ·· |  | ·· | ·· | ·· |  | ·· | ·· | ·· |  |
| Cyprus | All facilities | 2020 | 1 | 1 | 1 | 2020 | 1 | ·· | ·· | ·· |  | ·· | ·· | ·· |  |
| Egypt | Yes | 2019 | 54 | 78 | ·· | ·· |  | ·· | ·· | ·· |  | ·· | ·· | ·· |  |
| Iraq | Yes | 2012 | 55 | 55 | ·· | ·· |  | ·· | ·· | ·· |  | ·· | ·· | ·· |  |
| Israel | All facilities | 2013 | 56 | 30 | 30 | 2013 | 56 | ·· | ·· | ·· |  | ·· | ·· | ·· |  |
| Jordan | Unknown | ·· |  | 17 | ·· | ·· |  | ·· | ·· | ·· |  | ·· | ·· | ·· |  |
| Kuwait | Unknown | ·· |  | 3 | ·· | ·· |  | ·· | ·· | ·· |  | ·· | ·· | ·· |  |
| Lebanon | Yes | 2010 | 57 | 25 | ·· | ·· |  | ·· | ·· | ·· |  | ·· | ·· | ·· |  |
| Libya | Yes | 2015 | 58 | 26 | ·· | ·· |  | ·· | ·· | ·· |  | ·· | ·· | ·· |  |
| Morocco | All facilities | 2021 | 59 | 75 | 75 | 2021 | 59 | ·· | ·· | ·· |  | 15451 | Snapshot | 2021 | 59 |
| Oman | Unknown | ·· |  | 3 | ·· | ·· |  | ·· | ·· | ·· |  | ·· | ·· | ·· |  |
| Occupied Palestinian territories | Unknown | ·· |  | N/A | ·· | ·· |  | ·· | ·· | ·· |  | ·· | ·· | ·· |  |
| Qatar | Unknown | ·· |  | 1 | ·· | ·· |  | ·· | ·· | ·· |  | ·· | ·· | ·· |  |
| Saudi Arabia | Unknown | ·· |  | 110 | ·· | ·· |  | ·· | ·· | ·· |  | ·· | ·· | ·· |  |
| South Sudan | Unknown | ·· |  | 80 | ·· | ·· |  | ·· | ·· | ·· |  | ·· | ·· | ·· |  |
| Sudan | Unknown | ·· |  | 125 | ·· | ·· |  | ·· | ·· | ·· |  | ·· | ·· | ·· |  |
| Syrian Arab Republic | Unknown | ·· |  | 35 | ·· | ·· |  | ·· | ·· | ·· |  | ·· | ·· | ·· |  |
| Tunisia | Unknown | ·· |  | 32 | ·· | ·· |  | ·· | ·· | ·· |  | ·· | ·· | ·· |  |
| Türkiye | Yes | 2022 | 3 | 403 | ·· | ·· |  | ·· | ·· | ·· |  | ·· | ·· | ·· |  |
| United Arab Emirates | Unknown | ·· |  | 21 | ·· | ·· |  | ·· | ·· | ·· |  | ·· | ·· | ·· |  |
| Yemen | Unknown | ·· |  | 43 | ·· | ·· |  | ·· | ·· | ·· |  | ·· | ·· | ·· |  |

**Notes:**

**Ref:** Reference; **HIV**: Human Immunodeficiency Virus.

***Total sites:** Including prisons, jails or other carceral setting, Country level data that informed these regional and global estimates were sourced from the World Prison Brief, collated by the Institute for Crime and Justice Policy Research at Burbeck University. See <https://www.prisonstudies.org/world-prison-brief-data>**;**

** The total number of sites for England and Wales was reported together in the World Prison Brief, therefore the total number of sites for both England and Wales is reported as 120.

† For reporting purposes, these countries or territories are reported separately due to differences in service provision.

*** Pilot data only

·· Indicates there were no data to inform a country’s estimate.

**References for Table 6.3**

1. World Health Organization. Status report on prison health in the WHO European Region 2022. Copenhagen, 2023.

2. Burrows D, Boltaev A, Musa M, Gottfreðsson M, Suleymanova J. Review of the HIV programme in Azerbaijan: November 2014: World Health Organization. Regional Office for Europe, 2016.

3. European Union Drug Agency. EUDA Bulletin - Interventions in Prisons, Europe. 2024.

4. Alexander Panayotov, Georgi Shopov, Illiana Petkova, Tsvetelina Ivanova, Chipeva S. Annual report on the problems related to drugs and drug addiction in Bulgaria 2022: National Focal Point Division Team, Mental Health and Addiction Prevention Directorate, National Center for Public Health and Analysis, 2022.

5. NIHD. HIV in Estonia. Narrative Report for Global AIDS Monitoring 2017. 2017.

6. Rasimaite B. DRID in prisons Lituhania. 2024.

7. Harm Reduction International. Availability, accessibility, acceptability and quality of harm reduction services in Moldovan prisons. London, 2021.

8. Malczewski A, Misiurek, A., Bukowska, B., Chojecki, D., Jabłoński, P., Kidawa, M., Niedźwiedzka-Stadnik, M., Poleganow, A., Radomska, A., Rosińska, M., Sollich, K., Stawecka, E., Struzik, M., Strzelecka, A., & Walichnowska, M. National Report 2013, Poland: new developments, trends and in-depth information on selected issues, 2012.

9. Kolomiets VM, Polshikova NA, Petrov AYU, Kovalenko AL, Talikova EV. Epidemic Situation and Features of Accompanying Therapy in the Treatment of Socially Significant Infectious Diseases in Penitentiary Populations Before, During, and after the COVID-19 Pandemic. [Russian]. *Antibiotiki i Khimioterapiya* 2024; **69(1-2)**: 44-50.

10. Klymova O, Hetman L, Legkostup L. Impact of COVID-19 on access to HIV care in the penitentiary system in Ukraine. *HIV Med* 2021.

11. Andreić J-L. Prison Workbook 2023 - Croatia, 2023.

12. UK Health Security Agency. Taking a place-based approach to tackling hepatitis in prisons. 2023.

13. Monarca R, Madeddu G, Ranieri R, et al. HIV treatment and care among Italian inmates: a one-month point survey. *BMC Infect Dis* 2015.

14. Kugener T, Berndt N, Origer A, Debacker M. Prison workbook 2023 Luxembourg. 2023.

15. de Castro MV, Mendes SCC, Marques JPA, Santos AS, Duarte O. Prison Workbook 2023 - Portugal. 2023.

16. Bell N, Humphries K, Heskin J, Dunn J, Chan SY. A review of sexual health and blood-borne virus care provided to inmates at admission into UK prisons and secure facilities. *HIV Med* 2023; **24(Supplement 3)**: 4.

17. Kilibarda B, Simić D, Baroš S, Brandić I. National Report on Drug Situation in Serbia, 2014, 2014.

18. Perrett SE, Plimmer A, Shankar AG, Craine N. Prevalence of HCV in prisons in Wales, UK and the impact of moving to opt-out HCV testing. *J Public Health (Oxf)* 2020.

19. World Health Organization Western Pacific Region. Joint Review of the Cambodian National Health Sector Response to HIV 2013, 2013.

20. Special Preventive Programme. HIV Surveillance Report - 2021 Update. Centre for Health Protection, Hong Kong Special Administrative Region, 2023.

21. Indonesian National AIDS Commission. Global AIIndonesia Country Progress Report 2014: Indonesian National AIDS Commission, 2014.

22. National AIDS Commission. National HIV and AIDS Strategy and Action Plan 2010 – 2014, 2010.

23. Ministry of Health Malaysia. Global AIDS response progress. Report Malaysia 2015: Ministry of Health Malaysia Kuala Lumpur, 2015.

24. Myanmar Ministry of Health and Sports. National Strategic Framework on Health and Drugs: Myanmar Ministry of Health and Sports, 2020.

25. National AIDS Management Center. 2017 Thailand Progress Report Prevention and Control of AIDS. Bureau of AIDS, TB and STIs, 2017.

26. The Global Fund. Technical Brief: Addresing HIV and TB in Prisons, Pre-Trial Detention and Other Closed Settings, 2020.

27. National AIDS Control Organisation. Sankalak: Status of National AIDS & STD Response (Fifth edition, 2023). New Delhi: NACO, Ministry of Health and Family Welfare, Government of India., 2023.

28. Farhoudi B, SeyedAlinaghi S, Dadras O, et al. Health service provision for disease control among prisoners: a conceptual note. *J Health Res* 2020; **34**(4): 353-8.

29. Maldives Correctional Service. Healthcare in Prison. 2017. https://en.corrections.gov.mv/2017/09/21/healthcare/.

30. National Centre for AIDS and STD Control GoN. Equalize: National HIV Fact Sheet: National Centre for AIDS and STD Control, Government of Nepal, 2022.

31. Kazi AM, Shah SA, Jenkins CA, Shepherd BE, Vermund SH. Risk factors and prevalence of tuberculosis, human immunodeficiency virus, syphilis, hepatitis B virus, and hepatitis C virus among prisoners in Pakistan. *Int J Infect Dis* 2010.

32. Ministry of Health Sri Lanka. NSACP Annual Report 2014, 2014.

33. United Nations Office on Drugs and Crime. Accessibility of HIV Prevention, Treatment and Care Services for People who Use Drugs and Incarcerated People in Azerbaijan, Kazakhstan, Kyrgyzstan, Tajikistan, Turkmenistan, and Uzbekistan: Legislative and Policy Recommentations for Reform. Ashgabat, 2010.

34. United Nations Development Programme. United Nations Development Programme Annual Report on the implementation of UNDP project in support of the Government of the Kyrgyz Republic, funded by The Global Fund to Fight AIDS, Tuberculoses and Malaria - 2014: United Nations Development Programme, 2014.

35. Ministry of Health and Social Protection The Republic of Tajikistan. Reference on the implementation of the National Epidemic Control Program human immunodeficiency syndrome in the Republic of Tajikistan for 2017 2020, 2019.

36. Andrinopoulos K, Kerrigan D, Figueroa JP, et al. Establishment of an HIV/sexually transmitted disease programme and prevalence of infection among incarcerated men in Jamaica. *Int J STD AIDS* 2010.

37. Hariga F. Evaluation and recommendations for the improvement of the health programmes, including for the prevention and treatment of drug dependence and of HIV and AIDS, implemented in the establishments under the responsibility of the federal penitentiary service in Argentina - A follow-up of the 2008 assessment, 2011.

38. Saude Md. Plano Nacional de Saude no Sistema Penitenciario, 2003.

39. Hincapie C. In: Degenhardt L, editor.; 2024.

40. Ministerio de Gobernación. Realizan jornadas de estudio e información sobre el VIH y Sífilis en centros de privación de libertad. 2021. https://dgsp.gob.gt/realizan-jornadas-de-estudio-e-informacion-sobre-el-vih-y-sifilis-en-centros-de-privacion-de-libertad/.

41. Instituto Nacional Penitenciario de Honduras. Realizan pruebas rápidas de VIH y donan kits de higiene en el Centro Penitenciario de La Paz. 2025. https://inp.gob.hn/archivos/14730.

42. Borquez A. Coverage data in Mexico prisons. In: Thomas Santo Jr., editor.; 2024.

43. Calero G, Fratti J, Larranaga J, Gallo S, Hernandez T, Bittar G. State of health of persons deprived of their liberty when entering the Uruguayan Penitentiary System between January and June 2016. *Anales de la Facultad de Medicina* 2017.

44. Correctional Service of Canada. Infectious disease surveillance in Canadian federal penitentiaries 2007–2008: pre-release report. Correctional Service of Canada Ottawa, ON; 2012.

45. Maruschak LM. HIV in Prisons, 2021 – Statistical Tables: Bureau of Justice Statistics, 2023.

46. Simpson PL, Gardoll B, White L, Butler T. HIV policies in Australian prisons: a structured review assessing compliance with international guidelines. *Lancet Reg Health West Pac* 2023; **41**.

47. Noeske J, Ndi NF, Nga FHM, Mely G, Kuaban C. Prison health services across ten central prisons in Cameroon. *J Public Health Afr* 2023; **14**(9).

48. Mallewa J, Kaombe, T & Simbeye, J. Access to preventive, testing, treatment services and retention in care for HIV key populations groups in Malawi. 2023.

49. Ministry of Health and Wellness. Health Statistics Report Mauritius & Rodrigues, 2022.

50. De Seleme J, Couto A, Chicuecue N, et al. Integration of key population classification into national routine HIV testing services in Mozambique, 2019. *J Int AIDS SocConference: 23rd International AIDS Conference Virtual* 2020.

51. Hoffmann CJ, Herce ME, Chimoyi L, et al. Reaching for 90:90:90 in Correctional Facilities in South Africa and Zambia: Virtual Cross-Section of Coverage of HIV Testing and Antiretroviral Therapy during Universal Test and Treat Implementation. *J Acquir Immune Defic Syndr* 2024; **96(5)**: 465-71.

52. Lukoye D, Kalamya JN, Awor AC, et al. Treatment Outcomes for Tuberculosis Infection and Disease Among Persons Deprived of Liberty, Uganda, 2020. *Emerg Infect Dis* 2024; **30**(7): 1402EP-5.

53. Claassen CW, Lindsay B, Siyambango M, et al. PrEP Uptake and Persistence Among Incarcerated People in Zambia: Early Results From a Cohort Study. *Top antiviral med* 2024; **32(1)**: 362.

54. United Nations Office on Drugs and Crime ROMENA. Egypt: UNODC inaugurates first-ever prison Voluntary Confidential Counseling, Testing and aftercare services. 2019.

55. The Joint United Nations Programme on HIV and AIDS. Global AIDS Progress Reporting 2012 Country Progress report Iraq. 2012.

56. Mor Z, Vider H, Grotto I, Tischler Aurkin D. Universal HIV screening of inmates in israeli prisons: Should the policy be updated? *Sexually Transmitted Infections Conference: STI and AIDS World Congress* 2013.

57. Mahfoud Z, Kassak K, Kreidieh K, Shamra S, Ramia S. Prevalence of antibodies to human immunodeficiency virus (HIV), hepatitis B and hepatitis C and risk factors in prisoners in Lebanon. *J Infect Dev Ctries* 2010.

58. National Centre for Disease Control. GARPR 2015 Country Progress Report Libya: National Centre for Disease Control, 2015.

59. Royaume du Maroc Ministère de la Santé et de la Protection Sociale. Rapport National SIDA 2022, 2021.

## *Appendix Table 6.4*: Country-level availability of interventions and programmatic data for HIV Treatment (ART)

|  | | | | **Number of Carceral Sites with HIV Treatment (ART)** | | | | **Number of People Accessing HIV Treatment (ART) in Carceral Settings** | | | |
| --- | --- | --- | --- | --- | --- | --- | --- | --- | --- | --- | --- |
| **Country** | **Available** | **Year** | **Ref** | **Total sites*** | **N Sites with HIV Testing** | **Year** | **Ref** | **N People Accessing HIV Treatment in Carceral Settings** | **Timeframe** | **Year** | **Ref** |
| **Eastern Europe** |  |  |  |  |  |  |  |  |  |  |  |
| Armenia | Yes | 2020 | 1 | 12 | ·· | ·· |  | 26 | Past year | 2020 | 1 |
| Azerbaijan | Yes | 2019 | 2 | 53 | ·· | ·· |  | ·· | ·· | ·· |  |
| Belarus | Yes | 2019 | 2 | 67 | ·· | ·· |  | ·· | ·· | ·· |  |
| Bosnia & Herzegovina | Yes | 2020 | 1 | 13 | ·· | ·· |  | 2 | Past year | 2020 | 1 |
| Bulgaria | Yes | 2022 | 3 | 55 | ·· | ·· |  | ·· | ·· | ·· |  |
| Czechia | Yes | 2022 | 4 | 35 | ·· | ·· |  | 38 | Past year | 2020 | 1 |
| Estonia | All facilities | 2022 | 4 | 3 | 3 | 2022 | 4 | 251 | Past year | 2020 | 1 |
| Georgia | Yes | 2020 | 1 | 14 | ·· | ·· |  | 90 | Past year | 2020 | 1 |
| Hungary | Yes | 2022 | 4 | 30 | 5 | 2022 | 4 | 17 | Past year | 2020 | 1 |
| Latvia | All facilities | 2022 | 4 | 9 | 9 | 2022 | 4 | 403 | Snapshot | 2020 | 1 |
| Lithuania | Yes | 2022 | 4 | 8 | 2 | 2022 | 4 | 131 | Past year | 2020 | 1 |
| Republic of Moldova | All facilities | 2021 | 5 | 17 | 17 | 2021 | 5 | 140 | Snapshot | 2020 | 5 |
| Poland | All facilities | 2022 | 4 | 215 | 215 | 2022 | 4 | 236 | Past year | 2012 | 6 |
| Romania | Yes | 2022 | 4 | 45 | ·· | ·· |  | 384 | Past year | 2020 | 1 |
| Russian Federation | Yes | 2022 | 7 | 872 | ·· | ·· |  | 58105 | Snapshot | 2021 | 8 |
| Slovakia | All facilities | 2022 | 4 | 18 | 18 | 2022 | 4 | 5 | Past year | 2020 | 1 |
| Ukraine | Yes | 2020 | 1 | 110 | ·· | ·· |  | 3601 | Past year | 2021 | 9 |
| **Western Europe** |  |  |  |  |  |  |  |  |  |  |  |
| Albania | Yes | 2020 | 1 | 23 | ·· | ·· |  | 8 | Past year | 2020 | 1 |
| Andorra | Unknown | ·· |  | 1 | ·· | ·· |  | ·· | ·· | ·· |  |
| Austria | All facilities | 2022 | 4 | 27 | 27 | 2022 | 4 | 201 | Past year | 2020 | 1 |
| Belgium | Yes | 2022 | 10 | 35 | ·· | ·· |  | ·· | ·· | ·· |  |
| Croatia | All facilities | 2022 | 11 | 24 | 24 | 2022 | 11 | ·· | ·· | ·· |  |
| Denmark | Yes | 2022 | 10 | 54 | ·· | ·· |  | ·· | ·· | ·· |  |
| England** | All facilities | 2016 | 12 | 120 | 120 | 2016 | 12 | ·· | ·· | ·· |  |
| Finland | All facilities | 2022 | 4 | 26 | 26 | 2022 | 4 | 4 | Census | 2022 | 4 |
| France | All facilities | 2022 | 4 | 187 | 187 | 2022 | 4 | ·· | ·· | ·· |  |
| Germany | Yes | 2022 | 4 | 179 | ·· | ·· |  | 25 | Past year | 2020 | 1 |
| Greece | Yes | 2022 | 4 | 34 | 3 | 2022 | 4 | 110 | Snapshot | 2020 | 1 |
| Greenland | Unknown | ·· |  | 6 | ·· | ·· |  | ·· | ·· | ·· |  |
| Iceland | Unknown | ·· |  | 5 | ·· | ·· |  | ·· | ·· | ·· |  |
| Ireland | Yes | 2020 | 1 | 12 | ·· | ·· |  | 96 | Past year | 2020 | 1 |
| Italy | Yes | 2022 | 4 | 206 | ·· | ·· |  | 275 | Snapshot | 2013 | 13 |
| Liechtenstein | Unknown | ·· |  | 1 | ·· | ·· |  | ·· | ·· | ·· |  |
| Luxembourg | All facilities | 2022 | 14 | 3 | 3 | 2022 | 14 | 6 | Past year | 2020 | 1 |
| Malta | Yes | 2022 | 10 | 1 | ·· | ·· |  | 4 | Past year | 2020 | 1 |
| Monaco | Unknown | ·· |  | 2 | ·· | ·· |  | ·· | ·· | ·· |  |
| Montenegro | Unknown | ·· |  | 3 | ·· | ·· |  | ·· | ·· | ·· |  |
| Netherlands | Yes | 2022 | 10 | 49 | ·· | ·· |  | ·· | ·· | ·· |  |
| North Macedonia | Unknown | ·· |  | 13 | ·· | ·· |  | ·· | ·· | ·· |  |
| Northern Ireland | Unknown | ·· |  | 3 | ·· | ·· |  | ·· | ·· | ·· |  |
| Norway | Yes | 2022 | 10 | 33 | ·· | ·· |  | ·· | ·· | ·· |  |
| Portugal | All facilities | 2022 | 15 | 49 | 49 | 2022 | 15 | 386 | Past year | 2020 | 1 |
| San Marino | Unknown | ·· |  | 1 | ·· | ·· |  | ·· | ·· | ·· |  |
| Scotland | Yes | 2016 | 12 | 15 | ·· | ·· |  | ·· | ·· | ·· |  |
| Serbia | All facilities | 2013 | 16 | 30 | 30 | 2013 | 16 | ·· | ·· | ·· |  |
| Slovenia | All facilities | 2022 | 4 | 7 | 7 | 2022 | 4 | 1 | Past year | 2020 | 1 |
| Spain | All facilities | 2022 | 4 | 82 | 82 | 2022 | 4 | 1566 | Past year | 2020 | 1 |
| Sweden | All facilities | 2022 | 4 | 79 | 79 | 2022 | 4 | ·· | ·· | ·· |  |
| Switzerland | Yes | 2004 | 17 | 88 | 36 | 2004 | 17 | ·· | ·· | ·· |  |
| Wales** | All facilities | 2016 | 12 | 120 | 120 | 2016 | 12 | ·· | ·· | ·· |  |
| **East and South East Asia** |  | ·· |  |  |  |  |  |  |  |  |  |
| Brunei Darussalam | Unknown | ·· |  | 3 | ·· | ·· |  | ·· | ·· | ·· |  |
| Cambodia | Unknown | ·· |  | 29 | ·· | ·· |  | ·· | ·· | ·· |  |
| China | Unknown | ·· |  | 683 | ·· | ·· |  | ·· | ·· | ·· |  |
| Hong Kong† | Unknown | ·· |  | 24 | ·· | ·· |  | ·· | ·· | ·· |  |
| Indonesia | Yes | 2009 | 18 | 526 | 53 | 2009 | 18 | ·· | ·· | ·· |  |
| Japan | Unknown | ·· |  | 184 | ·· | ·· |  | ·· | ·· | ·· |  |
| Lao People's Democratic Republic | Unknown | ·· |  | 19 | ·· | ·· |  | ·· | ·· | ·· |  |
| Malaysia | Yes | 2014 | 19 | 43 | ·· | ·· |  | ·· | ·· | ·· |  |
| Mongolia | Unknown | ·· |  | 50 | ·· | ·· |  | ·· | ·· | ·· |  |
| Myanmar | Yes | 2019 | 20 | 96 | 45 | 2019 | 20 | 3240 | Snapshot | 2019 | 20 |
| Democratic People's Republic of Korea | Unknown | ·· |  | N/A | ·· | ·· |  | ·· | ·· | ·· |  |
| Philippines | Unknown | ·· |  | 440 | ·· | ·· |  | ·· | ·· | ·· |  |
| Singapore | Unknown | ·· |  | 13 | ·· | ·· |  | ·· | ·· | ·· |  |
| Republic of Korea | Unknown | ·· |  | 54 | ·· | ·· |  | ·· | ·· | ·· |  |
| Taiwan† | Yes | 2016 | 21 | 49 | ·· | ·· |  | ·· | ·· | ·· |  |
| Thailand | Yes | 2009 | 22 | 143 | ·· | ·· |  | 1172 | Snapshot | 2009 | 22 |
| Timor-Leste | Unknown | ·· |  | 3 | ·· | ·· |  | ·· | ·· | ·· |  |
| Viet Nam | Yes | 2020 | 23 | 54 | ·· | ·· |  | ·· | ·· | ·· |  |
| **South Asia** |  | ·· |  |  |  |  |  |  |  |  |  |
| Afghanistan | Unknown | ·· |  | 251 | ·· | ·· |  | ·· | ·· | ·· |  |
| Bangladesh | Unknown | ·· |  | 68 | ·· | ·· |  | ·· | ·· | ·· |  |
| Bhutan | Unknown | ·· |  | 7 | ·· | ·· |  | ·· | ·· | ·· |  |
| India | Yes | 2023 | 24 | 1330 | ·· | ·· |  | 5001 | Past year | 2023 | 24 |
| Islamic Republic of Iran | All facilities | 2020 | 2 | 253 | 253 | 2020 | 2 | ·· | ·· | ·· |  |
| Maldives | Unknown | ·· |  | 11 | ·· | ·· |  | ·· | ·· | ·· |  |
| Nepal | Unknown | ·· |  | 74 | ·· | ·· |  | ·· | ·· | ·· |  |
| Pakistan | Unknown | ·· |  | 116 | ·· | ·· |  | ·· | ·· | ·· |  |
| Sri Lanka | Unknown | ·· |  | 60 | ·· | ·· |  | ·· | ·· | ·· |  |
| **Central Asia** |  | ·· |  |  |  |  |  |  |  |  |  |
| Kazakhstan | Yes | 2011 | 25 | 80 | ·· | ·· |  | ·· | ·· | ·· |  |
| Kyrgyzstan | Yes | 2018 | 26 | 28 | ·· | ·· |  | ·· | ·· | ·· |  |
| Tajikistan | Yes | 2019 | 2 | 19 | ·· | ·· |  | ·· | ·· | ·· |  |
| Turkmenistan | No | 2010 | 25 | 27 | ·· | ·· |  | ·· | ·· | ·· |  |
| Uzbekistan | Yes | 2011 | 25 | 54 | ·· | ·· |  | ·· | ·· | ·· |  |
| **Caribbean** |  | ·· |  |  |  |  |  |  |  |  |  |
| Antigua & Barbuda | Unknown | ·· |  | 1 | ·· | ·· |  | ·· | ·· | ·· |  |
| Bahamas | Unknown | ·· |  | 1 | ·· | ·· |  | ·· | ·· | ·· |  |
| Barbados | Unknown | ·· |  | 1 | ·· | ·· |  | ·· | ·· | ·· |  |
| Bermuda | Unknown | ·· |  | 4 | ·· | ·· |  | ·· | ·· | ·· |  |
| Cuba | Unknown | ·· |  | 200 | ·· | ·· |  | ·· | ·· | ·· |  |
| Dominica | Unknown | ·· |  | 1 | ·· | ·· |  | ·· | ·· | ·· |  |
| Dominican Republic | Unknown | ·· |  | 41 | ·· | ·· |  | ·· | ·· | ·· |  |
| Grenada | Unknown | ·· |  | 1 | ·· | ·· |  | ·· | ·· | ·· |  |
| Haiti | All facilities | 2020 | 2 | 17 | 17 | 2020 | 2 | - | ·· | ·· |  |
| Jamaica | Yes | 2010 | 27 | 11 | ·· | ·· |  | ·· | ·· | ·· |  |
| Commonwealth of Puerto Rico | Unknown | ·· |  | 34 | ·· | ·· |  | ·· | ·· | ·· |  |
| Saint Kitts & Nevis | Unknown | ·· |  | 2 | ·· | ·· |  | ·· | ·· | ·· |  |
| Saint Lucia | Unknown | ·· |  | 1 | ·· | ·· |  | ·· | ·· | ·· |  |
| Saint Vincent & the Grenadines | Unknown | ·· |  | 2 | ·· | ·· |  | ·· | ·· | ·· |  |
| Trinidad & Tobago | Unknown | ·· |  | 9 | ·· | ·· |  | ·· | ·· | ·· |  |
| **Latin America** |  | ·· |  |  |  |  |  |  |  |  |  |
| Argentina | Yes | 2011 | 28 | 338 | ·· | ·· |  | ·· | ·· | ·· |  |
| Belize | Unknown | ·· |  | 1 | ·· | ·· |  | ·· | ·· | ·· |  |
| Plurinational State of Bolivia | No | 2005 | 29 | 40 | ·· | ·· |  | ·· | ·· | ·· |  |
| Brazil | All facilities | 2003 | 30 | 1386 | 1386 | 2003 | 30 | ·· | ·· | ·· |  |
| Chile | Unknown | ·· |  | 113 | ·· | ·· |  | ·· | ·· | ·· |  |
| Colombia | Yes | 2024 | 2 | 125 | ·· | ·· |  | ·· | ·· | ·· |  |
| Costa Rica | Unknown | ·· |  | 32 | ·· | ·· |  | ·· | ·· | ·· |  |
| Ecuador | Unknown | ·· |  | 52 | ·· | ·· |  | ·· | ·· | ·· |  |
| El Salvador | Unknown | ·· |  | 25 | ·· | ·· |  | ·· | ·· | ·· |  |
| Guatemala | Unknown | ·· |  | 21 | ·· | ·· |  | ·· | ·· | ·· |  |
| Guyana | Unknown | ·· |  | 7 | ·· | ·· |  | ·· | ·· | ·· |  |
| Honduras | Unknown | ·· |  | 26 | ·· | ·· |  | ·· | ·· | ·· |  |
| Mexico | All facilities | 2024 | 31 | 279 | 279 | 2024 | 31 | ·· | ·· | ·· |  |
| Nicaragua | Unknown | ·· |  | 9 | ·· | ·· |  | ·· | ·· | ·· |  |
| Panama | Unknown | ·· |  | 23 | ·· | ·· |  | ·· | ·· | ·· |  |
| Paraguay | Unknown | ·· |  | 18 | ·· | ·· |  | ·· | ·· | ·· |  |
| Peru | Yes | 2016 | 32 | 68 | ·· | ·· |  | 259 | Snapshot | 2016 | 32 |
| Suriname | Unknown | ·· |  | 21 | ·· | ·· |  | ·· | ·· | ·· |  |
| Uruguay | Unknown | ·· |  | 27 | ·· | ·· |  | ·· | ·· | ·· |  |
| Bolivarian Republic of Venezuela | Unknown | ·· |  | 41 | ·· | ·· |  | ·· | ·· | ·· |  |
| **North America** |  | ·· |  |  |  |  |  |  |  |  |  |
| Canada (Federal) | All facilities | 2008 | 33 | 43 | 43 | 2008 | 33 | 147 | Snapshot | 2008 | 33 |
| Canada (Provincial/Territorial) | Unknown | ·· |  | 173 | ·· | ·· |  | ·· | ·· | ·· |  |
| United States of America (Federal) | All facilities | 2013 | 34 | 102 | 102 | 2013 | 34 | ·· | ·· | ·· |  |
| United States of America (State) | All facilities | 2013 | 34 | 1190 | 1190 | 2013 | 34 | ·· | ·· | ·· |  |
| **Pacific Island States & Terr.** |  | ·· |  |  |  |  |  |  |  |  |  |
| American Samoa | Unknown | ·· |  | 1 | ·· | ·· |  | ·· | ·· | ·· |  |
| Federated States of Micronesia | Unknown | ·· |  | 4 | ·· | ·· |  | ·· | ·· | ·· |  |
| Fiji | Unknown | ·· |  | 15 | ·· | ·· |  | ·· | ·· | ·· |  |
| French Polynesia | Unknown | ·· |  | 2 | ·· | ·· |  | ·· | ·· | ·· |  |
| Guam | Unknown | ·· |  | 2 | ·· | ·· |  | ·· | ·· | ·· |  |
| Kiribati | Unknown | ·· |  | 4 | ·· | ·· |  | ·· | ·· | ·· |  |
| Marshall Islands | Unknown | ·· |  | 2 | ·· | ·· |  | ·· | ·· | ·· |  |
| Nauru | Unknown | ·· |  | 1 | ·· | ·· |  | ·· | ·· | ·· |  |
| New Caledonia | Unknown | ·· |  | 1 | ·· | ·· |  | ·· | ·· | ·· |  |
| Northern Mariana Islands | Unknown | ·· |  | 1 | ·· | ·· |  | ·· | ·· | ·· |  |
| Palau | Unknown | ·· |  | 1 | ·· | ·· |  | ·· | ·· | ·· |  |
| Papua New Guinea | Unknown | ·· |  | 18 | ·· | ·· |  | ·· | ·· | ·· |  |
| Samoa | Unknown | ·· |  | 3 | ·· | ·· |  | ·· | ·· | ·· |  |
| Solomon Islands | Unknown | ·· |  | 6 | ·· | ·· |  | ·· | ·· | ·· |  |
| Tonga | Unknown | ·· |  | 4 | ·· | ·· |  | ·· | ·· | ·· |  |
| Tuvalu | Unknown | ·· |  | 2 | ·· | ·· |  | ·· | ·· | ·· |  |
| Vanuatu | Unknown | ·· |  | 4 | ·· | ·· |  | ·· | ·· | ·· |  |
| **Australasia** |  | ·· |  |  |  |  |  |  |  |  |  |
| Australia | Yes | 2020 | 35 | 111 | ·· | ·· |  | ·· | ·· | ·· |  |
| New Zealand | Yes | 2022 | 36 | 18 | ·· | ·· |  | 3 | Snapshot | 2022 | 36 |
| **Sub Saharan Africa** |  | ·· |  |  |  |  |  |  |  |  |  |
| Angola | Yes | 2020 | 2 | 47 | ·· | ·· |  | ·· | ·· | ·· |  |
| Benin | Unknown | ·· |  | 11 | ·· | ·· |  | ·· | ·· | ·· |  |
| Botswana | Unknown | ·· |  | 23 | ·· | ·· |  | ·· | ·· | ·· |  |
| Burkina Faso | Unknown | ·· |  | 27 | ·· | ·· |  | ·· | ·· | ·· |  |
| Burundi | Unknown | ·· |  | 13 | ·· | ·· |  | ·· | ·· | ·· |  |
| Cameroon | Unknown | ·· |  | 76 | ·· | ·· |  | ·· | ·· | ·· |  |
| Cabo Verde | Unknown | ·· |  | 5 | ·· | ·· |  | ·· | ·· | ·· |  |
| Central African Republic | Unknown | ·· |  | 15 | ·· | ·· |  | ·· | ·· | ·· |  |
| Chad | Unknown | ·· |  | 41 | ·· | ·· |  | ·· | ·· | ·· |  |
| Comoros | Unknown | ·· |  | 3 | ·· | ·· |  | ·· | ·· | ·· |  |
| Côte d'Ivoire | Yes | 2020 | 2 | 34 | ·· | ·· |  | ·· | ·· | ·· |  |
| Democratic Republic of the Congo | Unknown | ·· |  | 142 | ·· | ·· |  | ·· | ·· | ·· |  |
| Djibouti | Unknown | ·· |  | 1 | ·· | ·· |  | ·· | ·· | ·· |  |
| Equatorial Guinea | Unknown | ·· |  | 15 | ·· | ·· |  | ·· | ·· | ·· |  |
| Eritrea | Unknown | ·· |  | N/A | ·· | ·· |  | ·· | ·· | ·· |  |
| Eswatini | Unknown | ·· |  | 12 | ·· | ·· |  | ·· | ·· | ·· |  |
| Ethiopia | Yes | 2016 | 37 | 126 | ·· | ·· |  | ·· | ·· | ·· |  |
| Gabon | Unknown | ·· |  | 9 | ·· | ·· |  | ·· | ·· | ·· |  |
| Gambia | Unknown | ·· |  | 3 | ·· | ·· |  | ·· | ·· | ·· |  |
| Ghana | Unknown | ·· |  | 46 | ·· | ·· |  | ·· | ·· | ·· |  |
| Guinea | Unknown | ·· |  | 31 | ·· | ·· |  | ·· | ·· | ·· |  |
| Guinea-Bissau | Unknown | ·· |  | 3 | ·· | ·· |  | ·· | ·· | ·· |  |
| Kenya | Yes | 2012 | 38 | 134 | ·· | ·· |  | ·· | ·· | ·· |  |
| Lesotho | Yes | 2020 | 2 | 14 | ·· | ·· |  | ·· | ·· | ·· |  |
| Liberia | Unknown | ·· |  | 16 | ·· | ·· |  | ·· | ·· | ·· |  |
| Madagascar | Unknown | ·· |  | 82 | ·· | ·· |  | ·· | ·· | ·· |  |
| Malawi | Yes | 2022 | 39 | 30 | ·· | ·· |  | 1534 | Snapshot | 2022 | 39 |
| Mali | Unknown | ·· |  | 60 | ·· | ·· |  | ·· | ·· | ·· |  |
| Mauritania | Unknown | ·· |  | 23 | ·· | ·· |  | ·· | ·· | ·· |  |
| Mauritius | Yes | 2022 | 40 | 11 | ·· | ·· |  | 267 | Snapshot | 2022 | 40 |
| Mozambique | Yes | 2023 | 41 | 157 | ·· | ·· |  | ·· | ·· | ·· |  |
| Namibia | Unknown | ·· |  | 13 | ·· | ·· |  | ·· | ·· | ·· |  |
| Niger | Unknown | ·· |  | 41 | ·· | ·· |  | ·· | ·· | ·· |  |
| Nigeria | Unknown | ·· |  | 240 | ·· | ·· |  | ·· | ·· | ·· |  |
| Congo | Unknown | ·· |  | 12 | ·· | ·· |  | ·· | ·· | ·· |  |
| Rwanda | Unknown | ·· |  | 13 | ·· | ·· |  | ·· | ·· | ·· |  |
| Sao Tome & Principe | Unknown | ·· |  | 1 | ·· | ·· |  | ·· | ·· | ·· |  |
| Senegal | Unknown | ·· |  | 37 | ·· | ·· |  | ·· | ·· | ·· |  |
| Seychelles | Unknown | ·· |  | 3 | ·· | ·· |  | ·· | ·· | ·· |  |
| Sierra Leone | Unknown | ·· |  | 21 | ·· | ·· |  | ·· | ·· | ·· |  |
| Somalia | Unknown | ·· |  | 14 | ·· | ·· |  | ·· | ·· | ·· |  |
| South Africa | Yes | 2017 | 42 | 235 | ·· | ·· |  | ·· | ·· | ·· |  |
| United Republic of Tanzania | Unknown | ·· |  | 126 | ·· | ·· |  | ·· | ·· | ·· |  |
| Togo | Unknown | ·· |  | 13 | ·· | ·· |  | ·· | ·· | ·· |  |
| Uganda | Unknown*** | 2009 | 43 | 254 | ·· | ·· |  | ·· | ·· | ·· |  |
| Zambia | Yes | 2021 | 44 | 90 | ·· | ·· |  | ·· | ·· | ·· |  |
| Zimbabwe | Unknown | ·· |  | 72 | ·· | ·· |  | ·· | ·· | ·· |  |
| **Middle East & North Africa** |  | ·· |  |  |  |  |  |  |  |  |  |
| Algeria | Unknown | ·· |  | 162 | ·· | ·· |  | ·· | ·· | ·· |  |
| Bahrain | Unknown | ·· |  | 4 | ·· | ·· |  | ·· | ·· | ·· |  |
| Cyprus | Yes | 2020 | 1 | 1 | ·· | ·· |  | 4 | Snapshot | 2020 | 1 |
| Egypt | Yes | 2019 | 45 | 78 | ·· | ·· |  | ·· | ·· | ·· |  |
| Iraq | Unknown | ·· |  | 55 | ·· | ·· |  | ·· | ·· | ·· |  |
| Israel | Unknown | ·· |  | 30 | ·· | ·· |  | ·· | ·· | ·· |  |
| Jordan | Unknown | ·· |  | 17 | ·· | ·· |  | ·· | ·· | ·· |  |
| Kuwait | Unknown | ·· |  | 3 | ·· | ·· |  | ·· | ·· | ·· |  |
| Lebanon | Unknown | ·· |  | 25 | ·· | ·· |  | ·· | ·· | ·· |  |
| Libya | Yes | 2015 | 46 | 26 | ·· | ·· |  | 59 | Other | 2015 | 46 |
| Morocco | Yes | 2021 | 47 | 75 | ·· | ·· |  | ·· | ·· | ·· |  |
| Oman | Unknown | ·· |  | 3 | ·· | ·· |  | ·· | ·· | ·· |  |
| Occupied Palestinian territories | Unknown | ·· |  | N/A | ·· | ·· |  | ·· | ·· | ·· |  |
| Qatar | Unknown | ·· |  | 1 | ·· | ·· |  | ·· | ·· | ·· |  |
| Saudi Arabia | Unknown | ·· |  | 110 | ·· | ·· |  | ·· | ·· | ·· |  |
| South Sudan | Unknown | ·· |  | 80 | ·· | ·· |  | ·· | ·· | ·· |  |
| Sudan | Unknown | ·· |  | 125 | ·· | ·· |  | ·· | ·· | ·· |  |
| Syrian Arab Republic | Unknown | ·· |  | 35 | ·· | ·· |  | ·· | ·· | ·· |  |
| Tunisia | Unknown | ·· |  | 32 | ·· | ·· |  | ·· | ·· | ·· |  |
| Türkiye | Yes | 2022 | 4 | 403 | ·· | ·· |  | ·· | ·· | ·· |  |
| United Arab Emirates | Unknown | ·· |  | 21 | ·· | ·· |  | ·· | ·· | ·· |  |
| Yemen | Unknown | ·· |  | 43 | ·· | ·· |  | ·· | ·· | ·· |  |

**Notes:**

**Ref:** Reference; **HIV**: Human Immunodeficiency Virus; **ART**: Antiretroviral Therapy.

***Total sites:** Including prisons, jails or other carceral setting, Country level data that informed these regional and global estimates were sourced from the World Prison Brief, collated by the Institute for Crime and Justice Policy Research at Burbeck University. See: <https://www.prisonstudies.org/world-prison-brief-data>**;**

** The total number of sites for England and Wales was reported together in the World Prison Brief, therefore the total number of sites for both England and Wales is reported as 120.

† For reporting purposes, these countries or territories are reported separately due to differences in service provision.

*** Pilot data only

·· Indicates there were no data to inform a country’s estimate.

**References for Table 6.4**

1. World Health Organization. Status report on prison health in the WHO European Region 2022. Copenhagen, 2023.

2. Joint United Nations Programme on HIV/AIDS. UNAIDS DATA 2020: UNAIDS, 2020.

3. Alexander Panayotov, Georgi Shopov, Illiana Petkova, Tsvetelina Ivanova, Chipeva S. Annual report on the problems related to drugs and drug addiction in Bulgaria 2022: National Focal Point Division Team, Mental Health and Addiction Prevention Directorate, National Center for Public Health and Analysis, 2022.

4. European Union Drug Agency. EUDA Bulletin - Interventions in Prisons, Europe. 2024.

5. Harm Reduction International. Availability, accessibility, acceptability and quality of harm reduction services in Moldovan prisons. London, 2021.

6. Malczewski A, Misiurek, A., Bukowska, B., Chojecki, D., Jabłoński, P., Kidawa, M., Niedźwiedzka-Stadnik, M., Poleganow, A., Radomska, A., Rosińska, M., Sollich, K., Stawecka, E., Struzik, M., Strzelecka, A., & Walichnowska, M. National Report 2013, Poland: new developments, trends and in-depth information on selected issues, 2012.

7. International Treatment Preparedness Coalition. Monitoring results of state procurement of ARV drugs in 2022, 2023.

8. Egorova NV, Babikhina, K.A., Golovin, S.E., Soloviev, D.V, Vereshchagina, Y.O., Godlevsky, D.V., Mikhailov, A.V., T.A. Khan, T.A. & Shibaeva, M.V. . Analysis of ARV Procurement in the Russian Federation in 2021, 2022.

9. Klymova O, Hetman L, Legkostup L. Impact of COVID-19 on access to HIV care in the penitentiary system in Ukraine. *HIV Med* 2021.

10. European Union Drug Agency. EUDA Prison Data 2024. Lisbon; 2024.

11. Andreić J-L. Prison Workbook 2023 - Croatia, 2023.

12. Zurhold H, Stöver H. Provision of harm reduction and drug treatment services in custodial settings – Findings from the European ACCESS study. *Drugs Educ Prev Policy* 2016.

13. Monarca R, Madeddu G, Ranieri R, et al. HIV treatment and care among Italian inmates: a one-month point survey. *BMC Infect Dis* 2015.

14. Kugener T, Berndt N, Origer A, Debacker M. Prison workbook 2023 Luxembourg. 2023.

15. de Castro MV, Mendes SCC, Marques JPA, Santos AS, Duarte O. Prison Workbook 2023 - Portugal. 2023.

16. Kilibarda B, Simić D, Baroš S, Brandić I. National Report on Drug Situation in Serbia, 2014, 2014.

17. Gerlich MG, Frick U, Pirktl L, Uchtenhagen A. Detection and treatment of HIV and hepatitis virus infections in Swiss correctional facilities. *Int J Public Health* 2008.

18. National AIDS Commission. National HIV and AIDS Strategy and Action Plan 2010 – 2014, 2010.

19. Ministry of Health Malaysia. Global AIDS response progress. Report Malaysia 2015: Ministry of Health Malaysia Kuala Lumpur, 2015.

20. Myanmar Ministry of Health and Sports. National Strategic Framework on Health and Drugs: Myanmar Ministry of Health and Sports, 2020.

21. Lin T, Chen C-H, Chou P. Effects of combination approach on harm reduction programs: the Taiwan experience. *Harm Reduct J* 2016; **13**(1): 1-10.

22. National AIDS Prevention and Alleviation Committee. UNGASS COUNTRY PROGRESS REPORT THAILAND. *National AIDS Prevention and Alleviation Committee* 2009.

23. The Global Fund. Technical Brief: Addresing HIV and TB in Prisons, Pre-Trial Detention and Other Closed Settings, 2020.

24. National AIDS Control Organisation. Sankalak: Status of National AIDS & STD Response (Fifth edition, 2023). New Delhi: NACO, Ministry of Health and Family Welfare, Government of India., 2023.

25. United Nations Office on Drugs and Crime. Accessibility of HIV Prevention, Treatment and Care Services for People who Use Drugs and Incarcerated People in Azerbaijan, Kazakhstan, Kyrgyzstan, Tajikistan, Turkmenistan, and Uzbekistan: Legislative and Policy Recommentations for Reform. Ashgabat, 2010.

26. Bachireddy C, Shrestha R, Bromberg DJ, et al. Methadone within prison and linkage to and retention in treatment upon community release for people with opioid use disorder in Kyrgyzstan: Evaluation of a national program. *Int J Drug Policy* 2022.

27. Andrinopoulos K, Kerrigan D, Figueroa JP, et al. Establishment of an HIV/sexually transmitted disease programme and prevalence of infection among incarcerated men in Jamaica. *Int J STD AIDS* 2010.

28. Hariga F. Evaluation and recommendations for the improvement of the health programmes, including for the prevention and treatment of drug dependence and of HIV and AIDS, implemented in the establishments under the responsibility of the federal penitentiary service in Argentina - A follow-up of the 2008 assessment, 2011.

29. Lambert ML, Torrico F, Billot C, Mazina D, Marleen B, Van der Stuyft P. Street youths are the only high-risk group for HIV in a low-prevalence South American country. *Sex Transm Dis* 2005; **32**(4): 240-2.

30. Saude Md. Plano Nacional de Saude no Sistema Penitenciario, 2003.

31. Borquez A. Coverage data in Mexico prisons. In: Thomas Santo Jr., editor.; 2024.

32. Instituto Nacional de Estadistica e Informatica. Perú: Primer Censo Nacional Penitenciario 2016. Perú, 2016.

33. Correctional Service of Canada. Infectious disease surveillance in Canadian federal penitentiaries 2007–2008: pre-release report. Correctional Service of Canada Ottawa, ON; 2012.

34. Westergaard RP, Spaulding AC, Flanigan TP. HIV among persons incarcerated in the USA: a review of evolving concepts in testing, treatment, and linkage to community care. *Curr Opin Infect Dis* 2013.

35. Simpson PL, Gardoll B, White L, Butler T. HIV policies in Australian prisons: a structured review assessing compliance with international guidelines. *Lancet Reg Health West Pac* 2023; **41**.

36. Lythgoe J, Kolodziej, J & Hollingshead, B. People living with HIV in prisons. 2022.

37. Getaneh M, Reta MM, Assefa D, Yohannis Z, Demilew D. Two-third of inmates were depressed among HIV positive prisoners at central prison (Kaliti), Addis Ababa, Ethiopia. *BMC Res Notes* 2019; **12**(1): 170.

38. Ministry of Health Kenya. MARPs Surveillance Report. *Ministry of Health, National AIDS & STI Control Programme-NASCOP* 2012.

39. Mallewa J, Kaombe, T & Simbeye, J. Access to preventive, testing, treatment services and retention in care for HIV key populations groups in Malawi. 2023.

40. Ministry of Health and Wellness. Health Statistics Report Mauritius & Rodrigues, 2022.

41. Malimane I, Seleme J, Maibaze G, et al. Scale-up of HIV Services for Key Population in Mozambique, 2020-2023. *medRxiv* 2024; **30**.

42. Hoffmann CJ, Herce ME, Chimoyi L, et al. Reaching for 90:90:90 in Correctional Facilities in South Africa and Zambia: Virtual Cross-Section of Coverage of HIV Testing and Antiretroviral Therapy during Universal Test and Treat Implementation. *J Acquir Immune Defic Syndr* 2024; **96(5)**: 465-71.

43. Isabirye MB, Musoke Seruma E, Nambafu S, Tayebwa E, Mpiima D. Scaling up HIV prevention services among prisoners in Uganda - TASO Jinja Experience. *Sexually Transmitted Infections Conference: STI and AIDS World Congress* 2013.

44. Lindsay B, Nyirongo N, Mwango L, et al. Initial implementation of HIV pre-exposure prophylaxis for people who are incarcerated in Zambia: a cross-sectional observational study. *Lancet HIV* 2023.

45. United Nations Office on Drugs and Crime ROMENA. Egypt: UNODC inaugurates first-ever prison Voluntary Confidential Counseling, Testing and aftercare services. 2019.

46. National Centre for Disease Control. GARPR 2015 Country Progress Report Libya: National Centre for Disease Control, 2015.

47. Royaume du Maroc Ministère de la Santé et de la Protection Sociale. Rapport National SIDA 2022, 2021.

## *Appendix Table 6.5*: Country-level availability of interventions and programmatic data for HCV Testing

|  | | | | **Number of Carceral Sites with HCV Testing** | | | | **Number of People Accessing HCV Testing in Carceral Settings** | | | | **Number of HCV Tests Distributed** | | | |
| --- | --- | --- | --- | --- | --- | --- | --- | --- | --- | --- | --- | --- | --- | --- | --- |
| **Country** | **Available** | **Year** | **Ref** | **Total sites*** | **N Sites with HCV Testing** | **Year** | **Ref** | **N People Accessing HCV Testing in Carceral Settings** | **Timeframe** | **Year** | **Ref** | **N** | **Timeframe** | **Year** | **Ref** |
| **Eastern Europe** |  |  |  |  |  |  |  |  |  |  |  |  |  |  |  |
| Armenia | All facilities | 2020 | 1 | 12 | 12 | 2020 | 1 | ·· | ·· | ·· |  | ·· | ·· | ·· |  |
| Azerbaijan | Unknown | ·· |  | 53 | ·· | ·· |  | ·· | ·· | ·· |  | ·· | ·· | ·· |  |
| Belarus | Unknown | ·· |  | 67 | ·· | ·· |  | ·· | ·· | ·· |  | ·· | ·· | ·· |  |
| Bosnia & Herzegovina | Yes | 2020 | 1 | 13 | ·· | ·· |  | ·· | ·· | ·· |  | ·· | ·· | ·· |  |
| Bulgaria | Yes | 2022 | 2 | 55 | 5 | 2022 | 2 | 1858 | Snapshot | 2024 | 3 | ·· | ·· | ·· |  |
| Czechia | All facilities | 2020 | 1 | 35 | 35 | 2020 | 1 | ·· | ·· | ·· |  | ·· | ·· | ·· |  |
| Estonia | All facilities | 2022 | 2 | 3 | 3 | 2022 | 2 | ·· | ·· | ·· |  | ·· | ·· | ·· |  |
| Georgia | All facilities | 2020 | 1 | 14 | 14 | 2020 | 1 | 13500 | Other | 2015 | 4 | ·· | ·· | ·· |  |
| Hungary | Yes | 2022 | 2 | 30 | 12 | 2022 | 2 | 5779 | Other | 2019 | 5 | ·· | ·· | ·· |  |
| Latvia | All facilities | 2022 | 2 | 9 | 9 | 2022 | 2 | ·· | ·· | ·· |  | 684 | Snapshot | 2022 | 2 |
| Lithuania | Yes | 2022 | 2 | 8 | 2 | 2022 | 2 | ·· | ·· | ·· |  | ·· | ·· | ·· |  |
| Republic of Moldova | All facilities | 2024 | 6 | 17 | 17 | 2024 | 6 | ·· | ·· | ·· |  | 375 | Other | 2021 | 7 |
| Poland | All facilities | 2022 | 2 | 215 | 215 | 2022 | 2 | ·· | ·· | ·· |  | ·· | ·· | ·· |  |
| Romania | Yes | 2022 | 2 | 45 | ·· | ·· |  | ·· | ·· | ·· |  | ·· | ·· | ·· |  |
| Russian Federation | Unknown | ·· |  | 872 | ·· | ·· |  | ·· | ·· | ·· |  | ·· | ·· | ·· |  |
| Slovakia | All facilities | 2022 | 2 | 18 | 18 | 2022 | 2 | ·· | ·· | ·· |  | ·· | ·· | ·· |  |
| Ukraine | All facilities | 2020 | 1 | 110 | 110 | 2020 | 1 | ·· | ·· | ·· |  | ·· | ·· | ·· |  |
| **Western Europe** |  |  |  |  |  |  |  |  |  |  |  |  |  |  |  |
| Albania | Yes | 2020 | 1 | 23 | ·· | ·· |  | ·· | ·· | ·· |  | ·· | ·· | ·· |  |
| Andorra | Unknown | ·· |  | 1 | ·· | ·· |  | ·· | ·· | ·· |  | ·· | ·· | ·· |  |
| Austria | All facilities | 2022 | 2 | 27 | 27 | 2022 | 2 | ·· | ·· | ·· |  | ·· | ·· | ·· |  |
| Belgium | All facilities | 2020 | 1 | 35 | 35 | 2020 | 1 | ·· | ·· | ·· |  | ·· | ·· | ·· |  |
| Croatia | All facilities | 2022 | 8 | 24 | 24 | 2022 | 8 | ·· | ·· | ·· |  | 462 | Past year | 2022 | 8 |
| Denmark | Yes | 2022 | 2 | 54 | ·· | ·· |  | ·· | ·· | ·· |  | ·· | ·· | ·· |  |
| England** | All facilities | 2024 | 9 | 120 | 120 | 2024 | 9 | 22626 | Past year | 2021 | 10 | ·· | ·· | ·· |  |
| Finland | All facilities | 2022 | 2 | 26 | 26 | 2022 | 2 | ·· | ·· | ·· |  | ·· | ·· | ·· |  |
| France | All facilities | 2022 | 2 | 187 | 187 | 2022 | 2 | 692 | Snapshot | 2020 | 11 | ·· | ·· | ·· |  |
| Germany | Yes | 2022 | 2 | 179 | ·· | ·· |  | ·· | ·· | ·· |  | ·· | ·· | ·· |  |
| Greece | All facilities | 2020 | 1 | 34 | 34 | 2020 | 1 | ·· | ·· | ·· |  | ·· | ·· | ·· |  |
| Greenland | Unknown | ·· |  | 6 | ·· | ·· |  | ·· | ·· | ·· |  | ·· | ·· | ·· |  |
| Iceland | Yes | 2020 | 12 | 5 | ·· | ·· |  | ·· | ·· | ·· |  | ·· | ·· | ·· |  |
| Ireland | All facilities | 2022 | 2 | 12 | 12 | 2022 | 2 | ·· | ·· | ·· |  | ·· | ·· | ·· |  |
| Italy | All facilities | 2020 | 1 | 206 | 206 | 2020 | 1 | ·· | ·· | ·· |  | ·· | ·· | ·· |  |
| Liechtenstein | Unknown | ·· |  | 1 | ·· | ·· |  | ·· | ·· | ·· |  | ·· | ·· | ·· |  |
| Luxembourg | Yes | 2022 | 13 | 3 | 1 | 2022 | 13 | ·· | ·· | ·· |  | ·· | ·· | ·· |  |
| Malta | All facilities | 2020 | 1 | 1 | 1 | 2020 | 1 | ·· | ·· | ·· |  | ·· | ·· | ·· |  |
| Monaco | All facilities | 2020 | 1 | 2 | 2 | 2020 | 1 | ·· | ·· | ·· |  | ·· | ·· | ·· |  |
| Montenegro | Unknown | ·· |  | 3 | ·· | ·· |  | ·· | ·· | ·· |  | ·· | ·· | ·· |  |
| Netherlands | Yes | 2021 | 2 | 49 | ·· | ·· |  | ·· | ·· | ·· |  | ·· | ·· | ·· |  |
| North Macedonia | Unknown | ·· |  | 13 | ·· | ·· |  | ·· | ·· | ·· |  | ·· | ·· | ·· |  |
| Northern Ireland | All facilities | 2020 | 1 | 3 | 3 | 2020 | 1 | ·· | ·· | ·· |  | ·· | ·· | ·· |  |
| Norway | Yes | 2022 | 2 | 33 | ·· | ·· |  | ·· | ·· | ·· |  | ·· | ·· | ·· |  |
| Portugal | All facilities | 2022 | 14 | 49 | 49 | 2022 | 14 | ·· | ·· | ·· |  | ·· | ·· | ·· |  |
| San Marino | Unknown | ·· |  | 1 | ·· | ·· |  | ·· | ·· | ·· |  | ·· | ·· | ·· |  |
| Scotland | All facilities | 2022 | 15 | 15 | 15 | 2022 | 15 | 2036 | Past year | 2018 | 16 | ·· | ·· | ·· |  |
| Serbia | Yes | 2013 | 17 | 30 | ·· | ·· |  | ·· | ·· | ·· |  | ·· | ·· | ·· |  |
| Slovenia | All facilities | 2022 | 2 | 7 | 7 | 2022 | 2 | ·· | ·· | ·· |  | ·· | ·· | ·· |  |
| Spain | All facilities | 2022 | 2 | 82 | 82 | 2022 | 2 | ·· | ·· | ·· |  | ·· | ·· | ·· |  |
| Sweden | All facilities | 2022 | 2 | 79 | 79 | 2022 | 2 | ·· | ·· | ·· |  | ·· | ·· | ·· |  |
| Switzerland | Yes | 2020 | 1 | 88 | ·· | ·· |  | ·· | ·· | ·· |  | ·· | ·· | ·· |  |
| Wales | All facilities | 2020 | 1 | 120 | 120 | 2020 | 1 | ·· | ·· | ·· |  | ·· | ·· | ·· |  |
| **East and South East Asia** |  |  |  |  |  |  |  |  |  |  |  |  |  |  |  |
| Brunei Darussalam | Unknown | ·· |  | 3 | ·· | ·· |  | ·· | ·· | ·· |  | ·· | ·· | ·· |  |
| Cambodia | Unknown | ·· |  | 29 | ·· | ·· |  | ·· | ·· | ·· |  | ·· | ·· | ·· |  |
| China | Unknown | ·· |  | 683 | ·· | ·· |  | ·· | ·· | ·· |  | ·· | ·· | ·· |  |
| Hong Kong† | Unknown | ·· |  | 24 | ·· | ·· |  | ·· | ·· | ·· |  | ·· | ·· | ·· |  |
| Indonesia | Unknown*** | 2019 | 18 | 526 | ·· | ·· |  | ·· | ·· | ·· |  | ·· | ·· | ·· |  |
| Japan | Unknown | ·· |  | 184 | ·· | ·· |  | ·· | ·· | ·· |  | ·· | ·· | ·· |  |
| Lao People's Democratic Republic | Unknown | ·· |  | 19 | ·· | ·· |  | ·· | ·· | ·· |  | ·· | ·· | ·· |  |
| Malaysia | Unknown | ·· |  | 43 | ·· | ·· |  | ·· | ·· | ·· |  | ·· | ·· | ·· |  |
| Mongolia | Unknown | ·· |  | 50 | ·· | ·· |  | ·· | ·· | ·· |  | ·· | ·· | ·· |  |
| Myanmar | Unknown | ·· |  | 96 | ·· | ·· |  | ·· | ·· | ·· |  | ·· | ·· | ·· |  |
| Democratic People's Republic of Korea | Unknown | ·· |  | N/A | ·· | ·· |  | ·· | ·· | ·· |  | ·· | ·· | ·· |  |
| Philippines | Unknown | ·· |  | 440 | ·· | ·· |  | ·· | ·· | ·· |  | ·· | ·· | ·· |  |
| Singapore | Unknown | ·· |  | 13 | ·· | ·· |  | ·· | ·· | ·· |  | ·· | ·· | ·· |  |
| Republic of Korea | Unknown | ·· |  | 54 | ·· | ·· |  | ·· | ·· | ·· |  | ·· | ·· | ·· |  |
| Taiwan† | Yes | 2023 | 19 | 49 | ·· | ·· |  | ·· | ·· | ·· |  | ·· | ·· | ·· |  |
| Thailand | Unknown | ·· |  | 143 | ·· | ·· |  | ·· | ·· | ·· |  | ·· | ·· | ·· |  |
| Timor-Leste | Unknown | ·· |  | 3 | ·· | ·· |  | ·· | ·· | ·· |  | ·· | ·· | ·· |  |
| Viet Nam | Unknown | ·· |  | 54 | ·· | ·· |  | ·· | ·· | ·· |  | ·· | ·· | ·· |  |
| **South Asia** |  |  |  |  |  |  |  |  |  |  |  |  |  |  |  |
| Afghanistan | Unknown | ·· |  | 251 | ·· | ·· |  | ·· | ·· | ·· |  | ·· | ·· | ·· |  |
| Bangladesh | Unknown | ·· |  | 68 | ·· | ·· |  | ·· | ·· | ·· |  | ·· | ·· | ·· |  |
| Bhutan | Unknown | ·· |  | 7 | ·· | ·· |  | ·· | ·· | ·· |  | ·· | ·· | ·· |  |
| India | Yes | 2023 | 20 | 1330 | ·· | ·· |  | 219413 | Past year | 2023 | 20 | ·· | ·· | ·· |  |
| Islamic Republic of Iran | Unknown*** | 2021 | 21 | 253 | ·· | ·· |  | ·· | ·· | ·· |  | ·· | ·· | ·· |  |
| Maldives | Yes | 2017 | 22 | 11 | ·· | ·· |  | ·· | ·· | ·· |  | ·· | ·· | ·· |  |
| Nepal | Unknown | ·· |  | 74 | ·· | ·· |  | ·· | ·· | ·· |  | ·· | ·· | ·· |  |
| Pakistan | Unknown | ·· |  | 116 | ·· | ·· |  | ·· | ·· | ·· |  | ·· | ·· | ·· |  |
| Sri Lanka | Unknown | ·· |  | 60 | ·· | ·· |  | ·· | ·· | ·· |  | ·· | ·· | ·· |  |
| **Central Asia** |  |  |  |  |  |  |  |  |  |  |  |  |  |  |  |
| Kazakhstan | Unknown | ·· |  | 80 | ·· | ·· |  | ·· | ·· | ·· |  | ·· | ·· | ·· |  |
| Kyrgyzstan | Unknown | ·· |  | 28 | ·· | ·· |  | ·· | ·· | ·· |  | ·· | ·· | ·· |  |
| Tajikistan | Unknown | ·· |  | 19 | ·· | ·· |  | ·· | ·· | ·· |  | ·· | ·· | ·· |  |
| Turkmenistan | Unknown | ·· |  | 27 | ·· | ·· |  | ·· | ·· | ·· |  | ·· | ·· | ·· |  |
| Uzbekistan | Unknown | ·· |  | 54 | ·· | ·· |  | ·· | ·· | ·· |  | ·· | ·· | ·· |  |
| **Caribbean** |  |  |  |  |  |  |  |  |  |  |  |  |  |  |  |
| Antigua & Barbuda | Unknown | ·· |  | 1 | ·· | ·· |  | ·· | ·· | ·· |  | ·· | ·· | ·· |  |
| Bahamas | Unknown | ·· |  | 1 | ·· | ·· |  | ·· | ·· | ·· |  | ·· | ·· | ·· |  |
| Barbados | Unknown | ·· |  | 1 | ·· | ·· |  | ·· | ·· | ·· |  | ·· | ·· | ·· |  |
| Bermuda | Unknown | ·· |  | 4 | ·· | ·· |  | ·· | ·· | ·· |  | ·· | ·· | ·· |  |
| Cuba | Unknown | ·· |  | 200 | ·· | ·· |  | ·· | ·· | ·· |  | ·· | ·· | ·· |  |
| Dominica | Unknown | ·· |  | 1 | ·· | ·· |  | ·· | ·· | ·· |  | ·· | ·· | ·· |  |
| Dominican Republic | Unknown | ·· |  | 41 | ·· | ·· |  | ·· | ·· | ·· |  | ·· | ·· | ·· |  |
| Grenada | Unknown | ·· |  | 1 | ·· | ·· |  | ·· | ·· | ·· |  | ·· | ·· | ·· |  |
| Haiti | Unknown | ·· |  | 17 | ·· | ·· |  | ·· | ·· | ·· |  | ·· | ·· | ·· |  |
| Jamaica | Unknown | ·· |  | 11 | ·· | ·· |  | ·· | ·· | ·· |  | ·· | ·· | ·· |  |
| Commonwealth of Puerto Rico | Unknown | ·· |  | 34 | ·· | ·· |  | ·· | ·· | ·· |  | ·· | ·· | ·· |  |
| Saint Kitts & Nevis | Unknown | ·· |  | 2 | ·· | ·· |  | ·· | ·· | ·· |  | ·· | ·· | ·· |  |
| Saint Lucia | Unknown | ·· |  | 1 | ·· | ·· |  | ·· | ·· | ·· |  | ·· | ·· | ·· |  |
| Saint Vincent & the Grenadines | Unknown | ·· |  | 2 | ·· | ·· |  | ·· | ·· | ·· |  | ·· | ·· | ·· |  |
| Trinidad & Tobago | Unknown | ·· |  | 9 | ·· | ·· |  | ·· | ·· | ·· |  | ·· | ·· | ·· |  |
| **Latin America** |  |  |  |  |  |  |  |  |  |  |  |  |  |  |  |
| Argentina | Unknown | ·· |  | 338 | ·· | ·· |  | ·· | ·· | ·· |  | ·· | ·· | ·· |  |
| Belize | Unknown | ·· |  | 1 | ·· | ·· |  | ·· | ·· | ·· |  | ·· | ·· | ·· |  |
| Plurinational State of Bolivia | Unknown | ·· |  | 40 | ·· | ·· |  | ·· | ·· | ·· |  | ·· | ·· | ·· |  |
| Brazil | All facilities | 2003 | 23 | 1386 | 1386 | 2003 | 23 | ·· | ·· | ·· |  | ·· | ·· | ·· |  |
| Chile | Unknown | ·· |  | 113 | ·· | ·· |  | ·· | ·· | ·· |  | ·· | ·· | ·· |  |
| Colombia | Yes | 2024 | 24 | 125 | ·· | ·· |  | ·· | ·· | ·· |  | ·· | ·· | ·· |  |
| Costa Rica | Unknown | ·· |  | 32 | ·· | ·· |  | ·· | ·· | ·· |  | ·· | ·· | ·· |  |
| Ecuador | Unknown | ·· |  | 52 | ·· | ·· |  | ·· | ·· | ·· |  | ·· | ·· | ·· |  |
| El Salvador | Unknown | ·· |  | 25 | ·· | ·· |  | ·· | ·· | ·· |  | ·· | ·· | ·· |  |
| Guatemala | Unknown | ·· |  | 21 | ·· | ·· |  | ·· | ·· | ·· |  | ·· | ·· | ·· |  |
| Guyana | Unknown | ·· |  | 7 | ·· | ·· |  | ·· | ·· | ·· |  | ·· | ·· | ·· |  |
| Honduras | Unknown | ·· |  | 26 | ·· | ·· |  | ·· | ·· | ·· |  | ·· | ·· | ·· |  |
| Mexico | Yes | 2024 | 25 | 279 | ·· | ·· |  | ·· | ·· | ·· |  | ·· | ·· | ·· |  |
| Nicaragua | Unknown | ·· |  | 9 | ·· | ·· |  | ·· | ·· | ·· |  | ·· | ·· | ·· |  |
| Panama | Unknown | ·· |  | 23 | ·· | ·· |  | ·· | ·· | ·· |  | ·· | ·· | ·· |  |
| Paraguay | Unknown | ·· |  | 18 | ·· | ·· |  | ·· | ·· | ·· |  | ·· | ·· | ·· |  |
| Peru | Unknown | ·· |  | 68 | ·· | ·· |  | ·· | ·· | ·· |  | ·· | ·· | ·· |  |
| Suriname | Unknown | ·· |  | 21 | ·· | ·· |  | ·· | ·· | ·· |  | ·· | ·· | ·· |  |
| Uruguay | Unknown | ·· |  | 27 | ·· | ·· |  | ·· | ·· | ·· |  | ·· | ·· | ·· |  |
| Bolivarian Republic of Venezuela | Unknown | ·· |  | 41 | ·· | ·· |  | ·· | ·· | ·· |  | ·· | ·· | ·· |  |
| **North America** |  |  |  |  |  |  |  |  |  |  |  |  |  |  |  |
| Canada (Federal) | All facilities | 2008 | 26 | 43 | 43 | 2008 | 26 | 5644 | Snapshot | 2008 | 26 | ·· | ·· | ·· |  |
| Canada (Provincial/Territorial) | Yes | 2020 | 27 | 173 | 56 | 2020 | 27 | ·· | ·· | ·· |  | ·· | ·· | ·· |  |
| United States of America (Federal) | Unknown | ·· |  | 102 | ·· | ·· |  | ·· | ·· | ·· |  | ·· | ·· | ·· |  |
| United States of America (State) | Yes | 2015 | 28 | 1190 | ·· | ·· |  | ·· | ·· | ·· |  | ·· | ·· | ·· |  |
| **Pacific Island States & Terr.** |  |  |  |  |  |  |  |  |  |  |  |  |  |  |  |
| American Samoa | Unknown | ·· |  | 1 | ·· | ·· |  | ·· | ·· | ·· |  | ·· | ·· | ·· |  |
| Federated States of Micronesia | Unknown | ·· |  | 4 | ·· | ·· |  | ·· | ·· | ·· |  | ·· | ·· | ·· |  |
| Fiji | Unknown | ·· |  | 15 | ·· | ·· |  | ·· | ·· | ·· |  | ·· | ·· | ·· |  |
| French Polynesia | Unknown | ·· |  | 2 | ·· | ·· |  | ·· | ·· | ·· |  | ·· | ·· | ·· |  |
| Guam | Unknown | ·· |  | 2 | ·· | ·· |  | ·· | ·· | ·· |  | ·· | ·· | ·· |  |
| Kiribati | Unknown | ·· |  | 4 | ·· | ·· |  | ·· | ·· | ·· |  | ·· | ·· | ·· |  |
| Marshall Islands | Unknown | ·· |  | 2 | ·· | ·· |  | ·· | ·· | ·· |  | ·· | ·· | ·· |  |
| Nauru | Unknown | ·· |  | 1 | ·· | ·· |  | ·· | ·· | ·· |  | ·· | ·· | ·· |  |
| New Caledonia | Unknown | ·· |  | 1 | ·· | ·· |  | ·· | ·· | ·· |  | ·· | ·· | ·· |  |
| Northern Mariana Islands | Unknown | ·· |  | 1 | ·· | ·· |  | ·· | ·· | ·· |  | ·· | ·· | ·· |  |
| Palau | Unknown | ·· |  | 1 | ·· | ·· |  | ·· | ·· | ·· |  | ·· | ·· | ·· |  |
| Papua New Guinea | Unknown | ·· |  | 18 | ·· | ·· |  | ·· | ·· | ·· |  | ·· | ·· | ·· |  |
| Samoa | Unknown | ·· |  | 3 | ·· | ·· |  | ·· | ·· | ·· |  | ·· | ·· | ·· |  |
| Solomon Islands | Unknown | ·· |  | 6 | ·· | ·· |  | ·· | ·· | ·· |  | ·· | ·· | ·· |  |
| Tonga | Unknown | ·· |  | 4 | ·· | ·· |  | ·· | ·· | ·· |  | ·· | ·· | ·· |  |
| Tuvalu | Unknown | ·· |  | 2 | ·· | ·· |  | ·· | ·· | ·· |  | ·· | ·· | ·· |  |
| Vanuatu | Unknown | ·· |  | 4 | ·· | ·· |  | ·· | ·· | ·· |  | ·· | ·· | ·· |  |
| **Australasia** |  |  |  |  |  |  |  |  |  |  |  |  |  |  |  |
| Australia | Yes | 2022 | 29 | 111 | 101 | 2022 | 29 | ·· | ·· | ·· |  | 28201 | Past year | 2022 | 29 |
| New Zealand | Unknown | ·· |  | 18 | ·· | ·· |  | ·· | ·· | ·· |  | ·· | ·· | ·· |  |
| **Sub Saharan Africa** |  |  |  |  |  |  |  |  |  |  |  |  |  |  |  |
| Angola | Unknown | ·· |  | 47 | ·· | ·· |  | ·· | ·· | ·· |  | ·· | ·· | ·· |  |
| Benin | Unknown | ·· |  | 11 | ·· | ·· |  | ·· | ·· | ·· |  | ·· | ·· | ·· |  |
| Botswana | Unknown | ·· |  | 23 | ·· | ·· |  | ·· | ·· | ·· |  | ·· | ·· | ·· |  |
| Burkina Faso | Unknown | ·· |  | 27 | ·· | ·· |  | ·· | ·· | ·· |  | ·· | ·· | ·· |  |
| Burundi | Unknown | ·· |  | 13 | ·· | ·· |  | ·· | ·· | ·· |  | ·· | ·· | ·· |  |
| Cameroon | Yes | 2021 | 30 | 76 | ·· | ·· |  | ·· | ·· | ·· |  | ·· | ·· | ·· |  |
| Cabo Verde | Unknown | ·· |  | 5 | ·· | ·· |  | ·· | ·· | ·· |  | ·· | ·· | ·· |  |
| Central African Republic | Unknown | ·· |  | 15 | ·· | ·· |  | ·· | ·· | ·· |  | ·· | ·· | ·· |  |
| Chad | Unknown | ·· |  | 41 | ·· | ·· |  | ·· | ·· | ·· |  | ·· | ·· | ·· |  |
| Comoros | Unknown | ·· |  | 3 | ·· | ·· |  | ·· | ·· | ·· |  | ·· | ·· | ·· |  |
| Côte d'Ivoire | Unknown | ·· |  | 34 | ·· | ·· |  | ·· | ·· | ·· |  | ·· | ·· | ·· |  |
| Democratic Republic of the Congo | Unknown | ·· |  | 142 | ·· | ·· |  | ·· | ·· | ·· |  | ·· | ·· | ·· |  |
| Djibouti | Unknown | ·· |  | 1 | ·· | ·· |  | ·· | ·· | ·· |  | ·· | ·· | ·· |  |
| Equatorial Guinea | Unknown | ·· |  | 15 | ·· | ·· |  | ·· | ·· | ·· |  | ·· | ·· | ·· |  |
| Eritrea | Unknown | ·· |  | N/A | ·· | ·· |  | ·· | ·· | ·· |  | ·· | ·· | ·· |  |
| Eswatini | Unknown | ·· |  | 12 | ·· | ·· |  | ·· | ·· | ·· |  | ·· | ·· | ·· |  |
| Ethiopia | Unknown | ·· |  | 126 | ·· | ·· |  | ·· | ·· | ·· |  | ·· | ·· | ·· |  |
| Gabon | Unknown | ·· |  | 9 | ·· | ·· |  | ·· | ·· | ·· |  | ·· | ·· | ·· |  |
| Gambia | Unknown | ·· |  | 3 | ·· | ·· |  | ·· | ·· | ·· |  | ·· | ·· | ·· |  |
| Ghana | Unknown | ·· |  | 46 | ·· | ·· |  | ·· | ·· | ·· |  | ·· | ·· | ·· |  |
| Guinea | Unknown | ·· |  | 31 | ·· | ·· |  | ·· | ·· | ·· |  | ·· | ·· | ·· |  |
| Guinea-Bissau | Unknown | ·· |  | 3 | ·· | ·· |  | ·· | ·· | ·· |  | ·· | ·· | ·· |  |
| Kenya | Unknown | ·· |  | 134 | ·· | ·· |  | ·· | ·· | ·· |  | ·· | ·· | ·· |  |
| Lesotho | Unknown | ·· |  | 14 | ·· | ·· |  | ·· | ·· | ·· |  | ·· | ·· | ·· |  |
| Liberia | Unknown | ·· |  | 16 | ·· | ·· |  | ·· | ·· | ·· |  | ·· | ·· | ·· |  |
| Madagascar | Unknown | ·· |  | 82 | ·· | ·· |  | ·· | ·· | ·· |  | ·· | ·· | ·· |  |
| Malawi | Unknown | ·· |  | 30 | ·· | ·· |  | ·· | ·· | ·· |  | ·· | ·· | ·· |  |
| Mali | Unknown | ·· |  | 60 | ·· | ·· |  | ·· | ·· | ·· |  | ·· | ·· | ·· |  |
| Mauritania | Unknown | ·· |  | 23 | ·· | ·· |  | ·· | ·· | ·· |  | ·· | ·· | ·· |  |
| Mauritius | Unknown | ·· |  | 11 | ·· | ·· |  | ·· | ·· | ·· |  | ·· | ·· | ·· |  |
| Mozambique | Unknown | ·· |  | 157 | ·· | ·· |  | ·· | ·· | ·· |  | ·· | ·· | ·· |  |
| Namibia | Unknown | ·· |  | 13 | ·· | ·· |  | ·· | ·· | ·· |  | ·· | ·· | ·· |  |
| Niger | Unknown | ·· |  | 41 | ·· | ·· |  | ·· | ·· | ·· |  | ·· | ·· | ·· |  |
| Nigeria | Unknown | ·· |  | 240 | ·· | ·· |  | ·· | ·· | ·· |  | ·· | ·· | ·· |  |
| Congo | Unknown | ·· |  | 12 | ·· | ·· |  | ·· | ·· | ·· |  | ·· | ·· | ·· |  |
| Rwanda | Unknown | ·· |  | 13 | ·· | ·· |  | ·· | ·· | ·· |  | ·· | ·· | ·· |  |
| Sao Tome & Principe | Unknown | ·· |  | 1 | ·· | ·· |  | ·· | ·· | ·· |  | ·· | ·· | ·· |  |
| Senegal | Unknown | ·· |  | 37 | ·· | ·· |  | ·· | ·· | ·· |  | ·· | ·· | ·· |  |
| Seychelles | Unknown | ·· |  | 3 | ·· | ·· |  | ·· | ·· | ·· |  | ·· | ·· | ·· |  |
| Sierra Leone | Unknown | ·· |  | 21 | ·· | ·· |  | ·· | ·· | ·· |  | ·· | ·· | ·· |  |
| Somalia | Unknown | ·· |  | 14 | ·· | ·· |  | ·· | ·· | ·· |  | ·· | ·· | ·· |  |
| South Africa | Unknown | ·· |  | 235 | ·· | ·· |  | ·· | ·· | ·· |  | ·· | ·· | ·· |  |
| United Republic of Tanzania | Unknown | ·· |  | 126 | ·· | ·· |  | ·· | ·· | ·· |  | ·· | ·· | ·· |  |
| Togo | Unknown | ·· |  | 13 | ·· | ·· |  | ·· | ·· | ·· |  | ·· | ·· | ·· |  |
| Uganda | Unknown | ·· |  | 254 | ·· | ·· |  | ·· | ·· | ·· |  | ·· | ·· | ·· |  |
| Zambia | Unknown | ·· |  | 90 | ·· | ·· |  | ·· | ·· | ·· |  | ·· | ·· | ·· |  |
| Zimbabwe | Unknown | ·· |  | 72 | ·· | ·· |  | ·· | ·· | ·· |  | ·· | ·· | ·· |  |
| **Middle East & North Africa** |  |  |  |  |  |  |  |  |  |  |  |  |  |  |  |
| Algeria | Unknown | ·· |  | 162 | ·· | ·· |  | ·· | ·· | ·· |  | ·· | ·· | ·· |  |
| Bahrain | Unknown | ·· |  | 4 | ·· | ·· |  | ·· | ·· | ·· |  | ·· | ·· | ·· |  |
| Cyprus | All facilities | 2020 | 1 | 1 | 1 | 2020 | 1 | ·· | ·· | ·· |  | ·· | ·· | ·· |  |
| Egypt | Yes | 2019 | 31 | 78 | ·· | ·· |  | ·· | ·· | ·· |  | ·· | ·· | ·· |  |
| Iraq | Unknown | ·· |  | 55 | ·· | ·· |  | ·· | ·· | ·· |  | ·· | ·· | ·· |  |
| Israel | Unknown | ·· |  | 30 | ·· | ·· |  | ·· | ·· | ·· |  | ·· | ·· | ·· |  |
| Jordan | Unknown | ·· |  | 17 | ·· | ·· |  | ·· | ·· | ·· |  | ·· | ·· | ·· |  |
| Kuwait | All facilities | 2020 | 32 | 3 | 3 | 2020 | 32 | ·· | ·· | ·· |  | ·· | ·· | ·· |  |
| Lebanon | Unknown | ·· |  | 25 | ·· | ·· |  | ·· | ·· | ·· |  | ·· | ·· | ·· |  |
| Libya | Unknown | ·· |  | 26 | ·· | ·· |  | ·· | ·· | ·· |  | ·· | ·· | ·· |  |
| Morocco | Unknown | ·· |  | 75 | ·· | ·· |  | ·· | ·· | ·· |  | ·· | ·· | ·· |  |
| Oman | Unknown | ·· |  | 3 | ·· | ·· |  | ·· | ·· | ·· |  | ·· | ·· | ·· |  |
| Occupied Palestinian territories | Unknown | ·· |  | N/A | ·· | ·· |  | ·· | ·· | ·· |  | ·· | ·· | ·· |  |
| Qatar | Unknown | ·· |  | 1 | ·· | ·· |  | ·· | ·· | ·· |  | ·· | ·· | ·· |  |
| Saudi Arabia | Unknown | ·· |  | 110 | ·· | ·· |  | ·· | ·· | ·· |  | ·· | ·· | ·· |  |
| South Sudan | Unknown | ·· |  | 80 | ·· | ·· |  | ·· | ·· | ·· |  | ·· | ·· | ·· |  |
| Sudan | Unknown | ·· |  | 125 | ·· | ·· |  | ·· | ·· | ·· |  | ·· | ·· | ·· |  |
| Syrian Arab Republic | Unknown | ·· |  | 35 | ·· | ·· |  | ·· | ·· | ·· |  | ·· | ·· | ·· |  |
| Tunisia | Unknown | ·· |  | 32 | ·· | ·· |  | ·· | ·· | ·· |  | ·· | ·· | ·· |  |
| Türkiye | Yes | 2022 | 2 | 403 | ·· | ·· |  | ·· | ·· | ·· |  | ·· | ·· | ·· |  |
| United Arab Emirates | Unknown | ·· |  | 21 | ·· | ·· |  | ·· | ·· | ·· |  | ·· | ·· | ·· |  |
| Yemen | Unknown | ·· |  | 43 | ·· | ·· |  | ·· | ·· | ·· |  | ·· | ·· | ·· |  |

**Notes:**

**Ref:** Reference; **HCV**: Hepatitis C.

***Total sites:** Including prisons, jails or other carceral setting, Country level data that informed these regional and global estimates were sourced from the World Prison Brief, collated by the Institute for Crime and Justice Policy Research at Burbeck University. See: <https://www.prisonstudies.org/world-prison-brief-data>;

** The total number of sites for England and Wales was reported together in the World Prison Brief, therefore the total number of sites for both England and Wales is reported as 120.

† For reporting purposes, these countries or territories are reported separately due to differences in service provision.

*** Pilot data only

·· Indicates there were no data to inform a country’s estimate.

**References for Table 6.5**

1. World Health Organization. Status report on prison health in the WHO European Region 2022. Copenhagen, 2023.

2. European Union Drug Agency. EUDA Bulletin - Interventions in Prisons, Europe. 2024.

3. Kanev KA, D. The Reform of The Bulgarian Penitentiary System: Current State and Future Prospects. Sofia, 2024.

4. Harris AM, Chokoshvili O, Biddle J, et al. An evaluation of the hepatitis C testing, care and treatment program in the country of Georgia's corrections system, December 2013 - April 2015. *BMC Public Health* 2019.

5. Werling K, Hunyady B, Makara M, et al. Hepatitis C Screening and Treatment Program in Hungarian Prisons in the Era of Direct Acting Antiviral Agents. *Viruses* 2022.

6. The International Network on Health and Hepatitis in Substance Users: Prisons. HCV Testing & Treatment for All Citizens - Prisons, Moldova. 2024.

7. Harm Reduction International. Availability, accessibility, acceptability and quality of harm reduction services in Moldovan prisons. London, 2021.

8. Andreić J-L. Prison Workbook 2023 - Croatia, 2023.

9. The International Network on Health and Hepatitis in Substance Users: Prisons. Working Together to Eliminate HCV - Prisons, England. 2024.

10. UK Health Security Agency. Taking a place-based approach to tackling hepatitis in prisons. 2023.

11. Remy AJ, Roy B, Hervet J, Bouchkira H. Lack of COVID-19 impact on managing hepatitis c in prison like the general population! *Hepatology* 2021.

12. Fridriksdottir R, Ingibergsdotir, B, Frodjonsdottir, H, Alexiusdottir, K, Tomasdottir, A, Bjornsdottir, Th, Finnbogsdottir, A, Olafsdottir, B, Tyrfingsson, Th, Runarsdottir, V, Bergmann, OM, Bjornsson, ES, Johansson, B, Sigurdardottir, B, Heimisdottir, M, Olafsson, S & Gottfredsson, M. Sustained reduction in prevalence of hepatitis C viremia in the prison setting after 3rd year of TrapHepC (Treatment as Prevention for hepatitis C) program in Iceland. 2020.

13. Kugener T, Berndt N, Origer A, Debacker M. Prison workbook 2023 Luxembourg. 2023.

14. de Castro MV, Mendes SCC, Marques JPA, Santos AS, Duarte O. Prison Workbook 2023 - Portugal. 2023.

15. Coalition for Global Hepatitis Elimination and INHSU Prisons. Expanding access to hepatitis C prevention, testing, and treatment in prisons: Recommendations from the INHSU 2022 Prisons Workshop. Atlanta, United States, 2023.

16. McLeod A, Glancy, M, Went, A Smith, S, Weir, A, McAuley, A, Hutchinson, S & Goldberg, D. Surveillance report. Surveillance of hepatitis C testing, diagnosis and treatment in Scotland, 2019 update. Health Protection Scotland, 2019.

17. Kilibarda B, Simić D, Baroš S, Brandić I. National Report on Drug Situation in Serbia, 2014, 2014.

18. Thomas C, Widiastuti H. HCV microelimination project in 7 Jakarta prison. *Hepatol Int* 2020.

19. Yu ML. Elimination policy in special population ''NO PATIENT LEFT BEHIND'' prisoners test & treatment. *Hepatol Int* 2023; **17(Supplement 1)**: S56.

20. National AIDS Control Organisation. Sankalak: Status of National AIDS & STD Response (Fifth edition, 2023). New Delhi: NACO, Ministry of Health and Family Welfare, Government of India., 2023.

21. Hariri S, Alavi M, Roshandel G, et al. An intervention to increase hepatitis C virus diagnosis and treatment uptake among people in custody in Iran. *Int J Drug Policy* 2021.

22. Maldives Correctional Service. Healthcare in Prison. 2017. https://en.corrections.gov.mv/2017/09/21/healthcare/.

23. Saude Md. Plano Nacional de Saude no Sistema Penitenciario, 2003.

24. Hincapie C. In: Degenhardt L, editor.; 2024.

25. Borquez A. Coverage data in Mexico prisons. In: Thomas Santo Jr., editor.; 2024.

26. Correctional Service of Canada. Infectious disease surveillance in Canadian federal penitentiaries 2007–2008: pre-release report. Correctional Service of Canada Ottawa, ON; 2012.

27. Kronfli N, Dussault C, Bartlett S, et al. Disparities in hepatitis C care across Canadian provincial prisons: Implications for hepatitis C micro-elimination. *Can Liver J* 2021.

28. Beckman AL, Bilinski A, Boyko R, et al. New Hepatitis C Drugs Are Very Costly And Unavailable To Many State Prisoners. *Health Aff (Millwood)* 2016.

29. Burnet Institute and Kirby Institute. Australia’s progress towards hepatitis C elimination: annual report 2023. Melbourne: Burnet Institute, 2023.

30. Noeske J, Ndi NF, Nga FHM, Mely G, Kuaban C. Prison health services across ten central prisons in Cameroon. *J Public Health Afr* 2023; **14**(9).

31. United Nations Office on Drugs and Crime ROMENA. Egypt: UNODC inaugurates first-ever prison Voluntary Confidential Counseling, Testing and aftercare services. 2019.

32. Hasan F, Alfadhli A, Al-Gharabally A, Alkhaldi M, Colombo M, Lazarus JV. Accelerating the elimination of hepatitis C in Kuwait: An expert opinion. *World J Gastroenterol* 2020; **26**(30): 4415.

## *Appendix Table 6.6*: Country-level availability of interventions and programmatic data for HCV Treatment (DAAs)

|  | | | | **Number of Carceral Sites with HCV Treatment (DAAs)** | | | | **Number of People Accessing HCV Treatment (DAAs) in Carceral Settings** | | | |
| --- | --- | --- | --- | --- | --- | --- | --- | --- | --- | --- | --- |
| **Country** | **Available** | **Year** | **Ref** | **Total sites*** | **N Sites with HCV Treatment** | **Year** | **Ref** | **N People Accessing HCV Treatment in Carceral Settings** | **Timeframe** | **Year** | **Ref** |
| **Eastern Europe** |  |  |  |  |  |  |  |  |  |  |  |
| Armenia | Yes | 2020 | 1 | 12 | ·· | ·· | ·· | 1 | Past year | 2020 | 1 |
| Azerbaijan | Unknown | ·· |  | 53 | ·· | ·· | ·· | ·· | ·· | ·· |  |
| Belarus | Unknown | ·· |  | 67 | ·· | ·· | ·· | ·· | ·· | ·· |  |
| Bosnia and Herzegovina | Yes | 2020 | 1 | 13 | ·· | ·· | ·· | 13 | Past year | 2020 | 1 |
| Bulgaria | No | 2022 | 2 | 55 | ·· | ·· | ·· | ·· | ·· | ·· |  |
| Czechia | Yes | 2022 | 2 | 35 | ·· | ·· | ·· | 213 | Past year | 2020 | 1 |
| Estonia | All facilities | 2022 | 2 | 3 | 3 | 2022 | 2 | 136 | Past year | 2020 | 1 |
| Georgia | Yes | 2020 | 1 | 14 | ·· | ·· | ·· | 320 | Past year | 2020 | 1 |
| Hungary | Yes | 2022 | 2 | 30 | 16 | 2022 | 2 | 64 | Past year | 2020 | 1 |
| Latvia | Yes | 2022 | 2 | 9 | 8 | 2022 | 2 | 442 | Snapshot | 2020 | 1 |
| Lithuania | Yes | 2022 | 2 | 8 | 2 | 2022 | 2 | 23 | Past year | 2020 | 1 |
| Republic of Moldova | All facilities | 2024 | 3 | 17 | 17 | 2024 | 3 | 66 | Past year | 2020 | 1 |
| Poland | Yes | 2022 | 2 | 215 | 1 | 2022 | 2 | ·· | ·· | ·· |  |
| Romania | Yes | 2022 | 2 | 45 | ·· | ·· | ·· | ·· | ·· | ·· |  |
| Russian Federation | Unknown | ·· |  | 872 | ·· | ·· | ·· | ·· | ·· | ·· |  |
| Slovakia | All facilities | 2022 | 2 | 18 | 18 | 2022 | 2 | 289 | Past year | 2020 | 1 |
| Ukraine | Yes | 2020 | 1 | 110 | ·· | ·· | ·· | 2145 | Past year | 2020 | 1 |
| **Western Europe** |  |  |  |  |  |  |  |  | ·· |  |  |
| Albania | Yes | 2020 | 1 | 23 | ·· | ·· | ·· | 2 | Past year | 2020 | 1 |
| Andorra | Unknown | ·· |  | 1 | ·· | ·· | ·· | ·· | ·· | ·· |  |
| Austria | All facilities | 2022 | 2 | 27 | 27 | 2022 | 2 | 64 | Past year | 2020 | 1 |
| Belgium | Yes | 2022 | 2 | 35 | ·· | ·· | ·· | 54 | Snapshot | 2020 | 1 |
| Croatia | All facilities | 2022 | 4 | 24 | 24 | 2022 | 4 | 17 | Past year | 2022 | 4 |
| Denmark | No | 2022 | 2 | 54 | ·· | ·· | ·· | ·· | ·· | ·· |  |
| England** | All facilities | 2024 | 5 | 120 | 120 | 2024 | 5 | ·· | ·· | ·· |  |
| Finland | All facilities | 2022 | 2 | 26 | 26 | 2022 | 2 | ·· | ·· | ·· |  |
| France | Yes | 2022 | 2 | 187 | 37 | 2022 | 2 | 173 | Snapshot | 2018 | 6 |
| Germany | Yes | 2022 | 2 | 179 | ·· | ·· | ·· | 31 | Past year | 2020 | 1 |
| Greece | Yes | 2020 | 1 | 34 | ·· | ·· | ·· | 0 | Past year | 2020 | 1 |
| Greenland | Unknown | ·· |  | 6 | ·· | ·· | ·· | ·· | ·· | ·· |  |
| Iceland | Unknown | ·· |  | 5 | ·· | ·· | ·· | ·· | ·· | ·· |  |
| Ireland | Yes | 2021 | 2 | 12 | ·· | ·· | ·· | 55 | Snapshot | 2020 | 1 |
| Italy | Yes | 2022 | 2 | 206 | ·· | ·· | ·· | ·· | ·· | ·· |  |
| Liechtenstein | Unknown | ·· |  | 1 | ·· | ·· | ·· | ·· | ·· | ·· |  |
| Luxembourg | All facilities | 2022 | 7 | 3 | 3 | 2022 | 7 | 23 | Snapshot | 2020 | 1 |
| Malta | Yes | 2022 | 2 | 1 | ·· | ·· | ·· | 37 | Past year | 2020 | 1 |
| Monaco | Unknown | ·· |  | 2 | ·· | ·· | ·· | ·· | ·· | ·· |  |
| Montenegro | Unknown | ·· |  | 3 | ·· | ·· | ·· | ·· | ·· | ·· |  |
| Netherlands | Yes | 2022 | 2 | 49 | ·· | ·· | ·· | ·· | ·· | ·· |  |
| North Macedonia | No | 2017 | 8 | 13 | ·· | ·· | ·· | ·· | ·· | ·· |  |
| Northern Ireland | All facilities | 2018 | 8 | 3 | 3 | 2018 | 8 | ·· | ·· | ·· |  |
| Norway | Yes | 2022 | 2 | 33 | ·· | ·· | ·· | ·· | ·· | ·· |  |
| Portugal | All facilities | 2022 | 9 | 49 | 49 | 2022 | 9 | 81 | Past year | 2020 | 1 |
| San Marino | Unknown | ·· |  | 1 | ·· | ·· | ·· | ·· | ·· | ·· |  |
| Scotland | All facilities | 2022 | 10 | 15 | 15 | 2022 | 10 | 313 | Snapshot | 2019 | 11 |
| Serbia | Yes | 2017 | 8 | 30 | ·· | ·· | ·· | ·· | ·· | ·· |  |
| Slovenia | All facilities | 2022 | 2 | 7 | 7 | 2022 | 2 | 37 | Past year | 2020 | 1 |
| Spain | All facilities | 2022 | 2 | 82 | 82 | 2022 | 2 | 489 | ·· | 2020 | 1 |
| Sweden | All facilities | 2022 | 2 | 79 | 79 | 2022 | 2 | ·· | ·· | ·· |  |
| Switzerland | Yes | 2004 | 12 | 88 | 34 | 2004 | 12 | ·· | ·· | ·· |  |
| Wales** | All facilities | 2018 | 8 | 120 | 120 | 2018 | 8 | ·· | ·· | ·· |  |
| **East and South East Asia** |  |  |  |  |  |  |  |  | ·· |  |  |
| Brunei Darussalam | Unknown | ·· |  | 3 | ·· | ·· | ·· | ·· | ·· | ·· |  |
| Cambodia | Unknown | ·· |  | 29 | ·· | ·· | ·· | ·· | ·· | ·· |  |
| China | Unknown | ·· |  | 683 | ·· | ·· | ·· | ·· | ·· | ·· |  |
| Hong Kong† | Unknown | ·· |  | 24 | ·· | ·· | ·· | ·· | ·· | ·· |  |
| Indonesia | Unknown*** | 2019 | 13 | 526 | ·· | ·· | ·· | ·· | ·· | ·· |  |
| Japan | Unknown | ·· |  | 184 | ·· | ·· | ·· | ·· | ·· | ·· |  |
| Lao People's Democratic Republic | Unknown | ·· |  | 19 | ·· | ·· | ·· | ·· | ·· | ·· |  |
| Malaysia | Unknown | ·· |  | 43 | ·· | ·· | ·· | ·· | ·· | ·· |  |
| Mongolia | Unknown | ·· |  | 50 | ·· | ·· | ·· | ·· | ·· | ·· |  |
| Myanmar | Unknown | ·· |  | 96 | ·· | ·· | ·· | ·· | ·· | ·· |  |
| Democratic People's Republic of Korea | Unknown | ·· |  | N/A | ·· | ·· | ·· | ·· | ·· | ·· |  |
| Philippines | Unknown | ·· |  | 440 | ·· | ·· | ·· | ·· | ·· | ·· |  |
| Singapore | Unknown | ·· |  | 13 | ·· | ·· | ·· | ·· | ·· | ·· |  |
| Republic of Korea | Unknown | ·· |  | 54 | ·· | ·· | ·· | ·· | ·· | ·· |  |
| Taiwan† | Unknown | ·· |  | 49 | ·· | ·· | ·· | ·· | ·· | ·· |  |
| Thailand | No | 2019 | 14 | 143 | ·· | ·· | ·· | ·· | ·· | ·· |  |
| Timor-Leste | Unknown | ·· |  | 3 | ·· | ·· | ·· | ·· | ·· | ·· |  |
| Viet Nam | Unknown | ·· |  | 54 | ·· | ·· | ·· | ·· | ·· | ·· |  |
| **South Asia** |  |  |  |  |  |  |  |  |  |  |  |
| Afghanistan | Unknown | ·· |  | 251 | ·· | ·· | ·· | ·· | ·· | ·· |  |
| Bangladesh | Unknown | ·· |  | 68 | ·· | ·· | ·· | ·· | ·· | ·· |  |
| Bhutan | Unknown | ·· |  | 7 | ·· | ·· | ·· | ·· | ·· | ·· |  |
| India | Yes | 2020 | 15 | 1330 | ·· | ·· | ·· | ·· | ·· | ·· |  |
| Islamic Republic of Iran | Unknown*** | 2018 | 16 | 253 | ·· | ·· | ·· | ·· | ·· | ·· |  |
| Maldives | Unknown | ·· |  | 11 | ·· | ·· | ·· | ·· | ·· | ·· |  |
| Nepal | Unknown | ·· |  | 74 | ·· | ·· | ·· | ·· | ·· | ·· |  |
| Pakistan | Unknown | ·· |  | 116 | ·· | ·· | ·· | ·· | ·· | ·· |  |
| Sri Lanka | Unknown | ·· |  | 60 | ·· | ·· | ·· | ·· | ·· | ·· |  |
| **Central Asia** |  |  |  |  |  |  |  |  | ·· |  |  |
| Kazakhstan | Unknown | ·· |  | 80 | ·· | ·· | ·· | ·· | ·· | ·· |  |
| Kyrgyzstan | Unknown | ·· |  | 28 | ·· | ·· | ·· | ·· | ·· | ·· |  |
| Tajikistan | Unknown | ·· |  | 19 | ·· | ·· | ·· | ·· | ·· | ·· |  |
| Turkmenistan | Unknown | ·· |  | 27 | ·· | ·· | ·· | ·· | ·· | ·· |  |
| Uzbekistan | Unknown | ·· |  | 54 | ·· | ·· | ·· | ·· | ·· | ·· |  |
| **Caribbean** |  |  |  |  |  |  |  |  | ·· |  |  |
| Antigua & Barbuda | Unknown | ·· |  | 1 | ·· | ·· | ·· | ·· | ·· | ·· |  |
| Bahamas | Unknown | ·· |  | 1 | ·· | ·· | ·· | ·· | ·· | ·· |  |
| Barbados | Unknown | ·· |  | 1 | ·· | ·· | ·· | ·· | ·· | ·· |  |
| Bermuda | Unknown | ·· |  | 4 | ·· | ·· | ·· | ·· | ·· | ·· |  |
| Cuba | Unknown | ·· |  | 200 | ·· | ·· | ·· | ·· | ·· | ·· |  |
| Dominica | Unknown | ·· |  | 1 | ·· | ·· | ·· | ·· | ·· | ·· |  |
| Dominican Republic | Unknown | ·· |  | 41 | ·· | ·· | ·· | ·· | ·· | ·· |  |
| Grenada | Unknown | ·· |  | 1 | ·· | ·· | ·· | ·· | ·· | ·· |  |
| Haiti | Unknown | ·· |  | 17 | ·· | ·· | ·· | ·· | ·· | ·· |  |
| Jamaica | Unknown | ·· |  | 11 | ·· | ·· | ·· | ·· | ·· | ·· |  |
| Commonwealth of Puerto Rico | Unknown | ·· |  | 34 | ·· | ·· | ·· | ·· | ·· | ·· |  |
| Saint Kitts & Nevis | Unknown | ·· |  | 2 | ·· | ·· | ·· | ·· | ·· | ·· |  |
| Saint Lucia | Unknown | ·· |  | 1 | ·· | ·· | ·· | ·· | ·· | ·· |  |
| Saint Vincent & the Grenadines | No | 2023 | 17 | 2 | ·· | ·· | ·· | ·· | ·· | ·· |  |
| Trinidad & Tobago | No | 2023 | 17 | 9 | ·· | ·· | ·· | ·· | ·· | ·· |  |
| **Latin America** |  |  |  |  |  |  |  |  | ·· |  |  |
| Argentina | Unknown | ·· |  | 338 | ·· | ·· | ·· | ·· | ·· | ·· |  |
| Belize | Unknown | ·· |  | 1 | ·· | ·· | ·· | ·· | ·· | ·· |  |
| Plurinational State of Bolivia | Unknown | ·· |  | 40 | ·· | ·· | ·· | ·· | ·· | ·· |  |
| Brazil | Unknown | ·· |  | 1386 | ·· | ·· | ·· | ·· | ·· | ·· |  |
| Chile | Unknown | ·· |  | 113 | ·· | ·· | ·· | ·· | ·· | ·· |  |
| Colombia | Yes | 2024 | 18 | 125 | ·· | ·· | ·· | ·· | ·· | ·· |  |
| Costa Rica | Unknown | ·· |  | 32 | ·· | ·· | ·· | ·· | ·· | ·· |  |
| Ecuador | Unknown | ·· |  | 52 | ·· | ·· | ·· | ·· | ·· | ·· |  |
| El Salvador | Unknown | ·· |  | 25 | ·· | ·· | ·· | ·· | ·· | ·· |  |
| Guatemala | Unknown | ·· |  | 21 | ·· | ·· | ·· | ·· | ·· | ·· |  |
| Guyana | Unknown | ·· |  | 7 | ·· | ·· | ·· | ·· | ·· | ·· |  |
| Honduras | Unknown | ·· |  | 26 | ·· | ·· | ·· | ·· | ·· | ·· |  |
| Mexico | Yes | 2024 | 19 | 279 | ·· | ·· | ·· | ·· | ·· | ·· |  |
| Nicaragua | Unknown | ·· |  | 9 | ·· | ·· | ·· | ·· | ·· | ·· |  |
| Panama | Unknown | ·· |  | 23 | ·· | ·· | ·· | ·· | ·· | ·· |  |
| Paraguay | Unknown | ·· |  | 18 | ·· | ·· | ·· | ·· | ·· | ·· |  |
| Peru | Unknown | ·· |  | 68 | ·· | ·· | ·· | ·· | ·· | ·· |  |
| Suriname | Unknown | ·· |  | 21 | ·· | ·· | ·· | ·· | ·· | ·· |  |
| Uruguay | Unknown | ·· |  | 27 | ·· | ·· | ·· | ·· | ·· | ·· |  |
| Bolivarian Republic of Venezuela | Unknown | ·· |  | 41 | ·· | ·· | ·· | ·· | ·· | ·· |  |
| **North America** |  |  |  |  |  |  |  |  | ·· |  |  |
| Canada (Federal) | All facilities | 2008 | 20 | 43 | 43 | 2008 | 20 | 319 | Snapshot | 2008 | 20 |
| Canada (Provincial/Territorial) | Yes | 2020 | 21 | 173 | 32 | 2020 | 21 | ·· | ·· | ·· |  |
| United States of America (Federal) | Unknown | ·· |  | 102 | ·· | ·· | ·· | ·· | ·· | ·· |  |
| United States of America (State) | Yes | 2015 | 22 | 1190 | ·· | ·· | ·· | 949 | Snapshot | 2015 | 22 |
| **Pacific Island States & Terr.** |  |  |  |  |  |  |  |  | ·· |  |  |
| American Samoa | Unknown | ·· |  | 1 | ·· | ·· | ·· | ·· | ·· | ·· |  |
| Federated States of Micronesia | Unknown | ·· |  | 4 | ·· | ·· | ·· | ·· | ·· | ·· |  |
| Fiji | No | 2023 | 17 | 15 | ·· | ·· | ·· | ·· | ·· | ·· |  |
| French Polynesia | Unknown | ·· |  | 2 | ·· | ·· | ·· | ·· | ·· | ·· |  |
| Guam | Unknown | ·· |  | 2 | ·· | ·· | ·· | ·· | ·· | ·· |  |
| Kiribati | Unknown | ·· |  | 4 | ·· | ·· | ·· | ·· | ·· | ·· |  |
| Marshall Islands | Unknown | ·· |  | 2 | ·· | ·· | ·· | ·· | ·· | ·· |  |
| Nauru | Unknown | ·· |  | 1 | ·· | ·· | ·· | ·· | ·· | ·· |  |
| New Caledonia | Unknown | ·· |  | 1 | ·· | ·· | ·· | ·· | ·· | ·· |  |
| Northern Mariana Islands | Unknown | ·· |  | 1 | ·· | ·· | ·· | ·· | ·· | ·· |  |
| Palau | Unknown | ·· |  | 1 | ·· | ·· | ·· | ·· | ·· | ·· |  |
| Papua New Guinea | No | 2023 | 17 | 18 | ·· | ·· | ·· | ·· | ·· | ·· |  |
| Samoa | Unknown | ·· |  | 3 | ·· | ·· | ·· | ·· | ·· | ·· |  |
| Solomon Islands | Unknown | ·· |  | 6 | ·· | ·· | ·· | ·· | ·· | ·· |  |
| Tonga | Unknown | ·· |  | 4 | ·· | ·· | ·· | ·· | ·· | ·· |  |
| Tuvalu | Unknown | ·· |  | 2 | ·· | ·· | ·· | ·· | ·· | ·· |  |
| Vanuatu | Unknown | ·· |  | 4 | ·· | ·· | ·· | ·· | ·· | ·· |  |
| **Australasia** |  |  |  |  |  |  |  |  | ·· |  |  |
| Australia | Yes | 2022 | 23 | 111 | 102 | 2022 | 23 | 2560 | Past year | 2022 | 23 |
| New Zealand | Unknown | ·· |  | 18 | ·· | ·· | ·· | ·· | ·· | ·· |  |
| **Sub Saharan Africa** |  |  |  |  |  |  |  |  | ·· |  |  |
| Angola | No | 2023 | 17 | 47 | ·· | ·· | ·· | ·· | ·· | ·· |  |
| Benin | Unknown | ·· |  | 11 | ·· | ·· | ·· | ·· | ·· | ·· |  |
| Botswana | Unknown | ·· |  | 23 | ·· | ·· | ·· | ·· | ·· | ·· |  |
| Burkina Faso | Unknown | ·· |  | 27 | ·· | ·· | ·· | ·· | ·· | ·· |  |
| Burundi | Unknown | ·· |  | 13 | ·· | ·· | ·· | ·· | ·· | ·· |  |
| Cameroon | Unknown | ·· |  | 76 | ·· | ·· | ·· | ·· | ·· | ·· |  |
| Cabo Verde | Unknown | ·· |  | 5 | ·· | ·· | ·· | ·· | ·· | ·· |  |
| Central African Republic | Unknown | ·· |  | 15 | ·· | ·· | ·· | ·· | ·· | ·· |  |
| Chad | Unknown | ·· |  | 41 | ·· | ·· | ·· | ·· | ·· | ·· |  |
| Comoros | Unknown | ·· |  | 3 | ·· | ·· | ·· | ·· | ·· | ·· |  |
| Côte d'Ivoire | Unknown | ·· |  | 34 | ·· | ·· | ·· | ·· | ·· | ·· |  |
| Democratic Republic of the Congo | Unknown | ·· |  | 142 | ·· | ·· | ·· | ·· | ·· | ·· |  |
| Djibouti | Unknown | ·· |  | 1 | ·· | ·· | ·· | ·· | ·· | ·· |  |
| Equatorial Guinea | Unknown | ·· |  | 15 | ·· | ·· | ·· | ·· | ·· | ·· |  |
| Eritrea | Unknown | ·· |  | N/A | ·· | ·· | ·· | ·· | ·· | ·· |  |
| Eswatini | Unknown | ·· |  | 12 | ·· | ·· | ·· | ·· | ·· | ·· |  |
| Ethiopia | Unknown | ·· |  | 126 | ·· | ·· | ·· | ·· | ·· | ·· |  |
| Gabon | Unknown | ·· |  | 9 | ·· | ·· | ·· | ·· | ·· | ·· |  |
| Gambia | No | 2023 | 17 | 3 | ·· | ·· | ·· | ·· | ·· | ·· |  |
| Ghana | No | 2023 | 17 | 46 | ·· | ·· | ·· | ·· | ·· | ·· |  |
| Guinea | Unknown | ·· |  | 31 | ·· | ·· | ·· | ·· | ·· | ·· |  |
| Guinea-Bissau | Unknown | ·· |  | 3 | ·· | ·· | ·· | ·· | ·· | ·· |  |
| Kenya | Unknown | ·· |  | 134 | ·· | ·· | ·· | ·· | ·· | ·· |  |
| Lesotho | Unknown | ·· |  | 14 | ·· | ·· | ·· | ·· | ·· | ·· |  |
| Liberia | Unknown | ·· |  | 16 | ·· | ·· | ·· | ·· | ·· | ·· |  |
| Madagascar | Unknown | ·· |  | 82 | ·· | ·· | ·· | ·· | ·· | ·· |  |
| Malawi | Unknown | ·· |  | 30 | ·· | ·· | ·· | ·· | ·· | ·· |  |
| Mali | Unknown | ·· |  | 60 | ·· | ·· | ·· | ·· | ·· | ·· |  |
| Mauritania | Unknown | ·· |  | 23 | ·· | ·· | ·· | ·· | ·· | ·· |  |
| Mauritius | Unknown | ·· |  | 11 | ·· | ·· | ·· | ·· | ·· | ·· |  |
| Mozambique | Unknown | ·· |  | 157 | ·· | ·· | ·· | ·· | ·· | ·· |  |
| Namibia | Unknown | ·· |  | 13 | ·· | ·· | ·· | ·· | ·· | ·· |  |
| Niger | Unknown | ·· |  | 41 | ·· | ·· | ·· | ·· | ·· | ·· |  |
| Nigeria | Unknown | ·· |  | 240 | ·· | ·· | ·· | ·· | ·· | ·· |  |
| Congo | Unknown | ·· |  | 12 | ·· | ·· | ·· | ·· | ·· | ·· |  |
| Rwanda | Unknown | ·· |  | 13 | ·· | ·· | ·· | ·· | ·· | ·· |  |
| Sao Tome & Principe | Unknown | ·· |  | 1 | ·· | ·· | ·· | ·· | ·· | ·· |  |
| Senegal | Unknown | ·· |  | 37 | ·· | ·· | ·· | ·· | ·· | ·· |  |
| Seychelles | Unknown | ·· |  | 3 | ·· | ·· | ·· | ·· | ·· | ·· |  |
| Sierra Leone | Unknown | ·· |  | 21 | ·· | ·· | ·· | ·· | ·· | ·· |  |
| Somalia | Unknown | ·· |  | 14 | ·· | ·· | ·· | ·· | ·· | ·· |  |
| South Africa | Unknown | ·· |  | 235 | ·· | ·· | ·· | ·· | ·· | ·· |  |
| United Republic of Tanzania | Unknown | ·· |  | 126 | ·· | ·· | ·· | ·· | ·· | ·· |  |
| Togo | Unknown | ·· |  | 13 | ·· | ·· | ·· | ·· | ·· | ·· |  |
| Uganda | Unknown | ·· |  | 254 | ·· | ·· | ·· | ·· | ·· | ·· |  |
| Zambia | Unknown | ·· |  | 90 | ·· | ·· | ·· | ·· | ·· | ·· |  |
| Zimbabwe | Unknown | ·· |  | 72 | ·· | ·· | ·· | ·· | ·· | ·· |  |
| **Middle East & North Africa** |  |  |  |  |  |  |  |  | ·· |  |  |
| Algeria | Unknown | ·· |  | 162 | ·· | ·· | ·· | ·· | ·· | ·· |  |
| Bahrain | Unknown | ·· |  | 4 | ·· | ·· | ·· | ·· | ·· | ·· |  |
| Cyprus | Yes | 2022 | 2 | 1 | ·· | ·· | ·· | 0 | Snapshot | 2020 | 1 |
| Egypt | Unknown | ·· |  | 78 | ·· | ·· | ·· | ·· | ·· | ·· |  |
| Iraq | Unknown | ·· |  | 55 | ·· | ·· | ·· | ·· | ·· | ·· |  |
| Israel | Unknown | ·· |  | 30 | ·· | ·· | ·· | ·· | ·· | ·· |  |
| Jordan | Unknown | ·· |  | 17 | ·· | ·· | ·· | ·· | ·· | ·· |  |
| Kuwait | All facilities | 2020 | 24 | 3 | 3 | 2020 | 24 | ·· | ·· | ·· |  |
| Lebanon | Unknown | ·· |  | 25 | ·· | ·· | ·· | ·· | ·· | ·· |  |
| Libya | Unknown | ·· |  | 26 | ·· | ·· | ·· | ·· | ·· | ·· |  |
| Morocco | Yes | 2021 | 25 | 75 | ·· | ·· | ·· | ·· | ·· | ·· |  |
| Oman | Unknown | ·· |  | 3 | ·· | ·· | ·· | ·· | ·· | ·· |  |
| Occupied Palestinian territories | No | 2023 | 17 | N/A | ·· | ·· | ·· | ·· | ·· | ·· |  |
| Qatar | Unknown | ·· |  | 1 | ·· | ·· | ·· | ·· | ·· | ·· |  |
| Saudi Arabia | Unknown | ·· |  | 110 | ·· | ·· | ·· | ·· | ·· | ·· |  |
| South Sudan | Unknown | ·· |  | 80 | ·· | ·· | ·· | ·· | ·· | ·· |  |
| Sudan | Unknown | ·· |  | 125 | ·· | ·· | ·· | ·· | ·· | ·· |  |
| Syrian Arab Republic | Unknown | ·· |  | 35 | ·· | ·· | ·· | ·· | ·· | ·· |  |
| Tunisia | Unknown | ·· |  | 32 | ·· | ·· | ·· | ·· | ·· | ·· |  |
| Türkiye | Yes | 2022 | 2 | 403 | ·· | ·· | ·· | ·· | ·· | ·· |  |
| United Arab Emirates | Unknown | ·· |  | 21 | ·· | ·· | ·· | ·· | ·· | ·· |  |
| Yemen | Unknown | ·· |  | 43 | ·· | ·· | ·· | ·· | ·· | ·· |  |

**Notes:**

**Ref:** Reference; **HCV**: Hepatitis C; **DAA**: Direct-Acting Antiviral.

***Total sites:** Including prisons, jails or other carceral setting, Country level data that informed these regional and global estimates were sourced from the World Prison Brief, collated by the Institute for Crime and Justice Policy Research at Burbeck University. See: <https://www.prisonstudies.org/world-prison-brief-data>;

** The total number of sites for England and Wales was reported together in the World Prison Brief, therefore the total number of sites for both England and Wales is reported as 120.

*** Pilot data only

† For reporting purposes, these countries or territories are reported separately due to differences in service provision.

·· Indicates there were no data to inform a country’s estimate.

**References for Table 6.6**

1. World Health Organization. Status report on prison health in the WHO European Region 2022. Copenhagen, 2023.

2. European Union Drug Agency. EUDA Bulletin - Interventions in Prisons, Europe. 2024.

3. The International Network on Health and Hepatitis in Substance Users: Prisons. HCV Testing & Treatment for All Citizens - Prisons, Moldova. 2024.

4. Andreić J-L. Prison Workbook 2023 - Croatia, 2023.

5. The International Network on Health and Hepatitis in Substance Users: Prisons. Working Together to Eliminate HCV - Prisons, England. 2024.

6. Celse M, Ayachi, L., Artteres, P., Fischer, H., Linden, A., Musso, S., Preau, M., Rousinoux, C., Silvain, C., Suzan-Moni, M., Vana, L., Yeni, P., & Goujard, C. Insufficient HIV, HBV and HCV screening in French penal institutions and impact on actual access of inmates with chronic HCV infection to Direct-Acting Antiviral (DAA) therapy, 2020.

7. Kugener T, Berndt N, Origer A, Debacker M. Prison workbook 2023 Luxembourg. 2023.

8. Bielen R, Stumo, S. R., Halford, R., Werling, K., Reic, T., Stover, H., Robaeys, G., & Lazarus, J. V. Harm reduction and viral hepatitis C in European prisons: A cross-sectional survey of 25 countries. *Harm Reduct J* 2018.

9. de Castro MV, Mendes SCC, Marques JPA, Santos AS, Duarte O. Prison Workbook 2023 - Portugal. 2023.

10. Coalition for Global Hepatitis Elimination and INHSU Prisons. Expanding access to hepatitis C prevention, testing, and treatment in prisons: Recommendations from the INHSU 2022 Prisons Workshop. Atlanta, United States, 2023.

11. McLeod A, Glancy, M, Went, A Smith, S, Weir, A, McAuley, A, Hutchinson, S & Goldberg, D. Surveillance report. Surveillance of hepatitis C testing, diagnosis and treatment in Scotland, 2019 update. Health Protection Scotland, 2019.

12. Gerlich MG, Frick U, Pirktl L, Uchtenhagen A. Detection and treatment of HIV and hepatitis virus infections in Swiss correctional facilities. *Int J Public Health* 2008.

13. Thomas C, Widiastuti H. HCV microelimination project in 7 Jakarta prison. *Hepatol Int* 2020.

14. Harnpariphan W, Han, W. M., Supanun R, Ubolyam S, et al. High Proportion of Blood-Borne and Sexually Transmitted Infections Among People Deprived of Liberty in a Central Male Prison in Thailand: A Cross-Sectional Study 2018-2019. *AIDS Res Hum Retroviruses* 2022.

15. De A, Kaur K, Charak S, et al. Hepatitis C and HIV Co-Infection in Prisons of Punjab. *J Clin Exp Hepatol* 2022.

16. Hariri S, Sharafi H, Sheikh M, et al. Continuum of hepatitis C care cascade in prison and following release in the direct-acting antivirals era. *Harm Reduct J* 2020.

17. Marshall AD, Willing AR, Kairouz A, et al. Direct-acting antiviral therapies for hepatitis C infection: global registration, reimbursement, and restrictions. *Lancet Gastroenterol Hepatol* 2024.

18. Hincapie C. In: Degenhardt L, editor.; 2024.

19. Borquez A. Coverage data in Mexico prisons. In: Thomas Santo Jr., editor.; 2024.

20. Correctional Service of Canada. Infectious disease surveillance in Canadian federal penitentiaries 2007–2008: pre-release report. Correctional Service of Canada Ottawa, ON; 2012.

21. Kronfli N, Dussault C, Bartlett S, et al. Disparities in hepatitis C care across Canadian provincial prisons: Implications for hepatitis C micro-elimination. *Can Liver J* 2021.

22. Beckman AL, Bilinski A, Boyko R, et al. New Hepatitis C Drugs Are Very Costly And Unavailable To Many State Prisoners. *Health Aff (Millwood)* 2016.

23. Burnet Institute and Kirby Institute. Australia’s progress towards hepatitis C elimination: annual report 2023. Melbourne: Burnet Institute, 2023.

24. Hasan F, Alfadhli A, Al-Gharabally A, Alkhaldi M, Colombo M, Lazarus JV. Accelerating the elimination of hepatitis C in Kuwait: An expert opinion. *World J Gastroenterol* 2020; **26**(30): 4415.

25. Royaume du Maroc Ministère de la Santé et de la Protection Sociale. Rapport National SIDA 2022, 2021.

## *Appendix Table 6.7*: Country-level availability of interventions and programmatic data for HBV Testing

|  | | | | **Number of Carceral Sites with HBV Testing** | | | | **Number of People Accessing HBV Testing in Carceral Settings** | | | |
| --- | --- | --- | --- | --- | --- | --- | --- | --- | --- | --- | --- |
| **Country** | **Available** | **Year** | **Ref** | **Total sites*** | **N Sites with HBV Testing** | **Year** | **Ref** | **N People Accessing HBV Testing in Carceral Settings** | **Timeframe** | **Year** | **Ref** |
| **Eastern Europe** |  |  |  |  |  |  |  |  |  |  |  |
| Armenia | All facilities | 2020 | 1 | 12 | 12 | 2020 | 1 | ·· | ·· | ·· |  |
| Azerbaijan | Unknown | ·· |  | 53 | ·· | ·· |  | ·· | ·· | ·· |  |
| Belarus | Unknown | ·· |  | 67 | ·· | ·· |  | ·· | ·· | ·· |  |
| Bosnia & Herzegovina | Yes | 2020 | 1 | 13 | ·· | ·· |  | ·· | ·· | ·· |  |
| Bulgaria | Yes | 2022 | 2 | 55 | 6 | 2022 | 2 | ·· | ·· | ·· |  |
| Czechia | All facilities | 2020 | 1 | 35 | 35 | 2020 | 1 | ·· | ·· | ·· |  |
| Estonia | All facilities | 2022 | 2 | 3 | 3 | 2022 | 2 | ·· | ·· | ·· |  |
| Georgia | All facilities | 2020 | 1 | 14 | 14 | 2020 | 1 | ·· | ·· | ·· |  |
| Hungary | Yes | 2022 | 2 | 30 | 8 | 2022 | 2 | ·· | ·· | ·· |  |
| Latvia | Yes | 2022 | 2 | 9 | ·· | ·· |  | ·· | ·· | ·· |  |
| Lithuania | Yes | 2022 | 2 | 8 | 2 | 2022 | 2 | ·· | ·· | ·· |  |
| Republic of Moldova | All facilities | 2020 | 1 | 17 | 17 | 2020 | 1 | ·· | ·· | ·· |  |
| Poland | All facilities | 2022 | 2 | 215 | 215 | 2022 | 2 | 8570 | Snapshot | 2012 | 3 |
| Romania | Yes | 2022 | 2 | 45 | ·· | ·· |  | ·· | ·· | ·· |  |
| Russian Federation | Unknown | ·· |  | 872 | ·· | ·· |  | ·· | ·· | ·· |  |
| Slovakia | All facilities | 2022 | 2 | 18 | 18 | 2022 | 2 | ·· | ·· | ·· |  |
| Ukraine | All facilities | 2020 | 1 | 110 | 110 | 2020 | 1 | ·· | ·· | ·· |  |
| **Western Europe** |  |  |  |  |  |  |  |  | ·· |  |  |
| Albania | Yes | 2020 | 1 | 23 | 6 | 2017 | 4 | ·· | ·· | ·· |  |
| Andorra | Unknown | ·· |  | 1 | ·· | ·· |  | ·· | ·· | ·· |  |
| Austria | All facilities | 2023 | 5 | 27 | 27 | 2023 | 5 | ·· | ·· | ·· |  |
| Belgium | All facilities | 2020 | 1 | 35 | 35 | 2020 | 1 | ·· | ·· | ·· |  |
| Croatia | Yes | 2023 | 6 | 24 | ·· | ·· |  | ·· | ·· | ·· |  |
| Denmark | Yes | 2022 | 2 | 54 | ·· | ·· |  | ·· | ·· | ·· |  |
| England** | All facilities | 2023 | 7 | 120 | 120 | 2023 | 7 | 24226 | Past year | 2021 | 7 |
| Finland | All facilities | 2022 | 2 | 26 | 26 | 2022 | 2 | ·· | ·· | ·· |  |
| France | All facilities | 2022 | 2 | 187 | 187 | 2022 | 2 | ·· | ·· | ·· |  |
| Germany | Yes | 2022 | 2 | 179 | ·· | ·· |  | ·· | ·· | ·· |  |
| Greece | All facilities | 2020 | 1 | 34 | 34 | 2020 | 1 | ·· | ·· | ·· |  |
| Greenland | Unknown | ·· |  | 6 | ·· | ·· |  | ·· | ·· | ·· |  |
| Iceland | Unknown | ·· |  | 5 | ·· | ·· |  | ·· | ·· | ·· |  |
| Ireland | All facilities | 2022 | 2 | 12 | 12 | 2022 | 2 | ·· | ·· | ·· |  |
| Italy | All facilities | 2020 | 1 | 206 | 206 | 2020 | 1 | ·· | ·· | ·· |  |
| Liechtenstein | Unknown | ·· |  | 1 | ·· | ·· |  | ·· | ·· | ·· |  |
| Luxembourg | Yes | 2023 | 8 | 3 | 1 | 2023 | 8 | ·· | ·· | ·· |  |
| Malta | All facilities | 2020 | 1 | 1 | 1 | 2020 | 1 | 481 | Other | 2019 | 9 |
| Monaco | All facilities | 2020 | 1 | 2 | 2 | 2020 | 1 | ·· | ·· | ·· |  |
| Montenegro | Unknown | ·· |  | 3 | ·· | ·· |  | ·· | ·· | ·· |  |
| Netherlands | Yes | 2021 | 2 | 49 | ·· | ·· |  | ·· | ·· | ·· |  |
| North Macedonia | Unknown | ·· |  | 13 | ·· | ·· |  | ·· | ·· | ·· |  |
| Northern Ireland | All facilities | 2020 | 1 | 3 | 3 | 2020 | 1 | ·· | ·· | ·· |  |
| Norway | Yes | 2022 | 2 | 33 | ·· | ·· |  | ·· | ·· | ·· |  |
| Portugal | All facilities | 2023 | 10 | 49 | 49 | 2023 | 10 | ·· | ·· | ·· |  |
| San Marino | Unknown | ·· |  | 1 | ·· | ·· |  | ·· | ·· | ·· |  |
| Scotland | All facilities | 2022 | 11 | 15 | 15 | 2022 | 11 | ·· | ·· | ·· |  |
| Serbia | Unknown | ·· |  | 30 | ·· | ·· |  | ·· | ·· | ·· |  |
| Slovenia | All facilities | 2022 | 2 | 7 | 7 | 2022 | 2 | ·· | ·· | ·· |  |
| Spain | All facilities | 2022 | 2 | 82 | 82 | 2022 | 2 | ·· | ·· | ·· |  |
| Sweden | All facilities | 2022 | 2 | 79 | 79 | 2022 | 2 | ·· | ·· | ·· |  |
| Switzerland | Yes | 2020 | 1 | 88 | ·· | ·· |  | ·· | ·· | ·· |  |
| Wales** | All facilities | 2020 | 1 | 120 | 120 | 2020 | 1 | ·· | ·· | ·· |  |
| **East and South East Asia** |  |  |  |  |  |  |  |  | ·· |  |  |
| Brunei Darussalam | Unknown | ·· |  | 3 | ·· | ·· |  | ·· | ·· | ·· |  |
| Cambodia | Unknown | ·· |  | 29 | ·· | ·· |  | ·· | ·· | ·· |  |
| China | Unknown | ·· |  | 683 | ·· | ·· |  | ·· | ·· | ·· |  |
| Hong Kong† | Unknown | ·· |  | 24 | ·· | ·· |  | ·· | ·· | ·· |  |
| Indonesia | Unknown | ·· |  | 526 | ·· | ·· |  | ·· | ·· | ·· |  |
| Japan | Unknown | ·· |  | 184 | ·· | ·· |  | ·· | ·· | ·· |  |
| Lao People's Democratic Republic | Unknown | ·· |  | 19 | ·· | ·· |  | ·· | ·· | ·· |  |
| Malaysia | Unknown | ·· |  | 43 | ·· | ·· |  | ·· | ·· | ·· |  |
| Mongolia | Unknown | ·· |  | 50 | ·· | ·· |  | ·· | ·· | ·· |  |
| Myanmar | Unknown | ·· |  | 96 | ·· | ·· |  | ·· | ·· | ·· |  |
| Democratic People's Republic of Korea | Unknown | ·· |  | N/A | ·· | ·· |  | ·· | ·· | ·· |  |
| Philippines | Unknown | ·· |  | 440 | ·· | ·· |  | ·· | ·· | ·· |  |
| Singapore | Unknown | ·· |  | 13 | ·· | ·· |  | ·· | ·· | ·· |  |
| Republic of Korea | Unknown | ·· |  | 54 | ·· | ·· |  | ·· | ·· | ·· |  |
| Taiwan† | Unknown | ·· |  | 49 | ·· | ·· |  | ·· | ·· | ·· |  |
| Thailand | Unknown | ·· |  | 143 | ·· | ·· |  | ·· | ·· | ·· |  |
| Timor-Leste | Unknown | ·· |  | 3 | ·· | ·· |  | ·· | ·· | ·· |  |
| Viet Nam | Unknown | ·· |  | 54 | ·· | ·· |  | ·· | ·· | ·· |  |
| **South Asia** |  |  |  |  |  |  |  |  | ·· |  |  |
| Afghanistan | Unknown | ·· |  | 251 | ·· | ·· |  | ·· | ·· | ·· |  |
| Bangladesh | Unknown | ·· |  | 68 | ·· | ·· |  | ·· | ·· | ·· |  |
| Bhutan | Unknown | ·· |  | 7 | ·· | ·· |  | ·· | ·· | ·· |  |
| India | Unknown | ·· |  | 1330 | ·· | ·· |  | ·· | ·· | ·· |  |
| Islamic Republic of Iran | Unknown | ·· |  | 253 | ·· | ·· |  | ·· | ·· | ·· |  |
| Maldives | Yes | 2017 | ^12^} | 11 | ·· | ·· |  | ·· | ·· | ·· |  |
| Nepal | Unknown | ·· |  | 74 | ·· | ·· |  | ·· | ·· | ·· |  |
| Pakistan | No | 2010 | 13 | 116 | ·· | ·· |  | ·· | ·· | ·· |  |
| Sri Lanka | Unknown | ·· |  | 60 | ·· | ·· |  | ·· | ·· | ·· |  |
| **Central Asia** |  |  |  |  |  |  |  |  | ·· |  |  |
| Kazakhstan | Unknown | ·· |  | 80 | ·· | ·· |  | ·· | ·· | ·· |  |
| Kyrgyzstan | Unknown | ·· |  | 28 | ·· | ·· |  | ·· | ·· | ·· |  |
| Tajikistan | Unknown | ·· |  | 19 | ·· | ·· |  | ·· | ·· | ·· |  |
| Turkmenistan | Unknown | ·· |  | 27 | ·· | ·· |  | ·· | ·· | ·· |  |
| Uzbekistan | Unknown | ·· |  | 54 | ·· | ·· |  | ·· | ·· | ·· |  |
| **Caribbean** |  |  |  |  |  |  |  |  | ·· |  |  |
| Antigua & Barbuda | Unknown | ·· |  | 1 | ·· | ·· |  | ·· | ·· | ·· |  |
| Bahamas | Unknown | ·· |  | 1 | ·· | ·· |  | ·· | ·· | ·· |  |
| Barbados | Unknown | ·· |  | 1 | ·· | ·· |  | ·· | ·· | ·· |  |
| Bermuda | Unknown | ·· |  | 4 | ·· | ·· |  | ·· | ·· | ·· |  |
| Cuba | Unknown | ·· |  | 200 | ·· | ·· |  | ·· | ·· | ·· |  |
| Dominica | Unknown | ·· |  | 1 | ·· | ·· |  | ·· | ·· | ·· |  |
| Dominican Republic | Unknown | ·· |  | 41 | ·· | ·· |  | ·· | ·· | ·· |  |
| Grenada | Unknown | ·· |  | 1 | ·· | ·· |  | ·· | ·· | ·· |  |
| Haiti | Unknown | ·· |  | 17 | ·· | ·· |  | ·· | ·· | ·· |  |
| Jamaica | Unknown | ·· |  | 11 | ·· | ·· |  | ·· | ·· | ·· |  |
| Commonwealth of Puerto Rico | Unknown | ·· |  | 34 | ·· | ·· |  | ·· | ·· | ·· |  |
| Saint Kitts & Nevis | Unknown | ·· |  | 2 | ·· | ·· |  | ·· | ·· | ·· |  |
| Saint Lucia | Unknown | ·· |  | 1 | ·· | ·· |  | ·· | ·· | ·· |  |
| Saint Vincent & the Grenadines | Unknown | ·· |  | 2 | ·· | ·· |  | ·· | ·· | ·· |  |
| Trinidad & Tobago | Unknown | ·· |  | 9 | ·· | ·· |  | ·· | ·· | ·· |  |
| **Latin America** |  |  |  |  |  |  |  |  | ·· |  |  |
| Argentina | Yes | 2011 | 14 | 338 | ·· | ·· |  | ·· | ·· | ·· |  |
| Belize | Unknown | ·· |  | 1 | ·· | ·· |  | ·· | ·· | ·· |  |
| Plurinational State of Bolivia | Unknown | ·· |  | 40 | ·· | ·· |  | ·· | ·· | ·· |  |
| Brazil | Yes | 2018 | 15 | 1386 | ·· | ·· |  | ·· | ·· | ·· |  |
| Chile | Unknown | ·· |  | 113 | ·· | ·· |  | ·· | ·· | ·· |  |
| Colombia | Unknown | ·· |  | 125 | ·· | ·· |  | ·· | ·· | ·· |  |
| Costa Rica | Unknown | ·· |  | 32 | ·· | ·· |  | ·· | ·· | ·· |  |
| Ecuador | Unknown | ·· |  | 52 | ·· | ·· |  | ·· | ·· | ·· |  |
| El Salvador | Unknown | ·· |  | 25 | ·· | ·· |  | ·· | ·· | ·· |  |
| Guatemala | Unknown | ·· |  | 21 | ·· | ·· |  | ·· | ·· | ·· |  |
| Guyana | Unknown | ·· |  | 7 | ·· | ·· |  | ·· | ·· | ·· |  |
| Honduras | Unknown | ·· |  | 26 | ·· | ·· |  | ·· | ·· | ·· |  |
| Mexico | Yes | ·· | 16 | 279 | ·· | ·· |  | ·· | ·· | ·· |  |
| Nicaragua | Unknown | ·· |  | 9 | ·· | ·· |  | ·· | ·· | ·· |  |
| Panama | Unknown | ·· |  | 23 | ·· | ·· |  | ·· | ·· | ·· |  |
| Paraguay | Unknown | ·· |  | 18 | ·· | ·· |  | ·· | ·· | ·· |  |
| Peru | Unknown | ·· |  | 68 | ·· | ·· |  | ·· | ·· | ·· |  |
| Suriname | Unknown | ·· |  | 21 | ·· | ·· |  | ·· | ·· | ·· |  |
| Uruguay | Unknown | ·· |  | 27 | ·· | ·· |  | ·· | ·· | ·· |  |
| Bolivarian Republic of Venezuela | Unknown | ·· |  | 41 | ·· | ·· |  | ·· | ·· | ·· |  |
| **North America** |  |  |  |  |  |  |  |  | ·· |  |  |
| Canada (Federal) | All facilities | 2008 | 17 | 43 | 43 | 2008 | 17 | ·· | ·· | ·· |  |
| Canada (Provincial/Territorial) | Unknown | ·· |  | 173 | ·· | ·· |  | ·· | ·· | ·· |  |
| United States of America (Federal) | Unknown | ·· |  | 102 | ·· | ·· |  | ·· | ·· | ·· |  |
| United States of America (State) | Yes | 2006 | 18 | 1190 | ·· | ·· |  | ·· | ·· | ·· |  |
| **Pacific Island States & Terr.** |  |  |  |  |  |  |  |  | ·· |  |  |
| American Samoa | Unknown | ·· |  | 1 | ·· | ·· |  | ·· | ·· | ·· |  |
| Federated States of Micronesia | Unknown | ·· |  | 4 | ·· | ·· |  | ·· | ·· | ·· |  |
| Fiji | Unknown | ·· |  | 15 | ·· | ·· |  | ·· | ·· | ·· |  |
| French Polynesia | Unknown | ·· |  | 2 | ·· | ·· |  | ·· | ·· | ·· |  |
| Guam | Unknown | ·· |  | 2 | ·· | ·· |  | ·· | ·· | ·· |  |
| Kiribati | Unknown | ·· |  | 4 | ·· | ·· |  | ·· | ·· | ·· |  |
| Marshall Islands | Unknown | ·· |  | 2 | ·· | ·· |  | ·· | ·· | ·· |  |
| Nauru | Unknown | ·· |  | 1 | ·· | ·· |  | ·· | ·· | ·· |  |
| New Caledonia | Unknown | ·· |  | 1 | ·· | ·· |  | ·· | ·· | ·· |  |
| Northern Mariana Islands | Unknown | ·· |  | 1 | ·· | ·· |  | ·· | ·· | ·· |  |
| Palau | Unknown | ·· |  | 1 | ·· | ·· |  | ·· | ·· | ·· |  |
| Papua New Guinea | Unknown | ·· |  | 18 | ·· | ·· |  | ·· | ·· | ·· |  |
| Samoa | Unknown | ·· |  | 3 | ·· | ·· |  | ·· | ·· | ·· |  |
| Solomon Islands | Unknown | ·· |  | 6 | ·· | ·· |  | ·· | ·· | ·· |  |
| Tonga | Unknown | ·· |  | 4 | ·· | ·· |  | ·· | ·· | ·· |  |
| Tuvalu | Unknown | ·· |  | 2 | ·· | ·· |  | ·· | ·· | ·· |  |
| Vanuatu | Unknown | ·· |  | 4 | ·· | ·· |  | ·· | ·· | ·· |  |
| **Australasia** |  |  |  |  |  |  |  |  | ·· |  |  |
| Australia | Yes | 2009 | 19 | 111 | ·· | ·· |  | ·· | ·· | ·· |  |
| New Zealand | Unknown | ·· |  | 18 | ·· | ·· |  | ·· | ·· | ·· |  |
| **Sub Saharan Africa** |  |  |  |  |  |  |  |  | ·· |  |  |
| Angola | Unknown | ·· |  | 47 | ·· | ·· |  | ·· | ·· | ·· |  |
| Benin | Unknown | ·· |  | 11 | ·· | ·· |  | ·· | ·· | ·· |  |
| Botswana | Unknown | ·· |  | 23 | ·· | ·· |  | ·· | ·· | ·· |  |
| Burkina Faso | Unknown | ·· |  | 27 | ·· | ·· |  | ·· | ·· | ·· |  |
| Burundi | Unknown | ·· |  | 13 | ·· | ·· |  | ·· | ·· | ·· |  |
| Cameroon | Yes | 2021 | 20 | 76 | ·· | ·· |  | ·· | ·· | ·· |  |
| Cabo Verde | Unknown | ·· |  | 5 | ·· | ·· |  | ·· | ·· | ·· |  |
| Central African Republic | Unknown | ·· |  | 15 | ·· | ·· |  | ·· | ·· | ·· |  |
| Chad | Unknown | ·· |  | 41 | ·· | ·· |  | ·· | ·· | ·· |  |
| Comoros | Unknown | ·· |  | 3 | ·· | ·· |  | ·· | ·· | ·· |  |
| Côte d'Ivoire | Unknown | ·· |  | 34 | ·· | ·· |  | ·· | ·· | ·· |  |
| Democratic Republic of the Congo | Unknown | ·· |  | 142 | ·· | ·· |  | ·· | ·· | ·· |  |
| Djibouti | Unknown | ·· |  | 1 | ·· | ·· |  | ·· | ·· | ·· |  |
| Equatorial Guinea | Unknown | ·· |  | 15 | ·· | ·· |  | ·· | ·· | ·· |  |
| Eritrea | Unknown | ·· |  | N/A | ·· | ·· |  | ·· | ·· | ·· |  |
| Eswatini | Unknown | ·· |  | 12 | ·· | ·· |  | ·· | ·· | ·· |  |
| Ethiopia | Unknown | ·· |  | 126 | ·· | ·· |  | ·· | ·· | ·· |  |
| Gabon | Unknown | ·· |  | 9 | ·· | ·· |  | ·· | ·· | ·· |  |
| Gambia | Unknown | ·· |  | 3 | ·· | ·· |  | ·· | ·· | ·· |  |
| Ghana | Unknown | ·· |  | 46 | ·· | ·· |  | ·· | ·· | ·· |  |
| Guinea | Unknown | ·· |  | 31 | ·· | ·· |  | ·· | ·· | ·· |  |
| Guinea-Bissau | Unknown | ·· |  | 3 | ·· | ·· |  | ·· | ·· | ·· |  |
| Kenya | Unknown | ·· |  | 134 | ·· | ·· |  | ·· | ·· | ·· |  |
| Lesotho | Unknown | ·· |  | 14 | ·· | ·· |  | ·· | ·· | ·· |  |
| Liberia | Unknown | ·· |  | 16 | ·· | ·· |  | ·· | ·· | ·· |  |
| Madagascar | Unknown | ·· |  | 82 | ·· | ·· |  | ·· | ·· | ·· |  |
| Malawi | Unknown | ·· |  | 30 | ·· | ·· |  | ·· | ·· | ·· |  |
| Mali | Unknown | ·· |  | 60 | ·· | ·· |  | ·· | ·· | ·· |  |
| Mauritania | Unknown | ·· |  | 23 | ·· | ·· |  | ·· | ·· | ·· |  |
| Mauritius | Unknown | ·· |  | 11 | ·· | ·· |  | ·· | ·· | ·· |  |
| Mozambique | Unknown | ·· |  | 157 | ·· | ·· |  | ·· | ·· | ·· |  |
| Namibia | Unknown | ·· |  | 13 | ·· | ·· |  | ·· | ·· | ·· |  |
| Niger | Unknown | ·· |  | 41 | ·· | ·· |  | ·· | ·· | ·· |  |
| Nigeria | No | 2021 | 21 | 240 | ·· | ·· |  | ·· | ·· | ·· |  |
| Congo | Unknown | ·· |  | 12 | ·· | ·· |  | ·· | ·· | ·· |  |
| Rwanda | Unknown | ·· |  | 13 | ·· | ·· |  | ·· | ·· | ·· |  |
| Sao Tome & Principe | Unknown | ·· |  | 1 | ·· | ·· |  | ·· | ·· | ·· |  |
| Senegal | Unknown | ·· |  | 37 | ·· | ·· |  | ·· | ·· | ·· |  |
| Seychelles | Unknown | ·· |  | 3 | ·· | ·· |  | ·· | ·· | ·· |  |
| Sierra Leone | Unknown | ·· |  | 21 | ·· | ·· |  | ·· | ·· | ·· |  |
| Somalia | Unknown | ·· |  | 14 | ·· | ·· |  | ·· | ·· | ·· |  |
| South Africa | Unknown | ·· |  | 235 | ·· | ·· |  | ·· | ·· | ·· |  |
| United Republic of Tanzania | Unknown | ·· |  | 126 | ·· | ·· |  | ·· | ·· | ·· |  |
| Togo | Unknown | ·· |  | 13 | ·· | ·· |  | ·· | ·· | ·· |  |
| Uganda | Unknown | ·· |  | 254 | ·· | ·· |  | ·· | ·· | ·· |  |
| Zambia | Unknown | ·· |  | 90 | ·· | ·· |  | ·· | ·· | ·· |  |
| Zimbabwe | Unknown | ·· |  | 72 | ·· | ·· |  | ·· | ·· | ·· |  |
| **Middle East & North Africa** |  |  |  |  |  |  |  |  | ·· |  |  |
| Algeria | Unknown | ·· |  | 162 | ·· | ·· |  | ·· | ·· | ·· |  |
| Bahrain | Unknown | ·· |  | 4 | ·· | ·· |  | ·· | ·· | ·· |  |
| Cyprus | All facilities | 2022 | 2 | 1 | 1 | 2022 | 2 | ·· | ·· | ·· |  |
| Egypt | Yes | 2019 | 22 | 78 | 3 | 2019 | 22 | ·· | ·· | ·· |  |
| Iraq | Unknown | ·· |  | 55 | ·· | ·· |  | ·· | ·· | ·· |  |
| Israel | Unknown | ·· |  | 30 | ·· | ·· |  | ·· | ·· | ·· |  |
| Jordan | Unknown | ·· |  | 17 | ·· | ·· |  | ·· | ·· | ·· |  |
| Kuwait | Unknown | ·· |  | 3 | ·· | ·· |  | ·· | ·· | ·· |  |
| Lebanon | Yes | 2010 | 23 | 25 | ·· | ·· |  | ·· | ·· | ·· |  |
| Libya | Unknown | ·· |  | 26 | ·· | ·· |  | ·· | ·· | ·· |  |
| Morocco | Unknown | ·· |  | 75 | ·· | ·· |  | ·· | ·· | ·· |  |
| Oman | Unknown | ·· |  | 3 | ·· | ·· |  | ·· | ·· | ·· |  |
| Occupied Palestinian territories | Unknown | ·· |  | N/A | ·· | ·· |  | ·· | ·· | ·· |  |
| Qatar | Unknown | ·· |  | 1 | ·· | ·· |  | ·· | ·· | ·· |  |
| Saudi Arabia | Unknown | ·· |  | 110 | ·· | ·· |  | ·· | ·· | ·· |  |
| South Sudan | Unknown | ·· |  | 80 | ·· | ·· |  | ·· | ·· | ·· |  |
| Sudan | Unknown | ·· |  | 125 | ·· | ·· |  | ·· | ·· | ·· |  |
| Syrian Arab Republic | Unknown | ·· |  | 35 | ·· | ·· |  | ·· | ·· | ·· |  |
| Tunisia | Unknown | ·· |  | 32 | ·· | ·· |  | ·· | ·· | ·· |  |
| Türkiye | Yes | 2022 | 2 | 403 | ·· | ·· |  | ·· | ·· | ·· |  |
| United Arab Emirates | Unknown | ·· |  | 21 | ·· | ·· |  | ·· | ·· | ·· |  |
| Yemen | Unknown | ·· |  | 43 | ·· | ·· |  | ·· | ·· | ·· |  |

**Notes:**

**Ref:** Reference; **HBV**: Hepatitis B.

***Total sites:** Including prisons, jails or other carceral setting, Country level data that informed these regional and global estimates were sourced from the World Prison Brief, collated by the Institute for Crime and Justice Policy Research at Burbeck University. See: <https://www.prisonstudies.org/world-prison-brief-data>;

** The total number of sites for England and Wales was reported together in the World Prison Brief, therefore the total number of sites for both England and Wales is reported as 120.

† For reporting purposes, these countries or territories are reported separately due to differences in service provision.

·· Indicates there were no data to inform a country’s estimate.

**References for Table 6.7**

1. World Health Organization. Status report on prison health in the WHO European Region 2022. Copenhagen, 2023.

2. European Union Drug Agency. EUDA Bulletin - Interventions in Prisons, Europe. 2024.

3. Malczewski A, Misiurek, A., Bukowska, B., Chojecki, D., Jabłoński, P., Kidawa, M., Niedźwiedzka-Stadnik, M., Poleganow, A., Radomska, A., Rosińska, M., Sollich, K., Stawecka, E., Struzik, M., Strzelecka, A., & Walichnowska, M. National Report 2013, Poland: new developments, trends and in-depth information on selected issues, 2012.

4. EMCDDA. Albania National Drug Report 2017: EMCDDA, 2017.

5. Reitox Focal Point Austria, Gesundheit Österreich GmbH Vienna. Prison workbook 2023 - Austria. Austria; 2023.

6. Andreić J-L. Prison Workbook 2023 - Croatia, 2023.

7. UK Health Security Agency. Taking a place-based approach to tackling hepatitis in prisons. 2023.

8. Kugener T, Berndt N, Origer A, Debacker M. Prison workbook 2023 Luxembourg. 2023.

9. Muscat K, Cremona C, Fenech TM, Abela M, Padovese V. Sexually transmitted infections epidemiology and risk assessment at the main correctional facility in Malta (2017-2019). *J Eur Acad Dermatol Venereol* 2022.

10. de Castro MV, Mendes SCC, Marques JPA, Santos AS, Duarte O. Prison Workbook 2023 - Portugal. 2023.

11. Bell N, Humphries K, Heskin J, Dunn J, Chan SY. A review of sexual health and blood-borne virus care provided to inmates at admission into UK prisons and secure facilities. *HIV Med* 2023; **24(Supplement 3)**: 4.

12. Maldives Correctional Service. Healthcare in Prison. 2017. https://en.corrections.gov.mv/2017/09/21/healthcare/.

13. Kazi AM, Shah SA, Jenkins CA, Shepherd BE, Vermund SH. Risk factors and prevalence of tuberculosis, human immunodeficiency virus, syphilis, hepatitis B virus, and hepatitis C virus among prisoners in Pakistan. *Int J Infect Dis* 2010.

14. Hariga F. Evaluation and Recommendations for the Improvement of the health Programmes, Including for the Prevention and Treatment of Drug Dependence and of HIV and AIDS, Implemented in the Establishments Under the Responsibility of the Federal Penitentiary Service in Argentina: a Follow-up of the 2008 Assessment. Vienna, Austria, 2011.

15. do Nascimento CT, Pena DZ, Giuffrida R, et al. Prevalence and epidemiological characteristics of inmates diagnosed with infectious diseases living in a region with a high number of prisons in Sao Paulo state, Brazil. *BMJ Open* 2020.

16. Center for the Prevention and Comprehensive Care of HIV/AIDS of the Federal District Mexico City. HIV Detection and Care: Centro para la Prevención y Atención Integral del VIH/SIDA del Distrito Federal, Programa de VIH/sida de la Ciudad de México, Clínica Especializada Condesa.

17. Correctional Service of Canada. Infectious disease surveillance in Canadian federal penitentiaries 2007–2008: pre-release report. Correctional Service of Canada Ottawa, ON; 2012.

18. Baillargen J, Snyder N, Soloway RD, et al. Hepatocellular Carcinoma Prevalence and Mortality in a Male State Prison Population. *Public Health Rep* 2009.

19. Watkins RE, Mak DB, Connelly C. Testing for sexually transmitted infections and blood borne viruses on admission to Western Australian prisons. *BMC Public Health* 2009.

20. Noeske J, Ndi NF, Nga FHM, Mely G, Kuaban C. Prison health services across ten central prisons in Cameroon. *J Public Health Afr* 2023; **14**(9).

21. Dan-Nwafor CC, Adeoye I, Aderemi K, et al. Serological markers and risk factors associated with Hepatitis B virus infection among Federal Capital Territory prison inmates, Nigeria: Should we be concerned? *PLoS One* 2021.

22. United Nations Office on Drugs and Crime ROMENA. Egypt: UNODC inaugurates first-ever prison Voluntary Confidential Counseling, Testing and aftercare services. 2019.

23. Mahfoud Z, Kassak K, Kreidieh K, Shamra S, Ramia S. Prevalence of antibodies to human immunodeficiency virus (HIV), hepatitis B and hepatitis C and risk factors in prisoners in Lebanon. *J Infect Dev Ctries* 2010.

## *Appendix Table 6.8*: Country-level availability of interventions and programmatic data for HBV Treatment

|  | | | | **Number of Carceral Sites with HBV Treatment** | | | | **Number of People Accessing HBV Treatment in Carceral Settings** | | | |
| --- | --- | --- | --- | --- | --- | --- | --- | --- | --- | --- | --- |
| **Country** | **Available** | **Year** | **Ref** | **Total sites*** | **N Sites with HBV Treatment** | **Year** | **Ref** | **N People Accessing HBV Treatment in Carceral Settings** | **Timeframe** | **Year** | **Ref** |
| **Eastern Europe** |  |  |  |  |  |  |  |  |  |  |  |
| Armenia | Unknown | ·· |  | 12 | ·· | ·· |  | ·· | ·· | ·· |  |
| Azerbaijan | Unknown | ·· |  | 53 | ·· | ·· |  | ·· | ·· | ·· |  |
| Belarus | Unknown | ·· |  | 67 | ·· | ·· |  | ·· | ·· | ·· |  |
| Bosnia & Herzegovina | Yes | 2020 | 1 | 13 | ·· | ·· |  | 4 | 2020 | 2020 | 1 |
| Bulgaria | No | 2022 | 2 | 55 | ·· | ·· |  | ·· | ·· | ·· |  |
| Czechia | Yes | 2022 | 2 | 35 | ·· | ·· |  | 29 | 2020 | 2020 | 1 |
| Estonia | Yes | 2021 | 2 | 3 | ·· | ·· |  | 3 | 2020 | 2020 | 1 |
| Georgia | Yes | 2020 | 1 | 14 | ·· | ·· |  | 1 | 2020 | 2020 | 1 |
| Hungary | Yes | 2022 | 2 | 30 | 9 | 2022 | 2 | 3 | 2020 | 2020 | 1 |
| Latvia | Yes | 2022 | 2 | 9 | ·· | ·· |  | 9 | 2020 | 2020 | 1 |
| Lithuania | Yes | 2022 | 2 | 8 | 2 | 2022 | 2 | ·· | ·· | ·· |  |
| Republic of Moldova | Yes | 2021 | 3 | 17 | ·· | ·· |  | 94 | 2020 | 2020 | 1 |
| Poland | Yes | 2022 | 2 | 215 | ·· | ·· |  | ·· | ·· | ·· |  |
| Romania | Yes | 2022 | 2 | 45 | ·· | ·· |  | ·· | ·· | ·· |  |
| Russian Federation | Unknown | ·· |  | 872 | ·· | ·· |  | ·· | ·· | ·· |  |
| Slovakia | Yes | 2020 | 1 | 18 | ·· | ·· |  | 2 | 2020 | 2020 | 1 |
| Ukraine | Yes | 2020 | 1 | 110 | ·· | ·· |  | 1325 | 2020 | 2020 | 1 |
| **Western Europe** |  |  |  |  |  |  |  |  | ·· |  |  |
| Albania | Unknown | ·· |  | 23 | ·· | ·· |  | ·· | ·· | ·· |  |
| Andorra | Unknown | ·· |  | 1 | ·· | ·· |  | ·· | ·· | ·· |  |
| Austria | All facilities | 2023 | 4 | 27 | 27 | 2023 | 4 | 245 | 2020 | 2020 | 1 |
| Belgium | Yes | 2022 | 5 | 35 | ·· | ·· |  | ·· | ·· | ·· |  |
| Croatia | All facilities | 2023 | 6 | 24 | 24 | 2023 | 6 | ·· | ·· | ·· |  |
| Denmark | Unknown | ·· |  | 54 | ·· | ·· |  | ·· | ·· | ·· |  |
| England** | All facilities | 2023 | 7 | 120 | 120 | 2023 | 7 | ·· | ·· | ·· |  |
| Finland | All facilities | 2022 | 2 | 26 | 26 | 2022 | 2 | ·· | ·· | ·· |  |
| France | Yes | 2022 | 2 | 187 | ·· | ·· |  | ·· | ·· | ·· |  |
| Germany | Yes | 2022 | 2 | 179 | ·· | ·· |  | 8 | 2020 | 2020 | 1 |
| Greece | Yes | 2021 | 2 | 34 | ·· | ·· |  | 2 | 2020 | 2020 | 1 |
| Greenland | Unknown | ·· |  | 6 | ·· | ·· |  | ·· | ·· | ·· |  |
| Iceland | Unknown | ·· |  | 5 | ·· | ·· |  | ·· | ·· | ·· |  |
| Ireland | Yes | 2021 | 2 | 12 | ·· | ·· |  | ·· | ·· | ·· |  |
| Italy | Yes | 2022 | 2 | 206 | ·· | ·· |  | ·· | ·· | ·· |  |
| Liechtenstein | Unknown | ·· |  | 1 | ·· | ·· |  | ·· | ·· | ·· |  |
| Luxembourg | All facilities | 2023 | 8 | 3 | 3 | 2023 | 8 | 13 | 2020 | 2020 | 1 |
| Malta | Yes | 2022 | 5 | 1 | ·· | ·· |  | 27 | 2020 | 2020 | 1 |
| Monaco | Unknown | ·· |  | 2 | ·· | ·· |  | ·· | ·· | ·· |  |
| Montenegro | Unknown | ·· |  | 3 | ·· | ·· |  | ·· | ·· | ·· |  |
| Netherlands | Yes | 2021 | 2 | 49 | ·· | ·· |  | ·· | ·· | ·· |  |
| North Macedonia | Unknown | ·· |  | 13 | ·· | ·· |  | ·· | ·· | ·· |  |
| Northern Ireland | Unknown | ·· |  | 3 | ·· | ·· |  | ·· | ·· | ·· |  |
| Norway | Yes | 2022 | 2 | 33 | ·· | ·· |  | ·· | ·· | ·· |  |
| Portugal | All facilities | 2023 | 9 | 49 | 49 | 2023 | 9 | ·· | ·· | ·· |  |
| San Marino | Unknown | ·· |  | 1 | ·· | ·· |  | ·· | ·· | ·· |  |
| Scotland | Unknown | ·· |  | 15 | ·· | ·· |  | ·· | ·· | ·· |  |
| Serbia | Unknown | ·· |  | 30 | ·· | ·· |  | ·· | ·· | ·· |  |
| Slovenia | All facilities | 2022 | 2 | 7 | 7 | 2022 | 2 | ·· | ·· | ·· |  |
| Spain | All facilities | 2022 | 2 | 82 | 82 | 2022 | 2 | ·· | ·· | ·· |  |
| Sweden | All facilities | 2022 | 2 | 79 | 79 | 2022 | 2 | ·· | ·· | ·· |  |
| Switzerland | Yes | 2004 | 10 | 88 | 35 | 2004 | 10 | ·· | ·· | ·· |  |
| Wales** | Unknown | ·· |  | 120 | ·· | ·· |  | ·· | ·· | ·· |  |
| **East and South East Asia** |  |  |  |  |  |  |  |  |  |  |  |
| Brunei Darussalam | Unknown | ·· |  | 3 | ·· | ·· |  | ·· | ·· | ·· |  |
| Cambodia | Unknown | ·· |  | 29 | ·· | ·· |  | ·· | ·· | ·· |  |
| China | Unknown | ·· |  | 683 | ·· | ·· |  | ·· | ·· | ·· |  |
| Hong Kong† | Unknown | ·· |  | 24 | ·· | ·· |  | ·· | ·· | ·· |  |
| Indonesia | Unknown | ·· |  | 526 | ·· | ·· |  | ·· | ·· | ·· |  |
| Japan | Unknown | ·· |  | 184 | ·· | ·· |  | ·· | ·· | ·· |  |
| Lao People's Democratic Republic | Unknown | ·· |  | 19 | ·· | ·· |  | ·· | ·· | ·· |  |
| Malaysia | Unknown | ·· |  | 43 | ·· | ·· |  | ·· | ·· | ·· |  |
| Mongolia | Unknown | ·· |  | 50 | ·· | ·· |  | ·· | ·· | ·· |  |
| Myanmar | Unknown | ·· |  | 96 | ·· | ·· |  | ·· | ·· | ·· |  |
| Democratic People's Republic of Korea | Unknown | ·· |  | N/A | ·· | ·· |  | ·· | ·· | ·· |  |
| Philippines | Unknown | ·· |  | 440 | ·· | ·· |  | ·· | ·· | ·· |  |
| Singapore | Unknown | ·· |  | 13 | ·· | ·· |  | ·· | ·· | ·· |  |
| Republic of Korea | Unknown | ·· |  | 54 | ·· | ·· |  | ·· | ·· | ·· |  |
| Taiwan† | Unknown | ·· |  | 49 | ·· | ·· |  | ·· | ·· | ·· |  |
| Thailand | Unknown | ·· |  | 143 | ·· | ·· |  | ·· | ·· | ·· |  |
| Timor-Leste | Unknown | ·· |  | 3 | ·· | ·· |  | ·· | ·· | ·· |  |
| Viet Nam | Unknown | ·· |  | 54 | ·· | ·· |  | ·· | ·· | ·· |  |
| **South Asia** |  |  |  |  |  |  |  |  |  |  |  |
| Afghanistan | Unknown | ·· |  | 251 | ·· | ·· |  | ·· | ·· | ·· |  |
| Bangladesh | Unknown | ·· |  | 68 | ·· | ·· |  | ·· | ·· | ·· |  |
| Bhutan | Unknown | ·· |  | 7 | ·· | ·· |  | ·· | ·· | ·· |  |
| India | Unknown | ·· |  | 1330 | ·· | ·· |  | ·· | ·· | ·· |  |
| Islamic Republic of Iran | Unknown | ·· |  | 253 | ·· | ·· |  | ·· | ·· | ·· |  |
| Maldives | Unknown | ·· |  | 11 | ·· | ·· |  | ·· | ·· | ·· |  |
| Nepal | Unknown | ·· |  | 74 | ·· | ·· |  | ·· | ·· | ·· |  |
| Pakistan | Unknown | ·· |  | 116 | ·· | ·· |  | ·· | ·· | ·· |  |
| Sri Lanka | Unknown | ·· |  | 60 | ·· | ·· |  | ·· | ·· | ·· |  |
| **Central Asia** |  |  |  |  |  |  |  |  |  |  |  |
| Kazakhstan | Unknown | ·· |  | 80 | ·· | ·· |  | ·· | ·· | ·· |  |
| Kyrgyzstan | Unknown | ·· |  | 28 | ·· | ·· |  | ·· | ·· | ·· |  |
| Tajikistan | Unknown | ·· |  | 19 | ·· | ·· |  | ·· | ·· | ·· |  |
| Turkmenistan | Unknown | ·· |  | 27 | ·· | ·· |  | ·· | ·· | ·· |  |
| Uzbekistan | Unknown | ·· |  | 54 | ·· | ·· |  | ·· | ·· | ·· |  |
| **Caribbean** |  |  |  |  |  |  |  |  |  |  |  |
| Antigua & Barbuda | Unknown | ·· |  | 1 | ·· | ·· |  | ·· | ·· | ·· |  |
| Bahamas | Unknown | ·· |  | 1 | ·· | ·· |  | ·· | ·· | ·· |  |
| Barbados | Unknown | ·· |  | 1 | ·· | ·· |  | ·· | ·· | ·· |  |
| Bermuda | Unknown | ·· |  | 4 | ·· | ·· |  | ·· | ·· | ·· |  |
| Cuba | Unknown | ·· |  | 200 | ·· | ·· |  | ·· | ·· | ·· |  |
| Dominica | Unknown | ·· |  | 1 | ·· | ·· |  | ·· | ·· | ·· |  |
| Dominican Republic | Unknown | ·· |  | 41 | ·· | ·· |  | ·· | ·· | ·· |  |
| Grenada | Unknown | ·· |  | 1 | ·· | ·· |  | ·· | ·· | ·· |  |
| Haiti | Unknown | ·· |  | 17 | ·· | ·· |  | ·· | ·· | ·· |  |
| Jamaica | Unknown | ·· |  | 11 | ·· | ·· |  | ·· | ·· | ·· |  |
| Commonwealth of Puerto Rico | Unknown | ·· |  | 34 | ·· | ·· |  | ·· | ·· | ·· |  |
| Saint Kitts & Nevis | Unknown | ·· |  | 2 | ·· | ·· |  | ·· | ·· | ·· |  |
| Saint Lucia | Unknown | ·· |  | 1 | ·· | ·· |  | ·· | ·· | ·· |  |
| Saint Vincent & the Grenadines | Unknown | ·· |  | 2 | ·· | ·· |  | ·· | ·· | ·· |  |
| Trinidad & Tobago | Unknown | ·· |  | 9 | ·· | ·· |  | ·· | ·· | ·· |  |
| **Latin America** |  |  |  |  |  |  |  |  |  |  |  |
| Argentina | Yes | 2011 | 11 | 338 | ·· | ·· |  | ·· | ·· | ·· |  |
| Belize | Unknown | ·· |  | 1 | ·· | ·· |  | ·· | ·· | ·· |  |
| Plurinational State of Bolivia | Unknown | ·· |  | 40 | ·· | ·· |  | ·· | ·· | ·· |  |
| Brazil | Unknown | ·· |  | 1386 | ·· | ·· |  | ·· | ·· | ·· |  |
| Chile | Unknown | ·· |  | 113 | ·· | ·· |  | ·· | ·· | ·· |  |
| Colombia | Unknown | ·· |  | 125 | ·· | ·· |  | ·· | ·· | ·· |  |
| Costa Rica | Unknown | ·· |  | 32 | ·· | ·· |  | ·· | ·· | ·· |  |
| Ecuador | Unknown | ·· |  | 52 | ·· | ·· |  | ·· | ·· | ·· |  |
| El Salvador | Unknown | ·· |  | 25 | ·· | ·· |  | ·· | ·· | ·· |  |
| Guatemala | Unknown | ·· |  | 21 | ·· | ·· |  | ·· | ·· | ·· |  |
| Guyana | Unknown | ·· |  | 7 | ·· | ·· |  | ·· | ·· | ·· |  |
| Honduras | Unknown | ·· |  | 26 | ·· | ·· |  | ·· | ·· | ·· |  |
| Mexico | Unknown | ·· |  | 279 | ·· | ·· |  | ·· | ·· | ·· |  |
| Nicaragua | Unknown | ·· |  | 9 | ·· | ·· |  | ·· | ·· | ·· |  |
| Panama | Unknown | ·· |  | 23 | ·· | ·· |  | ·· | ·· | ·· |  |
| Paraguay | Unknown | ·· |  | 18 | ·· | ·· |  | ·· | ·· | ·· |  |
| Peru | Yes | 2016 | 12 | 68 | ·· | ·· |  | ·· | ·· | ·· |  |
| Suriname | Unknown | ·· |  | 21 | ·· | ·· |  | ·· | ·· | ·· |  |
| Uruguay | Unknown | ·· |  | 27 | ·· | ·· |  | ·· | ·· | ·· |  |
| Bolivarian Republic of Venezuela | Unknown | ·· |  | 41 | ·· | ·· |  | ·· | ·· | ·· |  |
| **North America** |  |  |  |  |  |  |  |  |  |  |  |
| Canada (Federal) | Unknown | ·· |  | 43 | ·· | ·· |  | ·· | ·· | ·· |  |
| Canada (Provincial/Territorial) | Unknown | ·· |  | 173 | ·· | ·· |  | ·· | ·· | ·· |  |
| United States of America (Federal) | Unknown | ·· |  | 102 | ·· | ·· |  | ·· | ·· | ·· |  |
| United States of America(State) | Unknown | ·· |  | 1190 | ·· | ·· |  | ·· | ·· | ·· |  |
| **Pacific Island States & Terr.** |  |  |  |  |  |  |  |  |  |  |  |
| American Samoa | Unknown | ·· |  | 1 | ·· | ·· |  | ·· | ·· | ·· |  |
| Federated States of Micronesia | Unknown | ·· |  | 4 | ·· | ·· |  | ·· | ·· | ·· |  |
| Fiji | Unknown | ·· |  | 15 | ·· | ·· |  | ·· | ·· | ·· |  |
| French Polynesia | Unknown | ·· |  | 2 | ·· | ·· |  | ·· | ·· | ·· |  |
| Guam | Unknown | ·· |  | 2 | ·· | ·· |  | ·· | ·· | ·· |  |
| Kiribati | Unknown | ·· |  | 4 | ·· | ·· |  | ·· | ·· | ·· |  |
| Marshall Islands | Unknown | ·· |  | 2 | ·· | ·· |  | ·· | ·· | ·· |  |
| Nauru | Unknown | ·· |  | 1 | ·· | ·· |  | ·· | ·· | ·· |  |
| New Caledonia | Unknown | ·· |  | 1 | ·· | ·· |  | ·· | ·· | ·· |  |
| Northern Mariana Islands | Unknown | ·· |  | 1 | ·· | ·· |  | ·· | ·· | ·· |  |
| Palau | Unknown | ·· |  | 1 | ·· | ·· |  | ·· | ·· | ·· |  |
| Papua New Guinea | Unknown | ·· |  | 18 | ·· | ·· |  | ·· | ·· | ·· |  |
| Samoa | Unknown | ·· |  | 3 | ·· | ·· |  | ·· | ·· | ·· |  |
| Solomon Islands | Unknown | ·· |  | 6 | ·· | ·· |  | ·· | ·· | ·· |  |
| Tonga | Unknown | ·· |  | 4 | ·· | ·· |  | ·· | ·· | ·· |  |
| Tuvalu | Unknown | ·· |  | 2 | ·· | ·· |  | ·· | ·· | ·· |  |
| Vanuatu | Unknown | ·· |  | 4 | ·· | ·· |  | ·· | ·· | ·· |  |
| **Australasia** |  |  |  |  |  |  |  |  |  |  |  |
| Australia | All facilities | 2020 | 13 | 111 | 111 | 2020 | 13 | ·· | ·· | ·· |  |
| New Zealand | Unknown | ·· |  | 18 | ·· | ·· |  | ·· | ·· | ·· |  |
| **Sub Saharan Africa** |  |  |  |  |  |  |  |  |  |  |  |
| Angola | Unknown | ·· |  | 47 | ·· | ·· |  | ·· | ·· | ·· |  |
| Benin | Unknown | ·· |  | 11 | ·· | ·· |  | ·· | ·· | ·· |  |
| Botswana | Unknown | ·· |  | 23 | ·· | ·· |  | ·· | ·· | ·· |  |
| Burkina Faso | Unknown | ·· |  | 27 | ·· | ·· |  | ·· | ·· | ·· |  |
| Burundi | Unknown | ·· |  | 13 | ·· | ·· |  | ·· | ·· | ·· |  |
| Cameroon | Unknown | ·· |  | 76 | ·· | ·· |  | ·· | ·· | ·· |  |
| Cabo Verde | Unknown | ·· |  | 5 | ·· | ·· |  | ·· | ·· | ·· |  |
| Central African Republic | Unknown | ·· |  | 15 | ·· | ·· |  | ·· | ·· | ·· |  |
| Chad | Unknown | ·· |  | 41 | ·· | ·· |  | ·· | ·· | ·· |  |
| Comoros | Unknown | ·· |  | 3 | ·· | ·· |  | ·· | ·· | ·· |  |
| Côte d'Ivoire | Unknown | ·· |  | 34 | ·· | ·· |  | ·· | ·· | ·· |  |
| Democratic Republic of the Congo | Unknown | ·· |  | 142 | ·· | ·· |  | ·· | ·· | ·· |  |
| Djibouti | Unknown | ·· |  | 1 | ·· | ·· |  | ·· | ·· | ·· |  |
| Equatorial Guinea | Unknown | ·· |  | 15 | ·· | ·· |  | ·· | ·· | ·· |  |
| Eritrea | Unknown | ·· |  | N/A | ·· | ·· |  | ·· | ·· | ·· |  |
| Eswatini | Unknown | ·· |  | 12 | ·· | ·· |  | ·· | ·· | ·· |  |
| Ethiopia | Unknown | ·· |  | 126 | ·· | ·· |  | ·· | ·· | ·· |  |
| Gabon | Unknown | ·· |  | 9 | ·· | ·· |  | ·· | ·· | ·· |  |
| Gambia | Unknown | ·· |  | 3 | ·· | ·· |  | ·· | ·· | ·· |  |
| Ghana | Unknown | ·· |  | 46 | ·· | ·· |  | ·· | ·· | ·· |  |
| Guinea | Unknown | ·· |  | 31 | ·· | ·· |  | ·· | ·· | ·· |  |
| Guinea-Bissau | Unknown | ·· |  | 3 | ·· | ·· |  | ·· | ·· | ·· |  |
| Kenya | Unknown | ·· |  | 134 | ·· | ·· |  | ·· | ·· | ·· |  |
| Lesotho | Unknown | ·· |  | 14 | ·· | ·· |  | ·· | ·· | ·· |  |
| Liberia | Unknown | ·· |  | 16 | ·· | ·· |  | ·· | ·· | ·· |  |
| Madagascar | Unknown | ·· |  | 82 | ·· | ·· |  | ·· | ·· | ·· |  |
| Malawi | Unknown | ·· |  | 30 | ·· | ·· |  | ·· | ·· | ·· |  |
| Mali | Unknown | ·· |  | 60 | ·· | ·· |  | ·· | ·· | ·· |  |
| Mauritania | Unknown | ·· |  | 23 | ·· | ·· |  | ·· | ·· | ·· |  |
| Mauritius | Unknown | ·· |  | 11 | ·· | ·· |  | ·· | ·· | ·· |  |
| Mozambique | Unknown | ·· |  | 157 | ·· | ·· |  | ·· | ·· | ·· |  |
| Namibia | Unknown | ·· |  | 13 | ·· | ·· |  | ·· | ·· | ·· |  |
| Niger | Unknown | ·· |  | 41 | ·· | ·· |  | ·· | ·· | ·· |  |
| Nigeria | No | 2021 | 14 | 240 | ·· | ·· |  | ·· | ·· | ·· |  |
| Congo | Unknown | ·· |  | 12 | ·· | ·· |  | ·· | ·· | ·· |  |
| Rwanda | Unknown | ·· |  | 13 | ·· | ·· |  | ·· | ·· | ·· |  |
| Sao Tome & Principe | Unknown | ·· |  | 1 | ·· | ·· |  | ·· | ·· | ·· |  |
| Senegal | Unknown | ·· |  | 37 | ·· | ·· |  | ·· | ·· | ·· |  |
| Seychelles | Unknown | ·· |  | 3 | ·· | ·· |  | ·· | ·· | ·· |  |
| Sierra Leone | Unknown | ·· |  | 21 | ·· | ·· |  | ·· | ·· | ·· |  |
| Somalia | Unknown | ·· |  | 14 | ·· | ·· |  | ·· | ·· | ·· |  |
| South Africa | Unknown | ·· |  | 235 | ·· | ·· |  | ·· | ·· | ·· |  |
| United Republic of Tanzania | Unknown | ·· |  | 126 | ·· | ·· |  | ·· | ·· | ·· |  |
| Togo | Unknown | ·· |  | 13 | ·· | ·· |  | ·· | ·· | ·· |  |
| Uganda | Unknown | ·· |  | 254 | ·· | ·· |  | ·· | ·· | ·· |  |
| Zambia | Unknown | ·· |  | 90 | ·· | ·· |  | ·· | ·· | ·· |  |
| Zimbabwe | Unknown | ·· |  | 72 | ·· | ·· |  | ·· | ·· | ·· |  |
| **Middle East & North Africa** |  |  |  |  |  |  |  |  |  |  |  |
| Algeria | Unknown | ·· |  | 162 | ·· | ·· |  | ·· | ·· | ·· |  |
| Bahrain | Unknown | ·· |  | 4 | ·· | ·· |  | ·· | ·· | ·· |  |
| Cyprus | All facilities | 2022 | 2 | 1 | 1 | 2022 | 2 | 0 | 2020 | 2020 | 1 |
| Egypt | Unknown | ·· |  | 78 | ·· | ·· |  | ·· | ·· | ·· |  |
| Iraq | Unknown | ·· |  | 55 | ·· | ·· |  | ·· | ·· | ·· |  |
| Israel | Unknown | ·· |  | 30 | ·· | ·· |  | ·· | ·· | ·· |  |
| Jordan | Unknown | ·· |  | 17 | ·· | ·· |  | ·· | ·· | ·· |  |
| Kuwait | Unknown | ·· |  | 3 | ·· | ·· |  | ·· | ·· | ·· |  |
| Lebanon | Unknown | ·· |  | 25 | ·· | ·· |  | ·· | ·· | ·· |  |
| Libya | Unknown | ·· |  | 26 | ·· | ·· |  | ·· | ·· | ·· |  |
| Morocco | Unknown | ·· |  | 75 | ·· | ·· |  | ·· | ·· | ·· |  |
| Oman | Unknown | ·· |  | 3 | ·· | ·· |  | ·· | ·· | ·· |  |
| Occupied Palestinian territories | Unknown | ·· |  | N/A | ·· | ·· |  | ·· | ·· | ·· |  |
| Qatar | Unknown | ·· |  | 1 | ·· | ·· |  | ·· | ·· | ·· |  |
| Saudi Arabia | Unknown | ·· |  | 110 | ·· | ·· |  | ·· | ·· | ·· |  |
| South Sudan | Unknown | ·· |  | 80 | ·· | ·· |  | ·· | ·· | ·· |  |
| Sudan | Unknown | ·· |  | 125 | ·· | ·· |  | ·· | ·· | ·· |  |
| Syrian Arab Republic | Unknown | ·· |  | 35 | ·· | ·· |  | ·· | ·· | ·· |  |
| Tunisia | Unknown | ·· |  | 32 | ·· | ·· |  | ·· | ·· | ·· |  |
| Türkiye | Yes | 2022 | 2 | 403 | ·· | ·· |  | ·· | ·· | ·· |  |
| United Arab Emirates | Unknown | ·· |  | 21 | ·· | ·· |  | ·· | ·· | ·· |  |
| Yemen | Unknown | ·· |  | 43 | ·· | ·· |  | ·· | ·· | ·· |  |

**Notes:**

**Ref:** Reference; **HBV**: Hepatitis B.

***Total sites:** Including prisons, jails or other carceral setting, Country level data that informed these regional and global estimates were sourced from the World Prison Brief, collated by the Institute for Crime and Justice Policy Research at Burbeck University. See: <https://www.prisonstudies.org/world-prison-brief-data>;

** The total number of sites for England and Wales was reported together in the World Prison Brief, therefore the total number of sites for both England and Wales is reported as 120.

† For reporting purposes, these countries or territories are reported separately due to differences in service provision.

·· Indicates there were no data to inform a country’s estimate.

**References for Table 6.8**

1. World Health Organization. Status report on prison health in the WHO European Region 2022. Copenhagen, 2023.

2. European Union Drug Agency. EUDA Bulletin - Interventions in Prisons, Europe. 2024.

3. Harm Reduction International. Availability, accessibility, acceptability and quality of harm reduction services in Moldovan prisons. London, 2021.

4. Reitox Focal Point Austria, Gesundheit Österreich GmbH Vienna. Prison workbook 2023 - Austria. Austria; 2023.

5. European Union Drug Agency. EUDA Prison Data 2024. Lisbon; 2024.

6. Andreić J-L. Prison Workbook 2023 - Croatia, 2023.

7. UK Health Security Agency. Taking a place-based approach to tackling hepatitis in prisons. 2023.

8. Kugener T, Berndt N, Origer A, Debacker M. Prison workbook 2023 Luxembourg. 2023.

9. de Castro MV, Mendes SCC, Marques JPA, Santos AS, Duarte O. Prison Workbook 2023 - Portugal. 2023.

10. Gerlich MG, Frick U, Pirktl L, Uchtenhagen A. Detection and treatment of HIV and hepatitis virus infections in Swiss correctional facilities. *Int J Public Health* 2008.

11. Hariga F. Evaluation and Recommendations for the Improvement of the health Programmes, Including for the Prevention and Treatment of Drug Dependence and of HIV and AIDS, Implemented in the Establishments Under the Responsibility of the Federal Penitentiary Service in Argentina: a Follow-up of the 2008 Assessment. Vienna, Austria, 2011.

12. Instituto Nacional de Estadistica e Informatica. Perú: Primer Censo Nacional Penitenciario 2016. Perú, 2016.

13. Simpson PL, Gardoll B, White L, Butler T. HIV policies in Australian prisons: a structured review assessing compliance with international guidelines. *Lancet Reg Health West Pac* 2023; **41**.

14. Dan-Nwafor CC, Adeoye I, Aderemi K, et al. Serological markers and risk factors associated with Hepatitis B virus infection among Federal Capital Territory prison inmates, Nigeria: Should we be concerned? *PLoS One* 2021.

## *Appendix 6.9*: Regional and Country-level availability of interventions and programmatic data for HBV Vaccination

### *Appendix Table 6.9.1*: Regional availability of interventions for HBV Vaccination

|  | | **HBV Vaccination** | | | | | | | | | | | |
| --- | --- | --- | --- | --- | --- | --- | --- | --- | --- | --- | --- | --- | --- |
|  |  | **Countries Implementing in One or More Carceral Setting (% of)** | | | **Countries Implementing in ALL Carceral Settings (% of countries)** | | | **Countries NOT Implementing in Any Carceral Setting (% of countries)** | | | **Countries with UNKNOWN Availability in Carceral Settings (% of countries)** | | |
| **Region** | **Total Countries** | **Countries** | **% of Countries** | **% of incarcerated population** | **Countries** | **% of Countries** | **% of incarcerated population** | **Countries** | **% of Countries** | **% of incarcerated population** | **Countries** | **% of Countries** | **% of incarcerated population** |
| Eastern Europe | 17 | 10 | 59% | 26% | 8 | 47% | 22% | 4 | 24% | 5% | 3 | 18% | 69% |
| Western Europe | 33 | 24 | 73% | 94% | 21 | 64% | 90% | 2 | 6% | 3% | 7 | 21% | 3% |
| East & South-East Asia | 18 | 1 | 6% | 3% | 0 | 0% | 0% | 0 | 0% | 0% | 17 | 94% | 97% |
| South Asia | 9 | 0 | 0% | 0% | 0 | 0% | 0% | 0 | 0% | 0% | 9 | 100% | 100% |
| Central Asia | 5 | 0 | 0% | 0% | 0 | 0% | 0% | 0 | 0% | 0% | 5 | 100% | 100% |
| Caribbean | 15 | 0 | 0% | 0% | 0 | 0% | 0% | 0 | 0% | 0% | 15 | 100% | 100% |
| Latin America | 20 | 2 | 10% | 54% | 0 | 0% | 0% | 0 | 0% | 0% | 18 | 90% | 46% |
| North America | 2 | 1 | 50% | 98% | 0 | 0% | 0% | 0 | 0% | 0% | 1 | 50% | 2% |
| Pacific Island states and territories | 17 | 0 | 0% | 0% | 0 | 0% | 0% | 0 | 0% | 0% | 17 | 100% | 100% |
| Australasia | 2 | 1 | 50% | 82% | 0 | 0% | 0% | 0 | 0% | 0% | 1 | 50% | 18% |
| Sub-Saharan Africa | 47 | 0 | 0% | 0% | 0 | 0% | 0% | 1 | 2% | 8% | 46 | 98% | 92% |
| Middle East & North Africa | 22 | 2 | 9% | 38% | 1 | 5% | <1% | 0 | 0% | 0% | 20 | 91% | 62% |
| **Global** | **207** | **41** | **20%** | **35%** | **30** | **14%** | **5%** | **7** | **3%** | **1%** | **159** | **77%** | **64%** |

**Note.**

**HBV**: Hepatitis B.

### *Appendix Table 6.9.2*: Country-level availability of interventions and programmatic data for HBV Vaccination

|  |  |  |  | **Number of Carceral Sites with HBV Vaccination** | | | | **Number of People Accessing HBV Vaccination in Carceral Settings** | | | |
| --- | --- | --- | --- | --- | --- | --- | --- | --- | --- | --- | --- |
| **Country** | **Available** | **Year** | **Ref** | **Total sites*** | **N Sites with HBV Vaccination** | **Year** | **Ref** | **N People Accessing HBV Vaccination in Carceral Settings** | **Timeframe** | **Year** | **Ref** |
| **Eastern Europe** |  |  |  |  |  |  |  |  |  |  |  |
| Armenia | All facilities | 2020 | 1 | 12 | 12 | 2020 | 1 | ·· | ·· | ·· | ·· |
| Azerbaijan | Unknown | ·· |  | 53 | ·· | ·· |  | ·· | ·· | ·· | ·· |
| Belarus | Unknown | ·· |  | 67 | ·· | ·· |  | ·· | ·· | ·· | ·· |
| Bosnia & Herzegovina | All facilities | 2020 | 1 | 13 | 13 | 2020 | 1 | ·· | ·· | ·· | ·· |
| Bulgaria | No | 2022 | 2 | 55 | ·· | ·· |  | ·· | ·· | ·· | ·· |
| Czechia | All facilities | 2022 | 2 | 35 | 35 | 2022 | 2 | ·· | ·· | ·· | ·· |
| Estonia | All facilities | 2022 | 2 | 3 | 3 | 2022 | 2 | ·· | ·· | ·· | ·· |
| Georgia | All facilities | 2020 | 1 | 14 | 14 | 2020 | 1 | ·· | ·· | ·· | ·· |
| Hungary | Yes | 2022 | 2 | 30 | 11 | 2022 | 2 | ·· | ·· | ·· | ·· |
| Latvia | No | 2022 | 2 | 9 | ·· | ·· |  | ·· | ·· | ·· | ·· |
| Lithuania | No | 2022 | 2 | 8 | ·· | ·· |  | ·· | ·· | ·· | ·· |
| Republic of Moldova | All facilities | 2024 | 3 | 17 | 17 | 2024 | 3 | ·· | ·· | ·· | ·· |
| Poland | All facilities | 2022 | 2 | 215 | 215 | 2022 | 2 | ·· | ·· | ·· | ·· |
| Romania | No | 2021 | 4 | 45 | ·· | ·· |  | ·· | ·· | ·· | ·· |
| Russian Federation | Unknown | ·· |  | 872 | ·· | ·· |  | ·· | ·· | ·· | ·· |
| Slovakia | Yes | 2021 | 4 | 18 | ·· | ·· |  | ·· | ·· | ·· | ·· |
| Ukraine | All facilities | 2024 | 3 | 110 | 110 | 2024 | 3 | ·· | ·· | ·· | ·· |
| **Western Europe** |  |  |  |  | 0 |  |  | ·· |  |  |  |
| Albania | No | 2020 | 1 | 23 | ·· | ·· |  | ·· | ·· | ·· | ·· |
| Andorra | Unknown | ·· |  | 1 | ·· | ·· |  | ·· | ·· | ·· | ·· |
| Austria | No | 2023 | 5 | 27 | ·· | ·· |  | ·· | ·· | ·· | ·· |
| Belgium | All facilities | 2024 | 3 | 35 | 35 | 2024 | 3 | ·· | ·· | ·· | ·· |
| Croatia | All facilities | 2024 | 3 | 24 | 24 | 2024 | 3 | ·· | ·· | ·· | ·· |
| Denmark | All facilities | 2020 | 1 | 54 | 54 | 2020 | 1 | ·· | ·· | ·· | ·· |
| England** | All facilities | 2024 | 3 | 120 | 120 | 2024 | 3 | ·· | ·· | ·· | ·· |
| Finland | All facilities | 2024 | 3 | 26 | 26 | 2024 | 3 | ·· | ·· | ·· | ·· |
| France | All facilities | 2024 | 3 | 187 | 187 | 2024 | 3 | ·· | ·· | ·· | ·· |
| Germany | All facilities | 2022 | 4 | 179 | 179 | 2022 | 4 | ·· | ·· | ·· | ·· |
| Greece | Yes | 2020 | 1 | 34 | ·· | ·· |  | ·· | ·· | ·· | ·· |
| Greenland | Unknown | ·· |  | 6 | ·· | ·· |  | ·· | ·· | ·· | ·· |
| Iceland | Unknown | ·· |  | 5 | ·· | ·· |  | ·· | ·· | ·· | ·· |
| Ireland | All facilities | 2024 | 3 | 12 | 12 | 2024 | 3 | ·· | ·· | ·· | ·· |
| Italy | All facilities | 2024 | 3 | 206 | 206 | 2024 | 3 | ·· | ·· | ·· | ·· |
| Liechtenstein | Unknown | ·· |  | 1 | ·· | ·· |  | ·· | ·· | ·· | ·· |
| Luxembourg | Yes | 2024 | 3 | 3 | 1 | 2023 | 6 | 200 | Past year | 2021 | 7 |
| Malta | All facilities | 2024 | 3 | 1 | 1 | 2024 | 3 | ·· | ·· | ·· | ·· |
| Monaco | All facilities | 2020 | 1 | 2 | 2 | 2020 | 1 | ·· | ·· | ·· | ·· |
| Montenegro | Unknown | ·· |  | 3 | ·· | ·· |  | ·· | ·· | ·· | ·· |
| Netherlands | All facilities | 2024 | 3 | 49 | 49 | 2024 | 3 | ·· | ·· | ·· | ·· |
| North Macedonia | Unknown | ·· |  | 13 | ·· | ·· |  | ·· | ·· | ·· | ·· |
| Northern Ireland | All facilities | 2024 | 8 | 3 | 3 | 2024 | 8 | ·· | ·· | ·· | ·· |
| Norway | All facilities | 2024 | 3 | 33 | 33 | 2024 | 3 | ·· | ·· | ·· | ·· |
| Portugal | All facilities | 2024 | 3 | 49 | 49 | 2024 | 3 | ·· | ·· | ·· | ·· |
| San Marino | All facilities | 2020 | 1 | 1 | 1 | 2020 | 1 | ·· | ·· | ·· | ·· |
| Scotland | All facilities | 2024 | 8 | 15 | 15 | 2024 | 8 | ·· | ·· | ·· | ·· |
| Serbia | Unknown | ·· |  | 30 | ·· | ·· |  | ·· | ·· | ·· | ·· |
| Slovenia | All facilities | 2022 | 2 | 7 | 7 | 2022 | 2 | ·· | ·· | ·· | ·· |
| Spain | All facilities | 2022 | 2 | 82 | 82 | 2022 | 2 | ·· | ·· | ·· | ·· |
| Sweden | All facilities | 2024 | 3 | 79 | 79 | 2024 | 3 | ·· | ·· | ·· | ·· |
| Switzerland | Yes | 2020 | 1 | 88 | ·· | ·· |  | ·· | ·· | ·· | ·· |
| Wales** | All facilities | 2024 | 8 | 120 | 120 | 2024 | 8 | ·· | ·· | ·· | ·· |
| **East and South East Asia** |  |  |  |  |  |  |  |  |  |  |  |
| Brunei Darussalam | Unknown | ·· |  | 3 | ·· | ·· |  | ·· | ·· | ·· | ·· |
| Cambodia | Unknown | ·· |  | 29 | ·· | ·· |  | ·· | ·· | ·· | ·· |
| China | Unknown | ·· |  | 683 | ·· | ·· |  | ·· | ·· | ·· | ·· |
| Hong Kong | Unknown | ·· |  | 24 | ·· | ·· |  | ·· | ·· | ·· | ·· |
| Indonesia | Unknown | ·· |  | 526 | ·· | ·· |  | ·· | ·· | ·· | ·· |
| Japan | Unknown | ·· |  | 184 | ·· | ·· |  | ·· | ·· | ·· | ·· |
| Lao People's Democratic Republic | Unknown | ·· |  | 19 | ·· | ·· |  | ·· | ·· | ·· | ·· |
| Malaysia | Unknown | ·· |  | 43 | ·· | ·· |  | ·· | ·· | ·· | ·· |
| Mongolia | Unknown | ·· |  | 50 | ·· | ·· |  | ·· | ·· | ·· | ·· |
| Myanmar | Yes | 2019 | 9 | 96 | ·· | ·· |  | ·· | ·· | ·· | ·· |
| Democratic People's Republic of Korea | Unknown | ·· |  | N/A | ·· | ·· |  | ·· | ·· | ·· | ·· |
| Philippines | Unknown | ·· |  | 440 | ·· | ·· |  | ·· | ·· | ·· | ·· |
| Singapore | Unknown | ·· |  | 13 | ·· | ·· |  | ·· | ·· | ·· | ·· |
| Republic of Korea | Unknown | ·· |  | 54 | ·· | ·· |  | ·· | ·· | ·· | ·· |
| Taiwan | Unknown | ·· |  | 49 | ·· | ·· |  | ·· | ·· | ·· | ·· |
| Thailand | Unknown | ·· |  | 143 | ·· | ·· |  | ·· | ·· | ·· | ·· |
| Timor-Leste | Unknown | ·· |  | 3 | ·· | ·· |  | ·· | ·· | ·· | ·· |
| Viet Nam | Unknown | ·· |  | 54 | ·· | ·· |  | ·· | ·· | ·· | ·· |
| **South Asia** |  |  |  |  |  |  |  |  |  |  |  |
| Afghanistan | Unknown | ·· |  | 251 | ·· | ·· |  | ·· | ·· | ·· | ·· |
| Bangladesh | Unknown | ·· |  | 68 | ·· | ·· |  | ·· | ·· | ·· | ·· |
| Bhutan | Unknown | ·· |  | 7 | ·· | ·· |  | ·· | ·· | ·· | ·· |
| India | Unknown | ·· |  | 1330 | ·· | ·· |  | ·· | ·· | ·· | ·· |
| Islamic Republic of Iran | Unknown | ·· |  | 253 | ·· | ·· |  | ·· | ·· | ·· | ·· |
| Maldives | Unknown | ·· |  | 11 | ·· | ·· |  | ·· | ·· | ·· | ·· |
| Nepal | Unknown | ·· |  | 74 | ·· | ·· |  | ·· | ·· | ·· | ·· |
| Pakistan | Unknown | ·· |  | 116 | ·· | ·· |  | ·· | ·· | ·· | ·· |
| Sri Lanka | Unknown | ·· |  | 60 | ·· | ·· |  | ·· | ·· | ·· | ·· |
| **Central Asia** |  |  |  |  |  |  |  |  | ·· |  |  |
| Kazakhstan | Unknown | ·· |  | 80 | ·· | ·· |  | ·· | ·· | ·· | ·· |
| Kyrgyzstan | Unknown | ·· |  | 28 | ·· | ·· |  | ·· | ·· | ·· | ·· |
| Tajikistan | Unknown | ·· |  | 19 | ·· | ·· |  | ·· | ·· | ·· | ·· |
| Turkmenistan | Unknown | ·· |  | 27 | ·· | ·· |  | ·· | ·· | ·· | ·· |
| Uzbekistan | Unknown | ·· |  | 54 | ·· | ·· |  | ·· | ·· | ·· | ·· |
| **Caribbean** |  |  |  |  |  |  |  |  |  |  |  |
| Antigua & Barbuda | Unknown | ·· |  | 1 | ·· | ·· |  | ·· | ·· | ·· | ·· |
| Bahamas | Unknown | ·· |  | 1 | ·· | ·· |  | ·· | ·· | ·· | ·· |
| Barbados | Unknown | ·· |  | 1 | ·· | ·· |  | ·· | ·· | ·· | ·· |
| Bermuda | Unknown | ·· |  | 4 | ·· | ·· |  | ·· | ·· | ·· | ·· |
| Cuba | Unknown | ·· |  | 200 | ·· | ·· |  | ·· | ·· | ·· | ·· |
| Dominica | Unknown | ·· |  | 1 | ·· | ·· |  | ·· | ·· | ·· | ·· |
| Dominican Republic | Unknown | ·· |  | 41 | ·· | ·· |  | ·· | ·· | ·· | ·· |
| Grenada | Unknown | ·· |  | 1 | ·· | ·· |  | ·· | ·· | ·· | ·· |
| Haiti | Unknown | ·· |  | 17 | ·· | ·· |  | ·· | ·· | ·· | ·· |
| Jamaica | Unknown | ·· |  | 11 | ·· | ·· |  | ·· | ·· | ·· | ·· |
| Commonwealth of Puerto Rico | Unknown | ·· |  | 34 | ·· | ·· |  | ·· | ·· | ·· | ·· |
| Saint Kitts & Nevis | Unknown | ·· |  | 2 | ·· | ·· |  | ·· | ·· | ·· | ·· |
| Saint Lucia | Unknown | ·· |  | 1 | ·· | ·· |  | ·· | ·· | ·· | ·· |
| Saint Vincent & the Grenadines | Unknown | ·· |  | 2 | ·· | ·· |  | ·· | ·· | ·· | ·· |
| Trinidad & Tobago | Unknown | ·· |  | 9 | ·· | ·· |  | ·· | ·· | ·· | ·· |
| **Latin America** |  |  |  |  |  |  |  |  |  |  |  |
| Argentina | Yes | 2011 | 10 | 338 | ·· | ·· |  | ·· | ·· | ·· | ·· |
| Belize | Unknown | ·· |  | 1 | ·· | ·· |  | ·· | ·· | ·· | ·· |
| Plurinational State of Bolivia | Unknown | ·· |  | 40 | ·· | ·· |  | ·· | ·· | ·· | ·· |
| Brazil | Yes | 2023 | 11 | 1386 | ·· | ·· |  | ·· | ·· | ·· | ·· |
| Chile | Unknown | ·· |  | 113 | ·· | ·· |  | ·· | ·· | ·· | ·· |
| Colombia | Unknown | ·· |  | 125 | ·· | ·· |  | ·· | ·· | ·· | ·· |
| Costa Rica | Unknown | ·· |  | 32 | ·· | ·· |  | ·· | ·· | ·· | ·· |
| Ecuador | Unknown | ·· |  | 52 | ·· | ·· |  | ·· | ·· | ·· | ·· |
| El Salvador | Unknown | ·· |  | 25 | ·· | ·· |  | ·· | ·· | ·· | ·· |
| Guatemala | Unknown | ·· |  | 21 | ·· | ·· |  | ·· | ·· | ·· | ·· |
| Guyana | Unknown | ·· |  | 7 | ·· | ·· |  | ·· | ·· | ·· | ·· |
| Honduras | Unknown | ·· |  | 26 | ·· | ·· |  | ·· | ·· | ·· | ·· |
| Mexico | Unknown | ·· |  | 279 | ·· | ·· |  | ·· | ·· | ·· | ·· |
| Nicaragua | Unknown | ·· |  | 9 | ·· | ·· |  | ·· | ·· | ·· | ·· |
| Panama | Unknown | ·· |  | 23 | ·· | ·· |  | ·· | ·· | ·· | ·· |
| Paraguay | Unknown | ·· |  | 18 | ·· | ·· |  | ·· | ·· | ·· | ·· |
| Peru | Unknown | ·· |  | 68 | ·· | ·· |  | ·· | ·· | ·· | ·· |
| Suriname | Unknown | ·· |  | 21 | ·· | ·· |  | ·· | ·· | ·· | ·· |
| Uruguay | Unknown | ·· |  | 27 | ·· | ·· |  | ·· | ·· | ·· | ·· |
| Bolivarian Republic of Venezuela | Unknown | ·· |  | 41 | ·· | ·· |  | ·· | ·· | ·· | ·· |
| **North America** |  |  |  |  |  |  |  |  |  |  |  |
| Canada (Federal) | Unknown | ·· |  | 43 | ·· | ·· |  | ·· | ·· | ·· | ·· |
| Canada (Provincial/Territorial) | Unknown | ·· |  | 173 | ·· | ·· |  | ·· | ·· | ·· | ·· |
| United States of America (Federal) | Yes | 2000 | 12 | 102 | ·· | ·· |  | ·· | ·· | ·· | ·· |
| United States of America (State) | Yes | 2000 | 12 | 1190 | ·· | ·· |  | ·· | ·· | ·· | ·· |
| **Pacific Island States & Terr.** |  |  |  |  |  |  |  |  |  |  |  |
| American Samoa | Unknown | ·· |  | 1 | ·· | ·· |  | ·· | ·· | ·· | ·· |
| Federated States of Micronesia | Unknown | ·· |  | 4 | ·· | ·· |  | ·· | ·· | ·· | ·· |
| Fiji | Unknown | ·· |  | 15 | ·· | ·· |  | ·· | ·· | ·· | ·· |
| French Polynesia | Unknown | ·· |  | 2 | ·· | ·· |  | ·· | ·· | ·· | ·· |
| Guam | Unknown | ·· |  | 2 | ·· | ·· |  | ·· | ·· | ·· | ·· |
| Kiribati | Unknown | ·· |  | 4 | ·· | ·· |  | ·· | ·· | ·· | ·· |
| Marshall Islands | Unknown | ·· |  | 2 | ·· | ·· |  | ·· | ·· | ·· | ·· |
| Nauru | Unknown | ·· |  | 1 | ·· | ·· |  | ·· | ·· | ·· | ·· |
| New Caledonia | Unknown | ·· |  | 1 | ·· | ·· |  | ·· | ·· | ·· | ·· |
| Northern Mariana Islands | Unknown | ·· |  | 1 | ·· | ·· |  | ·· | ·· | ·· | ·· |
| Palau | Unknown | ·· |  | 1 | ·· | ·· |  | ·· | ·· | ·· | ·· |
| Papua New Guinea | Unknown | ·· |  | 18 | ·· | ·· |  | ·· | ·· | ·· | ·· |
| Samoa | Unknown | ·· |  | 3 | ·· | ·· |  | ·· | ·· | ·· | ·· |
| Solomon Islands | Unknown | ·· |  | 6 | ·· | ·· |  | ·· | ·· | ·· | ·· |
| Tonga | Unknown | ·· |  | 4 | ·· | ·· |  | ·· | ·· | ·· | ·· |
| Tuvalu | Unknown | ·· |  | 2 | ·· | ·· |  | ·· | ·· | ·· | ·· |
| Vanuatu | Unknown | ·· |  | 4 | ·· | ·· |  | ·· | ·· | ·· | ·· |
| **Australasia** |  |  |  |  |  |  |  |  |  |  |  |
| Australia | Yes | 2020 | 13 | 111 | ·· | ·· |  | ·· | ·· | ·· | ·· |
| New Zealand | Unknown | ·· |  | 18 | ·· | ·· |  | ·· | ·· | ·· | ·· |
| **Sub Saharan Africa** |  |  |  |  |  |  |  |  |  |  |  |
| Angola | Unknown | ·· |  | 47 | ·· | ·· |  | ·· | ·· | ·· | ·· |
| Benin | Unknown | ·· |  | 11 | ·· | ·· |  | ·· | ·· | ·· | ·· |
| Botswana | Unknown | ·· |  | 23 | ·· | ·· |  | ·· | ·· | ·· | ·· |
| Burkina Faso | Unknown | ·· |  | 27 | ·· | ·· |  | ·· | ·· | ·· | ·· |
| Burundi | Unknown | ·· |  | 13 | ·· | ·· |  | ·· | ·· | ·· | ·· |
| Cameroon | Unknown | ·· |  | 76 | ·· | ·· |  | ·· | ·· | ·· | ·· |
| Cabo Verde | Unknown | ·· |  | 5 | ·· | ·· |  | ·· | ·· | ·· | ·· |
| Central African Republic | Unknown | ·· |  | 15 | ·· | ·· |  | ·· | ·· | ·· | ·· |
| Chad | Unknown | ·· |  | 41 | ·· | ·· |  | ·· | ·· | ·· | ·· |
| Comoros | Unknown | ·· |  | 3 | ·· | ·· |  | ·· | ·· | ·· | ·· |
| Côte d'Ivoire | Unknown | ·· |  | 34 | ·· | ·· |  | ·· | ·· | ·· | ·· |
| Democratic Republic of the Congo | Unknown | ·· |  | 142 | ·· | ·· |  | ·· | ·· | ·· | ·· |
| Djibouti | Unknown | ·· |  | 1 | ·· | ·· |  | ·· | ·· | ·· | ·· |
| Equatorial Guinea | Unknown | ·· |  | 15 | ·· | ·· |  | ·· | ·· | ·· | ·· |
| Eritrea | Unknown | ·· |  | N/A | ·· | ·· |  | ·· | ·· | ·· | ·· |
| Eswatini | Unknown | ·· |  | 12 | ·· | ·· |  | ·· | ·· | ·· | ·· |
| Ethiopia | Unknown | ·· |  | 126 | ·· | ·· |  | ·· | ·· | ·· | ·· |
| Gabon | Unknown | ·· |  | 9 | ·· | ·· |  | ·· | ·· | ·· | ·· |
| Gambia | Unknown | ·· |  | 3 | ·· | ·· |  | ·· | ·· | ·· | ·· |
| Ghana | Unknown | ·· |  | 46 | ·· | ·· |  | ·· | ·· | ·· | ·· |
| Guinea | Unknown | ·· |  | 31 | ·· | ·· |  | ·· | ·· | ·· | ·· |
| Guinea-Bissau | Unknown | ·· |  | 3 | ·· | ·· |  | ·· | ·· | ·· | ·· |
| Kenya | Unknown | ·· |  | 134 | ·· | ·· |  | ·· | ·· | ·· | ·· |
| Lesotho | Unknown | ·· |  | 14 | ·· | ·· |  | ·· | ·· | ·· | ·· |
| Liberia | Unknown | ·· |  | 16 | ·· | ·· |  | ·· | ·· | ·· | ·· |
| Madagascar | Unknown | ·· |  | 82 | ·· | ·· |  | ·· | ·· | ·· | ·· |
| Malawi | Unknown | ·· |  | 30 | ·· | ·· |  | ·· | ·· | ·· | ·· |
| Mali | Unknown | ·· |  | 60 | ·· | ·· |  | ·· | ·· | ·· | ·· |
| Mauritania | Unknown | ·· |  | 23 | ·· | ·· |  | ·· | ·· | ·· | ·· |
| Mauritius | Unknown | ·· |  | 11 | ·· | ·· |  | ·· | ·· | ·· | ·· |
| Mozambique | Unknown | ·· |  | 157 | ·· | ·· |  | ·· | ·· | ·· | ·· |
| Namibia | Unknown | ·· |  | 13 | ·· | ·· |  | ·· | ·· | ·· | ·· |
| Niger | Unknown | ·· |  | 41 | ·· | ·· |  | ·· | ·· | ·· | ·· |
| Nigeria | No | 2021 | 14 | 240 | ·· | ·· |  | ·· | ·· | ·· | ·· |
| Congo | Unknown | ·· |  | 12 | ·· | ·· |  | ·· | ·· | ·· | ·· |
| Rwanda | Unknown | ·· |  | 13 | ·· | ·· |  | ·· | ·· | ·· | ·· |
| Sao Tome & Principe | Unknown | ·· |  | 1 | ·· | ·· |  | ·· | ·· | ·· | ·· |
| Senegal | Unknown | ·· |  | 37 | ·· | ·· |  | ·· | ·· | ·· | ·· |
| Seychelles | Unknown | ·· |  | 3 | ·· | ·· |  | ·· | ·· | ·· | ·· |
| Sierra Leone | Unknown | ·· |  | 21 | ·· | ·· |  | ·· | ·· | ·· | ·· |
| Somalia | Unknown | ·· |  | 14 | ·· | ·· |  | ·· | ·· | ·· | ·· |
| South Africa | Unknown | ·· |  | 235 | ·· | ·· |  | ·· | ·· | ·· | ·· |
| United Republic of Tanzania | Unknown | ·· |  | 126 | ·· | ·· |  | ·· | ·· | ·· | ·· |
| Togo | Unknown | ·· |  | 13 | ·· | ·· |  | ·· | ·· | ·· | ·· |
| Uganda | Unknown | ·· |  | 254 | ·· | ·· |  | ·· | ·· | ·· | ·· |
| Zambia | Unknown | ·· |  | 90 | ·· | ·· |  | ·· | ·· | ·· | ·· |
| Zimbabwe | Unknown | ·· |  | 72 | ·· | ·· |  | ·· | ·· | ·· | ·· |
| **Middle East & North Africa** |  |  |  |  |  |  |  |  |  |  |  |
| Algeria | Unknown | ·· |  | 162 | ·· | ·· |  | ·· | ·· | ·· | ·· |
| Bahrain | Unknown | ·· |  | 4 | ·· | ·· |  | ·· | ·· | ·· | ·· |
| Cyprus | All facilities | 2024 | 3 | 1 | 1 | 2024 | 3 | ·· | ·· | ·· | ·· |
| Egypt | Unknown | ·· |  | 78 | ·· | ·· |  | ·· | ·· | ·· | ·· |
| Iraq | Unknown | ·· |  | 55 | ·· | ·· |  | ·· | ·· | ·· | ·· |
| Israel | Unknown | ·· |  | 30 | ·· | ·· |  | ·· | ·· | ·· | ·· |
| Jordan | Unknown | ·· |  | 17 | ·· | ·· |  | ·· | ·· | ·· | ·· |
| Kuwait | Unknown | ·· |  | 3 | ·· | ·· |  | ·· | ·· | ·· | ·· |
| Lebanon | Unknown | ·· |  | 25 | ·· | ·· |  | ·· | ·· | ·· | ·· |
| Libya | Unknown | ·· |  | 26 | ·· | ·· |  | ·· | ·· | ·· | ·· |
| Morocco | Unknown | ·· |  | 75 | ·· | ·· |  | ·· | ·· | ·· | ·· |
| Oman | Unknown | ·· |  | 3 | ·· | ·· |  | ·· | ·· | ·· | ·· |
| Occupied Palestinian territories | Unknown | ·· |  | N/A | ·· | ·· |  | ·· | ·· | ·· | ·· |
| Qatar | Unknown | ·· |  | 1 | ·· | ·· |  | ·· | ·· | ·· | ·· |
| Saudi Arabia | Unknown | ·· |  | 110 | ·· | ·· |  | ·· | ·· | ·· | ·· |
| South Sudan | Unknown | ·· |  | 80 | ·· | ·· |  | ·· | ·· | ·· | ·· |
| Sudan | Unknown | ·· |  | 125 | ·· | ·· |  | ·· | ·· | ·· | ·· |
| Syrian Arab Republic | Unknown | ·· |  | 35 | ·· | ·· |  | ·· | ·· | ·· | ·· |
| Tunisia | Unknown | ·· |  | 32 | ·· | ·· |  | ·· | ·· | ·· | ·· |
| Türkiye | Yes | 2022 | 2 | 403 | ·· | ·· |  | ·· | ·· | ·· | ·· |
| United Arab Emirates | Unknown | ·· |  | 21 | ·· | ·· |  | ·· | ·· | ·· | ·· |
| Yemen | Unknown | ·· |  | 43 | ·· | ·· |  | ·· | ·· | ·· | ·· |

**Notes:**

**Ref:** Reference; **HBV**: Hepatitis B.

***Total sites:** Including prisons, jails or other carceral setting, Country level data that informed these regional and global estimates were sourced from the World Prison Brief, collated by the Institute for Crime and Justice Policy Research at Burbeck University. See: <https://www.prisonstudies.org/world-prison-brief-data>;

** The total number of sites for England and Wales was reported together in the World Prison Brief, therefore the total number of sites for both England and Wales is reported as 120.

·· Indicates there were no data to inform a country’s estimate.

† For reporting purposes, these countries or territories are reported separately due to differences in service provision.

**References for Table 6.9**

1. World Health Organization. Status report on prison health in the WHO European Region 2022. Copenhagen, 2023.

2. European Union Drug Agency. EUDA Bulletin - Interventions in Prisons, Europe. 2024.

3. Moazen B, Tramonti Fantozzi M, De Vita E, et al. Vaccines and vaccination in prison settings: availability and model of service delivery in 20 European countries. *BMC Public Health* 2024; **24(1)**: 2716.

4. Seyler T PF, Indave I, Giraudon I & Noor, A. Viral hepatitis elimination barometer among people who inject drugs in Europe. 2024. https://www.euda.europa.eu/publications/data-factsheet/viral-hepatitis-elimination-barometer-among-people-who-inject-drugs-in-europe_en.

5. Reitox Focal Point Austria, Gesundheit Österreich GmbH Vienna. Prison workbook 2023 - Austria. Austria; 2023.

6. Kugener T, Berndt N, Origer A, Debacker M. Prison workbook 2023 Luxembourg. 2023.

7. Berndt N SR, Kugener T, Origer A. National Drug Report 2022, 2022.

8. Hickman M, Willner, S & Edge C. UK Prison HBV and TB Coverage In: Santo Jr T, editor.; 2024.

9. Myanmar Ministry of Health and Sports. National Strategic Framework on Health and Drugs: Myanmar Ministry of Health and Sports, 2020.

10. Hariga F. Evaluation and Recommendations for the Improvement of the health Programmes, Including for the Prevention and Treatment of Drug Dependence and of HIV and AIDS, Implemented in the Establishments Under the Responsibility of the Federal Penitentiary Service in Argentina: a Follow-up of the 2008 Assessment. Vienna, Austria, 2011.

11. Marques JMdS, Matos MA, Silva ÁMC, et al. Prevalence of overt and occult hepatitis B virus infection among an incarcerated population of Central-Western Brazil. *Acta Trop* 2023; **241**: 106886.

12. Charuvastra A, Stein J, Schwartzapfel B, et al. Hepatitis B vaccination practices in state and federal prisons. *Public Health Rep* 2001.

13. Simpson PL, Gardoll B, White L, Butler T. HIV policies in Australian prisons: a structured review assessing compliance with international guidelines. *Lancet Reg Health West Pac* 2023; **41**.

14. Dan-Nwafor CC, Adeoye I, Aderemi K, et al. Serological markers and risk factors associated with Hepatitis B virus infection among Federal Capital Territory prison inmates, Nigeria: Should we be concerned? *PLoS One* 2021.

## *Appendix Table 6.10*: Country-level availability of interventions and programmatic data for Tuberculosis Screening

|  |  |  |  | **Number of Carceral Sites with Tuberculosis Screening** | | | | **Number of People Accessing Tuberculosis Screening in Carceral Settings** | | | |
| --- | --- | --- | --- | --- | --- | --- | --- | --- | --- | --- | --- |
| **Country** | **Available** | **Year** | **Ref** | **Total sites*** | **N Sites with Tuberculosis Screening** | **Year** | **Ref** | **N People Accessing Tuberculosis Screening in Carceral Settings** | **Timeframe** | **Year** | **Ref** |
| **Eastern Europe** |  |  |  |  |  |  |  |  |  |  |  |
| Armenia | All facilities | 2020 | 1 | 12 | 12 | 2020 | 1 | ·· | ·· | ·· |  |
| Azerbaijan | Yes | 2014 | 2 | 53 | ·· | ·· |  | ·· | ·· | ·· |  |
| Belarus | Unknown | ·· |  | 67 | ·· | ·· |  | ·· | ·· | ·· |  |
| Bosnia & Herzegovina | All facilities | 2020 | 1 | ·· | ·· | 2020 | 1 | ·· | ·· | ·· |  |
| Bulgaria | All facilities | 2021 | 3 | 55 | 55 | 2021 | 3 | 1883 | Snapshot | 2021 | 3 |
| Czechia | All facilities | 2020 | 1 | 35 | 35 | 2020 | 1 | ·· | ·· | ·· |  |
| Estonia | All facilities | 2020 | 1 | 3 | 3 | 2020 | 1 | ·· | ·· | ·· |  |
| Georgia | All facilities | 2020 | 1 | 14 | 14 | 2020 | 1 | ·· | ·· | ·· |  |
| Hungary | All facilities | 2020 | 1 | 30 | 30 | 2020 | 1 | ·· | ·· | ·· |  |
| Latvia | All facilities | 2020 | 1 | 9 | 9 | 2020 | 1 | ·· | ·· | ·· |  |
| Lithuania | All facilities | 2020 | 1 | 8 | 8 | 2020 | 1 | ·· | ·· | ·· |  |
| Republic of Moldova | All facilities | 2021 | 4 | 17 | 17 | 2021 | 4 | ·· | ·· | ·· |  |
| Poland | All facilities | 2020 | 1 | 215 | 215 | 2020 | 1 | ·· | ·· | ·· |  |
| Romania | All facilities | 2020 | 1 | 45 | 45 | 2020 | 1 | ·· | ·· | ·· |  |
| Russian Federation | Yes | 2022 | 5 | 872 | ·· | ·· |  | ·· | ·· | ·· |  |
| Slovakia | All facilities | 2020 | 1 | 18 | 18 | 2020 | 1 | ·· | ·· | ·· |  |
| Ukraine | All facilities | 2020 | 1 | 110 | 110 | 2020 | 1 | ·· | ·· | ·· |  |
| **Western Europe** |  |  |  |  |  |  |  |  |  |  |  |
| Albania | All facilities | 2020 | 1 | 23 | 23 | 2020 | 1 | ·· | ·· | ·· |  |
| Andorra | Unknown | ·· |  | 1 | ·· | ·· |  | ·· | ·· | ·· |  |
| Austria | All facilities | 2023 | 6 | 27 | 27 | 2023 | 6 | ·· | ·· | ·· |  |
| Belgium | All facilities | 2020 | 1 | 35 | 35 | 2020 | 1 | ·· | ·· | ·· |  |
| Croatia | All facilities | 2023 | 7 | 24 | 24 | 2023 | 7 | ·· | ·· | ·· |  |
| Denmark | All facilities | 2020 | 1 | 54 | 54 | 2020 | 1 | ·· | ·· | ·· |  |
| England** | All facilities | 2020 | 1 | 120 | 120 | 2020 | 1 | ·· | ·· | ·· |  |
| Finland | All facilities | 2020 | 1 | 26 | 26 | 2020 | 1 | ·· | ·· | ·· |  |
| France | All facilities | 2020 | 1 | 187 | 187 | 2020 | 1 | ·· | ·· | ·· |  |
| Germany | All facilities | 2020 | 1 | 179 | 179 | 2020 | 1 | ·· | ·· | ·· |  |
| Greece | All facilities | 2020 | 1 | 34 | 34 | 2020 | 1 | ·· | ·· | ·· |  |
| Greenland | Unknown | ·· |  | 6 | ·· | ·· |  | ·· | ·· | ·· |  |
| Iceland | Unknown | ·· |  | 5 | ·· | ·· |  | ·· | ·· | ·· |  |
| Ireland | All facilities | 2020 | 1 | 12 | 12 | 2020 | 1 | ·· | ·· | ·· |  |
| Italy | All facilities | 2020 | 1 | 206 | 206 | 2020 | 1 | ·· | ·· | ·· |  |
| Liechtenstein | Unknown | ·· |  | 1 | ·· | ·· |  | ·· | ·· | ·· |  |
| Luxembourg | Yes | 2023 | 8 | 3 | 1 | 2023 | 8 | ·· | ·· | ·· |  |
| Malta | All facilities | 2020 | 1 | 1 | 1 | 2020 | 1 | 667 | Other | 2019 | 9 |
| Monaco | All facilities | 2020 | 1 | 2 | 2 | 2020 | 1 | ·· | ·· | ·· |  |
| Montenegro | Unknown | ·· |  | 3 | ·· | ·· |  | ·· | ·· | ·· |  |
| Netherlands | All facilities | 2020 | 10 | 49 | 49 | 2020 | 10 | ·· | ·· | ·· |  |
| North Macedonia | Unknown | ·· |  | 13 | ·· | ·· |  | ·· | ·· | ·· |  |
| Northern Ireland | All facilities | 2024 | 11 | 3 | 3 | 2024 | 11 | ·· | ·· | ·· |  |
| Norway | Unknown | ·· |  | 33 | ·· | ·· |  | ·· | ·· | ·· |  |
| Portugal | All facilities | 2023 | 12 | 49 | 49 | 2023 | 12 | ·· | ·· | ·· |  |
| San Marino | All facilities | 2020 | 1 | 1 | 1 | 2020 | 1 | ·· | ·· | ·· |  |
| Scotland | All facilities | 2024 | 11 | 15 | 15 | 2024 | 11 | ·· | ·· | ·· |  |
| Serbia | Unknown | ·· |  | 30 | ·· | ·· |  | ·· | ·· | ·· |  |
| Slovenia | All facilities | 2020 | 1 | 7 | 7 | 2020 | 1 | ·· | ·· | ·· |  |
| Spain | All facilities | 2020 | 1 | 82 | 82 | 2020 | 1 | ·· | ·· | ·· |  |
| Sweden | Unknown | ·· |  | 79 | ·· | ·· |  | ·· | ·· | ·· |  |
| Switzerland | All facilities | 2020 | 1 | 88 | 88 | 2020 | 1 | ·· | ·· | ·· |  |
| Wales | All facilities | 2024 | 11 | 120 | 120 | 2024 | 11 | ·· | ·· | ·· |  |
| **East and South East Asia** |  |  |  |  |  |  |  |  | ·· |  |  |
| Brunei Darussalam | Unknown | ·· |  | 3 | ·· | ·· |  | ·· | ·· | ·· |  |
| Cambodia | Yes | 2013 | 13 | 29 | ·· | ·· |  | ·· | ·· | ·· |  |
| China | Yes | 2020 | 14 | 683 | ·· | ·· |  | ·· | ·· | ·· |  |
| Hong Kong | Unknown | ·· |  | 24 | ·· | ·· |  | ·· | ·· | ·· |  |
| Indonesia | Yes | 2022 | 15 | 526 | 26 | 2020 | 16 | ·· | ·· | ·· |  |
| Japan | Unknown | ·· |  | 184 | ·· | ·· |  | ·· | ·· | ·· |  |
| Lao People's Democratic Republic | Yes | 2020 | 17 | 19 | ·· | ·· |  | ·· | ·· | ·· |  |
| Malaysia | All facilities | 2016 | 18 | 43 | 43 | 2016 | 18 | ·· | ·· | ·· |  |
| Mongolia | All facilities | 2020 | 19 | 50 | 50 | 2020 | 19 | ·· | ·· | ·· |  |
| Myanmar | Yes | 2018 | 20 | 96 | ·· | ·· |  | ·· | ·· | ·· |  |
| Democratic People's Republic of Korea | Unknown | ·· |  | N/A | ·· | ·· |  | ·· | ·· | ·· |  |
| Philippines | Yes | 2019 | 21 | 440 | ·· | ·· |  | ·· | ·· | ·· |  |
| Singapore | Yes | 2005 | 22 | 13 | ·· | ·· |  | ·· | ·· | ·· |  |
| Republic of Korea | Yes | 2018 | 23 | 54 | ·· | ·· |  | ·· | ·· | ·· |  |
| Taiwan | Yes | 2022 | 24 | 49 | ·· | ·· |  | ·· | ·· | ·· |  |
| Thailand | Yes | 2023 | 25 | 143 | ·· | ·· |  | ·· | ·· | ·· |  |
| Timor-Leste | Yes | 2020 | 26 | 3 | ·· | ·· |  | ·· | ·· | ·· |  |
| Viet Nam | Yes | 2012 | 27 | 54 | ·· | ·· |  | ·· | ·· | ·· |  |
| **South Asia** |  |  |  |  |  |  |  |  | ·· |  |  |
| Afghanistan | Unknown | ·· |  | 251 | ·· | ·· |  | ·· | ·· | ·· |  |
| Bangladesh | Yes | 2015 | 28 | 68 | ·· | ·· |  | ·· | ·· | ·· |  |
| Bhutan | Unknown | ·· |  | 7 | ·· | ·· |  | ·· | ·· | ·· |  |
| India | Yes | 2023 | 29 | 1330 | 1262 | 2023 | 29 | 1009176 | Past year | 2023 | 29 |
| Islamic Republic of Iran | Yes | 2019 | 30 | 253 | ·· | ·· |  | ·· | ·· | ·· |  |
| Maldives | No | 2020 | 31 | 11 | ·· | ·· |  | ·· | ·· | ·· |  |
| Nepal | Unknown | ·· |  | 74 | ·· | ·· |  | ·· | ·· | ·· |  |
| Pakistan | Yes | 2010 | 32 | 116 | ·· | ·· |  | ·· | ·· | ·· |  |
| Sri Lanka | Yes | 2023 | 33 | 60 | ·· | ·· |  | ·· | ·· | ·· |  |
| **Central Asia** |  |  |  |  |  |  |  |  | ·· |  |  |
| Kazakhstan | Unknown | ·· |  | 80 | ·· | ·· |  | ·· | ·· | ·· |  |
| Kyrgyzstan | Yes | 2024 | 34 | 28 | ·· | ·· |  | ·· | ·· | ·· |  |
| Tajikistan | Unknown | ·· |  | 19 | ·· | ·· |  | ·· | ·· | ·· |  |
| Turkmenistan | Unknown | ·· |  | 27 | ·· | ·· |  | ·· | ·· | ·· |  |
| Uzbekistan | Unknown | ·· |  | 54 | ·· | ·· |  | ·· | ·· | ·· |  |
| **Caribbean** |  |  |  |  |  |  |  |  | ·· |  |  |
| Antigua & Barbuda | Unknown | ·· |  | 1 | ·· | ·· |  | ·· | ·· | ·· |  |
| Bahamas | Unknown | ·· |  | 1 | ·· | ·· |  | ·· | ·· | ·· |  |
| Barbados | Unknown | ·· |  | 1 | ·· | ·· |  | ·· | ·· | ·· |  |
| Bermuda | Unknown | ·· |  | 4 | ·· | ·· |  | ·· | ·· | ·· |  |
| Cuba | Unknown | ·· |  | 200 | ·· | ·· |  | ·· | ·· | ·· |  |
| Dominica | Unknown | ·· |  | 1 | ·· | ·· |  | ·· | ·· | ·· |  |
| Dominican Republic | Yes | 2024 | 35 | 41 | ·· | ·· |  | ·· | ·· | ·· |  |
| Grenada | Unknown | ·· |  | 1 | ·· | ·· |  | ·· | ·· | ·· |  |
| Haiti | Unknown | ·· |  | 17 | ·· | ·· |  | ·· | ·· | ·· |  |
| Jamaica | Unknown | ·· |  | 11 | ·· | ·· |  | ·· | ·· | ·· |  |
| Commonwealth of Puerto Rico | Unknown | ·· |  | 34 | ·· | ·· |  | ·· | ·· | ·· |  |
| Saint Kitts & Nevis | Unknown | ·· |  | 2 | ·· | ·· |  | ·· | ·· | ·· |  |
| Saint Lucia | Unknown | ·· |  | 1 | ·· | ·· |  | ·· | ·· | ·· |  |
| Saint Vincent & the Grenadines | Unknown | ·· |  | 2 | ·· | ·· |  | ·· | ·· | ·· |  |
| Trinidad & Tobago | Unknown | ·· |  | 9 | ·· | ·· |  | ·· | ·· | ·· |  |
| **Latin America** |  |  |  |  |  |  |  |  | ·· |  |  |
| Argentina | Yes | 2018 | 36 | 338 | ·· | ·· |  | ·· | ·· | ·· |  |
| Belize | Unknown | ·· |  | 1 | ·· | ·· |  | ·· | ·· | ·· |  |
| Plurinational State of Bolivia | Unknown | ·· |  | 40 | ·· | ·· |  | ·· | ·· | ·· |  |
| Brazil | Yes | 2020 | 37 | 1386 | ·· | ·· |  | ·· | ·· | ·· |  |
| Chile | Unknown | ·· |  | 113 | ·· | ·· |  | ·· | ·· | ·· |  |
| Colombia | Yes | 2013 | 38 | 125 | ·· | ·· |  | ·· | ·· | ·· |  |
| Costa Rica | Unknown | ·· |  | 32 | ·· | ·· |  | ·· | ·· | ·· |  |
| Ecuador | Yes | 2021 | 39 | 52 | ·· | ·· |  | ·· | ·· | ·· |  |
| El Salvador | Yes | 2016 | 40 | 25 | ·· | ·· |  | ·· | ·· | ·· |  |
| Guatemala | Yes | 2020 | 41 | 21 | ·· | ·· |  | ·· | ·· | ·· |  |
| Guyana | Unknown | ·· |  | 7 | ·· | ·· |  | ·· | ·· | ·· |  |
| Honduras | Unknown | ·· |  | 26 | ·· | ·· |  | ·· | ·· | ·· |  |
| Mexico | Yes | 2013 | 42 | 279 | ·· | ·· |  | ·· | ·· | ·· |  |
| Nicaragua | Unknown | ·· |  | 9 | ·· | ·· |  | ·· | ·· | ·· |  |
| Panama | Unknown | ·· |  | 23 | ·· | ·· |  | ·· | ·· | ·· |  |
| Paraguay | Yes | 2018 | 43 | 18 | ·· | ·· |  | ·· | ·· | ·· |  |
| Peru | Yes | 2024 | 44 | 68 | 3 | 2024 | 44 | ·· | ·· | ·· |  |
| Suriname | Unknown | ·· |  | 21 | ·· | ·· |  | ·· | ·· | ·· |  |
| Uruguay | Unknown | ·· |  | 27 | ·· | ·· |  | ·· | ·· | ·· |  |
| Bolivarian Republic of Venezuela | Unknown | ·· |  | 41 | ·· | ·· |  | ·· | ·· | ·· |  |
| **North America** |  |  |  |  |  |  |  |  | ·· |  |  |
| Canada (Federal) | All facilities | 2014 | 45 | 43 | 43 | 2014 | 45 | 17891 | Past year | 2008 | 46 |
| Canada (Provincial/Territorial) | Unknown | ·· |  | 173 | ·· | ·· |  | ·· | ·· | ·· |  |
| United States of America (Federal) | All facilities | 2001 | 47 | 102 | 102 | 2001 | 47 | ·· | ·· | ·· |  |
| United States of America (State) | Yes | 2012 | 48 | 1190 | ·· | ·· |  | 46875 | Snapshot | 2009 | 48 |
| **Pacific Island States & Terr.** |  |  |  |  |  |  |  |  | ·· |  |  |
| American Samoa | Unknown | ·· |  | 1 | ·· | ·· |  | ·· | ·· | ·· |  |
| Federated States of Micronesia | Unknown | ·· |  | 4 | ·· | ·· |  | ·· | ·· | ·· |  |
| Fiji | Unknown | ·· |  | 15 | ·· | ·· |  | ·· | ·· | ·· |  |
| French Polynesia | Unknown | ·· |  | 2 | ·· | ·· |  | ·· | ·· | ·· |  |
| Guam | Unknown | ·· |  | 2 | ·· | ·· |  | ·· | ·· | ·· |  |
| Kiribati | Unknown | ·· |  | 4 | ·· | ·· |  | ·· | ·· | ·· |  |
| Marshall Islands | Unknown | ·· |  | 2 | ·· | ·· |  | ·· | ·· | ·· |  |
| Nauru | Unknown | ·· |  | 1 | ·· | ·· |  | ·· | ·· | ·· |  |
| New Caledonia | Unknown | ·· |  | 1 | ·· | ·· |  | ·· | ·· | ·· |  |
| Northern Mariana Islands | Unknown | ·· |  | 1 | ·· | ·· |  | ·· | ·· | ·· |  |
| Palau | Unknown | ·· |  | 1 | ·· | ·· |  | ·· | ·· | ·· |  |
| Papua New Guinea | Unknown | ·· |  | 18 | ·· | ·· |  | ·· | ·· | ·· |  |
| Samoa | Unknown | ·· |  | 3 | ·· | ·· |  | ·· | ·· | ·· |  |
| Solomon Islands | Unknown | ·· |  | 6 | ·· | ·· |  | ·· | ·· | ·· |  |
| Tonga | Unknown | ·· |  | 4 | ·· | ·· |  | ·· | ·· | ·· |  |
| Tuvalu | Unknown | ·· |  | 2 | ·· | ·· |  | ·· | ·· | ·· |  |
| Vanuatu | Unknown | ·· |  | 4 | ·· | ·· |  | ·· | ·· | ·· |  |
| **Australasia** |  |  |  |  |  |  |  |  | ·· |  |  |
| Australia | Yes | 2020 | 49 | 111 | ·· | ·· |  | ·· | ·· | ·· |  |
| New Zealand | Unknown | ·· |  | 18 | ·· | ·· |  | ·· | ·· | ·· |  |
| **Sub Saharan Africa** |  |  |  |  |  |  |  |  | ·· |  |  |
| Angola | Unknown | ·· |  | 47 | ·· | ·· |  | ·· | ·· | ·· |  |
| Benin | Unknown | ·· |  | 11 | ·· | ·· |  | ·· | ·· | ·· |  |
| Botswana | Unknown | ·· |  | 23 | ·· | ·· |  | ·· | ·· | ·· |  |
| Burkina Faso | Unknown | ·· |  | 27 | ·· | ·· |  | ·· | ·· | ·· |  |
| Burundi | Unknown | ·· |  | 13 | ·· | ·· |  | ·· | ·· | ·· |  |
| Cameroon | Yes | 2021 | 50 | 76 | ·· | ·· |  | ·· | ·· | ·· |  |
| Cabo Verde | Unknown | ·· |  | 5 | ·· | ·· |  | ·· | ·· | ·· |  |
| Central African Republic | Unknown | ·· |  | 15 | ·· | ·· |  | ·· | ·· | ·· |  |
| Chad | Yes | 2024 | 51 | 41 | ·· | ·· |  | ·· | ·· | ·· |  |
| Comoros | Unknown | ·· |  | 3 | ·· | ·· |  | ·· | ·· | ·· |  |
| Côte d'Ivoire | Yes | 2017 | 52 | 34 | ·· | ·· |  | ·· | ·· | ·· |  |
| Democratic Republic of the Congo | Unknown | ·· |  | 142 | ·· | ·· |  | ·· | ·· | ·· |  |
| Djibouti | Unknown | ·· |  | 1 | ·· | ·· |  | ·· | ·· | ·· |  |
| Equatorial Guinea | Unknown | ·· |  | 15 | ·· | ·· |  | ·· | ·· | ·· |  |
| Eritrea | Unknown | ·· |  | N/A | ·· | ·· |  | ·· | ·· | ·· |  |
| Eswatini | Yes | 2024 | 53 | 12 | ·· | ·· |  | ·· | ·· | ·· |  |
| Ethiopia | Yes | 2019 | 54 | 126 | ·· | ·· |  | ·· | ·· | ·· |  |
| Gabon | Unknown | ·· |  | 9 | ·· | ·· |  | ·· | ·· | ·· |  |
| Gambia | Yes | 2022 | 55 | 3 | ·· | ·· |  | ·· | ·· | ·· |  |
| Ghana | Yes | 2014 | 56 | 46 | ·· | ·· |  | ·· | ·· | ·· |  |
| Guinea | Unknown | ·· |  | 31 | ·· | ·· |  | ·· | ·· | ·· |  |
| Guinea-Bissau | Unknown | ·· |  | 3 | ·· | ·· |  | ·· | ·· | ·· |  |
| Kenya | Unknown | ·· |  | 134 | ·· | ·· |  | ·· | ·· | ·· |  |
| Lesotho | Unknown | ·· |  | 14 | ·· | ·· |  | ·· | ·· | ·· |  |
| Liberia | Unknown | ·· |  | 16 | ·· | ·· |  | ·· | ·· | ·· |  |
| Madagascar | Yes | 2000 | 57 | 82 | ·· | ·· |  | ·· | ·· | ·· |  |
| Malawi | Yes | 2020 | 58 | 30 | ·· | ·· |  | ·· | ·· | ·· |  |
| Mali | Unknown | ·· |  | 60 | ·· | ·· |  | ·· | ·· | ·· |  |
| Mauritania | Unknown | ·· |  | 23 | ·· | ·· |  | ·· | ·· | ·· |  |
| Mauritius | Unknown | ·· |  | 11 | ·· | ·· |  | ·· | ·· | ·· |  |
| Mozambique | Unknown | ·· |  | 157 | ·· | ·· |  | ·· | ·· | ·· |  |
| Namibia | Unknown | ·· |  | 13 | ·· | ·· |  | ·· | ·· | ·· |  |
| Niger | Yes | 2021 | 59 | 41 | ·· | ·· |  | ·· | ·· | ·· |  |
| Nigeria | Yes | 2015 | 60 | 240 | ·· | ·· |  | ·· | ·· | ·· |  |
| Congo | Unknown | ·· |  | 12 | ·· | ·· |  | ·· | ·· | ·· |  |
| Rwanda | Yes | 2017 | 61 | 13 | 8 | 2017 | 61 | 70083 | Past year | 2016 | 61 |
| Sao Tome & Principe | Unknown | ·· |  | 1 | ·· | ·· |  | ·· | ·· | ·· |  |
| Senegal | Yes | 2023 | 62 | 37 | ·· | ·· |  | ·· | ·· | ·· |  |
| Seychelles | Unknown | ·· |  | 3 | ·· | ·· |  | ·· | ·· | ·· |  |
| Sierra Leone | Yes | 2023 | 63 | 21 | ·· | ·· |  | ·· | ·· | ·· |  |
| Somalia | Yes | 2019 | 64 | 14 | ·· | ·· |  | ·· | ·· | ·· |  |
| South Africa | All facilities | 2021 | 65 | 235 | 235 | 2021 | 65 | ·· | ·· | ·· |  |
| United Republic of Tanzania | Yes | 2014 | 66 | 126 | ·· | ·· |  | ·· | ·· | ·· |  |
| Togo | Unknown | ·· |  | 13 | ·· | ·· |  | ·· | ·· | ·· |  |
| Uganda | All facilities | 2024 | 67 | 254 | 254 | 2024 | 67 | ·· | ·· | ·· |  |
| Zambia | Yes | 2016 | 68 | 90 | 6 | 2011 | 69 | ·· | ·· | ·· |  |
| Zimbabwe | Yes | 2020 | 70 | 72 | ·· | ·· |  | ·· | ·· | ·· |  |
| **Middle East & North Africa** |  |  |  |  |  |  |  |  | ·· |  |  |
| Algeria | Unknown | ·· |  | 162 | ·· | ·· |  | ·· | ·· | ·· |  |
| Bahrain | Unknown | ·· |  | 4 | ·· | ·· |  | ·· | ·· | ·· |  |
| Cyprus | All facilities | 2020 | 1 | 1 | 1 | 2020 | 1 | ·· | ·· | ·· |  |
| Egypt | Yes | 2019 | 71 | 78 | 3 | 2019 | 71 | ·· | ·· | ·· |  |
| Iraq | Unknown | ·· |  | 55 | ·· | ·· |  | ·· | ·· | ·· |  |
| Israel | Unknown | ·· |  | 30 | ·· | ·· |  | ·· | ·· | ·· |  |
| Jordan | Unknown | ·· |  | 17 | ·· | ·· |  | ·· | ·· | ·· |  |
| Kuwait | Unknown | ·· |  | 3 | ·· | ·· |  | ·· | ·· | ·· |  |
| Lebanon | Unknown | ·· |  | 25 | ·· | ·· |  | ·· | ·· | ·· |  |
| Libya | Unknown | ·· |  | 26 | ·· | ·· |  | ·· | ·· | ·· |  |
| Morocco | Unknown | ·· |  | 75 | ·· | ·· |  | ·· | ·· | ·· |  |
| Oman | Unknown | ·· |  | 3 | ·· | ·· |  | ·· | ·· | ·· |  |
| Occupied Palestinian territories | Unknown | ·· |  | N/A | ·· | ·· |  | ·· | ·· | ·· |  |
| Qatar | Unknown | ·· |  | 1 | ·· | ·· |  | ·· | ·· | ·· |  |
| Saudi Arabia | Unknown | ·· |  | 110 | ·· | ·· |  | ·· | ·· | ·· |  |
| South Sudan | Unknown | ·· |  | 80 | ·· | ·· |  | ·· | ·· | ·· |  |
| Sudan | Unknown | ·· |  | 125 | ·· | ·· |  | ·· | ·· | ·· |  |
| Syrian Arab Republic | Unknown | ·· |  | 35 | ·· | ·· |  | ·· | ·· | ·· |  |
| Tunisia | Unknown | ·· |  | 32 | ·· | ·· |  | ·· | ·· | ·· |  |
| Türkiye | Yes | 2013 | 72 | 403 | ·· | ·· |  | ·· | ·· | ·· |  |
| United Arab Emirates | Unknown | ·· |  | 21 | ·· | ·· |  | ·· | ·· | ·· |  |
| Yemen | Unknown | ·· |  | 43 | ·· | ·· |  | ·· | ·· | ·· |  |

**Notes:**

**Ref:** Reference.

***Total sites:** Including prisons, jails or other carceral setting, Country level data that informed these regional and global estimates were sourced from the World Prison Brief, collated by the Institute for Crime and Justice Policy Research at Burbeck University. See: <https://www.prisonstudies.org/world-prison-brief-data>;

** The total number of sites for England and Wales was reported together in the World Prison Brief, therefore the total number of sites for both England and Wales is reported as 120.

·· Indicates there were no data to inform a country’s estimate.

† For reporting purposes, these countries or territories are reported separately due to differences in service provision.

**References for Table 6.10**

1. World Health Organization. Status report on prison health in the WHO European Region 2022. Copenhagen, 2023.

2. Burrows D, Boltaev A, Musa M, Gottfreðsson M, Suleymanova J. Review of the HIV programme in Azerbaijan: November 2014: World Health Organization. Regional Office for Europe, 2016.

3. Kanev KA, D. The Reform of The Bulgarian Penitentiary System: Current State and Future Prospects. Sofia, 2024.

4. Harm Reduction International. Availability, accessibility, acceptability and quality of harm reduction services in Moldovan prisons. London, 2021.

5. Kolomiets VM, Polshikova NA, Petrov AYU, Kovalenko AL, Talikova EV. Epidemic Situation and Features of Accompanying Therapy in the Treatment of Socially Significant Infectious Diseases in Penitentiary Populations Before, During, and after the COVID-19 Pandemic. [Russian]. *Antibiotiki i Khimioterapiya* 2024; **69(1-2)**: 44-50.

6. Reitox Focal Point Austria, Gesundheit Österreich GmbH Vienna. Prison workbook 2023 - Austria. Austria; 2023.

7. Andreić J-L. Prison Workbook 2023 - Croatia, 2023.

8. Kugener T, Berndt N, Origer A, Debacker M. Prison workbook 2023 Luxembourg. 2023.

9. Muscat K, Cremona C, Fenech TM, Abela M, Padovese V. Sexually transmitted infections epidemiology and risk assessment at the main correctional facility in Malta (2017-2019). *J Eur Acad Dermatol Venereol* 2022.

10. de Vries G, Commandeur S, Erkens C, et al. Towards selective tuberculosis screening of people in prison in a low-incidence country. *Eur Respir J* 2019.

11. Hickman M, Willner, S & Edge C. UK Prison HBV and TB Coverage In: Santo Jr T, editor.; 2024.

12. de Castro MV, Mendes SCC, Marques JPA, Santos AS, Duarte O. Prison Workbook 2023 - Portugal. 2023.

13. World Health Organization Western Pacific Region. Joint Review of the Cambodian National Health Sector Response to HIV 2013, 2013.

14. Zhang GQ, Zhang YH, Zhong D, et al. High Prevalence of and Risk Factors for Latent Tuberculosis Infection among Prisoners, Tianjin, China. *Emerg Infect Dis* 2020.

15. Kementerian Keshehatan Republik Indonesia WHoI. Indonesia TB joint external monitoring mission (JEMM) report, 2022.

16. Control DGoDPa. Tuberculosis contl in Indonesia 2022 - Ministry of Health Republic of Indonesia, 2022.

17. LAO PDR Ministry of Health. LAO PDR National Tuberculosis Strategic Plan 2021-2025, 2020.

18. Al-Darraji HAA, Altice FL, Kamarulzaman A. Undiagnosed pulmonary tuberculosis among prisoners in Malaysia: An overlooked risk for tuberculosis in the community. *Trop Med Int Health* 2016.

19. The Global Fund. Technical Brief: Addresing HIV and TB in Prisons, Pre-Trial Detention and Other Closed Settings, 2020.

20. Myanmar Ministry of Health and Sports. National Strategic Framework on Health and Drugs: Myanmar Ministry of Health and Sports, 2020.

21. Health Do. 2019 Philippines TB joint program review, 2019.

22. Chee CB, Teleman MD, Boudville IC, Wang YT. Contact screening and latent TB infection treatment in Singapore correctional facilities. *Int J Tuberc Lung Dis* 2005.

23. Go U, Park M, Kim UN, et al. Tuberculosis prevention and care in Korea: Evolution of policy and practice. *J Clin Tuberc Other Mycobact Dis* 2018; **11**: 28-36.

24. Department of Health and Welfare CfDC. Tuberculosis Diagnosis and Treatment Guidelines - Taiwan Guidelines for TB Diagnosis & Treatment, 2022.

25. Department of Disease Control Ministry of Public Health - Division of Tuberculosis. Thailand Operational Plan To End Tuberculosis, Phase 2 (2023 - 2027). 2023.

26. Ministry of Health WHOT-l. National Strategic Plan for Ending TB 2020-2024, 2020.

27. Nhung NV, Hoa NB, Khanh PH, Hennig C. Tuberculosis case notification data in Viet Nam, 2007 to 2012. *Western Pac Surveill Response J* 2015; **6**(1): 7-14.

28. Banu S, Rahman MT, Uddin MKM, et al. Effect of active case finding on prevalence and transmission of pulmonary tuberculosis in Dhaka Central Jail, Bangladesh. *PLoS One* 2015.

29. National AIDS Control Organisation. Sankalak: Status of National AIDS & STD Response (Fifth edition, 2023). New Delhi: NACO, Ministry of Health and Family Welfare, Government of India., 2023.

30. Farhoudi B, SeyedAlinaghi S, Dadras O, et al. Health service provision for disease control among prisoners: a conceptual note. *J Health Res* 2020; **34**(4): 353-8.

31. WHO. Republic of the Maldives National Strategic Plan for TB Control 2015-2020 2020.

32. Kazi AM, Shah SA, Jenkins CA, Shepherd BE, Vermund SH. Risk factors and prevalence of tuberculosis, human immunodeficiency virus, syphilis, hepatitis B virus, and hepatitis C virus among prisoners in Pakistan. *Int J Infect Dis* 2010.

33. Ministry of Health SL. National programme for tuberculosis control & chest diseases 2023.

34. Frontline AIDS. Harm reduction services for drug users in Kyrgyzstan. 2024; 2024.

35. Procuraduría General de la República. DGSPC realiza segunda jornada de detección de tuberculosis en CCR El Pinito. 2014. https://pgr.gob.do/dgspc-realiza-segunda-jornada-de-deteccion-de-tuberculosis-en-ccr-el-pinito/.

36. Biasutti F, Rossi E, Moro A, et al. Study of tuberculosis cases registered among prisoners in South Santa Fe, Argentina. *J Int AIDS Soc Conference: HIV and Hepatitis in the Americas* 2019.

37. Bezerra da Silva Junior G, Vasconcelos C, Furtado J, et al. Genitourinary tuberculosis in prisoners in Brazil, 2010 - 2020. *Kidney Int Rep* 2024; **9**(4 Supplement): S648.

38. Rueda ZV, Lopez L, Velez LA, et al. High incidence of tuberculosis, low sensitivity of current diagnostic scheme and prolonged culture positivity in four Colombian prisons. A cohort study. *PLoS One* 2013.

39. Valcarcel-Perez I, Molina JL, Fuentes Z. Is mass screening enough to control tuberculosis in Ecuador's prisons? *Rev Esp Sanid Penit* 2021.

40. Ayala G, Garay J, Aragon M, Decroo T, Zachariah R. Trends in tuberculosis notification and treatment outcomes in prisons: a country-wide assessment in El Salvador from 2009–2014. *Rev Panam Salud Publica;39(1),ene 2016* 2016.

41. Collaboration PIoHMaEIDR. Global Fund Prospective Country Evaluation: 2019-2020 Guatemala Annual country report, 2020.

42. Joya M, Avilés, MAG, Saldaña, DA & Olivares LM. National TB Strategy in the prisons - 2014… the year of detection, treatment and cure of tuberculosis patients. 2014.

43. Sequera VG, Aguirre S, Estigarribia G, et al. Increased incarceration rates drive growing tuberculosis burden in prisons and jeopardize overall tuberculosis control in Paraguay. *Sci Rep* 2020.

44. Faust L, Caceres-Cardenas G, Martinez L, et al. Tuberculosis case notifications and outcomes in Peruvian prisons prior to and during the COVID-19 pandemic: a national-level interrupted time series analysis. *Lancet Reg Health Am* 2024; **33**.

45. Schwartz IS, Bach PJ, Roscoe B, et al. Interferon-gamma release assays piloted as a latent tuberculous infection screening tool in Canadian federal inmates. *Int J Tuberc Lung Dis* 2014.

46. Correctional Service of Canada. Infectious disease surveillance in Canadian federal penitentiaries 2007–2008: pre-release report. Correctional Service of Canada Ottawa, ON; 2012.

47. Saunders DL, Olive DM, Wallace SB, Lacy DFB, Leyba R, Kendig NE. Tuberculosis screening in the federal prison system: An opportunity to treat and prevent tuberculosis in foreign-born populations. *Public Health Rep* 2001.

48. Weant TE, Turner AN, Murphy-Weiss M, Murray DM, Wang SH. Can social history variables predict prison inmates' risk for latent tuberculosis infection? *Tuberc Res Treat* 2012; **2012**: 132406.

49. Simpson PL, Gardoll B, White L, Butler T. HIV policies in Australian prisons: a structured review assessing compliance with international guidelines. *Lancet Reg Health West Pac* 2023; **41**.

50. Noeske J, Ndi NF, Nga FHM, Mely G, Kuaban C. Prison health services across ten central prisons in Cameroon. *J Public Health Afr* 2023; **14**(9).

51. Ahmat AI, Hassan MTN, Richard NBN, et al. Tuberculosis in the Carcinal Environment in Chad Due to the Mycobacterium Tuberculosis Circulante Complex. *J Biosci Med* 2024; **12**(02): 214-24.

52. Seri B, Koffi A, Danel C, et al. Prevalence of pulmonary tuberculosis among prison inmates: A cross-sectional survey at the Correctional and Detention Facility of Abidjan, Cote d'Ivoire. *PLoS One* 2017.

53. Agency CID. Swaziland NTP (National Tuberculosis Control Programme). 2024.

54. Sahle ET, Blumenthal J, Jain S, et al. Bacteriologically-confirmed pulmonary tuberculosis in an Ethiopian prison: Prevalence from screening of entrant and resident prisoners. *PLoS One* 2019.

55. Ministry of Health The Gambia. Annual Report on TB & Leprosy in The Gambia (2022) 2022.

56. Bonsu F, Hanson-Nortey N, Afutu F, et al. The National Tuberculosis Health Sector Strategic Plan for Ghana 2015–2020, October 2014.

57. Rasolofo-Razanamparany V, Menard D, Ratsitorahina M, Auregan G, Gicquel B, Chanteau S. Transmission of tuberculosis in the prison of Antananarivo (Madagascar). *Res Microbiol* 2000.

58. Singano V, Kip E, Ching'ani W, Chiwaula L. Tuberculosis treatment outcomes among prisoners and general population in Zomba, Malawi. *BMC Public Health* 2020.

59. Sani K, Laouali HAM, Laminou AM, et al. Tuberculose pulmonaire en milieu carcéral au Niger: Aspects épidémiologiques, diagnostiques, thérapeutiques et évolutifs. *Eur Sci J* 2021; **17**(1).

60. Ekundayo EO, Onuka O, Mustapha G, Geoffrey M. Active case finding of pulmonary tuberculosis among prison inmates in aba Federal prison, Abia state, Nigeria. *Adv Infect Dis* 2015; **5**(01): 57.

61. Institute of HIV/AIDS Disease Prevention and Control. Tuberculosis National Strategic Plan 2013-2018 Extension 2018-2020, 2017.

62. Ministre de la Santé et de l’Action sociale. Plan strategique national pour une riposte multisectorielle integree contre le SIDA, la tuberculose, les hepatites virales et les IST, 2023.

63. Pape S, Ngaujah SDT, Gulma K, Shivalli S, de Kiev LC. Tuberculosis screening on admission to Sierra Leone’s correctional facilities: A SWOC analysis. 2023.

64. MInistry of Health S. Somali National Strategic Plan For Tuberculosis Control 2015-2019, 2019.

65. Velen K, Sathar F, Hoffmann CJ, et al. Digital Chest X-Ray with Computer-aided Detection for Tuberculosis Screening within Correctional Facilities. *Ann Am Thorac Soc* 2022.

66. Mangu C, van den Hombergh, J, Kowour, D, Maboko, L, Kasang, C, Malewa, J, Hoelscher, M & Clowes, R. . TB burden in Tanzanian prisons: active screening with X-pert MTB/RifW assay and establishment of associated characteristics for MTB infection. 45th World Conference on Lung Health of the International Union Against Tuberculosis and Lung Disease (The Union). BARCELONA; 2014.

67. Lukoye D, Kalamya JN, Awor AC, et al. Treatment Outcomes for Tuberculosis Infection and Disease Among Persons Deprived of Liberty, Uganda, 2020. *Emerg Infect Dis* 2024; **30**(7): 1402EP-5.

68. Crosby R, Salazar LF, DiClemente RJ, Yarber WL, Caliendo AM, Staples-Horne M. Health risk factors among detained adolescent females. *Am J Prev Med* 2004.

69. Henostroza G, Harris JB, Kancheya N, et al. Chest radiograph reading and recording system: evaluation in frontline clinicians in Zambia. *BMC Infect Dis* 2016.

70. Mandizvidza A, Dlodlo RA, Chinnakali P, et al. Tuberculosis Case Finding Cascade and Treatment Outcomes among Male Inmates in Two Prisons in Zimbabwe. *Tuberc res treat* 2020; **2020**(1): 5829471.

71. United Nations Office on Drugs and Crime ROMENA. Egypt: UNODC inaugurates first-ever prison Voluntary Confidential Counseling, Testing and aftercare services. 2019.

72. Borekci S, Ongen G, Icmeli OS, et al. Pulmonary tuberculosis incidence in Turkish prisons: Importance of screening and case finding strategies. *Tuberkuloz ve Toraks* 2013.

## *Appendix Table 6.11*: Country-level availability of interventions and programmatic data for tuberculosis treatment

|  |  |  |  | **Number of Carceral Sites with Tuberculosis Treatment** | | | | **Number of People Accessing Tuberculosis Treatment in Carceral Settings** | | | |
| --- | --- | --- | --- | --- | --- | --- | --- | --- | --- | --- | --- |
| **Country** | **Available** | **Year** | **Ref** | **Total sites*** | **N Sites with Tuberculosis Treatment** | **Year** | **Ref** | **N People Accessing Tuberculosis Treatment in Carceral Settings** | **Timeframe** | **Year** | **Ref** |
| **Eastern Europe** |  |  |  |  |  |  |  |  |  |  |  |
| Armenia | Yes | 2020 | 1 | 12 | ·· | ·· |  | 6 | Snapshot | 2020 | 1 |
| Azerbaijan | Yes | 2014 | 2 | 53 | ·· | ·· |  | ·· | ·· | ·· |  |
| Belarus | Unknown | ·· |  | 67 | ·· | ·· |  | ·· | ·· | ·· |  |
| Bosnia & Herzegovina | Yes | 2020 | 1 | 13 | ·· | ·· |  | 4 | Snapshot | 2020 | 1 |
| Bulgaria | Yes | 2024 | 3 | 55 | ·· | ·· |  | 8 | Snapshot | 2020 | 1 |
| Czechia | Yes | 2020 | 4 | 35 | ·· | ·· |  | 3 | Snapshot | 2020 | 1 |
| Estonia | Yes | 2020 | 1 | 3 | ·· | ·· |  | 5 | Snapshot | 2020 | 1 |
| Georgia | Yes | 2020 | 1 | 14 | ·· | ·· |  | 47 | Snapshot | 2020 | 1 |
| Hungary | Yes | 2020 | 1 | 30 | ·· | ·· |  | 24 | Past year | 2020 | 1 |
| Latvia | Yes | 2020 | 1 | 9 | ·· | ·· |  | 20 | Snapshot | 2020 | 1 |
| Lithuania | Yes | 2020 | 1 | 8 | ·· | ·· |  | 43 | Past year | 2020 | 1 |
| Republic of Moldova | All facilities | 2021 | 5 | 17 | 17 | 2021 | 5 | 97 | Past year | 2020 | 1 |
| Poland | Yes | 2020 | 4 | 215 | ·· | ·· |  | ·· | ·· | ·· |  |
| Romania | Yes | 2020 | 1 | 45 | ·· | ·· |  | 163 | Past year | 2020 | 1 |
| Russian Federation | Yes | 2003 | 6 | 872 | ·· | ·· |  | ·· | ·· | ·· |  |
| Slovakia | Yes | 2020 | 1 | 18 | ·· | ·· |  | 12 | Past year | 2020 | 1 |
| Ukraine | Yes | 2020 | 1 | 110 | ·· | ·· |  | 919 | Past year | 2020 | 1 |
| **Western Europe** |  |  |  |  |  |  |  |  | ·· |  |  |
| Albania | Yes | 2020 | 1 | 23 | ·· | ·· |  | 0 | ·· | 2020 | 1 |
| Andorra | Unknown | ·· |  | 1 | ·· | ·· |  | ·· | ·· | ·· |  |
| Austria | Yes | 2023 | 7 | 27 | 1 | 2023 | 7 | 6 | Snapshot | 2020 | 1 |
| Belgium | Yes | 2020 | 1 | 35 | ·· | ·· |  | 13 | Snapshot | 2020 | 1 |
| Croatia | All facilities | 2023 | 8 | 24 | 24 | 2023 | 8 | ·· | ·· | ·· |  |
| Denmark | Yes | 2013 | 9 | 54 | ·· | ·· |  | ·· | ·· | ·· |  |
| England** | Yes | 2025 | 10 | 120 | ·· | ·· |  | ·· | ·· | ·· |  |
| Finland | Yes | 2020 | 4 | 26 | ·· | ·· |  | ·· | ·· | ·· |  |
| France | Yes | 2020 | 4 | 187 | ·· | ·· |  | ·· | ·· | ·· |  |
| Germany | Yes | 2020 | 1 | 179 | ·· | ·· |  | 21 | Past year | 4019 | 11 |
| Greece | Yes | 2020 | 1 | 34 | ·· | ·· |  | 6 | Past year | 2020 | 1 |
| Greenland | Unknown | ·· |  | 6 | ·· | ·· |  | ·· | ·· | ·· |  |
| Iceland | Unknown | ·· |  | 5 | ·· | ·· |  | ·· | ·· | ·· |  |
| Ireland | Yes | 2020 | 1 | 12 | ·· | ·· |  | 6 | Past year | 2020 | 1 |
| Italy | Yes | 2020 | 4 | 206 | ·· | ·· |  | ·· | ·· | ·· |  |
| Liechtenstein | Unknown | ·· |  | 1 | ·· | ·· |  | ·· | ·· | ·· |  |
| Luxembourg | All facilities | 2023 | 12 | 3 | 3 | 2023 | 12 | 3 | Past year | 2020 | 1 |
| Malta | Yes | 2020 | 1 | 1 | ·· | ·· |  | 16 | Snapshot | 2020 | 1 |
| Monaco | Unknown | ·· |  | 2 | ·· | ·· |  | ·· | ·· | ·· |  |
| Montenegro | Unknown | ·· |  | 3 | ·· | ·· |  | ·· | ·· | ·· |  |
| Netherlands | Yes | 2020 | 4 | 49 | ·· | ·· |  | ·· | ·· | ·· |  |
| North Macedonia | Unknown | ·· |  | 13 | ·· | ·· |  | ·· | ·· | ·· |  |
| Northern Ireland | Yes | 2025 | 10 | 3 | ·· | ·· |  | ·· | ·· | ·· |  |
| Norway | Yes | 2013 | 9 | 33 | ·· | ·· |  | ·· | ·· | ·· |  |
| Portugal | All facilities | 2023 | 13 | 49 | 49 | 2023 | 13 | 76 | Past year | 2020 | 1 |
| San Marino | Unknown | ·· |  | 1 | ·· | ·· |  | ·· | ·· | ·· |  |
| Scotland | Yes | 2025 | 10 | 15 | ·· | ·· |  | ·· | ·· | ·· |  |
| Serbia | Unknown | ·· |  | 30 | ·· | ·· |  | ·· | ·· | ·· |  |
| Slovenia | Yes | 2020 | 4 | 7 | ·· | ·· |  | ·· | ·· | ·· |  |
| Spain | Yes | 2020 | 1 | 82 | ·· | ·· |  | 24 | Past year | 2020 | 1 |
| Sweden | Yes | 2013 | 9 | 79 | ·· | ·· |  | ·· | ·· | ·· |  |
| Switzerland | Yes | 2013 | 9 | 88 | ·· | ·· |  | ·· | ·· | ·· |  |
| Wales** | Yes | 2025 | 10 | 120 | ·· | ·· |  | ·· | ·· | ·· |  |
| **East and South East Asia** |  |  |  |  |  |  |  |  | ·· |  |  |
| Brunei Darussalam | Unknown | ·· |  | 3 | ·· | ·· |  | ·· | ·· | ·· |  |
| Cambodia | Unknown | ·· |  | 29 | ·· | ·· |  | ·· | ·· | ·· |  |
| China | Yes | 2020 | 14 | 683 | ·· | ·· |  | ·· | ·· | ·· |  |
| Hong Kong | Unknown | ·· |  | 24 | ·· | ·· |  | ·· | ·· | ·· |  |
| Indonesia | Unknown | ·· |  | 526 | ·· | ·· |  | ·· | ·· | ·· |  |
| Japan | Yes | 2018 | 15 | 184 | ·· | ·· |  | 89 | Past year | 2016 | 15 |
| Lao People's Democratic Republic | Unknown | ·· |  | 19 | ·· | ·· |  | ·· | ·· | ·· |  |
| Malaysia | Yes | 2016 | 16 | 43 | ·· | ·· |  | ·· | ·· | ·· |  |
| Mongolia | All facilities | 2020 | 17 | 50 | 50 | 2020 | 17 | ·· | ·· | ·· |  |
| Myanmar | Unknown | ·· |  | 96 | ·· | ·· |  | ·· | ·· | ·· |  |
| Democratic People's Republic of Korea | Unknown | ·· |  | N/A | ·· | ·· |  | ·· | ·· | ·· |  |
| Philippines | Yes | 2015 | 18 | 440 | ·· | ·· |  | ·· | ·· | ·· |  |
| Singapore | Yes | 2001 | 19 | 13 | ·· | ·· |  | ·· | ·· | ·· |  |
| Republic of Korea | Unknown | ·· |  | 54 | ·· | ·· |  | ·· | ·· | ·· |  |
| Taiwan | Unknown | ·· |  | 49 | ·· | ·· |  | ·· | ·· | ·· |  |
| Thailand | Yes | 2023 | 20 | 143 | ·· | ·· |  | ·· | ·· | ·· |  |
| Timor-Leste | Unknown | ·· |  | 3 | ·· | ·· |  | ·· | ·· | ·· |  |
| Viet Nam | Unknown | ·· |  | 54 | ·· | ·· |  | ·· | ·· | ·· |  |
| **South Asia** |  |  |  |  |  |  |  |  | ·· |  |  |
| Afghanistan | Unknown | ·· |  | 251 | ·· | ·· |  | ·· | ·· | ·· |  |
| Bangladesh | Yes | 2015 | 21 | 68 | ·· | ·· |  | ·· | ·· | ·· |  |
| Bhutan | Unknown | ·· |  | 7 | ·· | ·· |  | ·· | ·· | ·· |  |
| India | Yes | 2013 | 22 | 1330 | ·· | ·· |  | 424 | Snapshot | 2013 | 22 |
| Islamic Republic of Iran | Unknown | ·· |  | 253 | ·· | ·· |  | ·· | ·· | ·· |  |
| Maldives | Unknown | ·· |  | 11 | ·· | ·· |  | ·· | ·· | ·· |  |
| Nepal | Unknown | ·· |  | 74 | ·· | ·· |  | ·· | ·· | ·· |  |
| Pakistan | Unknown | ·· |  | 116 | ·· | ·· |  | ·· | ·· | ·· |  |
| Sri Lanka | Unknown | ·· |  | 60 | ·· | ·· |  | ·· | ·· | ·· |  |
| **Central Asia** |  |  |  |  |  |  |  |  |  |  |  |
| Kazakhstan | Yes | 2015 | 23 | 80 | ·· | ·· |  | 709 | Snapshot | 2015 | 23 |
| Kyrgyzstan | Yes | 2016 | 24 | 28 | ·· | ·· |  | ·· | ·· | ·· |  |
| Tajikistan | Yes | 2010 | 25 | 19 | ·· | ·· |  | ·· | ·· | ·· |  |
| Turkmenistan | Unknown | ·· |  | 27 | ·· | ·· |  | ·· | ·· | ·· |  |
| Uzbekistan | Yes | 2018 | 26 | 54 | ·· | ·· |  | 760 | Other | 2018 | 26 |
| **Caribbean** |  |  |  |  |  |  |  |  |  |  |  |
| Antigua & Barbuda | Unknown | ·· |  | 1 | ·· | ·· |  | ·· | ·· | ·· |  |
| Bahamas | Unknown | ·· |  | 1 | ·· | ·· |  | ·· | ·· | ·· |  |
| Barbados | Unknown | ·· |  | 1 | ·· | ·· |  | ·· | ·· | ·· |  |
| Bermuda | Unknown | ·· |  | 4 | ·· | ·· |  | ·· | ·· | ·· |  |
| Cuba | Unknown | ·· |  | 200 | ·· | ·· |  | ·· | ·· | ·· |  |
| Dominica | Unknown | ·· |  | 1 | ·· | ·· |  | ·· | ·· | ·· |  |
| Dominican Republic | Unknown | ·· |  | 41 | ·· | ·· |  | ·· | ·· | ·· |  |
| Grenada | Unknown | ·· |  | 1 | ·· | ·· |  | ·· | ·· | ·· |  |
| Haiti | Yes | 2020 | 27 | 17 | ·· | ·· |  | ·· | ·· | ·· |  |
| Jamaica | Unknown | ·· |  | 11 | ·· | ·· |  | ·· | ·· | ·· |  |
| Commonwealth of Puerto Rico | Unknown | ·· |  | 34 | ·· | ·· |  | ·· | ·· | ·· |  |
| Saint Kitts & Nevis | Unknown | ·· |  | 2 | ·· | ·· |  | ·· | ·· | ·· |  |
| Saint Lucia | Unknown | ·· |  | 1 | ·· | ·· |  | ·· | ·· | ·· |  |
| Saint Vincent & the Grenadines | Unknown | ·· |  | 2 | ·· | ·· |  | ·· | ·· | ·· |  |
| Trinidad & Tobago | Unknown | ·· |  | 9 | ·· | ·· |  | ·· | ·· | ·· |  |
| **Latin America** |  |  |  |  |  |  |  |  |  |  |  |
| Argentina | Yes | 2018 | 28 | 338 | ·· | ·· |  | ·· | ·· | ·· |  |
| Belize | Unknown | ·· |  | 1 | ·· | ·· |  | ·· | ·· | ·· |  |
| Plurinational State of Bolivia | Unknown | ·· |  | 40 | ·· | ·· |  | ·· | ·· | ·· |  |
| Brazil | Yes | 2024 | 29 | 1386 | ·· | ·· |  | ·· | ·· | ·· |  |
| Chile | Unknown | ·· |  | 113 | ·· | ·· |  | ·· | ·· | ·· |  |
| Colombia | Yes | 2015 | 30 | 125 | ·· | ·· |  | ·· | ·· | ·· |  |
| Costa Rica | Unknown | ·· |  | 32 | ·· | ·· |  | ·· | ·· | ·· |  |
| Ecuador | Yes | 2021 | 31 | 52 | ·· | ·· |  | ·· | ·· | ·· |  |
| El Salvador | Yes | 2016 | 32 | 25 | ·· | ·· |  | 1679 | Other | 2014 | 32 |
| Guatemala | Unknown | ·· |  | 21 | ·· | ·· |  | ·· | ·· | ·· |  |
| Guyana | Unknown | ·· |  | 7 | ·· | ·· |  | ·· | ·· | ·· |  |
| Honduras | Unknown | ·· |  | 26 | ·· | ·· |  | ·· | ·· | ·· |  |
| Mexico | Yes | 2014 | 33 | 279 | ·· | ·· |  | ·· | ·· | ·· |  |
| Nicaragua | Unknown | ·· |  | 9 | ·· | ·· |  | ·· | ·· | ·· |  |
| Panama | Unknown | ·· |  | 23 | ·· | ·· |  | ·· | ·· | ·· |  |
| Paraguay | Yes | 2018 | 34 | 18 | ·· | ·· |  | ·· | ·· | ·· |  |
| Peru | Yes | 2016 | 35 | 68 | ·· | ·· |  | ·· | ·· | ·· |  |
| Suriname | Unknown | ·· |  | 21 | ·· | ·· |  | ·· | ·· | ·· |  |
| Uruguay | Unknown | ·· |  | 27 | ·· | ·· |  | ·· | ·· | ·· |  |
| Bolivarian Republic of Venezuela | Unknown | ·· |  | 41 | ·· | ·· |  | ·· | ·· | ·· |  |
| **North America** |  |  |  |  |  |  |  |  |  |  |  |
| Canada (Federal) | Yes | 2008 | 36 | 43 | ·· | ·· |  | ·· | ·· | ·· |  |
| Canada (Provincial/Territorial) | Unknown | ·· |  | 173 | ·· | ·· |  | ·· | ·· | ·· |  |
| United States of America (Federal) | Unknown | ·· |  | 102 | ·· | ·· |  | ·· | ·· | ·· |  |
| United States of America (State) | Yes | 2009 | 37 | 1190 | ·· | ·· |  | ·· | ·· | ·· |  |
| **Pacific Island States & Terr.** |  |  |  |  |  |  |  |  |  |  |  |
| American Samoa | Unknown | ·· |  | 1 | ·· | ·· |  | ·· | ·· | ·· |  |
| Federated States of Micronesia | Unknown | ·· |  | 4 | ·· | ·· |  | ·· | ·· | ·· |  |
| Fiji | Unknown | ·· |  | 15 | ·· | ·· |  | ·· | ·· | ·· |  |
| French Polynesia | Unknown | ·· |  | 2 | ·· | ·· |  | ·· | ·· | ·· |  |
| Guam | Unknown | ·· |  | 2 | ·· | ·· |  | ·· | ·· | ·· |  |
| Kiribati | Unknown | ·· |  | 4 | ·· | ·· |  | ·· | ·· | ·· |  |
| Marshall Islands | Unknown | ·· |  | 2 | ·· | ·· |  | ·· | ·· | ·· |  |
| Nauru | Unknown | ·· |  | 1 | ·· | ·· |  | ·· | ·· | ·· |  |
| New Caledonia | Unknown | ·· |  | 1 | ·· | ·· |  | ·· | ·· | ·· |  |
| Northern Mariana Islands | Unknown | ·· |  | 1 | ·· | ·· |  | ·· | ·· | ·· |  |
| Palau | Unknown | ·· |  | 1 | ·· | ·· |  | ·· | ·· | ·· |  |
| Papua New Guinea | Unknown | ·· |  | 18 | ·· | ·· |  | ·· | ·· | ·· |  |
| Samoa | Unknown | ·· |  | 3 | ·· | ·· |  | ·· | ·· | ·· |  |
| Solomon Islands | Unknown | ·· |  | 6 | ·· | ·· |  | ·· | ·· | ·· |  |
| Tonga | Unknown | ·· |  | 4 | ·· | ·· |  | ·· | ·· | ·· |  |
| Tuvalu | Unknown | ·· |  | 2 | ·· | ·· |  | ·· | ·· | ·· |  |
| Vanuatu | Unknown | ·· |  | 4 | ·· | ·· |  | ·· | ·· | ·· |  |
| **Australasia** |  |  |  |  |  |  |  |  | ·· |  |  |
| Australia | Yes | 2020 | 38 | 111 | ·· | ·· |  | ·· | ·· | ·· |  |
| New Zealand | Unknown | ·· |  | 18 | ·· | ·· |  | ·· | ·· | ·· |  |
| **Sub Saharan Africa** |  |  |  |  |  |  |  |  | ·· |  |  |
| Angola | Unknown | ·· |  | 47 | ·· | ·· |  | ·· | ·· | ·· |  |
| Benin | Unknown | ·· |  | 11 | ·· | ·· |  | ·· | ·· | ·· |  |
| Botswana | Unknown | ·· |  | 23 | ·· | ·· |  | ·· | ·· | ·· |  |
| Burkina Faso | Unknown | ·· |  | 27 | ·· | ·· |  | ·· | ·· | ·· |  |
| Burundi | Unknown | ·· |  | 13 | ·· | ·· |  | ·· | ·· | ·· |  |
| Cameroon | Unknown | ·· |  | 76 | ·· | ·· |  | ·· | ·· | ·· |  |
| Cabo Verde | Unknown | ·· |  | 5 | ·· | ·· |  | ·· | ·· | ·· |  |
| Central African Republic | Unknown | ·· |  | 15 | ·· | ·· |  | ·· | ·· | ·· |  |
| Chad | Yes | 2024 | 39 | 41 | ·· | ·· |  | ·· | ·· | ·· |  |
| Comoros | Unknown | ·· |  | 3 | ·· | ·· |  | ·· | ·· | ·· |  |
| Côte d'Ivoire | Yes | 2017 | 40 | 34 | ·· | ·· |  | ·· | ·· | ·· |  |
| Democratic Republic of the Congo | Unknown | ·· |  | 142 | ·· | ·· |  | ·· | ·· | ·· |  |
| Djibouti | Unknown | ·· |  | 1 | ·· | ·· |  | ·· | ·· | ·· |  |
| Equatorial Guinea | Unknown | ·· |  | 15 | ·· | ·· |  | ·· | ·· | ·· |  |
| Eritrea | Unknown | ·· |  | N/A | ·· | ·· |  | ·· | ·· | ·· |  |
| Eswatini | Unknown | ·· |  | 12 | ·· | ·· |  | ·· | ·· | ·· |  |
| Ethiopia | Unknown | ·· |  | 126 | ·· | ·· |  | ·· | ·· | ·· |  |
| Gabon | Unknown | ·· |  | 9 | ·· | ·· |  | ·· | ·· | ·· |  |
| Gambia | Unknown | ·· |  | 3 | ·· | ·· |  | ·· | ·· | ·· |  |
| Ghana | Unknown | ·· |  | 46 | ·· | ·· |  | ·· | ·· | ·· |  |
| Guinea | Yes | 2010 | 41 | 31 | ·· | ·· |  | ·· | ·· | ·· |  |
| Guinea-Bissau | Unknown | ·· |  | 3 | ·· | ·· |  | ·· | ·· | ·· |  |
| Kenya | Unknown | ·· |  | 134 | ·· | ·· |  | ·· | ·· | ·· |  |
| Lesotho | Unknown | ·· |  | 14 | ·· | ·· |  | ·· | ·· | ·· |  |
| Liberia | Unknown | ·· |  | 16 | ·· | ·· |  | ·· | ·· | ·· |  |
| Madagascar | Yes | 2000 | 42 | 82 | ·· | ·· |  | ·· | ·· | ·· |  |
| Malawi | Yes | 2020 | 43 | 30 | ·· | ·· |  | ·· | ·· | ·· |  |
| Mali | Unknown | ·· |  | 60 | ·· | ·· |  | ·· | ·· | ·· |  |
| Mauritania | Unknown | ·· |  | 23 | ·· | ·· |  | ·· | ·· | ·· |  |
| Mauritius | Unknown | ·· |  | 11 | ·· | ·· |  | ·· | ·· | ·· |  |
| Mozambique | Unknown | ·· |  | 157 | ·· | ·· |  | ·· | ·· | ·· |  |
| Namibia | Unknown | ·· |  | 13 | ·· | ·· |  | ·· | ·· | ·· |  |
| Niger | Yes | 2021 | 44 | 41 | ·· | ·· |  | ·· | ·· | ·· |  |
| Nigeria | Yes | 2015 | 45 | 240 | ·· | ·· |  | ·· | ·· | ·· |  |
| Congo | Unknown | ·· |  | 12 | ·· | ·· |  | ·· | ·· | ·· |  |
| Rwanda | Yes | 2017 | 46 | 13 | 8 | 2017 | 46 | ·· | ·· | ·· |  |
| Sao Tome & Principe | Unknown | ·· |  | 1 | ·· | ·· |  | ·· | ·· | ·· |  |
| Senegal | Yes | 2023 | 47 | 37 | ·· | ·· |  | ·· | ·· | ·· |  |
| Seychelles | Unknown | ·· |  | 3 | ·· | ·· |  | ·· | ·· | ·· |  |
| Sierra Leone | Yes | 2023 | 48 | 21 | ·· | ·· |  | ·· | ·· | ·· |  |
| Somalia | Unknown | ·· |  | 14 | ·· | ·· |  | ·· | ·· | ·· |  |
| South Africa | Yes | 2017 | 49 | 235 | ·· | ·· |  | ·· | ·· | ·· |  |
| United Republic of Tanzania | Unknown | ·· |  | 126 | ·· | ·· |  | ·· | ·· | ·· |  |
| Togo | Unknown | ·· |  | 13 | ·· | ·· |  | ·· | ·· | ·· |  |
| Uganda | All facilities | 2014 | 50 | 254 | 254 | 2014 | 50 | ·· | ·· | ·· |  |
| Zambia | Yes | 2010 | 51 | 90 | 6 | 2010 | 51 | ·· | ·· | ·· |  |
| Zimbabwe | All facilities | 2020 | 52 | 72 | 72 | 2020 | 52 | ·· | ·· | ·· |  |
| **Middle East & North Africa** |  |  |  |  |  |  |  |  | ·· |  |  |
| Algeria | Unknown | ·· |  | 162 | ·· | ·· |  | ·· | ·· | ·· |  |
| Bahrain | Unknown | ·· |  | 4 | ·· | ·· |  | ·· | ·· | ·· |  |
| Cyprus | Yes | 2020 | 4 | 1 | ·· | ·· |  | 0 | ·· | 2020 | 1 |
| Egypt | Unknown | ·· |  | 78 | ·· | ·· |  | ·· | ·· | ·· |  |
| Iraq | Unknown | ·· |  | 55 | ·· | ·· |  | ·· | ·· | ·· |  |
| Israel | Unknown | ·· |  | 30 | ·· | ·· |  | ·· | ·· | ·· |  |
| Jordan | Unknown | ·· |  | 17 | ·· | ·· |  | ·· | ·· | ·· |  |
| Kuwait | Unknown | ·· |  | 3 | ·· | ·· |  | ·· | ·· | ·· |  |
| Lebanon | Unknown | ·· |  | 25 | ·· | ·· |  | ·· | ·· | ·· |  |
| Libya | Unknown | ·· |  | 26 | ·· | ·· |  | ·· | ·· | ·· |  |
| Morocco | Unknown | ·· |  | 75 | ·· | ·· |  | ·· | ·· | ·· |  |
| Oman | Unknown | ·· |  | 3 | ·· | ·· |  | ·· | ·· | ·· |  |
| Occupied Palestinian territories | Unknown | ·· |  | N/A | ·· | ·· |  | ·· | ·· | ·· |  |
| Qatar | Unknown | ·· |  | 1 | ·· | ·· |  | ·· | ·· | ·· |  |
| Saudi Arabia | Unknown | ·· |  | 110 | ·· | ·· |  | ·· | ·· | ·· |  |
| South Sudan | Unknown | ·· |  | 80 | ·· | ·· |  | ·· | ·· | ·· |  |
| Sudan | Unknown | ·· |  | 125 | ·· | ·· |  | ·· | ·· | ·· |  |
| Syrian Arab Republic | Unknown | ·· |  | 35 | ·· | ·· |  | ·· | ·· | ·· |  |
| Tunisia | Unknown | ·· |  | 32 | ·· | ·· |  | ·· | ·· | ·· |  |
| Türkiye | Yes | 2020 | 4 | 403 | ·· | ·· |  | ·· | ·· | ·· |  |
| United Arab Emirates | Unknown | ·· |  | 21 | ·· | ·· |  | ·· | ·· | ·· |  |
| Yemen | Unknown | ·· |  | 43 | ·· | ·· |  | ·· | ·· | ·· |  |

**Notes:**

**Ref:** Reference.
***Total sites:** Including prisons, jails or other carceral setting, Country level data that informed these regional and global estimates were sourced from the World Prison Brief, collated by the Institute for Crime and Justice Policy Research at Burbeck University. See: <https://www.prisonstudies.org/world-prison-brief-data>**;**

** The total number of sites for England and Wales was reported together in the World Prison Brief, therefore the total number of sites for both England and Wales is reported as 120.

·· Indicates there were no data to inform a country’s estimate.

† For reporting purposes, these countries or territories are reported separately due to differences in service provision.

**References for Table 6.11**

1. World Health Organization. Status report on prison health in the WHO European Region 2022. Copenhagen, 2023.

2. Burrows D, Boltaev A, Musa M, Gottfreðsson M, Suleymanova J. Review of the HIV programme in Azerbaijan: November 2014: World Health Organization. Regional Office for Europe, 2016.

3. Kanev KA, D. The Reform of The Bulgarian Penitentiary System: Current State and Future Prospects. Sofia, 2024.

4. European Monitoring Centre for Drugs and Drug Addiction. Prison and drugs in Europe: EMCDDA, 2022.

5. Harm Reduction International. Availability, accessibility, acceptability and quality of harm reduction services in Moldovan prisons. London, 2021.

6. IuA P, Punga V. Organization of tuberculosis-controlling work and its efficiency in the penitentiaries of the Ivanovo Region. *Probl Tuberk Bolezn Legk* 2003; (9): 3-5.

7. Reitox Focal Point Austria, Gesundheit Österreich GmbH Vienna. Prison workbook 2023 - Austria. Austria; 2023.

8. Andreić J-L. Prison Workbook 2023 - Croatia, 2023.

9. Zurhold H, Stöver H. Provision of harm reduction and drug treatment services in custodial settings – Findings from the European ACCESS study. *Drugs Educ Prev Policy* 2016.

10. Edge C. Tuberculosis Treatment in Carceral Settings in the UK. In: Santo Jr T, editor.; 2025.

11. Kinner SA, Winter R, Saxton K. A longitudinal study of health outcomes for people released from prison in Fiji: the HIP-Fiji project. *Australas psychiat* 2015; **23**(6_suppl): 17-21.

12. Kugener T, Berndt N, Origer A, Debacker M. Prison workbook 2023 Luxembourg. 2023.

13. de Castro MV, Mendes SCC, Marques JPA, Santos AS, Duarte O. Prison Workbook 2023 - Portugal. 2023.

14. Zhang GQ, Zhang YH, Zhong D, et al. High Prevalence of and Risk Factors for Latent Tuberculosis Infection among Prisoners, Tianjin, China. *Emerg Infect Dis* 2020.

15. Kawatsu L, Uchimura K, Ohkado A. A situational analysis of latent tuberculosis infection among incarcerated population in Japan. *PLoS One* 2018; **13**(9): NA.

16. Al-Darraji HAA, Altice FL, Kamarulzaman A. Undiagnosed pulmonary tuberculosis among prisoners in Malaysia: An overlooked risk for tuberculosis in the community. *Trop Med Int Health* 2016.

17. The Global Fund. Technical Brief: Addresing HIV and TB in Prisons, Pre-Trial Detention and Other Closed Settings, 2020.

18. Matibag RS. Surveillance and control of tuberculosis among the inmates of the Bataan provincial jail: Basis for a population-specific implementation program. *BMJ Open* 2015; **5**(Supplement 1): A40.

19. Chee CB, Teleman MD, Boudville IC, Wang YT. Contact screening and latent TB infection treatment in Singapore correctional facilities. *Int J Tuberc Lung Dis* 2005.

20. Department of Disease Control Ministry of Public Health - Division of Tuberculosis. Thailand Operational Plan To End Tuberculosis, Phase 2 (2023 - 2027). 2023.

21. Banu S, Rahman MT, Uddin MKM, et al. Effect of active case finding on prevalence and transmission of pulmonary tuberculosis in Dhaka Central Jail, Bangladesh. *PLoS One* 2015.

22. Prasad BM, Thapa B, Chadha SS, et al. Status of Tuberculosis services in Indian Prisons. *Int J Infect Dis* 2017.

23. WHO. WHO assessment of tuberculosis prevention, control and care in Kazakhstan, 2015.

24. Subata E, Moller L, Karymbaeva S. Evaluation of opioid substitution therapy in Kyrgyzstan. *Copenhagen, Denmark: World Health Organization[Google Scholar]* 2016.

25. Winetsky DE, Almukhamedov O, Pulatov D, Vezhnina N, Dooronbekova A, Zhussupov B. Prevalence, risk factors and social context of active pulmonary tuberculosis among prison inmates in Tajikistan. *PLoS One* 2014.

26. Safaev K, Parpieva N, Liverko I, et al. Trends, characteristics and treatment outcomes of patients with drug-resistant tuberculosis in Uzbekistan: 2013–2018. *Int J Environ Res Public Health* 2021; **18**(9): 4663.

27. Ekwebelem LC, Bury MR, Prophete EJ, Duverger K, Bircher PM, Spaulding AC. Measuring Depression, and Its Association with Substance Use, during an Adherence Study of Patients Treated for Tuberculosis in Haitian Prisons. *medRxiv* 2023.

28. Biasutti F, Rossi E, Moro A, et al. Study of tuberculosis cases registered among prisoners in South Santa Fe, Argentina. *J Int AIDS Soc Conference: HIV and Hepatitis in the Americas* 2019.

29. DATASUS. Ministry of Health/SVSA - Notifiable Diseases Information System - Sinan Net. 2024. http://tabnet.datasus.gov.br/cgi/tabcgi.exe?sinannet/cnv/tubercbr.def.

30. Guerra J, Mogollon D, Gonzalez D, et al. Active and latent tuberculosis among inmates in La Esperanza prison in Guaduas, Colombia. *PLoS One* 2019.

31. Valcarcel-Perez I, Molina JL, Fuentes Z. Is mass screening enough to control tuberculosis in Ecuador's prisons? *Rev Esp Sanid Penit* 2021.

32. Ayala G, Garay J, Aragon M, Decroo T, Zachariah R. Trends in tuberculosis notification and treatment outcomes in prisons: a country-wide assessment in El Salvador from 2009–2014. *Rev Panam Salud Publica;39(1),ene 2016* 2016.

33. Joya M, Avilés, MAG, Saldaña, DA & Olivares LM. National TB Strategy in the prisons - 2014… the year of detection, treatment and cure of tuberculosis patients. 2014.

34. Sequera VG, Aguirre S, Estigarribia G, et al. Increased incarceration rates drive growing tuberculosis burden in prisons and jeopardize overall tuberculosis control in Paraguay. *Sci Rep* 2020.

35. Ministerio de Salud DGdE. Análisis de la situación epidemiológica de la tuberculosis en el Perú, 2015. Lima, Peru, 2016.

36. Correctional Service of Canada. Infectious disease surveillance in Canadian federal penitentiaries 2007–2008: pre-release report. Correctional Service of Canada Ottawa, ON; 2012.

37. Weant TE, Turner AN, Murphy-Weiss M, Murray DM, Wang SH. Can social history variables predict prison inmates' risk for latent tuberculosis infection? *Tuberc Res Treat* 2012; **2012**: 132406.

38. Simpson PL, Gardoll B, White L, Butler T. HIV policies in Australian prisons: a structured review assessing compliance with international guidelines. *Lancet Reg Health West Pac* 2023; **41**.

39. Ahmat AI, Hassan MTN, Richard NBN, et al. Tuberculosis in the Carcinal Environment in Chad Due to the Mycobacterium Tuberculosis Circulante Complex. *J Biosci Med* 2024; **12**(02): 214-24.

40. Seri B, Koffi A, Danel C, et al. Prevalence of pulmonary tuberculosis among prison inmates: A cross-sectional survey at the Correctional and Detention Facility of Abidjan, Cote d'Ivoire. *PLoS One* 2017.

41. Bah H, Cisse FA, Camara LM, Diallo OH, Diallo M, Sow OY. Prevalence of tuberculosis in the prison population of Conakry, Guinea Republic. *Rev Med Leg* 2012.

42. Rasolofo-Razanamparany V, Menard D, Ratsitorahina M, Auregan G, Gicquel B, Chanteau S. Transmission of tuberculosis in the prison of Antananarivo (Madagascar). *Res Microbiol* 2000.

43. Singano V, Kip E, Ching'ani W, Chiwaula L. Tuberculosis treatment outcomes among prisoners and general population in Zomba, Malawi. *BMC Public Health* 2020.

44. Sani K, Laouali HAM, Laminou AM, et al. Tuberculose pulmonaire en milieu carcéral au Niger: Aspects épidémiologiques, diagnostiques, thérapeutiques et évolutifs. *Eur Sci J* 2021; **17**(1).

45. Ekundayo EO, Onuka O, Mustapha G, Geoffrey M. Active case finding of pulmonary tuberculosis among prison inmates in aba Federal prison, Abia state, Nigeria. *Adv Infect Dis* 2015; **5**(01): 57.

46. Institute of HIV/AIDS Disease Prevention and Control. Tuberculosis National Strategic Plan 2013-2018 Extension 2018-2020, 2017.

47. Ministre de la Santé et de l’Action sociale. Plan strategique national pour une riposte multisectorielle integree contre le SIDA, la tuberculose, les hepatites virales et les IST, 2023.

48. Pape S, Ngaujah SDT, Gulma K, Shivalli S, de Kiev LC. Tuberculosis screening on admission to Sierra Leone’s correctional facilities: A SWOC analysis. 2023.

49. Baird K, Said H, Koornhof HJ, Duse AG. Tuberculosis control at a South African correctional centre: Diagnosis, treatment and strain characterisation. *PLoS One* 2022; **17**(11-Nov): e0277459.

50. Schwitters A, Kaggwa M, Omiel P, Nagadya G, Kisa N, Dalal S. Tuberculosis incidence and treatment completion among Ugandan prison inmates. *Int J Tuberc Lung Dis* 2014; **18**(7): 781-6.

51. Todrys KW, Amon JJ, Malembeka G, Clayton M. Imprisoned and imperiled: access to HIV and TB prevention and treatment, and denial of human rights, in Zambian prisons. *J Int AIDS Soc* 2011.

52. Mandizvidza A, Dlodlo RA, Chinnakali P, et al. Tuberculosis Case Finding Cascade and Treatment Outcomes among Male Inmates in Two Prisons in Zimbabwe. *Tuberc res treat* 2020; **2020**(1): 5829471.

# Appendix 7: Additional maps of service availability

## *Figure 7a*: Global availability of HIV testing in carceral settings


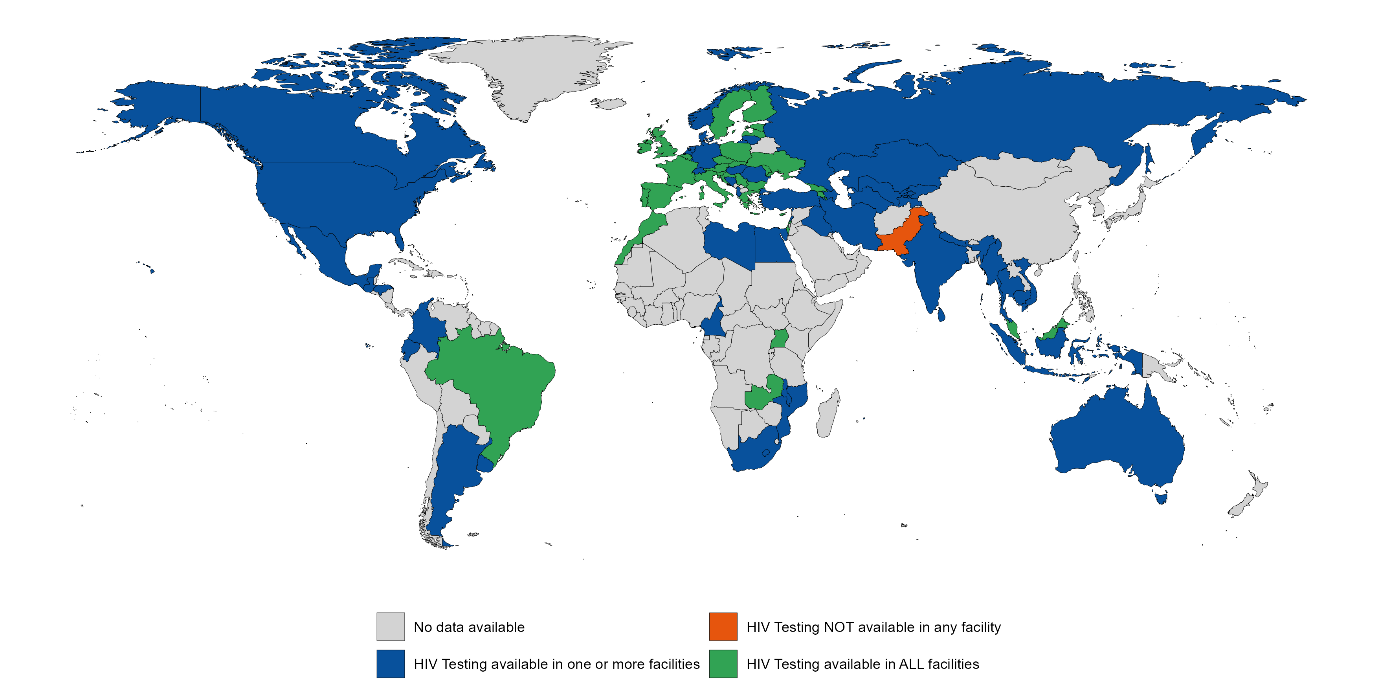


## *Figure 7b*: Global availability of HCV testing in carceral settings


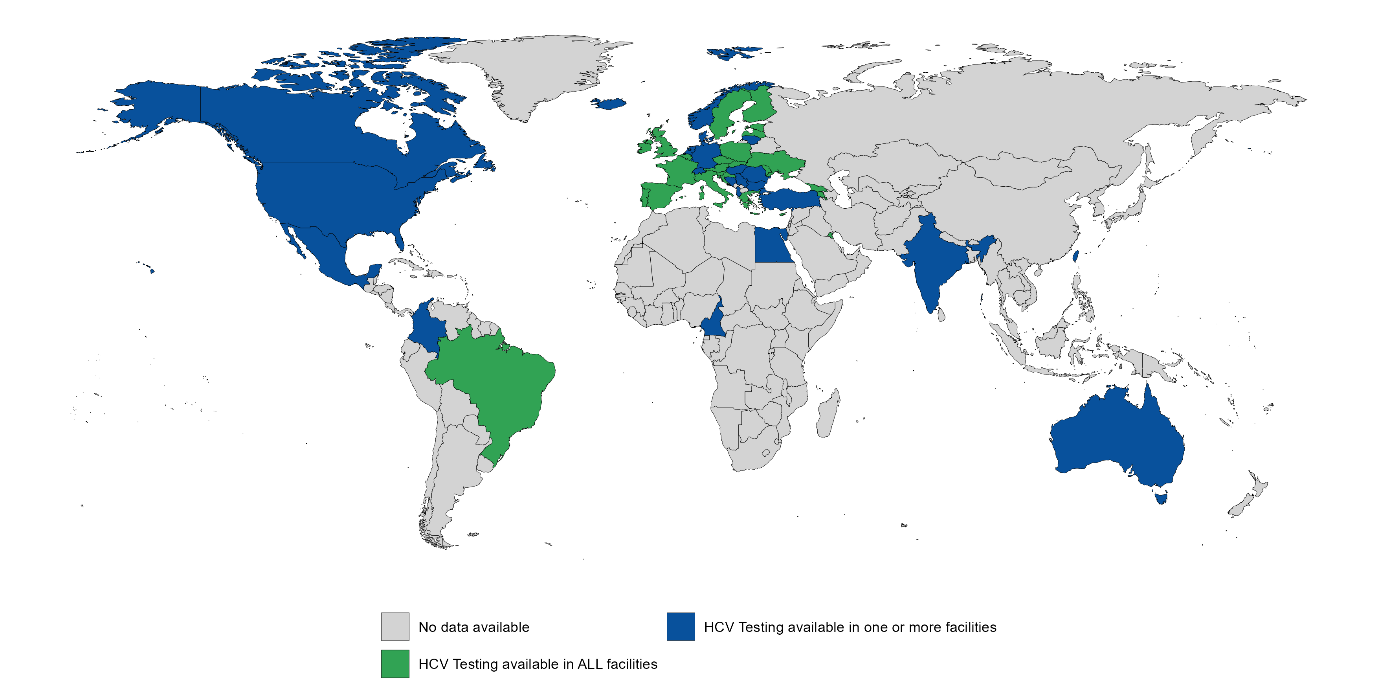


## *Figure 7c*: Global availability of HBV testing in carceral settings


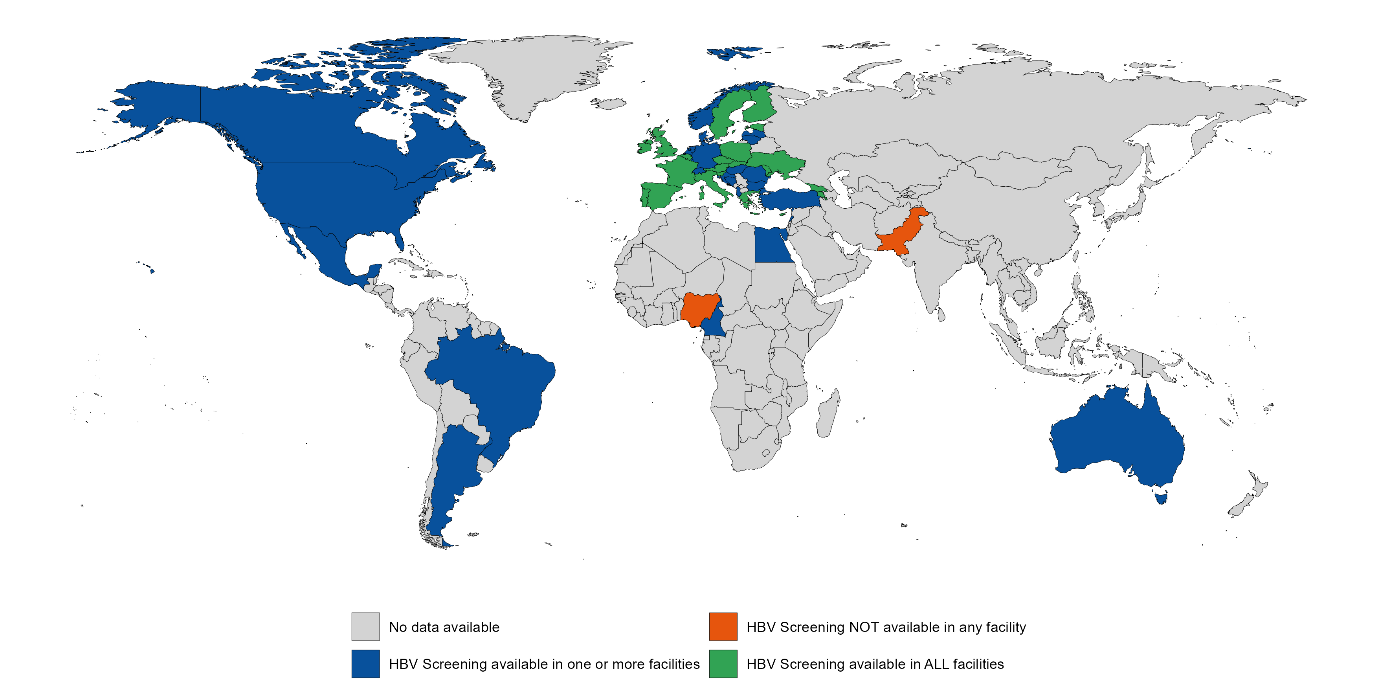


## *Figure 7d*: Global availability of HBV Vaccination availability in carceral settings


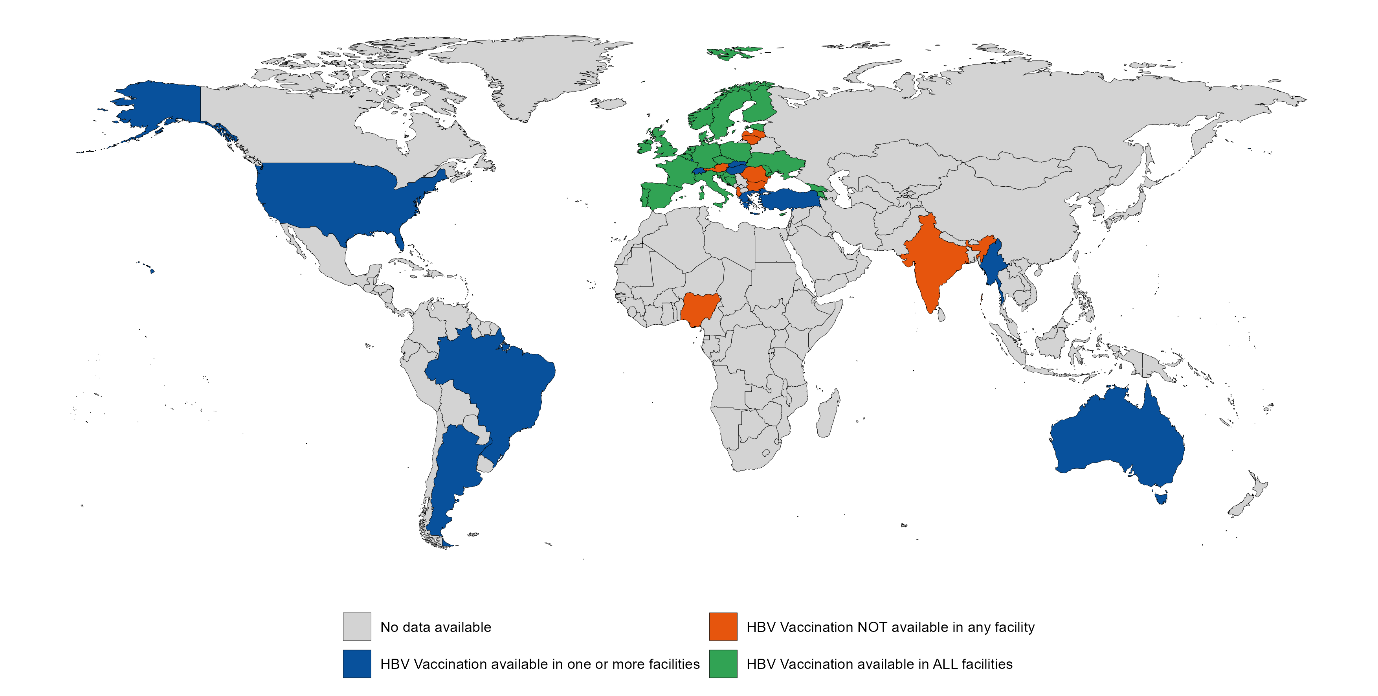


## *Figure 7e*: Global availability of Tuberculosis Screening in carceral settings


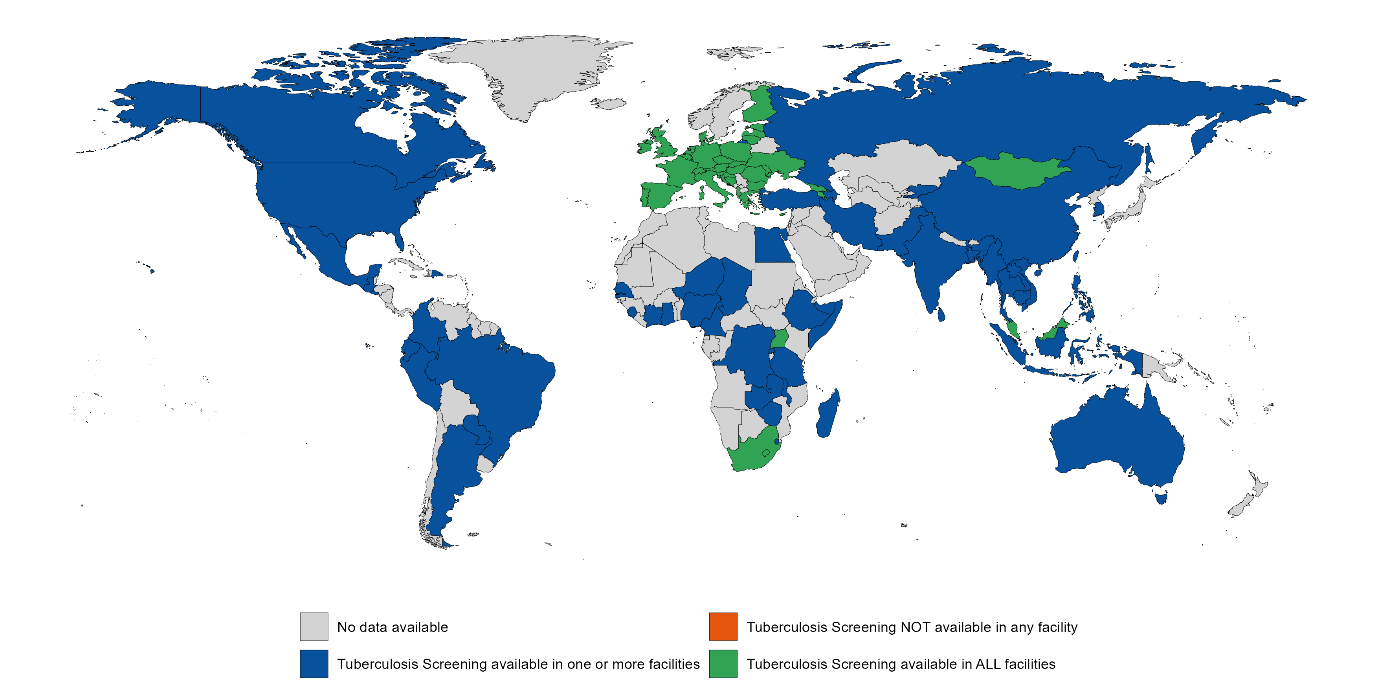


**Notes: Carceral Settings:** Including prisons, jails or other carceral setting; **OAT:** Opioid Agonist Treatment; **NSP**: Needle & Syringe Programme(s); **HIV**: Human Immunodeficiency Virus; **ART**: Antiretroviral Therapy; **HCV**: Hepatitis C; **HBV**: Hepatitis B

# Appendix 8: Country-level availability of all outcomes

| **Country** | **OAT** | | **NSP** | | **HIV Testing** | | **HIV Treatment** | | **HCV Testing** | | **HCV Treatment** | | **Tuberculosis Screening** | | **Tuberculosis**  **Treatment** | | **HBV Testing** | | **HBV Vaccination** | | **HBV Treatment** |
| --- | --- | --- | --- | --- | --- | --- | --- | --- | --- | --- | --- | --- | --- | --- | --- | --- | --- | --- | --- | --- | --- |
| **Eastern Europe** | |  | |  | |  | |  | |  | |  | |  | |  | |  | |  | |
| Armenia | One or more | | No | | All facilities | | One or more | | All facilities | | One or more | | All facilities | | One or more | | All facilities | | All facilities | | Unknown |
| Azerbaijan | No | | No | | One or more | | One or more | | Unknown | | Unknown | | One or more | | One or more | | Unknown | | Unknown | | Unknown |
| Belarus | No | | No | | Unknown | | One or more | | Unknown | | Unknown | | Unknown | | Unknown | | Unknown | | Unknown | | Unknown |
| Bosnia & Herzegovina | One or more | | No | | One or more | | One or more | | One or more | | One or more | | All facilities | | One or more | | One or more | | All facilities | | One or more |
| Bulgaria | One or more | | No | | All facilities | | One or more | | One or more | | No | | All facilities | | One or more | | One or more | | No | | No |
| Czechia | One or more | | No | | All facilities | | One or more | | All facilities | | One or more | | All facilities | | One or more | | All facilities | | All facilities | | One or more |
| Estonia | One or more | | No | | All facilities | | All facilities | | All facilities | | All facilities | | All facilities | | One or more | | All facilities | | All facilities | | One or more |
| Georgia | One or more | | No | | All facilities | | One or more | | All facilities | | One or more | | All facilities | | One or more | | All facilities | | All facilities | | One or more |
| Hungary | No | | No | | One or more | | One or more | | One or more | | One or more | | All facilities | | One or more | | One or more | | One or more | | One or more |
| Latvia | One or more | | No | | All facilities | | All facilities | | All facilities | | One or more | | All facilities | | One or more | | One or more | | No | | One or more |
| Lithuania | All facilities | | No | | One or more | | One or more | | One or more | | One or more | | All facilities | | One or more | | One or more | | No | | One or more |
| Republic of Moldova | One or more | | One or more | | All facilities | | All facilities | | All facilities | | All facilities | | All facilities | | All facilities | | All facilities | | All facilities | | One or more |
| Poland | One or more | | No | | All facilities | | All facilities | | All facilities | | One or more | | All facilities | | One or more | | All facilities | | All facilities | | One or more |
| Romania | One or more | | No | | One or more | | One or more | | One or more | | One or more | | All facilities | | One or more | | One or more | | No | | One or more |
| Russian Federation | No | | No | | One or more | | One or more | | Unknown | | Unknown | | One or more | | One or more | | Unknown | | Unknown | | Unknown |
| Slovakia | No | | No | | All facilities | | All facilities | | All facilities | | All facilities | | All facilities | | One or more | | All facilities | | One or more | | One or more |
| Ukraine | One or more | | One or more | | All facilities | | One or more | | All facilities | | One or more | | All facilities | | One or more | | All facilities | | All facilities | | One or more |
| **Western Europe** | |  | |  | |  | |  | |  | |  | |  | |  | |  | |  | |
| Albania | One or more | | No | | One or more | | One or more | | One or more | | One or more | | All facilities | | One or more | | One or more | | No | | Unknown |
| Andorra | No | | No | | Unknown | | Unknown | | Unknown | | Unknown | | Unknown | | Unknown | | Unknown | | Unknown | | Unknown |
| Austria | All facilities | | No | | All facilities | | All facilities | | All facilities | | All facilities | | All facilities | | One or more | | All facilities | | No | | All facilities |
| Belgium | All facilities | | No | | All facilities | | One or more | | All facilities | | One or more | | All facilities | | One or more | | All facilities | | All facilities | | One or more |
| Croatia | All facilities | | Unknown | | All facilities | | All facilities | | All facilities | | All facilities | | All facilities | | All facilities | | One or more | | All facilities | | All facilities |
| Denmark | All facilities | | No | | One or more | | One or more | | One or more | | No | | All facilities | | One or more | | One or more | | All facilities | | Unknown |
| England | All facilities | | No | | All facilities | | All facilities | | All facilities | | All facilities | | All facilities | | One or more | | All facilities | | All facilities | | All facilities |
| Finland | All facilities | | No | | All facilities | | All facilities | | All facilities | | All facilities | | All facilities | | One or more | | All facilities | | All facilities | | All facilities |
| France | All facilities | | No | | All facilities | | All facilities | | All facilities | | One or more | | All facilities | | One or more | | All facilities | | All facilities | | One or more |
| Germany | All facilities | | One or more | | One or more | | One or more | | One or more | | One or more | | All facilities | | One or more | | One or more | | All facilities | | One or more |
| Greece | One or more | | No | | All facilities | | One or more | | All facilities | | One or more | | All facilities | | One or more | | All facilities | | One or more | | One or more |
| Greenland | No | | No | | Unknown | | Unknown | | Unknown | | Unknown | | Unknown | | Unknown | | Unknown | | Unknown | | Unknown |
| Iceland | One or more | | Unknown | | Unknown | | Unknown | | One or more | | Unknown | | Unknown | | Unknown | | Unknown | | Unknown | | Unknown |
| Ireland | One or more | | No | | All facilities | | One or more | | All facilities | | One or more | | All facilities | | One or more | | All facilities | | All facilities | | One or more |
| Italy | One or more | | No | | All facilities | | One or more | | All facilities | | One or more | | All facilities | | One or more | | All facilities | | All facilities | | One or more |
| Liechtenstein | No | | No | | Unknown | | Unknown | | Unknown | | Unknown | | Unknown | | Unknown | | Unknown | | Unknown | | Unknown |
| Luxembourg | One or more | | All facilities | | One or more | | All facilities | | One or more | | All facilities | | One or more | | All facilities | | One or more | | One or more | | All facilities |
| Malta | One or more | | No | | All facilities | | One or more | | All facilities | | One or more | | All facilities | | One or more | | All facilities | | All facilities | | One or more |
| Monaco | Unknown | | No | | All facilities | | Unknown | | All facilities | | Unknown | | All facilities | | Unknown | | All facilities | | All facilities | | Unknown |
| Montenegro | One or more | | Unknown | | Unknown | | Unknown | | Unknown | | Unknown | | Unknown | | Unknown | | Unknown | | Unknown | | Unknown |
| Netherlands | One or more | | No | | One or more | | One or more | | One or more | | One or more | | All facilities | | One or more | | One or more | | All facilities | | One or more |
| North Macedonia | All facilities | | No | | Unknown | | Unknown | | Unknown | | No | | Unknown | | Unknown | | Unknown | | Unknown | | Unknown |
| Northern Ireland | All facilities | | No | | All facilities | | Unknown | | All facilities | | All facilities | | All facilities | | One or more | | All facilities | | All facilities | | Unknown |
| Norway | All facilities | | No | | One or more | | One or more | | One or more | | One or more | | Unknown | | One or more | | One or more | | All facilities | | One or more |
| Portugal | All facilities | | No | | All facilities | | All facilities | | All facilities | | All facilities | | All facilities | | All facilities | | All facilities | | All facilities | | All facilities |
| San Marino | Unknown | | All facilities | | All facilities | | Unknown | | Unknown | | Unknown | | All facilities | | Unknown | | Unknown | | All facilities | | Unknown |
| Scotland | All facilities | | No | | All facilities | | One or more | | All facilities | | All facilities | | All facilities | | One or more | | All facilities | | All facilities | | Unknown |
| Serbia | All facilities | | No | | All facilities | | All facilities | | One or more | | One or more | | Unknown | | Unknown | | Unknown | | Unknown | | Unknown |
| Slovenia | One or more | | No | | All facilities | | All facilities | | All facilities | | All facilities | | All facilities | | One or more | | All facilities | | All facilities | | All facilities |
| Spain | All facilities | | All facilities | | All facilities | | All facilities | | All facilities | | All facilities | | All facilities | | One or more | | All facilities | | All facilities | | All facilities |
| Sweden | One or more | | No | | All facilities | | All facilities | | All facilities | | All facilities | | Unknown | | One or more | | All facilities | | All facilities | | All facilities |
| Switzerland | One or more | | One or more | | One or more | | One or more | | One or more | | One or more | | All facilities | | One or more | | One or more | | One or more | | One or more |
| Wales | All facilities | | No | | All facilities | | All facilities | | All facilities | | All facilities | | All facilities | | One or more | | All facilities | | All facilities | | Unknown |
| **East and South East Asia** | |  | |  | |  | |  | |  | |  | |  | |  | |  | |  | |
| Brunei Darussalam | No | | No | | Unknown | | Unknown | | Unknown | | Unknown | | Unknown | | Unknown | | Unknown | | Unknown | | Unknown |
| Cambodia | Unknown | | Unknown | | One or more | | Unknown | | Unknown | | Unknown | | One or more | | Unknown | | Unknown | | Unknown | | Unknown |
| China | Unknown | | Unknown | | Unknown | | Unknown | | Unknown | | Unknown | | One or more | | One or more | | Unknown | | Unknown | | Unknown |
| Hong Kong | Unknown | | No | | One or more | | Unknown | | Unknown | | Unknown | | Unknown | | Unknown | | Unknown | | Unknown | | Unknown |
| Indonesia | One or more | | No | | One or more | | One or more | | No | | No | | One or more | | Unknown | | Unknown | | Unknown | | Unknown |
| Japan | No | | No | | Unknown | | Unknown | | Unknown | | Unknown | | Unknown | | One or more | | Unknown | | Unknown | | Unknown |
| Lao People's Democratic Republic | No | | Unknown | | Unknown | | Unknown | | Unknown | | Unknown | | One or more | | Unknown | | Unknown | | Unknown | | Unknown |
| Malaysia | One or more | | No | | All facilities | | One or more | | Unknown | | Unknown | | All facilities | | One or more | | Unknown | | Unknown | | Unknown |
| Mongolia | No | | Unknown | | Unknown | | Unknown | | Unknown | | Unknown | | All facilities | | All facilities | | Unknown | | Unknown | | Unknown |
| Myanmar | No | | Unknown | | One or more | | One or more | | Unknown | | Unknown | | One or more | | Unknown | | Unknown | | One or more | | Unknown |
| Democratic People's Republic of Korea | No | | No | | Unknown | | Unknown | | Unknown | | Unknown | | Unknown | | Unknown | | Unknown | | Unknown | | Unknown |
| Philippines | No | | No | | Unknown | | Unknown | | Unknown | | Unknown | | One or more | | One or more | | Unknown | | Unknown | | Unknown |
| Singapore | No | | No | | Unknown | | Unknown | | Unknown | | Unknown | | One or more | | One or more | | Unknown | | Unknown | | Unknown |
| Republic of Korea | No | | No | | Unknown | | Unknown | | Unknown | | Unknown | | One or more | | Unknown | | Unknown | | Unknown | | Unknown |
| Taiwan | Unknown | | Unknown | | Unknown | | One or more | | One or more | | Unknown | | One or more | | Unknown | | Unknown | | Unknown | | Unknown |
| Thailand | Unknown | | Unknown | | One or more | | One or more | | Unknown | | No | | One or more | | One or more | | Unknown | | Unknown | | Unknown |
| Timor-Leste | No | | No | | Unknown | | Unknown | | Unknown | | Unknown | | One or more | | Unknown | | Unknown | | Unknown | | Unknown |
| Viet Nam | One or more | | Unknown | | One or more | | One or more | | Unknown | | Unknown | | One or more | | Unknown | | Unknown | | Unknown | | Unknown |
| **South Asia** |  | |  | |  | |  | |  | |  | |  | |  | |  | |  | |  |
| Afghanistan | Unknown | | No | | Unknown | | Unknown | | Unknown | | Unknown | | Unknown | | Unknown | | Unknown | | Unknown | | Unknown |
| Bangladesh | Unknown | | Unknown | | Unknown | | Unknown | | Unknown | | Unknown | | One or more | | One or more | | Unknown | | Unknown | | Unknown |
| Bhutan | No | | No | | Unknown | | Unknown | | Unknown | | Unknown | | Unknown | | Unknown | | Unknown | | Unknown | | Unknown |
| India | One or more | | No | | One or more | | One or more | | One or more | | One or more | | One or more | | One or more | | Unknown | | Unknown | | Unknown |
| Islamic Republic of Iran | All facilities | | No | | One or more | | All facilities | | No | | No | | One or more | | Unknown | | Unknown | | Unknown | | Unknown |
| Maldives | Unknown | | No | | One or more | | Unknown | | One or more | | Unknown | | No | | Unknown | | One or more | | Unknown | | Unknown |
| Nepal | Unknown | | Unknown | | One or more | | Unknown | | Unknown | | Unknown | | Unknown | | Unknown | | Unknown | | Unknown | | Unknown |
| Pakistan | No | | Unknown | | No | | Unknown | | Unknown | | Unknown | | One or more | | Unknown | | No | | Unknown | | Unknown |
| Sri Lanka | No | | No | | One or more | | Unknown | | Unknown | | Unknown | | One or more | | Unknown | | Unknown | | Unknown | | Unknown |
| **Central Asia** |  | |  | |  | |  | |  | |  | |  | |  | |  | |  | |  |
| Kazakhstan | No | | No | | One or more | | One or more | | Unknown | | Unknown | | Unknown | | One or more | | Unknown | | Unknown | | Unknown |
| Kyrgyzstan | One or more | | One or more | | One or more | | One or more | | Unknown | | Unknown | | One or more | | One or more | | Unknown | | Unknown | | Unknown |
| Tajikistan | One or more | | One or more | | One or more | | One or more | | Unknown | | Unknown | | Unknown | | One or more | | Unknown | | Unknown | | Unknown |
| Turkmenistan | No | | No | | One or more | | No | | Unknown | | Unknown | | Unknown | | Unknown | | Unknown | | Unknown | | Unknown |
| Uzbekistan | No | | No | | One or more | | One or more | | Unknown | | Unknown | | Unknown | | One or more | | Unknown | | Unknown | | Unknown |
| **Caribbean** |  | |  | |  | |  | |  | |  | |  | |  | |  | |  | |  |
| Antigua & Barbuda | No | | No | | Unknown | | Unknown | | Unknown | | Unknown | | Unknown | | Unknown | | Unknown | | Unknown | | Unknown |
| Bahamas | No | | No | | Unknown | | Unknown | | Unknown | | Unknown | | Unknown | | Unknown | | Unknown | | Unknown | | Unknown |
| Barbados | No | | No | | Unknown | | Unknown | | Unknown | | Unknown | | Unknown | | Unknown | | Unknown | | Unknown | | Unknown |
| Bermuda | No | | No | | Unknown | | Unknown | | Unknown | | Unknown | | Unknown | | Unknown | | Unknown | | Unknown | | Unknown |
| Cuba | No | | No | | Unknown | | Unknown | | Unknown | | Unknown | | Unknown | | Unknown | | Unknown | | Unknown | | Unknown |
| Dominica | No | | No | | Unknown | | Unknown | | Unknown | | Unknown | | Unknown | | Unknown | | Unknown | | Unknown | | Unknown |
| Dominican Republic | Unknown | | Unknown | | Unknown | | Unknown | | Unknown | | Unknown | | One or more | | Unknown | | Unknown | | Unknown | | Unknown |
| Grenada | No | | No | | Unknown | | Unknown | | Unknown | | Unknown | | Unknown | | Unknown | | Unknown | | Unknown | | Unknown |
| Haiti | No | | No | | Unknown | | All facilities | | Unknown | | Unknown | | Unknown | | One or more | | Unknown | | Unknown | | Unknown |
| Jamaica | No | | No | | No | | One or more | | Unknown | | Unknown | | Unknown | | Unknown | | Unknown | | Unknown | | Unknown |
| Commonwealth of Puerto Rico | Unknown | | Unknown | | Unknown | | Unknown | | Unknown | | Unknown | | Unknown | | Unknown | | Unknown | | Unknown | | Unknown |
| Saint Kitts & Nevis | No | | No | | Unknown | | Unknown | | Unknown | | Unknown | | Unknown | | Unknown | | Unknown | | Unknown | | Unknown |
| Saint Lucia | No | | No | | Unknown | | Unknown | | Unknown | | Unknown | | Unknown | | Unknown | | Unknown | | Unknown | | Unknown |
| Saint Vincent & the Grenadines | No | | No | | Unknown | | Unknown | | Unknown | | No | | Unknown | | Unknown | | Unknown | | Unknown | | Unknown |
| Trinidad & Tobago | No | | No | | Unknown | | Unknown | | Unknown | | No | | Unknown | | Unknown | | Unknown | | Unknown | | Unknown |
| **Latin America** | |  | |  | |  | |  | |  | |  | |  | |  | |  | |  | |
| Argentina | Unknown | | No | | One or more | | One or more | | Unknown | | Unknown | | One or more | | One or more | | One or more | | One or more | | One or more |
| Belize | No | | No | | Unknown | | Unknown | | Unknown | | Unknown | | Unknown | | Unknown | | Unknown | | Unknown | | Unknown |
| Plurinational State of Bolivia | No | | No | | Unknown | | No | | Unknown | | Unknown | | Unknown | | Unknown | | Unknown | | Unknown | | Unknown |
| Brazil | No | | No | | All facilities | | All facilities | | All facilities | | Unknown | | One or more | | One or more | | One or more | | One or more | | Unknown |
| Chile | No | | No | | Unknown | | Unknown | | Unknown | | Unknown | | Unknown | | Unknown | | Unknown | | Unknown | | Unknown |
| Colombia | One or more | | No | | One or more | | One or more | | One or more | | One or more | | One or more | | One or more | | Unknown | | Unknown | | Unknown |
| Costa Rica | No | | No | | Unknown | | Unknown | | Unknown | | Unknown | | Unknown | | Unknown | | Unknown | | Unknown | | Unknown |
| Ecuador | No | | No | | One or more | | Unknown | | Unknown | | Unknown | | One or more | | One or more | | Unknown | | Unknown | | Unknown |
| El Salvador | No | | No | | Unknown | | Unknown | | Unknown | | Unknown | | One or more | | One or more | | Unknown | | Unknown | | Unknown |
| Guatemala | No | | No | | One or more | | Unknown | | Unknown | | Unknown | | One or more | | Unknown | | Unknown | | Unknown | | Unknown |
| Guyana | No | | No | | Unknown | | Unknown | | Unknown | | Unknown | | Unknown | | Unknown | | Unknown | | Unknown | | Unknown |
| Honduras | No | | No | | One or more | | Unknown | | Unknown | | Unknown | | Unknown | | Unknown | | Unknown | | Unknown | | Unknown |
| Mexico | No | | No | | One or more | | All facilities | | One or more | | One or more | | One or more | | One or more | | One or more | | Unknown | | Unknown |
| Nicaragua | No | | No | | Unknown | | Unknown | | Unknown | | Unknown | | Unknown | | Unknown | | Unknown | | Unknown | | Unknown |
| Panama | No | | No | | Unknown | | Unknown | | Unknown | | Unknown | | Unknown | | Unknown | | Unknown | | Unknown | | Unknown |
| Paraguay | No | | No | | Unknown | | Unknown | | Unknown | | Unknown | | One or more | | One or more | | Unknown | | Unknown | | Unknown |
| Peru | No | | No | | Unknown | | One or more | | Unknown | | Unknown | | One or more | | One or more | | Unknown | | Unknown | | One or more |
| Suriname | No | | No | | Unknown | | Unknown | | Unknown | | Unknown | | Unknown | | Unknown | | Unknown | | Unknown | | Unknown |
| Uruguay | No | | No | | One or more | | Unknown | | Unknown | | Unknown | | Unknown | | Unknown | | Unknown | | Unknown | | Unknown |
| Bolivarian Republic of Venezuela | No | | No | | Unknown | | Unknown | | Unknown | | Unknown | | Unknown | | Unknown | | Unknown | | Unknown | | Unknown |
| **North America** | |  | |  | |  | |  | |  | |  | |  | |  | |  | |  | |
| Canada (Federal) | All facilities | | One or more | | All facilities | | All facilities | | All facilities | | All facilities | | All facilities | | One or more | | All facilities | | Unknown | | Unknown |
| Canada (Provincial/Territorial) | One or more | | No | | Unknown | | Unknown | | One or more | | One or more | | Unknown | | Unknown | | Unknown | | Unknown | | Unknown |
| United States of America (Federal) | Unknown | | No | | All facilities | | All facilities | | Unknown | | Unknown | | All facilities | | Unknown | | Unknown | | One or more | | Unknown |
| United States of America (State) | One or more | | No | | All facilities | | All facilities | | One or more | | One or more | | One or more | | One or more | | One or more | | One or more | | Unknown |
| **Pacific Islan States & Terr.** | |  | |  | |  | |  | |  | |  | |  | |  | |  | |  | |
| American Samoa | No | | No | | Unknown | | Unknown | | Unknown | | Unknown | | Unknown | | Unknown | | Unknown | | Unknown | | Unknown |
| Federated States of Micronesia | No | | No | | Unknown | | Unknown | | Unknown | | Unknown | | Unknown | | Unknown | | Unknown | | Unknown | | Unknown |
| Fiji | No | | No | | Unknown | | Unknown | | Unknown | | No | | Unknown | | Unknown | | Unknown | | Unknown | | Unknown |
| French Polynesia | No | | No | | Unknown | | Unknown | | Unknown | | Unknown | | Unknown | | Unknown | | Unknown | | Unknown | | Unknown |
| Guam | No | | No | | Unknown | | Unknown | | Unknown | | Unknown | | Unknown | | Unknown | | Unknown | | Unknown | | Unknown |
| Kiribati | No | | No | | Unknown | | Unknown | | Unknown | | Unknown | | Unknown | | Unknown | | Unknown | | Unknown | | Unknown |
| Marshall Islands | No | | No | | Unknown | | Unknown | | Unknown | | Unknown | | Unknown | | Unknown | | Unknown | | Unknown | | Unknown |
| Nauru | No | | No | | Unknown | | Unknown | | Unknown | | Unknown | | Unknown | | Unknown | | Unknown | | Unknown | | Unknown |
| New Caledonia | No | | No | | Unknown | | Unknown | | Unknown | | Unknown | | Unknown | | Unknown | | Unknown | | Unknown | | Unknown |
| Northern Mariana Islands | No | | No | | Unknown | | Unknown | | Unknown | | Unknown | | Unknown | | Unknown | | Unknown | | Unknown | | Unknown |
| Palau | No | | No | | Unknown | | Unknown | | Unknown | | Unknown | | Unknown | | Unknown | | Unknown | | Unknown | | Unknown |
| Papua New Guinea | No | | No | | Unknown | | Unknown | | Unknown | | No | | Unknown | | Unknown | | Unknown | | Unknown | | Unknown |
| Samoa | No | | No | | Unknown | | Unknown | | Unknown | | Unknown | | Unknown | | Unknown | | Unknown | | Unknown | | Unknown |
| Solomon Islands | No | | No | | Unknown | | Unknown | | Unknown | | Unknown | | Unknown | | Unknown | | Unknown | | Unknown | | Unknown |
| Tonga | No | | No | | Unknown | | Unknown | | Unknown | | Unknown | | Unknown | | Unknown | | Unknown | | Unknown | | Unknown |
| Tuvalu | No | | No | | Unknown | | Unknown | | Unknown | | Unknown | | Unknown | | Unknown | | Unknown | | Unknown | | Unknown |
| Vanuatu | No | | No | | Unknown | | Unknown | | Unknown | | Unknown | | Unknown | | Unknown | | Unknown | | Unknown | | Unknown |
| **Australasia** |  | |  | |  | |  | |  | |  | |  | |  | |  | |  | |  |
| Australia | One or more | | No | | One or more | | One or more | | One or more | | One or more | | One or more | | One or more | | One or more | | One or more | | All facilities |
| New Zealand | All facilities | | Unknown | | Unknown | | One or more | | Unknown | | Unknown | | Unknown | | Unknown | | Unknown | | Unknown | | Unknown |
| **Sub Saharan Africa** | |  | |  | |  | |  | |  | |  | |  | |  | |  | |  | |
| Angola | No | | No | | Unknown | | One or more | | Unknown | | No | | Unknown | | Unknown | | Unknown | | Unknown | | Unknown |
| Benin | No | | Unknown | | Unknown | | Unknown | | Unknown | | Unknown | | Unknown | | Unknown | | Unknown | | Unknown | | Unknown |
| Botswana | No | | No | | Unknown | | Unknown | | Unknown | | Unknown | | Unknown | | Unknown | | Unknown | | Unknown | | Unknown |
| Burkina Faso | No | | No | | Unknown | | Unknown | | Unknown | | Unknown | | Unknown | | Unknown | | Unknown | | Unknown | | Unknown |
| Burundi | No | | No | | Unknown | | Unknown | | Unknown | | Unknown | | Unknown | | Unknown | | Unknown | | Unknown | | Unknown |
| Cameroon | No | | No | | One or more | | Unknown | | One or more | | Unknown | | One or more | | Unknown | | One or more | | Unknown | | Unknown |
| Cabo Verde | No | | No | | Unknown | | Unknown | | Unknown | | Unknown | | Unknown | | Unknown | | Unknown | | Unknown | | Unknown |
| Central African Republic | No | | No | | Unknown | | Unknown | | Unknown | | Unknown | | Unknown | | Unknown | | Unknown | | Unknown | | Unknown |
| Chad | No | | No | | Unknown | | Unknown | | Unknown | | Unknown | | One or more | | One or more | | Unknown | | Unknown | | Unknown |
| Comoros | No | | No | | Unknown | | Unknown | | Unknown | | Unknown | | Unknown | | Unknown | | Unknown | | Unknown | | Unknown |
| Côte d'Ivoire | Unknown | | No | | Unknown | | One or more | | Unknown | | Unknown | | One or more | | One or more | | Unknown | | Unknown | | Unknown |
| Democratic Republic of the Congo | No | | No | | Unknown | | Unknown | | Unknown | | Unknown | | Unknown | | Unknown | | Unknown | | Unknown | | Unknown |
| Djibouti | No | | No | | Unknown | | Unknown | | Unknown | | Unknown | | Unknown | | Unknown | | Unknown | | Unknown | | Unknown |
| Equatorial Guinea | No | | No | | Unknown | | Unknown | | Unknown | | Unknown | | Unknown | | Unknown | | Unknown | | Unknown | | Unknown |
| Eritrea | No | | No | | Unknown | | Unknown | | Unknown | | Unknown | | Unknown | | Unknown | | Unknown | | Unknown | | Unknown |
| Eswatini | No | | No | | Unknown | | Unknown | | Unknown | | Unknown | | One or more | | Unknown | | Unknown | | Unknown | | Unknown |
| Ethiopia | No | | No | | Unknown | | One or more | | Unknown | | Unknown | | One or more | | Unknown | | Unknown | | Unknown | | Unknown |
| Gabon | No | | No | | Unknown | | Unknown | | Unknown | | Unknown | | Unknown | | Unknown | | Unknown | | Unknown | | Unknown |
| Gambia | No | | No | | Unknown | | Unknown | | Unknown | | No | | One or more | | Unknown | | Unknown | | Unknown | | Unknown |
| Ghana | Unknown | | No | | Unknown | | Unknown | | Unknown | | No | | One or more | | Unknown | | Unknown | | Unknown | | Unknown |
| Guinea | No | | No | | Unknown | | Unknown | | Unknown | | Unknown | | Unknown | | One or more | | Unknown | | Unknown | | Unknown |
| Guinea-Bissau | No | | No | | Unknown | | Unknown | | Unknown | | Unknown | | Unknown | | Unknown | | Unknown | | Unknown | | Unknown |
| Kenya | One or more | | No | | Unknown | | One or more | | Unknown | | Unknown | | Unknown | | Unknown | | Unknown | | Unknown | | Unknown |
| Lesotho | No | | No | | Unknown | | One or more | | Unknown | | Unknown | | Unknown | | Unknown | | Unknown | | Unknown | | Unknown |
| Liberia | Unknown | | No | | Unknown | | Unknown | | Unknown | | Unknown | | Unknown | | Unknown | | Unknown | | Unknown | | Unknown |
| Madagascar | Unknown | | Unknown | | Unknown | | Unknown | | Unknown | | Unknown | | One or more | | One or more | | Unknown | | Unknown | | Unknown |
| Malawi | No | | No | | One or more | | One or more | | Unknown | | Unknown | | One or more | | One or more | | Unknown | | Unknown | | Unknown |
| Mali | No | | Unknown | | Unknown | | Unknown | | Unknown | | Unknown | | Unknown | | Unknown | | Unknown | | Unknown | | Unknown |
| Mauritania | No | | No | | Unknown | | Unknown | | Unknown | | Unknown | | Unknown | | Unknown | | Unknown | | Unknown | | Unknown |
| Mauritius | One or more | | Unknown | | One or more | | One or more | | Unknown | | Unknown | | Unknown | | Unknown | | Unknown | | Unknown | | Unknown |
| Mozambique | Unknown | | Unknown | | One or more | | One or more | | Unknown | | Unknown | | Unknown | | Unknown | | Unknown | | Unknown | | Unknown |
| Namibia | No | | No | | Unknown | | Unknown | | Unknown | | Unknown | | Unknown | | Unknown | | Unknown | | Unknown | | Unknown |
| Niger | No | | No | | Unknown | | Unknown | | Unknown | | Unknown | | One or more | | One or more | | Unknown | | Unknown | | Unknown |
| Nigeria | No | | Unknown | | Unknown | | Unknown | | Unknown | | Unknown | | One or more | | One or more | | No | | No | | No |
| Congo | No | | No | | Unknown | | Unknown | | Unknown | | Unknown | | Unknown | | Unknown | | Unknown | | Unknown | | Unknown |
| Rwanda | No | | No | | Unknown | | Unknown | | Unknown | | Unknown | | One or more | | One or more | | Unknown | | Unknown | | Unknown |
| Sao Tome & Principe | No | | No | | Unknown | | Unknown | | Unknown | | Unknown | | Unknown | | Unknown | | Unknown | | Unknown | | Unknown |
| Senegal | Unknown | | Unknown | | Unknown | | Unknown | | Unknown | | Unknown | | One or more | | One or more | | Unknown | | Unknown | | Unknown |
| Seychelles | Unknown | | Unknown | | Unknown | | Unknown | | Unknown | | Unknown | | Unknown | | Unknown | | Unknown | | Unknown | | Unknown |
| Sierra Leone | No | | Unknown | | Unknown | | Unknown | | Unknown | | Unknown | | One or more | | One or more | | Unknown | | Unknown | | Unknown |
| Somalia | No | | No | | Unknown | | Unknown | | Unknown | | Unknown | | One or more | | Unknown | | Unknown | | Unknown | | Unknown |
| South Africa | No | | No | | One or more | | One or more | | Unknown | | Unknown | | All facilities | | One or more | | Unknown | | Unknown | | Unknown |
| United Republic of Tanzania | One or more | | Unknown | | Unknown | | Unknown | | Unknown | | Unknown | | One or more | | Unknown | | Unknown | | Unknown | | Unknown |
| Togo | No | | No | | Unknown | | Unknown | | Unknown | | Unknown | | Unknown | | Unknown | | Unknown | | Unknown | | Unknown |
| Uganda | Unknown | | Unknown | | All facilities | | No | | Unknown | | Unknown | | All facilities | | All facilities | | Unknown | | Unknown | | Unknown |
| Zambia | No | | No | | All facilities | | One or more | | Unknown | | Unknown | | One or more | | One or more | | Unknown | | Unknown | | Unknown |
| Zimbabwe | No | | No | | Unknown | | Unknown | | Unknown | | Unknown | | One or more | | All facilities | | Unknown | | Unknown | | Unknown |
| **Middle East & North Africa** | |  | |  | |  | |  | |  | |  | |  | |  | |  | |  | |
| Algeria | No | | Unknown | | Unknown | | Unknown | | Unknown | | Unknown | | Unknown | | Unknown | | Unknown | | Unknown | | Unknown |
| Bahrain | No | | No | | Unknown | | Unknown | | Unknown | | Unknown | | Unknown | | Unknown | | Unknown | | Unknown | | Unknown |
| Cyprus | One or more | | No | | All facilities | | One or more | | All facilities | | One or more | | All facilities | | One or more | | All facilities | | All facilities | | All facilities |
| Egypt | No | | Unknown | | One or more | | One or more | | One or more | | Unknown | | One or more | | Unknown | | One or more | | Unknown | | Unknown |
| Iraq | No | | No | | One or more | | Unknown | | Unknown | | Unknown | | Unknown | | Unknown | | Unknown | | Unknown | | Unknown |
| Israel | One or more | | Unknown | | All facilities | | Unknown | | Unknown | | Unknown | | Unknown | | Unknown | | Unknown | | Unknown | | Unknown |
| Jordan | No | | Unknown | | Unknown | | Unknown | | Unknown | | Unknown | | Unknown | | Unknown | | Unknown | | Unknown | | Unknown |
| Kuwait | No | | No | | Unknown | | Unknown | | All facilities | | All facilities | | Unknown | | Unknown | | Unknown | | Unknown | | Unknown |
| Lebanon | Unknown | | Unknown | | One or more | | Unknown | | Unknown | | Unknown | | Unknown | | Unknown | | One or more | | Unknown | | Unknown |
| Libya | No | | No | | One or more | | One or more | | Unknown | | Unknown | | Unknown | | Unknown | | Unknown | | Unknown | | Unknown |
| Morocco | One or more | | No | | All facilities | | One or more | | Unknown | | One or more | | Unknown | | Unknown | | Unknown | | Unknown | | Unknown |
| Oman | No | | No | | Unknown | | Unknown | | Unknown | | Unknown | | Unknown | | Unknown | | Unknown | | Unknown | | Unknown |
| Occupied Palestinian territories | Unknown | | Unknown | | Unknown | | Unknown | | Unknown | | No | | Unknown | | Unknown | | Unknown | | Unknown | | Unknown |
| Qatar | No | | No | | Unknown | | Unknown | | Unknown | | Unknown | | Unknown | | Unknown | | Unknown | | Unknown | | Unknown |
| Saudi Arabia | No | | No | | Unknown | | Unknown | | Unknown | | Unknown | | Unknown | | Unknown | | Unknown | | Unknown | | Unknown |
| South Sudan | No | | No | | Unknown | | Unknown | | Unknown | | Unknown | | Unknown | | Unknown | | Unknown | | Unknown | | Unknown |
| Sudan | No | | No | | Unknown | | Unknown | | Unknown | | Unknown | | Unknown | | Unknown | | Unknown | | Unknown | | Unknown |
| Syrian Arab Republic | No | | No | | Unknown | | Unknown | | Unknown | | Unknown | | Unknown | | Unknown | | Unknown | | Unknown | | Unknown |
| Tunisia | No | | Unknown | | Unknown | | Unknown | | Unknown | | Unknown | | Unknown | | Unknown | | Unknown | | Unknown | | Unknown |
| Türkiye | One or more | | No | | One or more | | One or more | | One or more | | One or more | | One or more | | One or more | | One or more | | One or more | | One or more |
| United Arab Emirates | No | | No | | Unknown | | Unknown | | Unknown | | Unknown | | Unknown | | Unknown | | Unknown | | Unknown | | Unknown |
| Yemen | No | | No | | Unknown | | Unknown | | Unknown | | Unknown | | Unknown | | Unknown | | Unknown | | Unknown | | Unknown |

# Appendix 9: People who provided data or advice regarding the reviews

**Many thanks to the following individuals who assisted with extraction, data, clarification or contacts for information:**

Mika Rautanen, Stuart A Kinner, Tanya Deshkno, Revati Chawta, Brooke Hollingshead, Claude Scheidegger, Andrew Scheibe, Irma Kirtatdze, Karla Thorton, Erika Duffell, Brian Eastwood, Monica Desai, Roberto Muga, Sonia Arias Garcia, Carole Devaux, Cielo Rios, Giovanni de Girolamo, Ólafsson Sigurður, Nadine Kronfli, Rodrigo Feliciano do Carmo, Nadine Berndt, Gráinne Cousins, Arkadiusz Komorowski, Tanja Schwarz, Els Plettinckx, Brigita Rasimaite, Josipa Andreić, Ludmila Carapinha, Diāna Vanaga-Arāja, Begoña Brime Beteta, Afarin Rahimi-Movaghar, Gail Matthews, Sharon Hutchinson, Deborah Sidall, Kelly Hosking, Holly Beasley, David Grace, Andrew Wiley, Graham Kraak, Annick Bórquez, Filippo Pericoli, Tariq Sonnan, Ruth Zimmerman, Francisca Fonseca, Marianne Martinello, Kristín I. Pálsdóttir, Susan Wambui, Rehema Mjindo, Steve Willner, Chantal Edge, Sarah Evans, Naomi Burke-Shyne, Triona Lenihan, Ajeng Larasati, Marcela Jofre, Giada Girelli, Cinzia Brentari, Josefina Mavrou, Katerina Grohmannova, Caroline Protais, Franziska Schneider, Bálint Réka, Roberta Potente, Daan van der Gouwe, Ruxanda Iliescu, Viviana Manolache, Ines Kvaternik, Ioulia Bafi, Annette Verster, Ehsanullah Ehsan, Elisabeth Loue-Andrée Embinga, Eriobu Nnakelu, Huan-Keat Chan, Imam Waked, Jeffrey Lazarus, Jens Reimer, Joe Rich, Jorge Sánchez, Keith Humphreys, Kimberly Page, Maria Elena Medina-Mora, Nigel Raymond, Paul Griffiths, Philip Bruggmann, Sergey Biryukov, Sunil Solomon.

# Appendix 10: Study quality tables for included studies for each indicator: NSPs, OAT, HIV, HCV, HBV and tuberculosis

## *Appendix Table 10.1: Source characteristics for any Opioid Agonist Treatment (OAT) coverage in carceral settings by country*

| **Country** | **Available** | **Year** | Source Scope (National, Subnational, etc.) | Any client or facility-level information available? (Yes/No) | Client Numbers Reported? | Client Number Year | Facility Numbers Reported? | Facility Number Year |
| --- | --- | --- | --- | --- | --- | --- | --- | --- |
| **Eastern Europe** |  |  |  |  |  |  |  |  |
| Armenia | Yes | 2024 | National | No | No | ·· | No | ·· |
| Azerbaijan | No | 2024 | National | No | No | ·· | No | ·· |
| Belarus | No | 2024 | National | No | No | ·· | No | ·· |
| Bosnia & Herzegovina | Yes | 2015 | National | Yes | No | ·· | Yes | 2015 |
| Bulgaria | Yes | 2022 | National | No | No | ·· | No | ·· |
| Czechia | Yes | 2024 | National | Yes | Yes | 2021 | No | ·· |
| Estonia | Yes | 2024 | National | No | No | ·· | No | ·· |
| Georgia | Yes | 2013 | Unspecified | Yes | Yes | 2020 | No | ·· |
| Hungary | No | 2024 | National | No | No | ·· | No | ·· |
| Latvia | Yes | 2022 | National | Yes | Yes | 2016 | No | ·· |
| Lithuania | All facilities | 2022 | National | No | No | ·· | No | ·· |
| Republic of Moldova | Yes | 2022 | National | Yes | Yes | 2022 | Yes | 2022 |
| Poland | Yes | 2024 | National | Yes | Yes | 2017 | No | ·· |
| Romania | Yes | 2024 | National | Yes | Yes | 2017 | No | ·· |
| Russian Federation | No | 2023 | National | No | No | ·· | No | ·· |
| Slovakia | No | 2022 | National | No | No | ·· | No | ·· |
| Ukraine | Yes | 2024 | National | Yes | Yes | 2020 | No | ·· |
| **Western Europe** |  |  |  |  |  |  |  |  |
| Albania | Yes | 2017 | National | No | No | ·· | No | ·· |
| Andorra | No | 2023 | Unspecified | No | No | ·· | No | ·· |
| Austria | All facilities | 2022 | National | Yes | Yes | 2022 | Yes | 2022 |
| Belgium | All facilities | 2022 | National | Yes | Yes | 2016 | No | ·· |
| Croatia | All facilities | 2022 | National | Yes | Yes | 2016 | Yes | 2022 |
| Denmark | All facilities | 2022 | National | Yes | Yes | 2015 | No | ·· |
| England | All facilities | 2023 | National | Yes | Yes | 2023 | No | ·· |
| Finland | All facilities | 2022 | National | Yes | Yes | 2016 | No | ·· |
| France | All facilities | 2022 | National | Yes | Yes | 2013 | No | ·· |
| Germany | All facilities | 2022 | National | Yes | Yes | 2011 | No | ·· |
| Greece | Yes | 2024 | National | Yes | Yes | 2016 | No | ·· |
| Greenland | No | 2023 | Unspecified | No | No | ·· | No | ·· |
| Iceland | Yes | 2024 | Unspecified | No | No | ·· | No | ·· |
| Ireland | Yes | 2022 | Unspecified | Yes | Yes | 2016 | No | ·· |
| Italy | Yes | 2022 | National | No | No | ·· | No | ·· |
| Liechtenstein | No | 2023 | Unspecified | No | No | ·· | No | ·· |
| Luxembourg | Yes | 2022 | National | Yes | Yes | 2021 | No | ·· |
| Malta | Yes | 2022 | National | No | No | ·· | No | ·· |
| Monaco | Unknown | ·· | ·· | ·· | ·· | ·· | ·· | ·· |
| Montenegro | Yes | 2010 | National | No | No | ·· | No | ·· |
| Netherlands | Yes | 2021 | National | Yes | Yes | 2014 | No | ·· |
| North Macedonia | All facilities | 2017 | National | Yes | Yes | 2010 | No | ·· |
| Northern Ireland | All facilities | 2018 | National | Yes | Yes | 2016 | No | ·· |
| Norway | All facilities | 2022 | National | Yes | Yes | 2016 | No | ·· |
| Portugal | All facilities | 2022 | National | Yes | Yes | 2016 | Yes | 2022 |
| San Marino | Unknown | ·· | ·· | ·· | ·· | ·· | ·· | ·· |
| Scotland | All facilities | 2021 | National | Yes | Yes | 2021 | No | ·· |
| Serbia | All facilities | 2017 | National | Yes | Yes | 2013 | No | ·· |
| Slovenia | Yes | 2022 | National | Yes | Yes | 2020 | No | ·· |
| Spain | All facilities | 2022 | National | Yes | Yes | 2016 | No | ·· |
| Sweden | Yes | 2022 | National | No | No | ·· | No | ·· |
| Switzerland | Yes | 2013 | National | No | No | ·· | No | ·· |
| Wales | All facilities | 2018 | National | No | No | ·· | No | ·· |
| **East and South East Asia** |  |  |  |  |  |  |  |  |
| Brunei Darussalam | No | 2023 | National | No | No | ·· | No | ·· |
| Cambodia | Unknown | ·· | ·· | ·· | ·· | ·· | ·· | ·· |
| China | Unknown | ·· | ·· | ·· | ·· | ·· | ·· | ·· |
| Hong Kong† | Unknown | ·· | ·· | ·· | ·· | ·· | ·· | ·· |
| Indonesia | Yes | 2018 | Unspecified | Yes | No | ·· | Yes | 2018 |
| Japan | No | 2023 | National | No | No | ·· | No | ·· |
| Lao People's Democratic Republic | No | 2023 | National | No | No | ·· | No | ·· |
| Malaysia | Yes | 2014 | National | Yes | No | ·· | Yes | 2014 |
| Mongolia | No | 2023 | National | No | No | ·· | No | ·· |
| Myanmar | No | 2020 | National | No | No | ·· | No | ·· |
| Democratic People's Republic of Korea | No | 2023 | Unspecified | No | No | ·· | No | ·· |
| Philippines | No | 2023 | National | No | No | ·· | No | ·· |
| Singapore | No | 2023 | National | No | No | ·· | No | ·· |
| Republic of Korea | No | 2023 | National | No | No | ·· | No | ·· |
| Taiwan† | Unknown | ·· | ·· | ·· | ·· | ·· | ·· | ·· |
| Thailand | Unknown | ·· | ·· | ·· | ·· | ·· | ·· | ·· |
| Timor-Leste | No | 2023 | National | No | No | ·· | No | ·· |
| Viet Nam | Yes | 2020 | Unspecified | No | No | ·· | No | ·· |
| **South Asia** |  |  |  |  |  |  |  |  |
| Afghanistan | Unknown | ·· | ·· | ·· | ·· | ·· | ·· | ·· |
| Bangladesh | Unknown | ·· | ·· | ·· | ·· | ·· | ·· | ·· |
| Bhutan | No | 2023 | National | No | No | ·· | No | ·· |
| India | Yes | 2024 | National | No | No | ·· | No | ·· |
| Islamic Republic of Iran | All facilities | 2024 | National | Yes | Yes | 2019 | No | ·· |
| Maldives | Unknown | ·· | ·· | ·· | ·· | ·· | ·· | ·· |
| Nepal | Unknown | ·· | ·· | ·· | ·· | ·· | ·· | ·· |
| Pakistan | No | 2023 | National | No | No | ·· | No | ·· |
| Sri Lanka | No | 2023 | National | No | No | ·· | No | ·· |
| **Central Asia** |  |  |  |  |  |  |  |  |
| Kazakhstan | No | 2024 | National | No | No | ·· | No | ·· |
| Kyrgyzstan | Yes | 2023 | National | Yes | Yes | 2023 | Yes | 2023 |
| Tajikistan | Yes | 2024 | National | Yes | Yes | 2024 | No | ·· |
| Turkmenistan | No | 2023 | National | No | No | ·· | No | ·· |
| Uzbekistan | No | 2014 | National | No | No | ·· | No | ·· |
| **Caribbean** |  |  |  |  |  |  |  |  |
| Antigua & Barbuda | No | 2023 | National | No | No | ·· | No | ·· |
| Bahamas | No | 2023 | National | No | No | ·· | No | ·· |
| Barbados | No | 2023 | National | No | No | ·· | No | ·· |
| Bermuda | No | 2023 | Unspecified | No | No | ·· | No | ·· |
| Cuba | No | 2023 | National | No | No | ·· | No | ·· |
| Dominica | No | 2023 | National | No | No | ·· | No | ·· |
| Dominican Republic | Unknown | ·· | ·· | ·· | ·· | ·· | ·· | ·· |
| Grenada | No | 2023 | National | No | No | ·· | No | ·· |
| Haiti | No | 2023 | National | No | No | ·· | No | ·· |
| Jamaica | No | 2023 | National | No | No | ·· | No | ·· |
| Commonwealth of Puerto Rico | Unknown | ·· | ·· | ·· | ·· | ·· | ·· | ·· |
| Saint Kitts & Nevis | No | 2023 | National | No | No | ·· | No | ·· |
| Saint Lucia | No | 2023 | National | No | No | ·· | No | ·· |
| Saint Vincent & the Grenadines | No | 2023 | National | No | No | ·· | No | ·· |
| Trinidad & Tobago | No | 2023 | National | No | No | ·· | No | ·· |
| **Latin America** |  |  |  |  |  |  |  |  |
| Argentina | Unknown | ·· | ·· | ·· | ·· | ·· | ·· | ·· |
| Belize | No | 2023 | National | No | No | ·· | No | ·· |
| Plurinational State of Bolivia | No | 2023 | National | No | No | ·· | No | ·· |
| Brazil | No | 2023 | National | No | No | ·· | No | ·· |
| Chile | No | 2023 | National | No | No | ·· | No | ·· |
| Colombia | Yes | 2024 | National | No | No | ·· | No | ·· |
| Costa Rica | No | 2023 | National | No | No | ·· | No | ·· |
| Ecuador | No | 2023 | National | No | No | ·· | No | ·· |
| El Salvador | No | 2023 | National | No | No | ·· | No | ·· |
| Guatemala | No | 2023 | National | No | No | ·· | No | ·· |
| Guyana | No | 2023 | National | No | No | ·· | No | ·· |
| Honduras | No | 2023 | National | No | No | ·· | No | ·· |
| Mexico | No | 2024 | National | No | No | ·· | No | ·· |
| Nicaragua | No | 2023 | National | No | No | ·· | No | ·· |
| Panama | No | 2023 | National | No | No | ·· | No | ·· |
| Paraguay | No | 2023 | National | No | No | ·· | No | ·· |
| Peru | No | 2023 | National | No | No | ·· | No | ·· |
| Suriname | No | 2023 | National | No | No | ·· | No | ·· |
| Uruguay | No | 2023 | National | No | No | ·· | No | ·· |
| Bolivarian Republic of Venezuela | No | 2023 | National | No | No | ·· | No | ·· |
| **North America** |  |  |  |  |  |  |  |  |
| Canada (Federal) | All facilities | 2023 | National | Yes | Yes | 2023 | Yes | 2023 |
| Canada (Provincial) | Yes | 2020 | National | Yes | No | ·· | Yes | 2020 |
| United States of America (Federal) | Unknown | ·· | ·· | ·· | ·· | ·· | ·· | ·· |
| United States of America (State) | Yes | 2021 | National | Yes | Yes | 2019 | No | ·· |
| **Pacific Island States & Terr.** |  |  |  |  |  |  |  |  |
| American Samoa | No | 2023 | Unspecified | No | No | ·· | No | ·· |
| Federated States of Micronesia | No | 2023 | Unspecified | No | No | ·· | No | ·· |
| Fiji | No | 2023 | National | No | No | ·· | No | ·· |
| French Polynesia | No | 2023 | Unspecified | No | No | ·· | No | ·· |
| Guam | No | 2023 | Unspecified | No | No | ·· | No | ·· |
| Kiribati | No | 2023 | Unspecified | No | No | ·· | No | ·· |
| Marshall Islands | No | 2023 | Unspecified | No | No | ·· | No | ·· |
| Nauru | No | 2023 | Unspecified | No | No | ·· | No | ·· |
| New Caledonia | No | 2023 | Unspecified | No | No | ·· | No | ·· |
| Northern Mariana Islands | No | 2023 | Unspecified | No | No | ·· | No | ·· |
| Palau | No | 2023 | Unspecified | No | No | ·· | No | ·· |
| Papua New Guinea | No | 2023 | National | No | No | ·· | No | ·· |
| Samoa | No | 2023 | Unspecified | No | No | ·· | No | ·· |
| Solomon Islands | No | 2023 | Unspecified | No | No | ·· | No | ·· |
| Tonga | No | 2023 | Unspecified | No | No | ·· | No | ·· |
| Tuvalu | No | 2023 | Unspecified | No | No | ·· | No | ·· |
| Vanuatu | No | 2023 | Unspecified | No | No | ·· | No | ·· |
| **Australasia** |  |  |  |  |  |  |  |  |
| Australia | Yes | 2023 | National | Yes | Yes | 2023 | No | ·· |
| New Zealand | All facilities | 2021 | National | Yes | Yes | 2021 | No | ·· |
| **Sub Saharan Africa** |  |  |  |  |  |  |  |  |
| Angola | No | 2023 | National | No | No | ·· | No | ·· |
| Benin | No | 2023 | National | No | No | ·· | No | ·· |
| Botswana | No | 2023 | National | No | No | ·· | No | ·· |
| Burkina Faso | No | 2023 | National | No | No | ·· | No | ·· |
| Burundi | No | 2023 | National | No | No | ·· | No | ·· |
| Cameroon | No | 2023 | National | No | No | ·· | No | ·· |
| Cabo Verde | No | 2023 | National | No | No | ·· | No | ·· |
| Central African Republic | No | 2023 | National | No | No | ·· | No | ·· |
| Chad | No | 2023 | National | No | No | ·· | No | ·· |
| Comoros | No | 2023 | National | No | No | ·· | No | ·· |
| Côte d'Ivoire | Unknown | ·· | ·· | ·· | ·· | ·· | ·· | ·· |
| Democratic Republic of the Congo | No | 2023 | National | No | No | ·· | No | ·· |
| Djibouti | No | 2023 | National | No | No | ·· | No | ·· |
| Equatorial Guinea | No | 2023 | National | No | No | ·· | No | ·· |
| Eritrea | No | 2023 | National | No | No | ·· | No | ·· |
| Eswatini | No | 2023 | National | No | No | ·· | No | ·· |
| Ethiopia | No | 2023 | National | No | No | ·· | No | ·· |
| Gabon | No | 2023 | National | No | No | ·· | No | ·· |
| Gambia | No | 2023 | National | No | No | ·· | No | ·· |
| Ghana | Unknown | ·· | ·· | ·· | ·· | ·· | ·· | ·· |
| Guinea | No | 2023 | National | No | No | ·· | No | ·· |
| Guinea··Bissau | No | 2023 | National | No | No | ·· | No | ·· |
| Kenya | Yes | 2024 | Unspecified | No | No | ·· | No | ·· |
| Lesotho | No | 2023 | National | No | No | ·· | No | ·· |
| Liberia | Unknown | ·· | ·· | ·· | ·· | ·· | ·· | ·· |
| Madagascar | Unknown | ·· | ·· | ·· | ·· | ·· | ·· | ·· |
| Malawi | No | 2023 | National | No | No | ·· | No | ·· |
| Mali | No | 2023 | National | No | No | ·· | No | ·· |
| Mauritania | No | 2023 | National | No | No | ·· | No | ·· |
| Mauritius | Yes | 2019 | National | Yes | Yes | 2019 | Yes | 2019 |
| Mozambique | Unknown | ·· | ·· | ·· | ·· | ·· | ·· | ·· |
| Namibia | No | 2023 | National | No | No | ·· | No | ·· |
| Niger | No | 2023 | National | No | No | ·· | No | ·· |
| Nigeria | No | 2023 | National | No | No | ·· | No | ·· |
| Congo | No | 2023 | National | No | No | ·· | No | ·· |
| Rwanda | No | 2023 | National | No | No | ·· | No | ·· |
| Sao Tome & Principe | No | 2023 | National | No | No | ·· | No | ·· |
| Senegal | Unknown | ·· | ·· | ·· | ·· | ·· | ·· | ·· |
| Seychelles | Unknown | ·· | ·· | ·· | ·· | ·· | ·· | ·· |
| Sierra Leone | No | 2023 | National | No | No | ·· | No | ·· |
| Somalia | No | 2023 | National | No | No | ·· | No | ·· |
| South Africa | No | 2020 | National | No | No | ·· | No | ·· |
| United Republic of Tanzania | Yes | 2024 | National | No | No | ·· | No | ·· |
| Togo | No | 2023 | National | No | No | ·· | No | ·· |
| Uganda | Unknown | ·· | ·· | ·· | ·· | ·· | ·· | ·· |
| Zambia | No | 2023 | National | No | No | ·· | No | ·· |
| Zimbabwe | No | 2023 | National | No | No | ·· | No | ·· |
| **Middle East & North Africa** |  |  |  |  |  |  |  |  |
| Algeria | No | 2023 | National | No | No | ·· | No | ·· |
| Bahrain | No | 2023 | National | No | No | ·· | No | ·· |
| Cyprus | Yes | 2022 | National | No | No | ·· | No | ·· |
| Egypt | No | 2024 | National | No | No | ·· | No | ·· |
| Iraq | No | 2023 | National | No | No | ·· | No | ·· |
| Israel | Yes | 2015 | National | Yes | Yes | 2015 | Yes | 2015 |
| Jordan | No | 2023 | National | No | No | ·· | No | ·· |
| Kuwait | No | 2023 | National | No | No | ·· | No | ·· |
| Lebanon | Unknown | ·· | ·· | ·· | ·· | ·· | ·· | ·· |
| Libya | No | 2023 | National | No | No | ·· | No | ·· |
| Morocco | Yes | 2021 | National | Yes | Yes | 2019 | No | ·· |
| Oman | No | 2023 | National | No | No | ·· | No | ·· |
| Occupied Palestinian territories | Unknown | ·· | ·· | ·· | ·· | ·· | ·· | ·· |
| Qatar | No | 2023 | National | No | No | ·· | No | ·· |
| Saudi Arabia | No | 2023 | National | No | No | ·· | No | ·· |
| South Sudan | No | 2023 | National | No | No | ·· | No | ·· |
| Sudan | No | 2023 | National | No | No | ·· | No | ·· |
| Syrian Arab Republic | No | 2023 | National | No | No | ·· | No | ·· |
| Tunisia | No | 2023 | National | No | No | ·· | No | ·· |
| Türkiye | Yes | 2022 | National | No | No | ·· | No | ·· |
| United Arab Emirates | No | 2023 | National | No | No | ·· | No | ·· |
| Yemen | No | 2023 | National | No | No | ·· | No | ·· |

## *Appendix Table 10.2*: Source characteristics for Needle Syringe Programs (NSPs) coverage in carceral settings by country

| **Country** | Availability | Year of Data Collection | Source Scope (National, Subnational, etc.) | Any client or facility-level information available? (Yes/No) | Client Numbers Reported? | Client Number Year | Facility Numbers Reported? | Facility Number Year |
| --- | --- | --- | --- | --- | --- | --- | --- | --- |
| **Eastern Europe** |  |  |  |  |  |  |  |  |
| Armenia | No | 2024 | National | No | No | ·· | No | ·· |
| Azerbaijan | No | 2024 | National | No | No | ·· | No | ·· |
| Belarus | No | 2024 | National | No | No | ·· | No | ·· |
| Bosnia & Herzegovina | No | 2020 | National | No | No | ·· | No | ·· |
| Bulgaria | No | 2022 | National | No | No | ·· | No | ·· |
| Czechia | No | 2022 | National | No | No | ·· | No | ·· |
| Estonia | No | 2022 | National | No | No | ·· | No | ·· |
| Georgia | No | 2020 | National | No | No | ·· | No | ·· |
| Hungary | No | 2024 | National | No | No | ·· | No | ·· |
| Latvia | No | 2022 | National | No | No | ·· | No | ·· |
| Lithuania | No | 2022 | National | No | No | ·· | No | ·· |
| Republic of Moldova | Yes | 2024 | National | No | No | ·· | No | ·· |
| Poland | No | 2022 | National | No | No | ·· | No | ·· |
| Romania | No | 2024 | National | No | No | ·· | No | ·· |
| Russian Federation | No | 2010 | National | No | No | ·· | No | ·· |
| Slovakia | No | 2022 | National | No | No | ·· | No | ·· |
| Ukraine | Yes | 2024 | National | No | No | ·· | No | ·· |
| **Western Europe** |  |  |  |  |  |  |  |  |
| Albania | No | 2020 | National | No | No | ·· | No | ·· |
| Andorra | No | 2023 | National | No | No | ·· | No | ·· |
| Austria | No | 2022 | National | No | No | ·· | No | ·· |
| Belgium | No | 2024 | National | No | No | ·· | No | ·· |
| Croatia | Unknown | ·· | ·· | ·· | ·· | ·· | ·· | ·· |
| Denmark | No | 2024 | National | No | No | ·· | No | ·· |
| England | No | 2020 | National | No | No | ·· | No | ·· |
| Finland | No | 2024 | National | No | No | ·· | No | ·· |
| France | No | 2024 | National | No | No | ·· | No | ·· |
| Germany | Yes | 2024 | National | No | No | ·· | No | ·· |
| Greece | No | 2024 | National | No | No | ·· | No | ·· |
| Greenland | No | 2023 | National | No | No | ·· | No | ·· |
| Iceland | Unknown | ·· | ·· | ·· | ·· | ·· | ·· | ·· |
| Ireland | No | 2022 | National | No | No | ·· | No | ·· |
| Italy | No | 2022 | National | No | No | ·· | No | ·· |
| Liechtenstein | No | 2023 | National | No | No | ·· | No | ·· |
| Luxembourg | All facilities | 2024 | National | Yes | Yes | 2021 | Yes | 2022 |
| Malta | No | 2022 | National | No | No | ·· | No | ·· |
| Monaco | No | 2023 | National | No | No | ·· | No | ·· |
| Montenegro | Unknown | ·· | ·· | ·· | ·· | ·· | ·· | ·· |
| Netherlands | No | 2022 | National | No | No | ·· | No | ·· |
| North Macedonia | No | 2024 | National | No | No | ·· | No | ·· |
| Northern Ireland | No | 2020 | National | No | No | ·· | No | ·· |
| Norway | No | 2024 | National | No | No | ·· | No | ·· |
| Portugal | No | 2024 | National | No | No | ·· | No | ·· |
| San Marino | All facilities | 2020 | National | No | No | ·· | No | ·· |
| Scotland | No | 2020 | National | No | No | ·· | No | ·· |
| Serbia | No | 2017 | National | No | No | ·· | No | ·· |
| Slovenia | No | 2022 | National | No | No | ·· | No | ·· |
| Spain | All facilities | 2024 | National | No | No | ·· | No | ·· |
| Sweden | No | 2024 | National | No | No | ·· | No | ·· |
| Switzerland | Yes | 2024 | National | Yes | Yes | 2010 | Yes | 2017 |
| Wales | No | 2020 | National | No | No | ·· | No | ·· |
| **East and South East Asia** |  |  |  |  |  |  |  |  |
| Brunei Darussalam | No | 2023 | National | No | No | ·· | No | ·· |
| Cambodia | Unknown | ·· | ·· | ·· | ·· | ·· | ·· | ·· |
| China | Unknown | ·· | ·· | ·· | ·· | ·· | ·· | ·· |
| Hong Kong† | No | 2023 | National | No | No | ·· | No | ·· |
| Indonesia | No | 2018 | National | No | No | ·· | No | ·· |
| Japan | No | 2023 | National | No | No | ·· | No | ·· |
| Lao People's Democratic Republic | Unknown | ·· | ·· | ·· | ·· | ·· | ·· | ·· |
| Malaysia | No | 2023 | National | No | No | ·· | No | ·· |
| Mongolia | Unknown | ·· | ·· | ·· | ·· | ·· | ·· | ·· |
| Myanmar | Unknown | ·· | ·· | ·· | ·· | ·· | ·· | ·· |
| Democratic People's Republic of Korea | No | 2023 | National | No | No | ·· | No | ·· |
| Philippines | No | 2023 | National | No | No | ·· | No | ·· |
| Singapore | No | 2023 | National | No | No | ·· | No | ·· |
| Republic of Korea | No | 2023 | National | No | No | ·· | No | ·· |
| Taiwan† | Unknown | ·· | ·· | ·· | ·· | ·· | ·· | ·· |
| Thailand | Unknown | ·· | ·· | ·· | ·· | ·· | ·· | ·· |
| Timor-Leste | No | 2023 | National | No | No | ·· | No | ·· |
| Viet Nam | Unknown | ·· | ·· | ·· | ·· | ·· | ·· | ·· |
| **South Asia** |  |  |  |  |  |  |  |  |
| Afghanistan | No | 2024 | National | No | No | ·· | No | ·· |
| Bangladesh | Unknown | ·· | ·· | ·· | ·· | ·· | ·· | ·· |
| Bhutan | No | 2023 | National | No | No | ·· | No | ·· |
| India | No | 2024 | National | No | No | ·· | No | ·· |
| Islamic Republic of Iran | No | 2024 | National | No | No | ·· | No | ·· |
| Maldives | No | 2023 | National | No | No | ·· | No | ·· |
| Nepal | Unknown | ·· | ·· | ·· | ·· | ·· | ·· | ·· |
| Pakistan | Unknown | ·· | ·· | ·· | ·· | ·· | ·· | ·· |
| Sri Lanka | No | 2023 | National | No | No | ·· | No | ·· |
| **Central Asia** |  |  |  |  |  |  |  |  |
| Kazakhstan | No | 2024 | National | No | No | ·· | No | ·· |
| Kyrgyzstan | Yes | 2024 | National | Yes | Yes | 2014 | Yes | 2014 |
| Tajikistan | Yes | 2024 | National | No | No | ·· | No | ·· |
| Turkmenistan | No | 2023 | National | No | No | ·· | No | ·· |
| Uzbekistan | No | 2010 | National | No | No | ·· | No | ·· |
| **Caribbean** |  |  |  |  |  |  |  |  |
| Antigua & Barbuda | No | 2023 | National | No | No | ·· | No | ·· |
| Bahamas | No | 2023 | National | No | No | ·· | No | ·· |
| Barbados | No | 2023 | National | No | No | ·· | No | ·· |
| Bermuda | No | 2023 | National | No | No | ·· | No | ·· |
| Cuba | No | 2023 | National | No | No | ·· | No | ·· |
| Dominica | No | 2023 | National | No | No | ·· | No | ·· |
| Dominican Republic | Unknown | ·· | ·· | ·· | ·· | ·· | ·· | ·· |
| Grenada | No | 2023 | National | No | No | ·· | No | ·· |
| Haiti | No | 2023 | National | No | No | ·· | No | ·· |
| Jamaica | No | 2023 | National | No | No | ·· | No | ·· |
| Commonwealth of Puerto Rico | Unknown | ·· | ·· | ·· | ·· | ·· | ·· | ·· |
| Saint Kitts & Nevis | No | 2023 | National | No | No | ·· | No | ·· |
| Saint Lucia | No | 2023 | National | No | No | ·· | No | ·· |
| Saint Vincent & the Grenadines | No | 2023 | National | No | No | ·· | No | ·· |
| Trinidad & Tobago | No | 2023 | National | No | No | ·· | No | ·· |
| **Latin America** |  |  |  |  |  |  |  |  |
| Argentina | No | 2023 | National | No | No | ·· | No | ·· |
| Belize | No | 2023 | National | No | No | ·· | No | ·· |
| Plurinational State of Bolivia | No | 2023 | National | No | No | ·· | No | ·· |
| Brazil | No | 2023 | National | No | No | ·· | No | ·· |
| Chile | No | 2023 | National | No | No | ·· | No | ·· |
| Colombia | No | 2024 | National | No | No | ·· | No | ·· |
| Costa Rica | No | 2023 | National | No | No | ·· | No | ·· |
| Ecuador | No | 2023 | National | No | No | ·· | No | ·· |
| El Salvador | No | 2023 | National | No | No | ·· | No | ·· |
| Guatemala | No | 2023 | National | No | No | ·· | No | ·· |
| Guyana | No | 2023 | National | No | No | ·· | No | ·· |
| Honduras | No | 2023 | National | No | No | ·· | No | ·· |
| Mexico | No | 2024 | National | No | No | ·· | No | ·· |
| Nicaragua | No | 2023 | National | No | No | ·· | No | ·· |
| Panama | No | 2023 | National | No | No | ·· | No | ·· |
| Paraguay | No | 2023 | National | No | No | ·· | No | ·· |
| Peru | No | 2023 | National | No | No | ·· | No | ·· |
| Suriname | No | 2023 | National | No | No | ·· | No | ·· |
| Uruguay | No | 2023 | National | No | No | ·· | No | ·· |
| Bolivarian Republic of Venezuela | No | 2023 | National | No | No | ·· | No | ·· |
| **North America** |  |  |  |  |  |  |  |  |
| Canada (Federal) | Yes | 2024 | National | Yes | Yes | 2022 | Yes | 2023 |
| Canada (Provincial) | No | 2024 | National | No | No | ·· | No | ·· |
| United States of America (Federal) | No | 2021 | National | No | No | ·· | No | ·· |
| United States of America (State) | No | 2021 | National | No | No | ·· | No | ·· |
| **Pacific Island States & Terr.** |  |  |  |  |  |  |  |  |
| American Samoa | No | 2023 | National | No | No | ·· | No | ·· |
| Federated States of Micronesia | No | 2023 | National | No | No | ·· | No | ·· |
| Fiji | No | 2023 | National | No | No | ·· | No | ·· |
| French Polynesia | No | 2023 | National | No | No | ·· | No | ·· |
| Guam | No | 2023 | National | No | No | ·· | No | ·· |
| Kiribati | No | 2023 | National | No | No | ·· | No | ·· |
| Marshall Islands | No | 2023 | National | No | No | ·· | No | ·· |
| Nauru | No | 2023 | National | No | No | ·· | No | ·· |
| New Caledonia | No | 2023 | National | No | No | ·· | No | ·· |
| Northern Mariana Islands | No | 2023 | National | No | No | ·· | No | ·· |
| Palau | No | 2023 | National | No | No | ·· | No | ·· |
| Papua New Guinea | No | 2023 | National | No | No | ·· | No | ·· |
| Samoa | No | 2023 | National | No | No | ·· | No | ·· |
| Solomon Islands | No | 2023 | National | No | No | ·· | No | ·· |
| Tonga | No | 2023 | National | No | No | ·· | No | ·· |
| Tuvalu | No | 2023 | National | No | No | ·· | No | ·· |
| Vanuatu | No | 2023 | National | No | No | ·· | No | ·· |
| **Australasia** |  |  |  |  |  |  |  |  |
| Australia | No | 2020 | National | No | No | ·· | No | ·· |
| New Zealand | Unknown | ·· | ·· | ·· | ·· | ·· | ·· | ·· |
| **Sub Saharan Africa** |  |  |  |  |  |  |  |  |
| Angola | No | 2023 | National | No | No | ·· | No | ·· |
| Benin | Unknown | ·· | ·· | ·· | ·· | ·· | ·· | ·· |
| Botswana | No | 2023 | National | No | No | ·· | No | ·· |
| Burkina Faso | No | 2023 | National | No | No | ·· | No | ·· |
| Burundi | No | 2023 | National | No | No | ·· | No | ·· |
| Cameroon | No | 2023 | National | No | No | ·· | No | ·· |
| Cabo Verde | No | 2023 | National | No | No | ·· | No | ·· |
| Central African Republic | No | 2023 | National | No | No | ·· | No | ·· |
| Chad | No | 2023 | National | No | No | ·· | No | ·· |
| Comoros | No | 2023 | National | No | No | ·· | No | ·· |
| Côte d'Ivoire | No | 2023 | National | No | No | ·· | No | ·· |
| Democratic Republic of the Congo | No | 2023 | National | No | No | ·· | No | ·· |
| Djibouti | No | 2023 | National | No | No | ·· | No | ·· |
| Equatorial Guinea | No | 2023 | National | No | No | ·· | No | ·· |
| Eritrea | No | 2023 | National | No | No | ·· | No | ·· |
| Eswatini | No | 2023 | National | No | No | ·· | No | ·· |
| Ethiopia | No | 2023 | National | No | No | ·· | No | ·· |
| Gabon | No | 2023 | National | No | No | ·· | No | ·· |
| Gambia | No | 2023 | National | No | No | ·· | No | ·· |
| Ghana | No | 2023 | National | No | No | ·· | No | ·· |
| Guinea | No | 2023 | National | No | No | ·· | No | ·· |
| Guinea··Bissau | No | 2023 | National | No | No | ·· | No | ·· |
| Kenya | No | 2012 | National | No | No | ·· | No | ·· |
| Lesotho | No | 2023 | National | No | No | ·· | No | ·· |
| Liberia | No | 2023 | National | No | No | ·· | No | ·· |
| Madagascar | Unknown | ·· | ·· | ·· | ·· | ·· | ·· | ·· |
| Malawi | No | 2023 | National | No | No | ·· | No | ·· |
| Mali | Unknown | ·· | ·· | ·· | ·· | ·· | ·· | ·· |
| Mauritania | No | 2023 | National | No | No | ·· | No | ·· |
| Mauritius | Unknown | ·· | ·· | ·· | ·· | ·· | ·· | ·· |
| Mozambique | Unknown | ·· | ·· | ·· | ·· | ·· | ·· | ·· |
| Namibia | No | 2023 | National | No | No | ·· | No | ·· |
| Niger | No | 2023 | National | No | No | ·· | No | ·· |
| Nigeria | Unknown | ·· | ·· | ·· | ·· | ·· | ·· | ·· |
| Congo | No | 2023 | National | No | No | ·· | No | ·· |
| Rwanda | No | 2023 | National | No | No | ·· | No | ·· |
| Sao Tome & Principe | No | 2023 | National | No | No | ·· | No | ·· |
| Senegal | Unknown | ·· | ·· | ·· | ·· | ·· | ·· | ·· |
| Seychelles | Unknown | ·· | ·· | ·· | ·· | ·· | ·· | ·· |
| Sierra Leone | Unknown | ·· | ·· | ·· | ·· | ·· | ·· | ·· |
| Somalia | No | 2023 | National | No | No | ·· | No | ·· |
| South Africa | No | 2020 | National | No | No | ·· | No | ·· |
| United Republic of Tanzania | Unknown | ·· | ·· | ·· | ·· | ·· | ·· | ·· |
| Togo | No | 2023 | National | No | No | ·· | No | ·· |
| Uganda | Unknown | ·· | ·· | ·· | ·· | ·· | ·· | ·· |
| Zambia | No | 2023 | National | No | No | ·· | No | ·· |
| Zimbabwe | No | 2023 | National | No | No | ·· | No | ·· |
| **Middle East & North Africa** |  |  |  |  |  |  |  |  |
| Algeria | Unknown | ·· | ·· | ·· | ·· | ·· | ·· | ·· |
| Bahrain | No | 2023 | National | No | No | ·· | No | ·· |
| Cyprus | No | 2023 | National | No | No | ·· | No | ·· |
| Egypt | Unknown | ·· | ·· | ·· | ·· | ·· | ·· | ·· |
| Iraq | No | 2023 | National | No | No | ·· | No | ·· |
| Israel | Unknown | ·· | ·· | ·· | ·· | ·· | ·· | ·· |
| Jordan | Unknown | ·· | ·· | ·· | ·· | ·· | ·· | ·· |
| Kuwait | No | 2023 | National | No | No | ·· | No | ·· |
| Lebanon | Unknown | ·· | ·· | ·· | ·· | ·· | ·· | ·· |
| Libya | No | 2023 | National | No | No | ·· | No | ·· |
| Morocco | No | 2021 | National | No | No | ·· | No | ·· |
| Oman | No | 2023 | National | No | No | ·· | No | ·· |
| Occupied Palestinian territories | Unknown | ·· | ·· | ·· | ·· | ·· | ·· | ·· |
| Qatar | No | 2023 | National | No | No | ·· | No | ·· |
| Saudi Arabia | No | 2023 | National | No | No | ·· | No | ·· |
| South Sudan | No | 2023 | National | No | No | ·· | No | ·· |
| Sudan | No | 2023 | National | No | No | ·· | No | ·· |
| Syrian Arab Republic | No | 2023 | National | No | No | ·· | No | ·· |
| Tunisia | Unknown | ·· | ·· | ·· | ·· | ·· | ·· | ·· |
| Türkiye | No | 2022 | National | No | No | ·· | No | ·· |
| United Arab Emirates | No | 2023 | National | No | No | ·· | No | ·· |
| Yemen | No | 2023 | National | No | No | ·· | No | ·· |

## *Appendix Table 10.3* Source characteristics for HIV Testing coverage in carceral settings by country

| **Country** | Availability | Year of Data Collection | Source Scope (National, Subnational, etc.) | Any client or facility-level information available? (Yes/No) | Client Numbers Reported? | Client Number Year | Facility Numbers Reported? | Facility Number Year |
| --- | --- | --- | --- | --- | --- | --- | --- | --- |
| **Eastern Europe** |  |  |  |  |  |  |  |  |
| Armenia | All facilities | 2020 | National | Yes | No | ·· | Yes | 2020 |
| Azerbaijan | Yes | 2013 | National | Yes | Yes | 2013 | No | ·· |
| Belarus | Unknown | ·· | ·· | ·· | ·· | ·· | ·· | ·· |
| Bosnia & Herzegovina | Yes | 2020 | National | No | No | ·· | No | ·· |
| Bulgaria | All facilities | 2022 | National | Yes | Yes | 2022 | Yes | 2022 |
| Czechia | All facilities | 2020 | National | Yes | No | ·· | Yes | 2020 |
| Estonia | All facilities | 2022 | National | Yes | No | ·· | Yes | 2022 |
| Georgia | All facilities | 2020 | National | Yes | No | ·· | Yes | 2020 |
| Hungary | Yes | 2022 | National | Yes | No | ·· | Yes | 2022 |
| Latvia | All facilities | 2022 | National | Yes | No | ·· | Yes | 2022 |
| Lithuania | Yes | 2022 | National | Yes | No | ·· | Yes | 2022 |
| Republic of Moldova | All facilities | 2021 | National | Yes | No | ·· | Yes | 2021 |
| Poland | All facilities | 2022 | National | Yes | No | ·· | Yes | 2022 |
| Romania | Yes | 2022 | National | No | No | ·· | No | ·· |
| Russian Federation | Yes | 2022 | National | No | No | ·· | No | ·· |
| Slovakia | All facilities | 2022 | National | Yes | No | ·· | Yes | 2022 |
| Ukraine | All facilities | 2020 | National | Yes | Yes | 2020 | Yes | 2020 |
| **Western Europe** |  |  |  |  |  |  |  |  |
| Albania | Yes | 2020 | National | No | No | ·· | No | ·· |
| Andorra | Unknown | ·· | ·· | ·· | ·· | ·· | ·· | ·· |
| Austria | All facilities | 2022 | National | Yes | No | ·· | Yes | ·· |
| Belgium | All facilities | 2020 | National | Yes | No | ·· | Yes | ·· |
| Croatia | All facilities | 2022 | National | Yes | No | ·· | Yes | ·· |
[truncated: 126,107 more chars]
